# Mechanochemistry‐Driven Borrowing Hydrogen Processes for Ru‐Catalyzed *N*‐Alkylation: A Pathway to Enhanced Sustainability and Efficiency

**DOI:** 10.1002/anie.202508050

**Published:** 2025-06-23

**Authors:** Sourav Behera, Dipak J. Fartade, Rita Mocci, Michela Matta, Lidia De Luca, Andrea Porcheddu

**Affiliations:** ^1^ Dipartimento di Scienze Chimiche e Geologiche Università degli Studi di Cagliari, Cittadella Universitaria Cagliari 09042 Italy; ^2^ Dipartimento di Scienze Chimiche Fisiche, Matematiche e Naturali, Università degli Studi di Sassari Via Vienna 2 Sassari 07100 Italy

**Keywords:** Borrowing hydrogenation, Green chemistry, Mechanocatalysis, *N*‐Alkylation, Ru‐MACHO

## Abstract

This study presents the first mechanochemical borrowing hydrogenation (BH) strategy, offering a direct and efficient route to *N*‐alkylated amines and heterocycles. This solvent‐free approach overcomes many challenges associated with conventional solution‐based syntheses, such as toxic reagents, inert atmospheres, high temperatures, lengthy reaction times, excessive catalyst loadings, and the use of solvents. By applying this method under mechanochemical conditions and employing a readily available ruthenium‐based catalyst, we achieved high conversions of a diverse set of primary amines and alcohols into *N*‐alkylated amines. Furthermore, kinetic isotope effect (*KIE*) studies and Hammett analyses provided key insights into the underlying reaction mechanism. Ultimately, this protocol expands synthetic possibilities by facilitating the preparation of heterocycles.

## Graphical abstract

This work pioneers a solvent‐free mechanochemical borrowing hydrogen (BH) strategy for the rapid and efficient *N*‐alkylation of amines and heterocycles. By removing harsh conditions and toxic reagents while employing a simple Ru catalyst, this approach redefines BH as a cleaner, more versatile, and sustainable platform for synthetic chemistry.

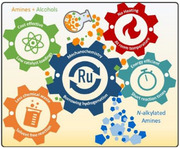

## Introduction

**Figure 1:**
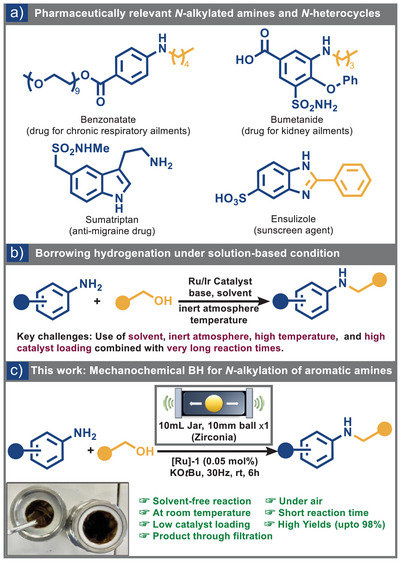
a) Some pharmaceutically relevant *N*‐alkylated amines and *N*‐heterocycles used in day‐to‐day life; b) Borrowing hydrogenation reaction for *N*‐alkylated amines under solution chemistry along with key challenges; c) This work: Mechanochemical BH reaction for the *N*‐alkylation of aromatic amines.

Nitrogen‐containing compounds, particularly *N‐*alkylated amines and *N*‐heterocycles, are crucial building blocks in the pharmaceutical and fine chemical industries (Figure 1a).^[^ ^1^, ^2^, ^3^, ^4^, ^5^ ^]^ Considering their critical importance in healthcare and medicine, creating effective strategies for forming C─N bonds is one of these industries' most desired and strategically vital transformations.^[^ ^6^, ^7^, ^8^, ^9^, ^10^ ^]^

Despite their widespread use, conventional protocols for *N*‐alkylation of primary amines present significant limitations.^[^ ^11^, ^12^, ^13^, ^14^ ^]^ These methods rely on dangerous alkyl halides, leading to poor atom economy, the generation of numerous over‐alkylated byproducts, and substantial stoichiometric waste.^[^ ^15^ ^]^ This compromises safety and results in a wasteful process that undermines efficiency and sustainability.^[^ ^16^ ^]^ In this context, alcohols have garnered significant interest due to their accessibility, cost‐effectiveness, and favorable environmental profile,^[^ ^17^, ^18^ ^]^ which includes origins from renewable and natural resources.^[^ ^19^, ^20^ ^]^ Notably, the direct use of alcohols as alkylating agents has been emphasized as a key goal in green chemistry literature.^[^ ^21^, ^22^ ^]^ Pioneering work by Watanabe,^[^ ^23^ ^]^ Grigg's,^[^ ^24^ ^]^ and Williams^[^ ^25^ ^]^ marked a paradigm shift by proving that alcohols can serve as effective alkylating agents for *N*‐alkylation and *N*‐heterocycle synthesis under metal catalysis. This breakthrough established the basis for “borrowing hydrogenation” (BH), which has since become a cornerstone of sustainable catalytic synthesis.^[^ ^26^, ^27^, ^28^, ^29^, ^30^ ^]^ BH strategy enhances safety, expands the scope of essential synthetic transformations, and offers a greener alternative by eliminating the need for carbon‐intensive hydrogen gas and pressurized cylinders.^[^ ^31^, ^32^ ^]^

The broad applicability of BH has spurred extensive exploration of homogeneous catalytic systems^[^ ^28^ ^]^ featuring noble metal complexes (Rh, Ru, Ir, Os, Pd),^[^ ^33^ ^]^ while recent studies show that first‐row transition metals (Fe,^[^ ^34^ ^]^ Cu,^[^ ^35^ ^]^ Co,^[^ ^36^, ^37^ ^]^ Ni,^[^ ^38^, ^39^ ^]^ and Mn^[^ ^6^, ^40^, ^41^ ^]^) can also mediate BH reactions, albeit typically requiring higher catalyst loadings and more demanding reaction conditions.^[^ ^6^, ^27^, ^34^, ^42^ ^]^ Consequently, Ru‐ and Ir‐based catalysts remain the most widely adopted for BH processes, striking a favorable balance between efficiency, sustainability, and practical feasibility (Figure 1b).^[^ ^33^, ^43^, ^44^, ^45^ ^]^ Despite their potential, many BH protocols still rely on energy‐intensive conditions,^[^ ^25^, ^46^ ^]^ an inert atmosphere,^[^ ^31^, ^47^ ^]^ long reaction times,^[^ ^48^ ^]^ and hydrocarbon‐based solvents,^[^ ^49^ ^]^ which limit their practicality and sustainability. As the industries shift toward greener standards, there is increasing demand for BH strategies that operate under safer, milder, and more efficient conditions.

To fulfil these requirements, mechanochemistry represents a groundbreaking, solvent‐free, and energy‐efficient synthetic approach rapidly gaining prominence as a transformative platform for achieving chemical reaction processes.^[^ ^50^, ^51^, ^52^, ^53^, ^54^, ^55^, ^56^, ^57^ ^]^ In this domain, mechanocatalysis is a specialized aspect of mechanochemistry where mechanical force drives catalytic transformations.^[^ ^58^, ^59^, ^60^, ^61^, ^62^, ^63^, ^64^, ^65^ ^]^ This approach has facilitated a broad range of emblematic transformations, such as Suzuki–Miyaura^[^ ^66^, ^67^ ^]^ and Negishi cross‐couplings,^[^ ^68^ ^]^ Mizoroki–Heck reactions,^[^ ^69^ ^]^ olefin metathesis,^[^ ^70^ ^]^ and Buchwald–Hartwig aminations^[^ ^71^ ^]^ often exceeding the limitations of conventional solution‐based methods. Under mechanochemical conditions, mechanical force directly activates unconventional modes of energy transfer, significantly influencing reaction kinetics, pathways, and interfacial phenomena, often in ways that differ significantly from those in solution‐based processes.^[^ ^50^, ^72^ ^]^

Here, we present the first demonstration of BH reactions under ball milling conditions, enabling the solvent‐free synthesis of *N*‐alkylated amines and *N*‐heterocycles at ambient temperature with minimal catalyst loadings, using a commercially available ruthenium‐based catalyst (Figure 1c).^[^ ^73^, ^74^, ^75^, ^76^ ^]^

## Results and Discussion

### Mechanochemical *N*‐Alkylation of Primary Aromatic Amines Using the BH Strategy

To achieve an efficient mechanochemical BH reaction, we first examined a selection of commercially available transition metal catalysts under mechanochemical conditions (Tables 1b and S1), focusing primarily on Ru‐based pincer catalysts that have shown prior applications in *transfer hydrogenation* and *acceptor‐less dehydrogenation processes*.^[^ ^76^, ^77^ ^]^ Our initial catalyst screening for the room‐temperature mechanochemical BH reaction was conducted in a 10 mL zirconia jar equipped with one 10 mm zirconia ball (2.87 g). The reaction mixture comprised *p*‐anisidine (**1a**, 1.0 mmol) and pentanol (**2a**, 1.0 mmol), along with potassium *tert*‐butoxide (KO*t*Bu, 2.0 mmol). Milling was carried out for 4 h at 30 Hz using a Retsch MM‐500 Vario mill. Among the tested catalysts, ruthenium complexes bearing phosphine ligand frameworks exhibited the most effective performance. In particular, Ru‐MACHO (**[Ru]‐1**) delivered the highest catalytic efficiency (Table 1a, entry 1), whereas Ru‐MACHO‐BH (**[Ru]‐2**) showed reduced activity under mechanochemical BH conditions (Table 1a, entry 2). Notably, *cis*‐[RuCl_2_(dppf)(ampy)] (**[Ru]‐3**), previously reported to drive BH reactions at room temperature in solution, achieved good conversion under ball milling (Table 1a, entry 3). Conversely, the Milstein catalyst (**[Ru]‐4**) and RuCl_2_[(*R*)‐xylbinap][(*R*)‐daipen (**[Ru]‐6**) catalyst, despite their proven effectiveness in dehydrocoupling reactions of alcohols and amines,^[^ ^78^ ^]^ displayed limited reactivity in the mechanochemical setting (Table 1a, entries 4,5). Other catalysts yielded only poor to moderate conversions under analogous conditions. In contrast, in the case of first‐row transition metal catalysts, the reaction failed to provide any products (see Tables S1 and 1b). We subsequently expanded our approach by reducing the catalyst loading of Ru‐MACHO from 2% to 0.05%. Under this reaction condition, the full conversion requires an increase in reaction time from 4 to 6 h (Table 1a, entries 6, 7).

**Table 1:** a) Optimization of reaction conditions for aromatic amines. b) Catalyst screening for product **3aa** (see SI for the complete list of catalyst screening).

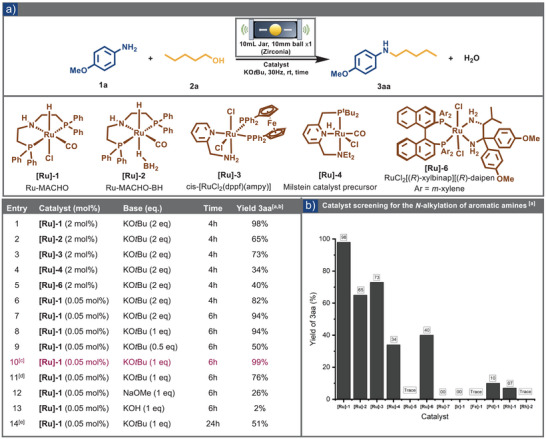

^a)^ Reaction conditions: *p*‐anisidine (1.0 mmol,1 eq.), pentanol (1.0 mmol, 1 eq.), potassium *tert*‐butoxide (KO*t*Bu), and catalyst were placed in a 10 mL zirconia jar with a 10 mm zirconia ball and milled at 30 Hz in MM‐500 Vario for the given time at 25 °C.

^b)^ All the yield determined by GC‐MS.

^c)^ Reaction performed in 4.0 mmol scale conditions: *p*‐anisidine (4.0 mmol, 1 eq.), pentanol (4.0 mmol, 1 eq.).

^d)^ Reaction performed at 15 Hz instead of 30 Hz.

^e)^ Reaction performed in toluene (1 M) at standard solution phase conditions.

We subsequently investigated how the base‐to‐alcohol ratio influences the reaction outcome. Reducing the potassium *tert*‐butoxide (KO*t*Bu) loading from 2 equivalents to 1 equivalent had no impact on yield (Table 1a, entry 8). However, additional reductions significantly diminished productivity: lowering the base to 0.5 equivalents led to a yield of 50% (Table 1a, entry 9). These results align with observations in solution‐based systems, where the base concentration significantly influences the formation rate of the alkoxide intermediate and the reaction kinetics.^[^ ^79^ ^]^ We extended our methodology by performing the reaction on a larger scale under standard milling conditions, resulting in excellent product yield (>99%, Table 1a, entry 10). Under these optimized conditions, a turnover number (TON) exceeding 2000 and a turnover frequency (TOF) of 9.36 × 10⁻^2^ s⁻¹ were attained, highlighting the catalyst's remarkable efficiency.^[^ ^80^ ^]^ Additionally, at the reduced milling frequency of 15 Hz, product formation was observed, though with a lower yield. The choice of the base also proved crucial for the mechanochemical transformation. Replacing potassium *tert*‐butoxide with sodium methoxide (NaOMe) resulted in a significant reduction in product yield (26%) and the generation of a 5% *N*‐methylated byproduct (Table 1a, entry 12). Weaker bases, such as potassium hydroxide (Table 1a, entry 13) and potassium carbonate, failed to drive the reaction to any extent (see Table S2 for a complete list). Ultimately, a series of control experiments in toluene confirmed the pivotal role of our mechanochemical approach in achieving a highly efficient and rapid BH reaction. Under solvent‐based conditions, the yield dropped to 16% at the 6‐h mark (Table S2, entry 16) and only 51% after 24 h of mixing (Table 1a, entry 14). Even at a slightly elevated temperature of 40 °C for more than 12 h, the yield only marginally increased to 58% (Table S2, entry 19). Moreover, a neat control experiment conducted under conventional conditions suffered from low reproducibility and sluggish kinetics (see Table S2 for the complete list). These results highlight the unique benefits of our mechanochemical methodology compared to traditional solvent‐based protocols, emphasizing that this methodology does not divert the reaction through an alternative pathway; rather, it enhances the kinetics resulting in a system that surpasses solution‐phase processes in terms of efficiency and reproducibility.

**Scheme 1:**
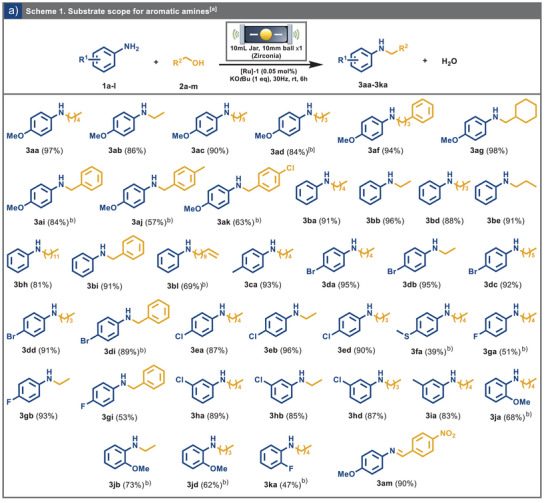
a) ^a)^ Reaction scope for aromatic amines. Reaction conditions: *p*‐anisidine (4.0 mmol,1 eq.), pentanol (4.0 mmol, 1 eq.), potassium *tert*‐butoxide (KO*t*Bu, 1 eq.), and Ru‐MACHO catalyst (**[Ru]‐1**, 0.05 mol%) were placed in a 10 mL zirconia jar with a 10 mm zirconia ball and milled at 30 Hz in Retsch MM‐500 Vario for 6 h at 25 °C; Yields of the products were calculated after extraction with 20 mL of ethyl acetate (EtOAc) and concentrating under reduced pressure. ^b)^ Yield calculated after column chromatography.

With the optimal conditions established, we examined the versatility of our catalytic system by broadening the reaction scope to various aromatic amines and alcohols. The alkylation of *p*‐anisidine **1a** with a range of alcohols proceeded efficiently, yielding the desired products with excellent selectivity (Scheme 1a). For instance, the reaction of *p*‐anisidine with ethanol resulted in the alkylation product **3ab** in a high yield. Increasing the alkyl chain length did not hinder reactivity, furnishing the desired product in excellent yields (Scheme 1a, entries **3ac** and **3ad**). Furthermore, *p*‐anisidine reacted effectively with 3‐phenylpropan‐1‐ol and cyclohexylmethanol, yielding the corresponding alkylated products **3af** and **3ag** in excellent yields. When using *para*‐substituted benzyl alcohols, satisfactory yields were achieved, and reactivity was consistent regardless of electron‐donating or electron‐withdrawing substituents. Specifically, product **3aj**, featuring a *p*‐methyl substituent on the benzyl alcohol, and product **3ak**, with a *p*‐chloro substituent, were obtained in respectable yields of 57% and 63%, respectively. Aniline reacted efficiently with a range of alcohols, spanning short to long alkyl chains, to produce alkylated products **3ba**–**3bh** in high yields (see Scheme 1a). Furthermore, the reaction of aniline with benzyl alcohol proceeded seamlessly, delivering product **3bi** in excellent yield alongside a minor imine byproduct. The reaction conditions also demonstrated remarkable selectivity, as no reduction of C═C double bond was observed in the case of alcohol **2l**, allowing for the isolation of product **3bl** with a yield of 69%. Similarly, *p*‐toluidine reacted efficiently with pentanol to yield product **3ca**. The electron‐deficient *p*‐bromoaniline **1d** exhibited consistent reactivity with various alcohols, resulting in products **3da–3dd** in excellent yields. Moreover, *p*‐bromoaniline reacted smoothly with benzyl alcohol, producing product **3di** in excellent yield alongside a minor imine byproduct. Aniline with a *p*‐chloro substituent was well‐tolerated, yielding products **3ea**–**3ed** with high selectivity and excellent yields. Interestingly, electron‐deficient anilines demonstrated superior reactivity to their electron‐rich counterparts, underscoring the influence of electronic effects on reaction efficiency. Notably, 4‐(methylthio)‐*N*‐pentylaniline **1f** produced the alkylated product with pentanol, **3fa**, in a satisfactory yield of 39%, despite the potential for catalyst deactivation by the sulfur‐containing group. However, *p*‐fluoro aniline provided products **3ga** and **3gi** in moderate yields of 51% and 53%, respectively. Anilines bearing substituents at various positions, such as *m*‐chloro and *m*‐methyl groups, did not significantly affect the reaction yields, with products **3ha‐3hb, 3hd,** and **3ia** being formed efficiently. In contrast, *o*‐methoxy substituents introduced a degree of steric hindrance, resulting in slightly reduced yields for products **3ja**,**3jb,** and **3jd**. Interestingly, this steric effect was less pronounced in the case of **3ka**, where the yield remained comparable to that of the *para*‐substituted counterpart. Notably, while the reaction conditions selectively avoided nitro group reduction, reactions involving *p*‐anisidine and 4‐nitrobenzyl alcohol stalled at the imine intermediate **3am**. This observation suggests the catalytic system could not complete the reduction step under these circumstances.

### Mechanochemical *N*‐Alkylation of Primary Aliphatic Amines Using BH Strategy and Synthesis of *N*‐heterocycles

**Scheme 2:**
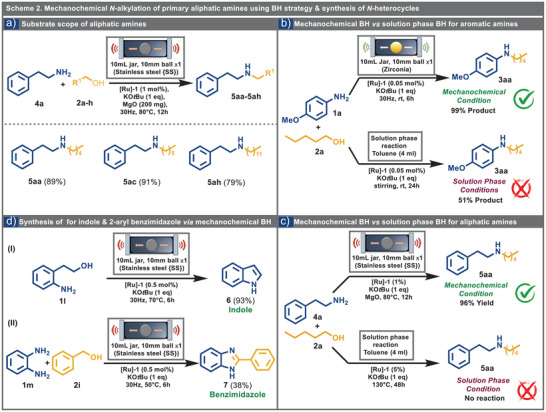
a) Reaction conditions: 2‐phenylethylamine (1.0 mmol,1 eq.), pentanol (2.0 mmol, 2 eq.), potassium *tert‐*butoxide (KO*t*Bu, 1 eq.), MgO (200 mg), and Ru‐MACHO ([Ru]‐1, 1 mol%) were placed in a 10 mL SS jar with a 10 mm SS ball and milled at 30 Hz in Fritsch P‐23 for 12 h at 80 °C; Yield calculated after column chromatography. b,c) Comparison between mechanochemical BH reaction with solution phase BH reaction; (b: with aromatic amine; c: with aliphatic amine). d) Reaction conditions: (I) 2‐aminophenethyl alcohol (1.0 mmol, 1 eq.), KO*t*Bu (1 eq.), and [Ru]‐1 (0.5 mol%) were placed in a 10 mL SS jar with a 10 mm SS ball and milled at 30 Hz in Fritsch P‐23 for 6 h at 70 °C; (II) *o*‐phenylenediamine (1.0 mmol, 1eq.), benzyl alcohol (1.0 mmol, 1 eq.) KO*t*Bu (1 eq.), and [Ru]‐1 (0.5 mol%) were placed in a 10 mL SS jar with a 10 mm SS ball and milled at 30 Hz in Fritsch P‐23 for 6 h at 50 °C.

Following the successful results of *N*‐alkylation of primary aromatic amines using the mechanochemical BH strategy, we aimed to broaden the substrate scope to include aliphatic amines. Unfortunately, the optimized conditions for aromatic amines proved ineffective for aliphatic substrates. We hypothesized that adjusting the reaction parameters might enhance the catalytic system's versatility and, therefore, undertook systematic screening. Initially, we applied the optimized conditions for aromatic amines to phenylethylamine (**4a**, 1.0 mmol) in the presence of pentanol (**2a**, 2.0 mmol), using **[Ru]−1** (0.05 mol%) and KO*t*Bu (1.0 mmol). Under these conditions, however, the desired alkylation product did not form (see Table 2a, entry 1). To achieve product formation, several attempts were made to increase the catalyst and base loadings, as well as the time (see Table 2a, entries 2–4); however, these efforts proved unproductive. Additionally, performing the reaction under a nitrogen atmosphere did not result in significant product formation (Table 2a, entry 5). Extending the reaction time or altering the milling material (Table 2a, entry 6) also yielded no detectable products. To tackle this challenge, we applied external heating to 80 °C in a Fritsch P‐23 mill using a heat gun on a 10 mL stainless‐steel (SS) jar containing a 10 mm SS milling ball (1 x 10 mm = 3.6 g), **[Ru]‐1** catalyst (1.0 mol%), and potassium *tert*‐butoxide, while monitoring the temperature externally with an IR‐gun apparatus.^[^ ^81^ ^]^ Under these conditions, the alkylated product **5aa** was obtained with a 28% NMR yield after 6 h of ball milling (Table 2a, entry 7). Extending the reaction time to 12 h, we achieved a nearly quantitative conversion of **5aa** (Table 2a, entry 8). Reducing the catalyst loading significantly decreased the yield (Table 2a, entry 9), while halving the base loading led to a modest decline, which was offset by adding MgO (200 mg mmol^−1^ of amine), bringing the yield back to 96% (Table 2a, entry 11). We conducted analogous reactions in solution to validate the advantages of mechanochemical conditions. Even when phenylethylamine in toluene was heated to 130 °C for 48 h, no formation of **5aa** was detected (Table 2a, entry 12). This stark contrast emphasizes the crucial role of mechanical action under solvent‐free conditions. The mechanical forces generated in a setup likely promote bond‐breaking and forming more efficiently than traditional solution‐phase reactions, where diffusion and solubility limitations often restrict reaction rates. Mechanochemistry intensifies molecular interactions by increasing their collision frequency and energy, thereby accelerating bond activation and hydrogen transfer during BH processes. This enhanced contact overcomes the diffusion and solubility limitations seen in solution‐based systems, significantly boosting hydrogenation rates (see Scheme 2b,c). The solvent‐free nature of mechanochemistry enhances reactant concentration and collision efficiency, while mechanical forces facilitate catalyst dispersion and accessibility.

**Table 2:** a) Optimization of reaction conditions for aliphatic amines.

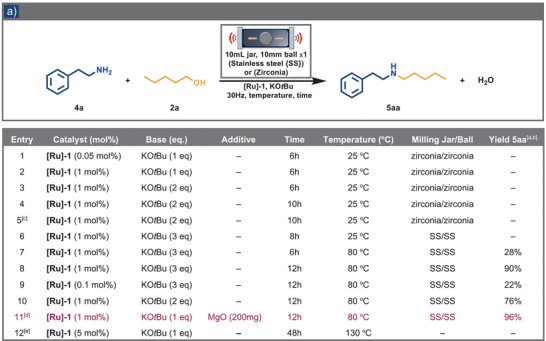

^a)^ Reaction conditions: 2‐phenylethylamine (1.0 mmol,1 eq.), pentanol (2.0 mmol, 2 eq.), potassium *tert*‐butoxide (KO*t*Bu), and catalyst **[Ru]‐1** were placed in a 10 mL jar with a 10 mm ball (Stainless steel {SS} or Zirconia) and milled at 30 Hz in Fritsch P‐23/MM‐500 Vario for the given time and temperature.

^b)^ All the yield determined by ^1^H NMR.

^c)^ Reaction conducted under N_2_.

^d)^ Milling additive (200 mg mmol^−1^ of amine).

^e)^ Reaction performed standard solution condition in toluene (2 M) with 5 mol% **[Ru]‐1** at 130 °C for 48 h.

Additionally, the localized heat generated during milling supplies the activation energy necessary for the reaction. Together, these factors highlight the unique advantages of mechanochemistry in overcoming the limitations of traditional solution‐phase methods, enabling more efficient hydrogen transfer for these transformations.

Applying this methodology under similar conditions yielded compounds **5ac** and **5ah** from hexanol and dodecanol, respectively, with excellent yields (Scheme 2a). The construction of *N*‐heterocyclic scaffolds is vital for pharmaceutical research and drug development due to their diverse pharmacological effects and adaptable structural properties. Thus far, the preparation of *aza*‐heterocycles has frequently depended on multi‐step reactions,^[^ ^7^, ^82^ ^]^ making this BH strategy particularly significant and appealing. We applied our mechanochemical BH methodology to synthesize indole **6** and 2‐phenyl benzimidazole **7** (Scheme 2d). Initial experiments were conducted by using 2‐aminophenethyl alcohol **1l** with the standard condition (see Scheme 2d) in a zirconia jar at room temperature for 3 h. However, the desired products were not observed under these conditions, even though increasing the reaction time to 6 h. We placed the reaction mixture in a 10 mL stainless‐steel (SS) jar with a 10 mm SS milling ball (3.6 g) on a Fritsch P‐23 mill to overcome these conditions. We applied external heating at 70 °C using a heat gun, with the temperature monitored externally via an IR gun apparatus. Under these conditions, the reaction converted completely, yielding indole **6** as the sole product with a yield of 93% (Scheme 2d). Additionally, when *o*‐phenylenediamine **1m** was mixed with benzyl alcohol **2i,** 2‐phenyl benzimidazole **7** was successfully obtained 38% at 50 °C (Scheme 2d).

### Mechanistic Investigations

Mechanistic studies in mechanochemistry are particularly challenging due to the complex, heterogeneous, and dynamic nature of solid‐state reactions.^[^ ^83^ ^]^ Unlike solution‐based processes, which allow real‐time monitoring of intermediates, mechanochemical transformations require continuous mechanical energy, shear forces, and fluctuating mixing conditions—factors that complicate direct observation.^[^ ^84^ ^]^ Hydrogenation reactions, involving sequential steps such as dehydrogenation, condensation, and re‐hydrogenation through transient intermediates like metal‐hydrides and imines or enones, pose particular challenges under mechanochemical conditions due to the elusive nature of these species. Nonetheless, elucidating these mechanisms is crucial for rational catalyst design, reaction optimization, and the advancement of sustainable, solvent‐free methodologies.

**Scheme 3:**
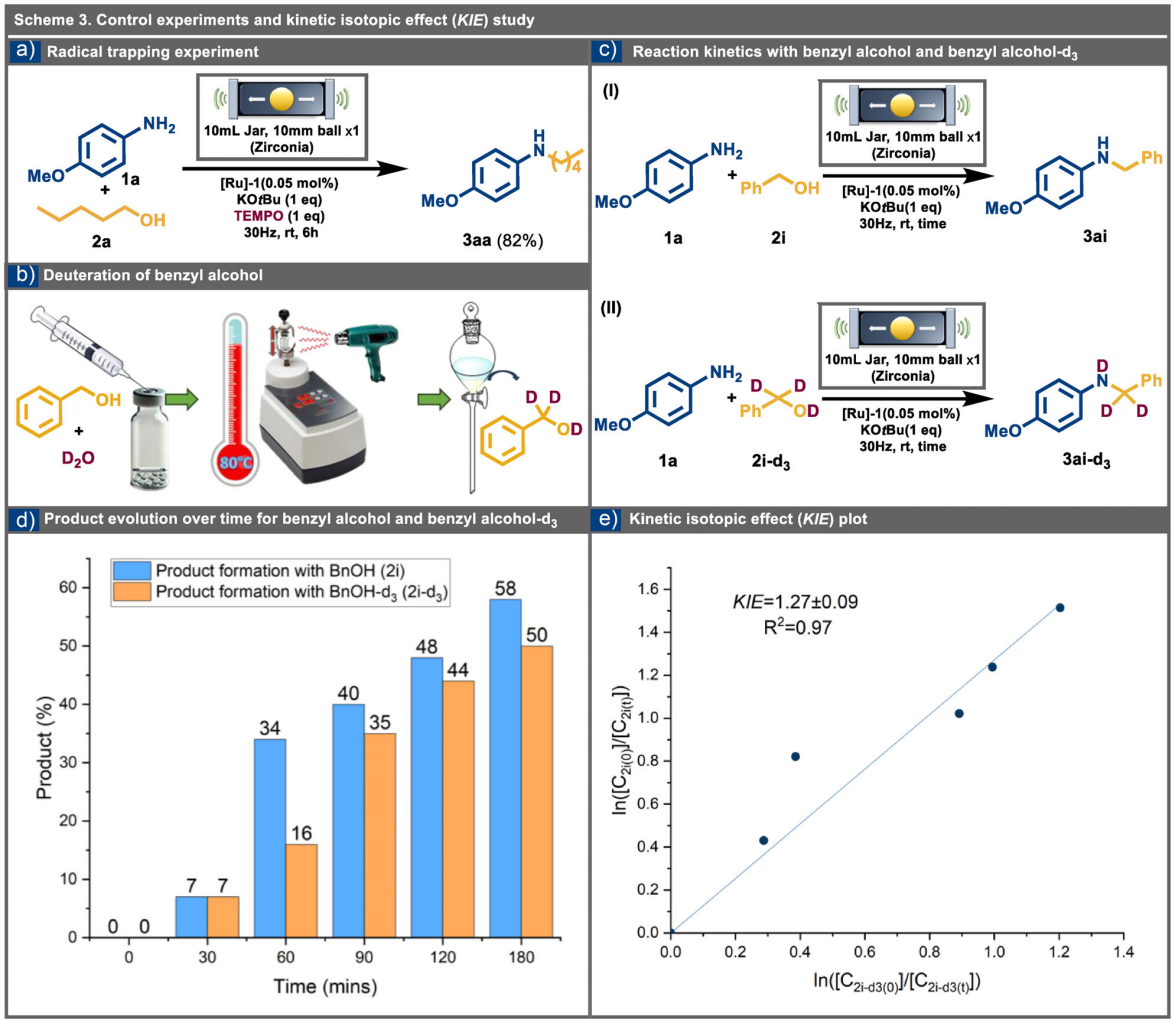
Control experiments for mechanochemical BH reactions. a) Radical trapping experiment using TEMPO. b) A simple schematic diagram for deuteration of benzyl alcohol **2i‐d_3_** using mechanochemistry. c) Deuterium isotope labelling experiment for *N*‐alkylation of *p*‐anisidine **1a** with benzyl alcohol‐d_3_ **2i‐d_3._** d) Product evolution over time for **3ai‐d_3_** and **3ai**. e) Kinetic isotopic effect (*KIE*) analysis with benzyl alcohol **2i** and benzyl alcohol‐d_3_ **2i‐d_3_** with the plot.

To investigate the BH mechanistic pathway, we conducted an initial examination using *p*‐anisidine (**1a**, 4.0 mmol), *n*‐pentanol (**2a**, 4.0 mmol), and KO*t*Bu (4.0 mmol) in the presence of the radical trapping agent TEMPO (4.0 mmol) under standardized optimized reaction conditions. The formation of product **3aa** with an 82% yield, even in the presence of TEMPO, suggests that the reaction does not follow a radical‐mediated pathway (Scheme 3a). Further mechanistic insights were gained through deuterium isotope labeling experiments, leading to a milder and more efficient strategy for synthesizing benzyl alcohol‐d_3_ (**2i‐d_3_**). Utilizing a simple mechanochemical mixing procedure, we employed D₂O as the deuterium source in the presence of Ru‐MACHO as a catalyst (see SI for the complete procedure), a method previously reported for isotopic incorporation in solution. By optimizing the reaction conditions, we significantly reduced the required amount of D₂O from 1 mL to just 300 µL per mmol,^[^ ^85^ ^]^ enhancing both efficiency and sustainability. This refined approach enabled the effective conversion of benzyl alcohol **2i** to benzyl alcohol‐d_3_ (**2i‐d_3_**). Notably, the final product (**2i‐d_3_**) exhibited approximately 99% di‐deuteration at the benzyl position, with only trace amounts of the non‐deuterated product **2i** detected. This achieves high isotopic enrichment while minimizing excess reagent consumption monitored by NMR spectroscopy and high‐resolution mass spectrometry (HRMS) (Scheme 3b). The synthesized benzyl alcohol‐d₃ (**2i‐d_3_**) was subsequently utilized in an *N*‐alkylation reaction with *p*‐anisidine, yielding an *N*‐alkylated amine with precise deuterium labelling. The reaction's progress was monitored over time using quantitative NMR analysis (Scheme 3c). For subsequent mechanistic insights, particularly into the rate‐determining step (rds) and the electronic effects of aniline substituents, we conducted a kinetic isotope effect (*KIE*) analysis and performed a Hammett analysis.

### Kinetic Isotopic Effect Investigations (*KIE*)

To gain deeper mechanistic insights into mechanochemical BH processes, *KIE* studies were carried out using deuterated benzyl alcohol, synthesized via an optimized BH‐based strategy (see SI for details). These experiments aimed to assess the impact of deuterium substitution on the reaction rate.^[^ ^86^ ^]^ Quantitative ¹H NMR spectroscopy, with ethylene carbonate as an internal standard, was employed to monitor reactions between benzyl alcohol **2i** or its deuterated analogue (**2i‐d₃**) and *p*‐anisidine **1a**, tracking alcohol consumption over time (Scheme 3c,d). The rate of decay in concentration for both benzyl alcohol **2i** and benzyl alcohol‐d_3_ (**2i‐d_3_**) exhibited similar trends; however, the reaction rate was faster for benzyl alcohol **2i**. The calculated low *KIE* value of 1.27 ± 0.09 (see Scheme 3e) with the ***R* ^2^** value of 0.97 suggests that the rds is not the alcohol oxidation step (see SI for complete data). During the reduction of the in situ formed imine, the reducing agent [**Ru1‐H_2_**] cleaves the Ru─H bond. The lower force constant of the Ru─H bond compared to that of C─H bonds results in a *KIE*, albeit a smaller one. The low *KIE* observed rule out not only the alcohol oxidation but also the imine reduction as the rds.^[^ ^87^ ^]^ From the substrate scope, the isolation of compound **3am** as an imine, without further reduction to the corresponding amine, suggests that imine formation is not the rds. This conclusion is further supported by ^1^H NMR spectroscopy, which revealed no peaks for aldehydes, confirming the absence of intermediate oxidation products. In summary, the low *KIE* value, along with these observations, strongly suggests that the rate‐determining step likely involves the coordination of the [**Ru1‐H_2_**] species with the imine intermediate.^[^ ^87^ ^]^

### Hammett Analysis with *para*‐Substituted Anilines

**Figure 2:**
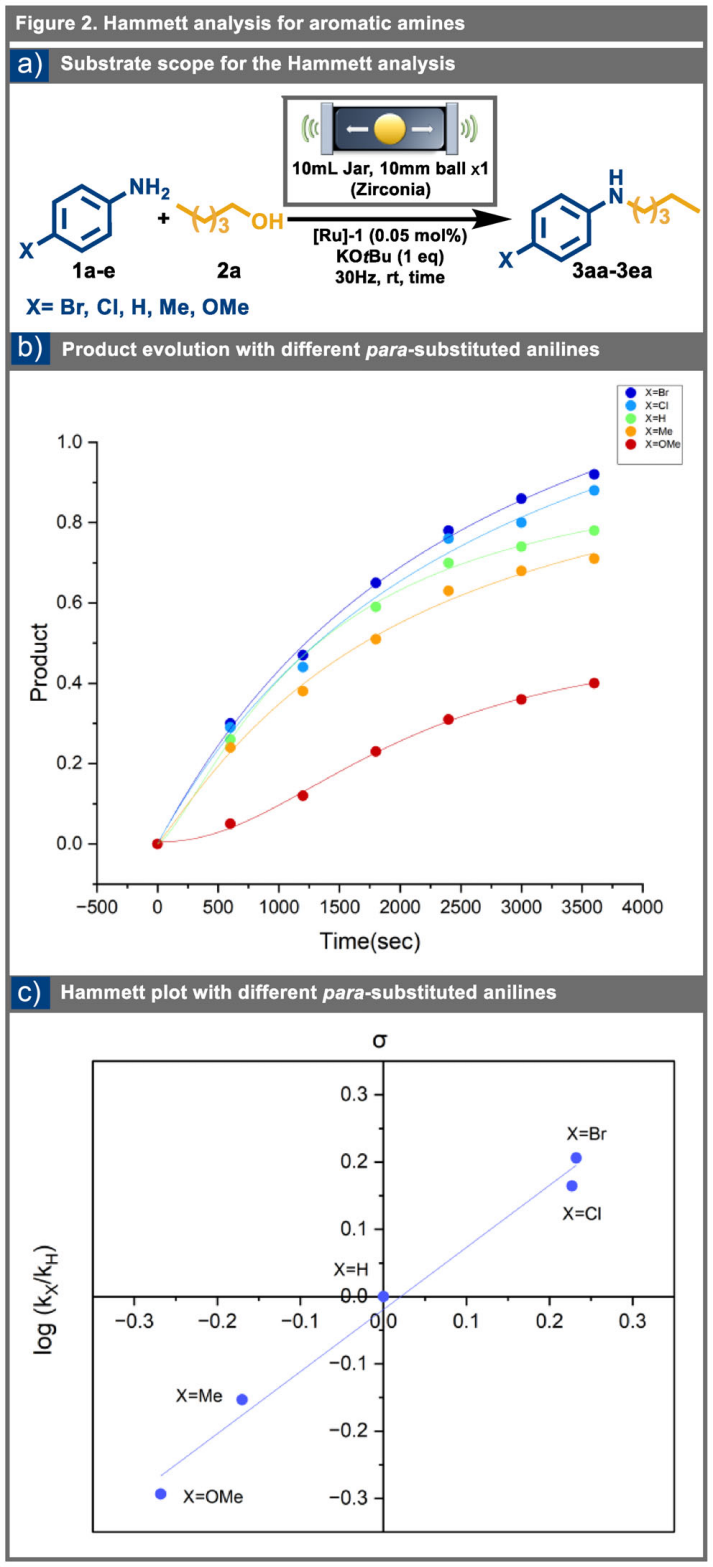
a) Substrate scope for the Hammett analysis; b) Product evolution with different *para*‐substituted anilines (**1a‐e**) and pentanol (**2a**); c) Hammett plot for *para*‐substituted anilines (slope = 0.92 ± 0.05, ***R* ^2^** = 0.98).

We conducted noncompetition Hammett experiments with a series of *para*‐substituted anilines that contain electron‐donating and electron‐withdrawing groups to further investigate the kinetics of the mechanochemical reaction process.^[^ ^88^, ^89^, ^90^, ^91^ ^]^ The reactions between pentanol **2a** and *para*‐substituted aniline derivatives **1a**‐**e** were closely monitored using quantitative ^1^H NMR spectroscopy, with ethylene carbonate as the internal standard for accurately measuring aniline concentrations over time (see Figure 2b). The results demonstrated a clear trend in reactivity based on the electronic nature of the substituents. Anilines with electron‐withdrawing groups in the *para* position (e.g., **1d‐e**) exhibited significantly higher reactivity than their electron‐donating counterparts (e.g., **1a**, **1c**) (see Figure 2a). *p*‐Bromoaniline showed the highest activity among the tested derivatives, while *p*‐anisidine exhibited the least reactivity. The Hammett analysis provided quantitative insights into the electronic effects of substituents on the reaction mechanism, correlating reaction kinetics or equilibria with substituent constants (**σ**).^[^ ^87^ ^]^ This experimental framework facilitated the isolation and assessment of the critical factors influencing the rate‐determining step in the hydrogenation pathway (see SI for complete data).

The Hammett plot for the mechanochemical Borrowing Hydrogen reaction was shaped by correlating log (**k*_X_*/k*_H_***) with **σ**. This plot exhibited a strong linear relationship (***R* ^2^** = 0.98) and displayed a reaction constant (**ρ**) of 0.92 ± 0.05 (see Figure 2c). The positive value of **ρ** indicates the development of a partial positive charge in the transition state, suggesting an electron‐deficient environment. These findings, altogether with insights from *KIE* studies and the other experimental evidences, lead to the conclusion that the coordination between the [**Ru1‐H_2_**] species and the imine might be the slowest step in the reaction mechanism. Under mechanochemical BH conditions, the ground‐state energy of **[Ru1‐H_2_]** and the energy barrier associated with the transition state, due to the interaction of **[Ru1‐H_2_]** with the imine, are critical factors to consider.^[^ ^87^ ^]^ This finding highlights the importance of electronic factors, as the binding affinity of imines with electron‐rich anilines shows sluggish reactive performance compared to their electron‐withdrawing counterparts.

**Scheme 4:**
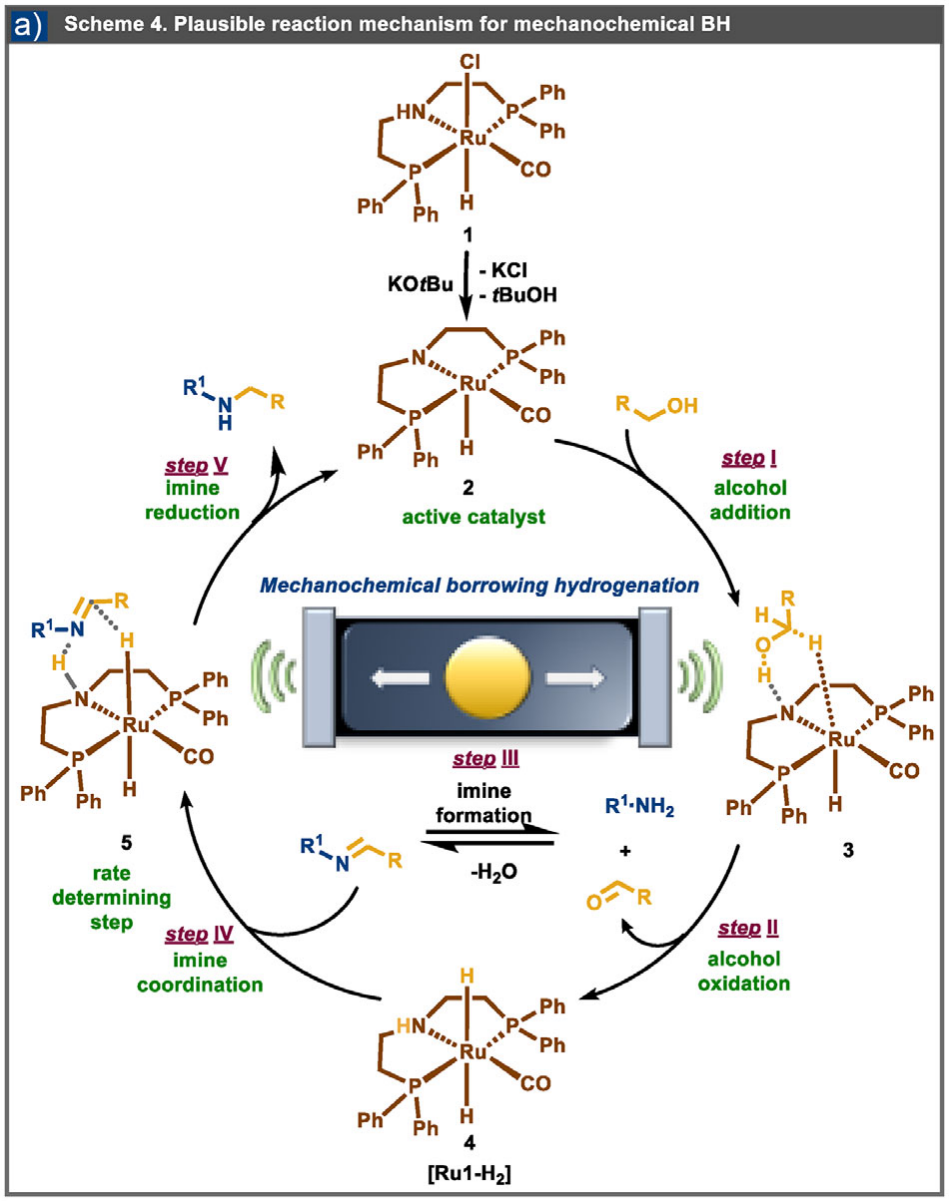
a) Plausible reaction mechanism for mechanochemical BH.

Based on the comprehensive experimental results outlined above, we propose a plausible reaction mechanism for the observed outcomes (Scheme 4a). Initially, in the presence of KO*t*Bu, the Ru‐MACHO complex (Scheme 4a, species 1) is activated through mechanical milling, forming the active catalyst (Scheme 4a, species 2). In the catalytic cycle's first step (step I), alcohol coordination occurs with the activated catalyst. This coordination facilitates the oxidation of the alcohol, leading to the formation of the corresponding aldehyde via a ruthenium−hydride complex **[Ru1‐H_2_]** (Scheme 4a, species 4). In a parallel reaction pathway (step III), the aldehyde produced in step II reacts with an amine species, forming the corresponding imine and the byproduct water. This reaction is succeeded by coordinating the imine species to the ruthenium−hydride complex [**Ru1‐H_2_**] (Scheme 4a, species 5), which prepares for the imine reduction.^[^ ^87^ ^]^ *KIE* studies, combined with Hammett analysis, suggest that the coordination of the imine to the catalyst is the rate‐determining step (rds) of the catalytic cycle. Following the effectively irreversible formation of **complex 5**, imine reduction occurs, forming the secondary amine product. Notably, the secondary amine product does not inhibit the reaction, as it does not form strong interactions with the ruthenium center at any point during the catalytic cycle.

### Green Metrics Calculations

We assessed the green chemistry metrics by comparing our methodology to a previously reported, solution‐based approach employing a Ru‐based catalyst at room temperature.^[^ ^44^ ^]^ We specifically conducted quantitative and qualitative evaluations by measuring the E‐factor^[^ ^56^, ^92^ ^]^ and eco‐scale values.^[^ ^93^ ^]^ Our results show that the mechanochemical procedure outperforms analogues in solution, achieving an E‐factor of 30 compared to 533 and an eco‐scale value of 70 versus 52.5 (for details, see SI). Notably, the high E‐factor in the solution‐based method largely stems from the need for chromatographic purification.

## Conclusion

This work presents a mechanochemical strategy for BH to synthesize *N*‐alkylated amines and heterocycles in a direct, solvent‐free, and efficient condition. By harnessing ball milling alongside a ruthenium‐based catalyst, our approach effectively addresses the challenges associated with solution‐phase syntheses, including the use of toxic reagents and the need for high temperatures, while achieving nearly quantitative conversion of a variety of primary amines and alcohol substrates. *KIE* studies and Hammett analyses underscore the crucial role of substrate‐derived hydrogen in the reaction, distinguishing it from conventional methods. Furthermore, this methodology allows the synthesis of various heterocycles, including indole and benzimidazole, showcasing its versatility. Mechanochemical ball milling has emerged as a more sustainable alternative to traditional methods, making it well‐suited for pharmaceutical, agrochemical, and fine chemical applications. Operating under mild, solvent‐free conditions, it aligns with current sustainability goals. Ongoing research will continue to deepen our understanding and expand the applications of this innovative approach in synthetic chemistry.

## Supporting Information

The authors have cited additional references within the Supporting Information.

## Conflict of Interests

The authors declare no conflict of interest.

## Supporting information



Supporting Information

## Data Availability

The data that support the findings of this study are available in the supplementary material of this article.
