# Supplementary material for: Mechanochemistry‐Driven Borrowing Hydrogen Processes for Ru‐Catalyzed N‐Alkylation: A Pathway to Enhanced Sustainability and Efficiency
Source: Angew Chem Int Ed Engl. 2025 Jun 23;64(32):e202508050. doi: 10.1002/anie.202508050 (PMC12322645; doi:10.1002/anie.202508050)
Supplement: Supplementary file 1 — Supporting Information [file ANIE-64-e202508050-s001.pdf]

Supporting Information

Mechanochemistry-Driven Borrowing Hydrogen Processes for Ru-Catalyzed *N*-Alkylation: A Pathway to Enhanced Sustainability and Efficiency

Sourav Behera,[a] Dipak J. Fartade,[a] Rita Mocci,*[a] Michela Matta,[a] Lidia De Luca,[b] and Andrea Porcheddu*[a]

[a] S. Behera, Dr. Dipak J. Fartade, Dr. Rita Mocci, Michela Matta, Prof. Andrea Porcheddu
Dipartimento di Scienze Chimiche e Geologiche
Università degli Studi di Cagliari
Cittadella Universitaria, 09042, Cagliari, Italy
E-mail: rita.mocci@unica.it; porcheddu@unica.it

[b] Prof. L. De Luca
Dipartimento di Scienze Chimiche, Fisiche, Matematiche e Naturali
Università degli Studi di Sassari
via Vienna 2, 07100, Sassari, Italy

Table of contents

General Information....3

General Reaction Conditions....4

General experimental procedure for the *N*-alkylation of aromatic amines **3aa-3am**....4

General experimental procedure for the *N*-alkylation of aliphatic amines 5aa-5ah....4

General experimental procedure for the synthesis of heterocycles....5

Reaction Optimization....6

Catalyst screening for mechanochemical borrowing hydrogenation....6

Table S1. Screening of catalysts for the *N*-alkylation of aromatic amine **1a**.[a]....7

Table S2. Optimization of the reaction conditions for the *N*-alkylation of aromatic amine **1a**....8

Control reaction analysis....10

General experimental procedure for radical trapping experiment....10

Kinetic isotopic effect analysis....11

General experimental procedure for deuteration of alcohol....11

General set-up for kinetic isotopic effect analysis....12

Table S3. KIE calculation....13

NMR spectra for KIE....15

Hammett analysis....20

General set-up for Hammett analysis....20

Table S4. Reaction rate coefficient calculation for para substituted anilines (**1a-e**) with pentanol (**2a**)....21

Table S5. Hammett analysis....25

Green Chemistry Metrics Calculations....26

Calculation of the Green Chemistry Metrics for the Mechanochemical Preparation of amine **3aa**....26

Environmental Factor (**E**) for the mechanochemical preparation of amine **3aa**....26

Environmental Factor (**E**) for the preparation of amine **3aa** based on solution synthesis[2]....26

The Eco-scale Score for the Mechanochemical Preparation of Amine **3aa**....27

Table S6. Calculation of Ecoscale score for the Mechanochemical Preparation of Amine **3aa** [a]....27

The Eco-scale Score for the of Amine **3aa** based on solution....27

Spectral data for products....28

Spectral data for aromatic amines **3aa-3am**....28

Spectral data for aliphatic amines....39

Spectral data for *N*-heterocyclic products **6-7**....40

NMR Spectra....41

REFERENCES....84

General Information

Commercially available reagents and solvents were purchased from Acros, Alfa-Aesar, Carlo Erba, Merck and TCI-Europe, and used as received without further purifications. All reactions were monitored by thin-layer chromatography (TLC) performed on glass-backed silica gel 60 F254, 0.2 mm plates (Merck) with suitable eluent, and compounds were visualized under UV light (254 nm) or using iodine vapor chamber. The eluents were technical grade. Mechanochemical reactions were conducted using a Retsch MM500 Vario (room temperature reaction) and Fritsch Pulverisette 23 (heating reaction) apparatus. The reagents were milled using a zirconia grinding jar (10 mL) or stainless-steel jar (10 ml) with one ball (ф = 10 mm) of the same material if otherwise not stated in the parameters. ^{1}H and ^{13}C NMR spectra were recorded on a Bruker Avance III HD 600 MHz NMR spectrometer at 298K unless otherwise specified. Chemical shifts were reported in parts per million (ppm, δ value), and the residual solvent peak was used as internal reference: in proton (chloroform δ 7.26; dimethyl sulfoxide δ 2.50) or in carbon (chloroform δ 77.16; dimethyl sulfoxide δ 39.52). Data are reported as follows: chemical shift δ, multiplicity (s = singlet, d = doublet, t = triplet, q = quartet, m = multiplet, br = broad and combination of thereof), coupling constants (J) in hertz (Hz) and integration. Samples were analyzed using an Agilent 5977B MS interfaced to the GC 7890B equipped with a DB-5ms column (J & W), injector temperature at 230 °C, detector temperature at 280 °C, helium carrier gas flow rate of 1 mL/min. The GC oven temperature program was 100 °C initial temperature with a 4 min hold-time and ramping at 15 °C/min to a final temperature of 270 °C with a 7 min hold time. One µL of each sample was injected in split (1:20) mode. After a solvent delay of 3 minutes, mass spectra were acquired in full scan mode using 2.28 scans/s with a 50–500 amu mass range. Retention times of different compounds were determined by injecting pure compounds under identical conditions. High resolution mass spectra (HRMS) were recorded on LTQ Orbitrap Elite (Thermofischer) instrument (ESI). The temperature for reactions was monitored using POWERFIX Profi (IAN 279816) infrared (IR) thermometer, with Pmax: < 1 mW; λ: 650nm; measuring range: -50 to +380°C. All the experiments were carried out in duplicate to ensure the reproducibility of the experimental data. Yields refer to pure, isolated materials.

General Reaction Conditions

General experimental procedure for the *N*-alkylation of aromatic amines **3aa-3am**

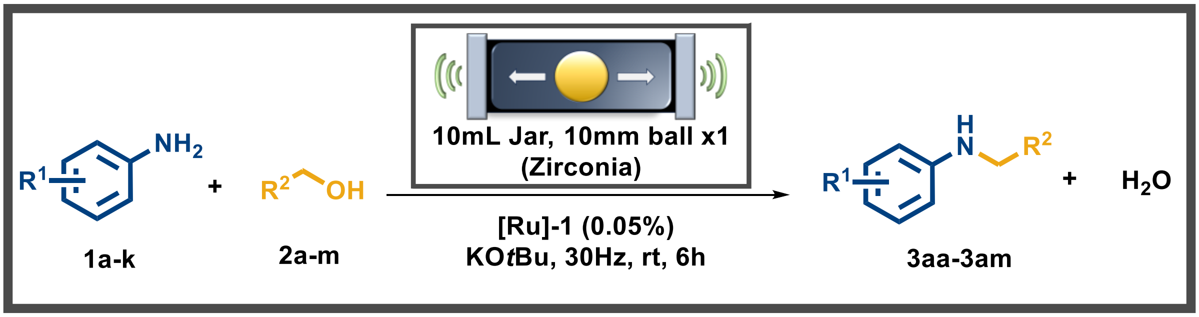

Aromatic amine (**1a-k**, 4 mmol), primary alcohol (**2a-m**, 4 mmol), potassium *tert*-butoxide (KO*t*Bu, 4 mmol) and Ru-MACHO catalyst (0.05 mol%, 1.2 mg) were loaded into a zirconium dioxide grinding jar (10 mL) equipped with 1 ball (ϕ = 10 mm, m 2.87 g) of the same material. The jar was sealed and milled at 30 Hz in a shaker mill MM-500 vario at room temperature, for 6 hours. The reaction was monitored by TLC (hexane/EtOAc: 9/1). After completion of the reaction, the resulting rection mixture was filtered using a sintered vacuum funnel through a small bed of celite (4g) with 20 mL of EtOAc. The filtrate was then evaporated under high vacuum. ^{1}H-NMR and ^{13}C spectrometry confirmed the structure of the compounds. Where necessary, further purification can be achieved by column chromatography (*n*-hexane/EtOAc: 9/1, v/v).

General experimental procedure for the *N*-alkylation of aliphatic amines 5aa-5ah

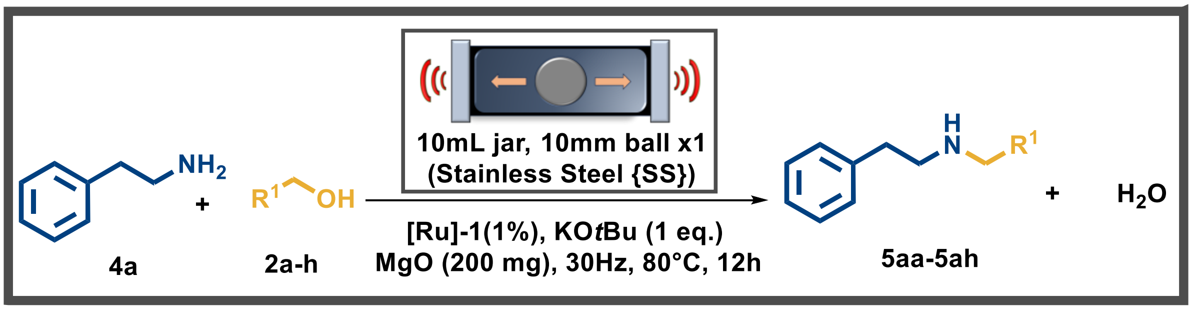

Aliphatic amine (1 mmol), primary alcohol (2 mmol), potassium *tert*-butoxide (KO*t*Bu, 1 mmol), Ru-MACHO catalyst (1 mol%, 6 mg) and MgO (200 mg) were loaded into a stainless-steel grinding jar (10 mL) equipped with 1 ball (ϕ = 10 mm, m 3.6g) of the same material. The jar was sealed and milled at 30 Hz in a shaker mill Fritsch P-23 for 12 hours. The reaction jar was heated to an external temperature of 80°C using a heat gun and maintained as such for the whole course of the reaction.

Then, the reaction jar was cooled down to room temperature, and the reaction progress was monitored by TLC (hexane/EtOAc: 8/2). After completion of the reaction, the resulting rection mixture was filtered using a sintered vacuum funnel through a small bed of celite (1.0 g) with 20 mL of EtOAc and then dried using sodium sulfate and the resultant filtrate was evaporated under high vacuum. The further purification the compound was achieved using column chromatography (*n*-hexane:EtOAc 8:2 v/v). ^{1}H-NMR and ^{13}C spectrometry confirmed the structure of the compounds.

General experimental procedure for the synthesis of heterocycles 6 and 7.

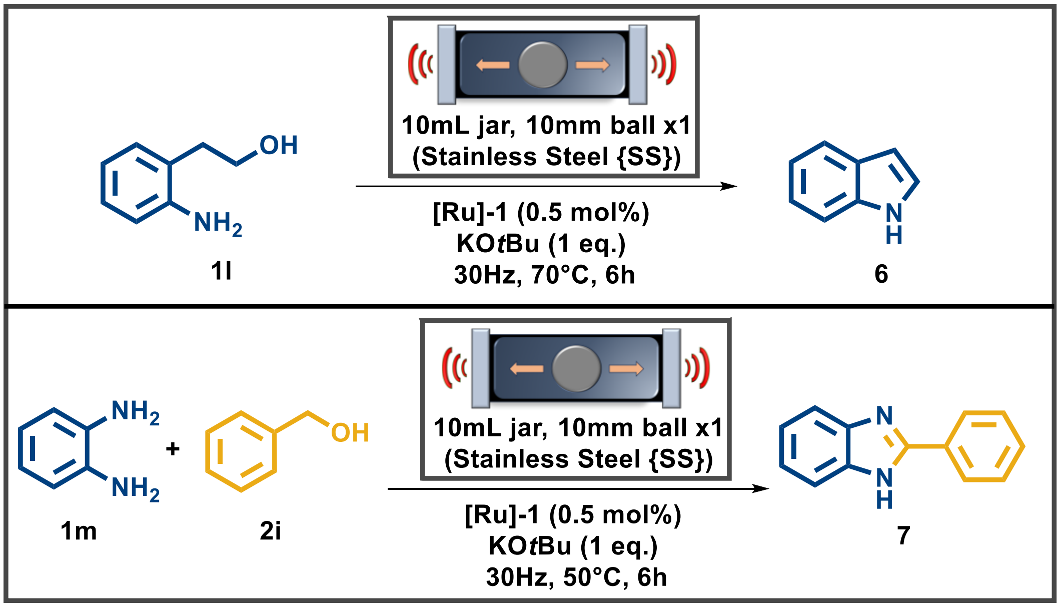

Amine (1 mmol), alcohol (1 mmol), potassium *tert*-butoxide (1 mmol) and Ru-MACHO catalyst (0.5 mol%, 3 mg) were loaded into a stainless-steel grinding jar (10 mL) equipped with 1 ball (ϕ = 10 mm, m 3.6g) of the same material. The jar was sealed and milled at 30 Hz in a shaker mill Fritsch P-23 for 6 hours at an appropriate temperature using a heat-gun. Then, the reaction jar was cooled down to room temperature, and the reaction progress is monitored by TLC (hexane/EtOAc: 7/3). After completion of the reaction, the resulting rection mixture was filtered using a sintered vacuum funnel through a small bed of celite with 20 mL of EtOAc. The filtrate was then evaporated under high vacuum. ^{1}H-NMR and ^{13}C spectrometry confirmed the structure of the compound.

Note: In the case of indole **6**, the product was obtained without any further purification, while in the case of 2-phenyl benzimidazole **7**, the pure product is isolated by column chromatography (*n*-hexane/EtOAc: 7:3 v/v).

Reaction Optimization

Catalyst screening for mechanochemical borrowing hydrogenation.

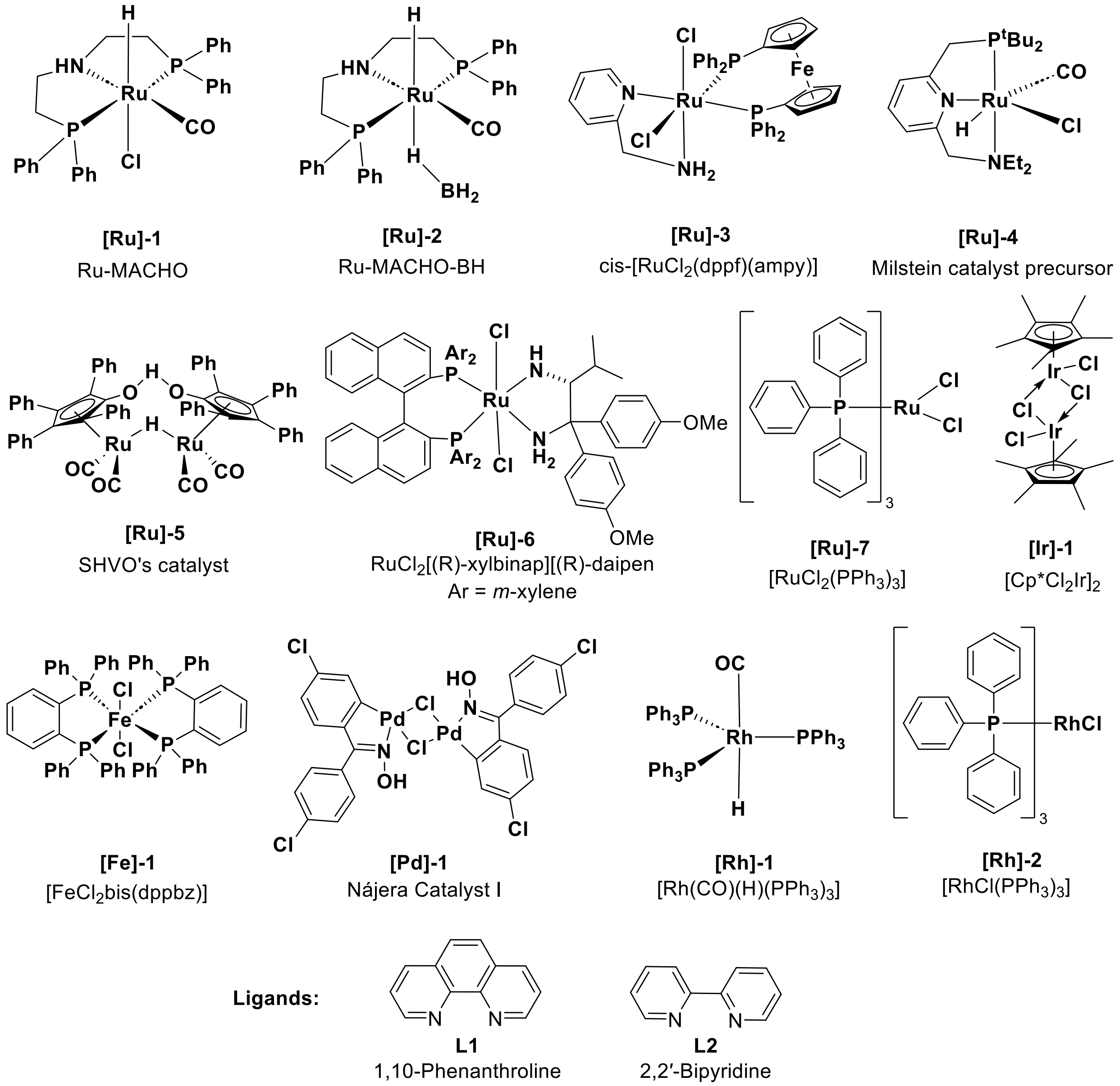

Fig.S1. Catalysts and ligands used for the screening of *N*-alkylation of amines under mechanochemical conditions.

Table S1. Screening of catalysts for the *N*-alkylation of aromatic amine **1a**.[a]

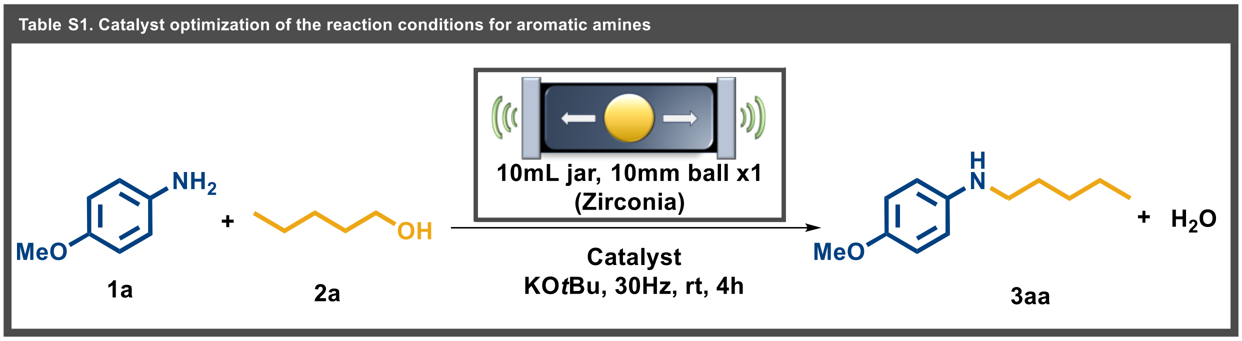

Entry	Catalyst	Ligand	Yield of 3aa[a,b]
1	[Ru]-1	–	98%
2	[Ru]-2	–	65%
3	[Ru]-3	–	73%
4	[Ru]-4	–	34%
5	[Ru]-5	–	Trace
6	[Ru]-6	–	40%
7	[Ru]-7	–	–
8	[Ir]-1	–	–
9	[Fe]-1	–	Trace
10	[Pd]-1	–	10%
11	[Rh]-1	–	7%
12	[Rh]-2	–	Trace
13	$Co_2(CO)_8$	–	–
14	$Ni(acac)_2$	L1	–
15	$NiCl_2$	L1	–
16	dppf	–	Trace
17	Fe_3O_4	–	–
18	$NiCl_2$	L2	–
19	$Co(acac)_2$	L1	–

Reaction Conditions: **[a]** *p*-anisidine (1 mmol), pentanol (1 mmol), potassium *tert*-butoxide (KO*t*Bu, 2 mmol), catalyst (2 mol%), ligand (4 mol%) in a 10mL zirconia jar with a 10mm zirconia ball milled in MM-500 Vario for 4 hours at 25°C; **[b]** All the yield determined by GC-MS.

Table S2. Optimization of the reaction conditions for the *N*-alkylation of aromatic amine **1a**

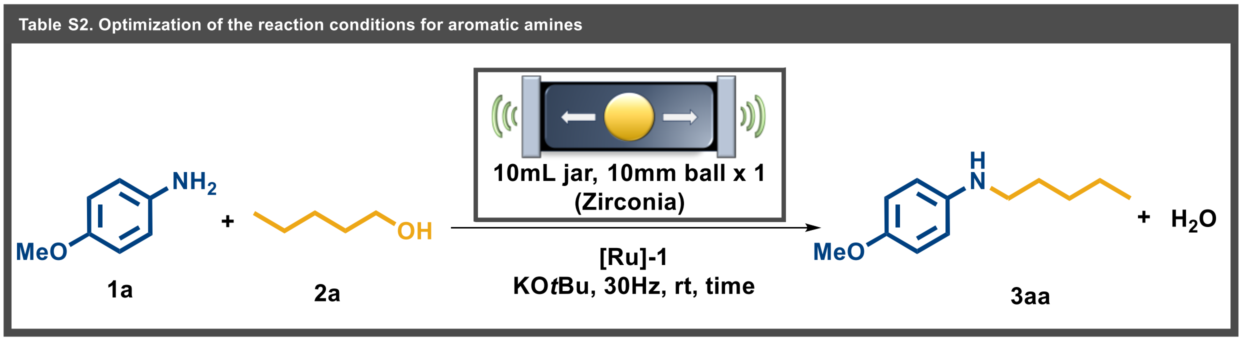

Entry	Catalyst (loading)	Base (loading)	Time (h)	Temp. (°C)	Milling Jar/ball Reaction Conditions)	Yield 3aa[a,b]
1	[Ru]-1 (0.05%)	KO*t*Bu (2 eq.)	4h	25°C	ZrO_2/ ZrO_2	82%
2	[Ru]-1 (0.05%)	KO*t*Bu (2 eq.)	6h	25°C	ZrO_2/ ZrO_2	94%
3	[Ru]-1 (0.05%)	KO*t*Bu (1 eq.)	6h	25°C	ZrO_2/ ZrO_2	94%
4	[Ru]-1 (0.05%)	KO*t*Bu (0.5 eq.)	6h	25°C	ZrO_2/ ZrO_2	50%
5[c]	[Ru]-1 (0.05%)	KO*t*Bu (1 eq.)	6h	25°C	ZrO_2/ ZrO_2	99%
6[d]	[Ru]-1 (0.05%)	KO*t*Bu (1 eq.)	6h	25°C	ZrO_2/ ZrO_2	76%
7	[Ru]-1 (0.05%)	KOH (1 eq.)	6h	25°C	ZrO_2/ ZrO_2	2%
8	[Ru]-1 (0.05%)	NaOH (1 eq.)	6h	25°C	ZrO_2/ ZrO_2	Trace
9	[Ru]-1 (0.05%)	NaOMe (1 eq.)	6h	25°C	ZrO_2/ ZrO_2	26%
10	[Ru]-1 (0.05%)	K_3PO_4 (1 Eq.)	6h	25°C	ZrO_2/ ZrO_2	–
11	[Ru]-1 (0.05%)	K_2CO_3 (1 eq.)	6h	25°C	ZrO_2/ ZrO_2	–
12	[Ru]-1 (0.05%)	Na_2CO_3 (1 eq.)	6h	25°C	ZrO_2/ ZrO_2	–
13	[Ru]-1 (0.05%)	Cs_2CO_3 (1 eq.)	6h	25°C	ZrO_2/ ZrO_2	6%
14	–	KO*t*Bu (1 eq.)	6h	25°C	ZrO_2/ ZrO_2	–
15	[Ru]-1 (0.05%)	–	6h	25°C	ZrO_2/ ZrO_2	–
16[e]	[Ru]-1 (0.05%)	KO*t*Bu (1 eq.)	6h	25°C	Toluene (4mL)	16%
17[e]	[Ru]-1 (0.05%)	KO*t*Bu (1 eq.)	18h	25°C	Toluene (4mL)	43%
18[e]	[Ru]-1 (0.05%)	KO*t*Bu (1 eq.)	24h	25°C	Toluene (4mL)	51%
19[e]	[Ru]-1 (0.05%)	KO*t*Bu (1 eq.)	12h	40°C	Toluene (4mL)	58%
20[f]	[Ru]-1 (0.05%)	KO*t*Bu (1 eq.)	24h	25°C	Neat stirring	67%
21[g]	[Ru]-1 (0.05%)	KO*t*Bu (2 eq.)	4h	25°C	SS/ SS	81%

Reaction Conditions: **[a]** *p*-anisidine (1 mmol), pentanol (1 mmol), potassium *tert*-butoxide (KO*t*Bu), **[Ru]-1** in a 10mL zirconia jar with a 10mm zirconia ball milled in MM-500 Vario for given time at 25°C; **[b]** All the yield determined by GC-MS; **[c]** reactions carried out at *p*-anisidine (4 mmol), pentanol (4 mmol), KO*t*Bu (4mmol), **[Ru]-1** (0.05 mol%) in a 10ml zirconia jar with a 10mm zirconia ball milled in MM-500 Vario at 25°C; **[d]** reaction carried out at 15 Hz; **[e]** reaction performed in toluene (1M) at standard stirring solution phase conditions; **[f]** reaction performed in neat stirring condition; **[g]** *p*-anisidine (1 mmol), pentanol (1 mmol), potassium *tert*-butoxide (KO*t*Bu), [Ru]-1 in a 10mL stainless steel (SS) jar with a 10mm SS ball milled in MM-500 Vario for given time at 25°C.

Note: Internal reaction temperature of jar before and after mechanochemical borrowing hydrogenation with *p*-anisidine (4 mmol), pentanol (4 mmol), potassium *tert*-butoxide (KO*t*Bu, 1 mmol), [Ru]-1 in a 10mL zirconia jar with a 10mm zirconia ball milled in MM-500 Vario for given time at 25°C for 6h.

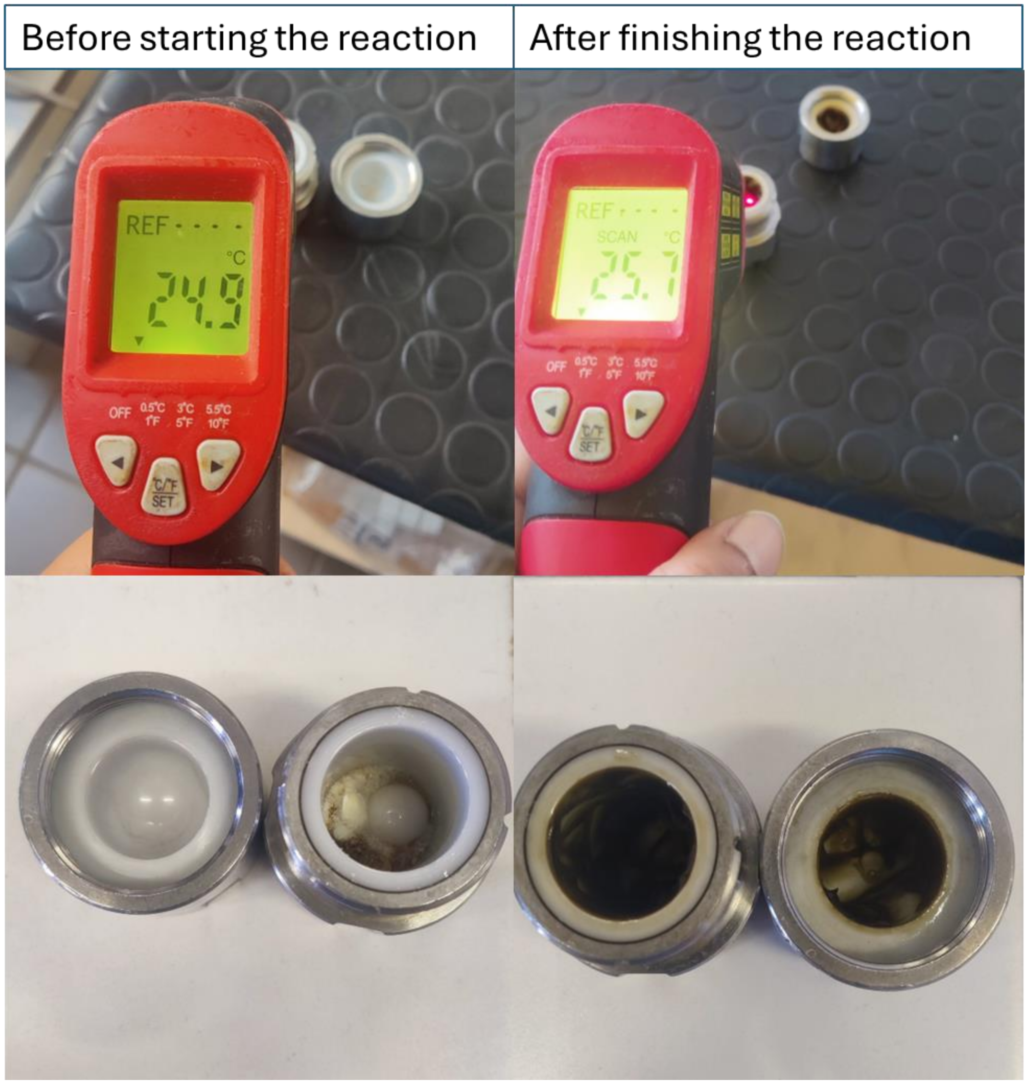

Control reaction analysis

General experimental procedure for radical trapping experiment

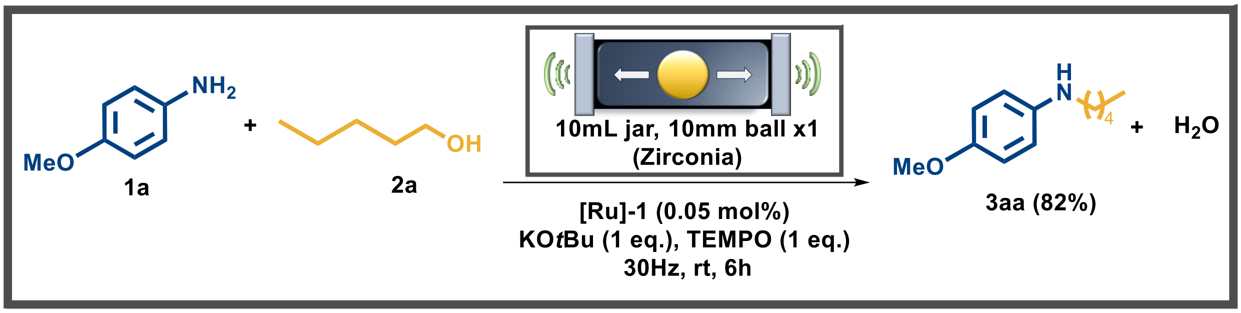

p-Anisidine (**1a**, 4 mmol), pentanol (**2a**, 4 mmol), potassium tert-butoxide (KO*t*Bu, 4 mmol), Ru-MACHO catalyst (0.05 mol%, 1.2 mg) and TEMPO (4 mmol, 625 mg) were loaded into a zirconium dioxide grinding jar (10 mL) equipped with 1 ball (ϕ = 10 mm, m 2.87 g) of the same material. The jar was sealed and milled at a frequency of 30 Hz in a shaker mill MM-500 vario at room temperature for 6 hours. After completion of the reaction, the resulting rection mixture was filtered using a sintered vacuum funnel through a small bed of celite with 20 mL of EtOAc. The filtrate was then evaporated under high vacuum.

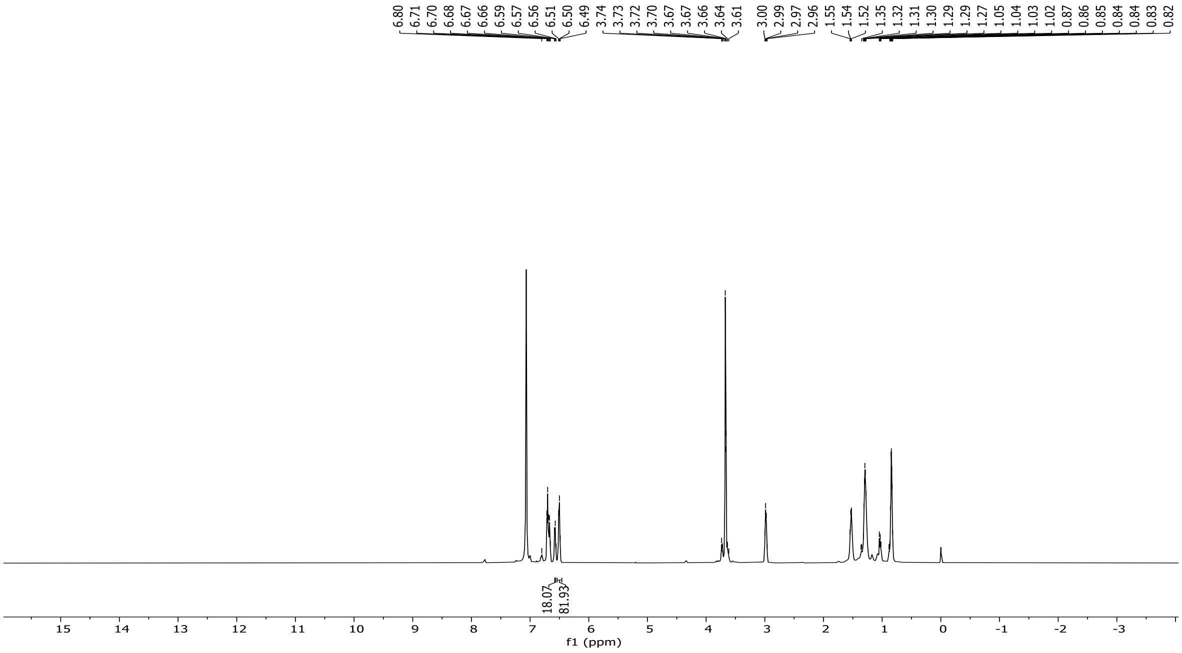

Figure S2. ^1H NMR (600 MHz, $CDCl_3$) spectrum of radical trapping experiment, amine **3aa**.

Kinetic isotopic effect analysis

General experimental procedure for deuteration of alcohol

Alcohol (1 mmol), potassium *tert*-butoxide (5 mol%), D_2O (300 μL) and Ru-MACHO catalyst (0.2 mol%, 1.2 mg) were loaded into a glass vial (5 mL) equipped with 10 balls (ϕ = 3 mm) of the same material. The jar was purged with argon gas, sealed and milled at 30 Hz in a shaker mill Fritsch P-23 for 3 hours at a temperature of 80°C using a heat gun. Then, the reaction jar was cooled down to room temperature. After completion of the reaction, the resulting reaction mixture was extracted by separating funnel using DCM (3x2 mL) **without the addition of any further water**. The DCM layer was then dried using sodium sulfate and the resultant filtrate was evaporated under high vacuum. ^{1}H-NMR and ^{13}C spectrometry confirmed the deuteration of the alcohol to **2i-d$_3$** up to 99%.

^{1}H NMR (600 MHz, $CDCl_3$) δ 7.37 (d, J = 4.8 Hz, 4H), 7.33 – 7.28 (m, 1H); HRMS (ESI) m/z calc. for $C_7H_5D_2O$ $[M-H]^-$: 109.0628, found: 109.0619.

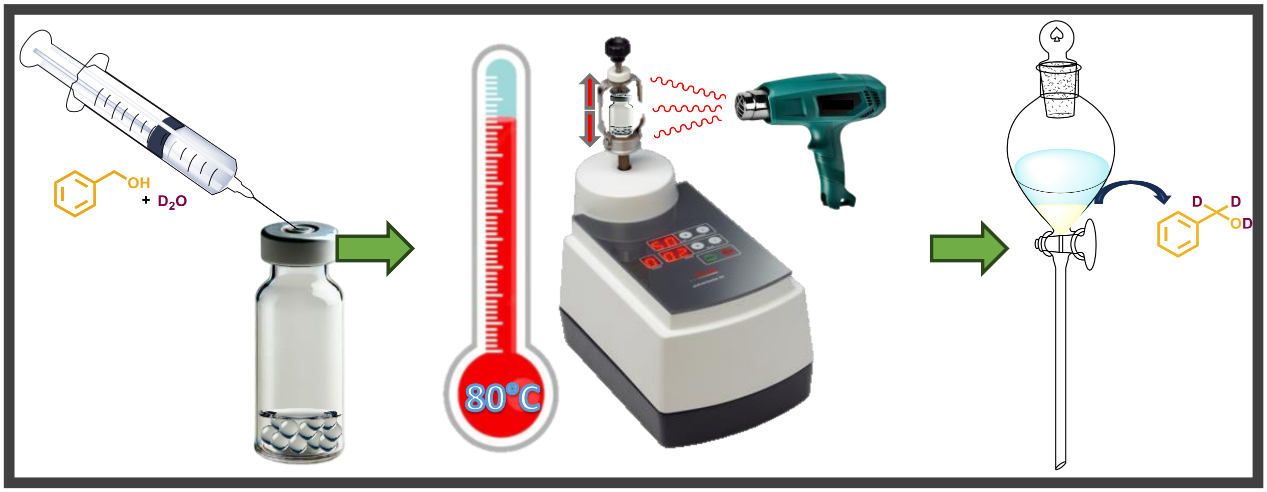

Figure S3. Reaction scheme for the synthesis of benzyl alcohol-d$_3$ (**2i-d$_3$**).

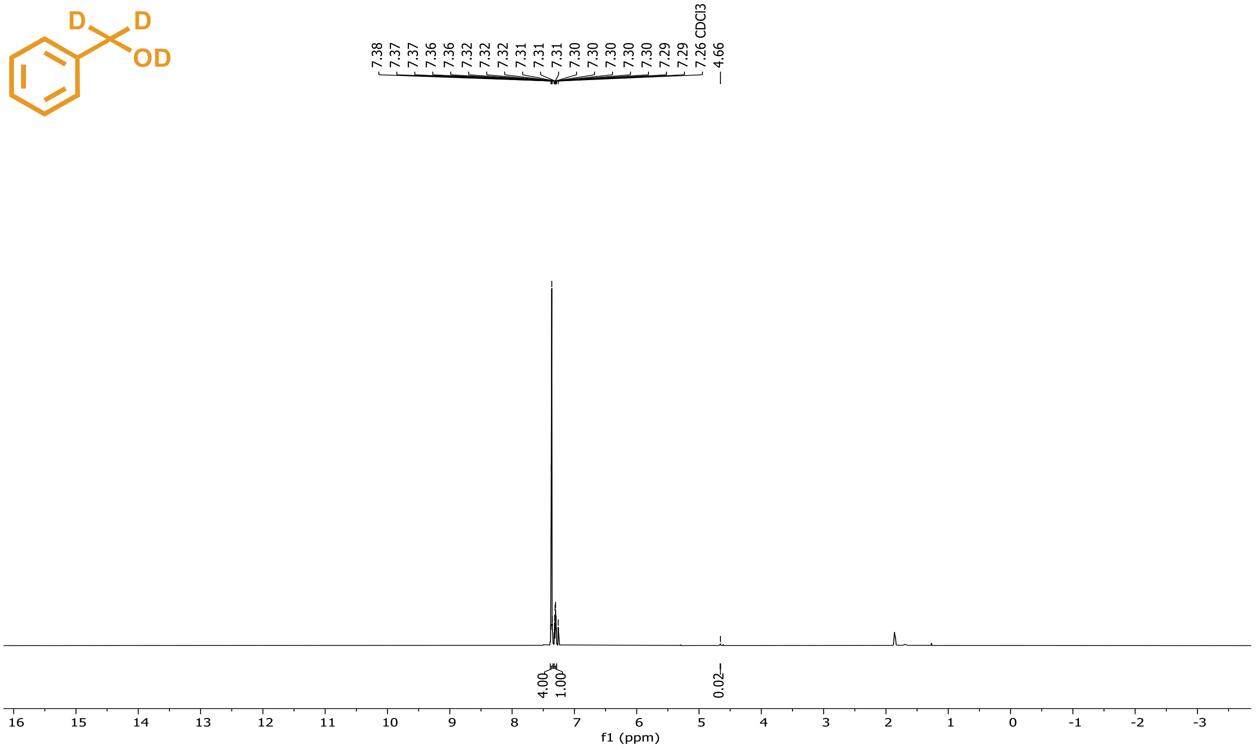

Figure S4. ^{1}H NMR (600 MHz, $CDCl_3$) spectrum of **benzyl alcohol-d$_3$ (2i-d$_3$).**

General set-up for kinetic isotopic effect analysis

p-Anisidine (**1a**, 4 mmol), benzyl alcohols (**2i/ 2i-d$_3$**, 4 mmol), potassium tert-butoxide (KO*t*Bu, 4 mmol) and Ru-MACHO catalyst (0.05 mol%, 1.2 mg) were loaded into a zirconium dioxide grinding jar (10 mL) equipped with 1 ball (ϕ = 10 mm, m 2.87 g) of the same material. The jar was sealed and milled at 30Hz in a shaker mill MM-500 vario at room temperature. All the reaction experiments were carried out separately with an interval of 30 minutes from time ranging from 0 minutes to 150 minutes. After completion of the reaction, the resulting rection mixture was filtered using a sintered vacuum funnel through a small bed of celite with 20 mL of EtOAc. The filtrates were evaporated under high vacuum. The products were then subjugated to quantitative ^{1}H-NMR spectrometry using ethylene carbonate (2 mmol, 176 mg) as an internal standard[1] in $CDCl_3$. The reaction coefficients (k) were measured and plotted. Finally, the *KIE* is measured by the formula k_H/k_D obtainable from the slope between $\ln([C_{2i\text{-}d3(0)}]/[C_{2i\text{-}d3(t)}])$ and $\ln([C_{2i(0)}]/[C_{2i(t)}])$, where $[C_{2i\text{-}d3(0)}]$ and $[C_{2i\text{-}d3(t)}]$ are the initial concentration and concentration at time '*t*' of **2i-d$_3$** respectively; contrast $[C_{2i(0)}]$ and $[C_{2i(t)}]$ represents the initial concentration and concentration at time '*t*' of **2i** respectively.

Table S3. KIE calculation.

Time (sec)	$[C_{2i}]$	$\ln([C_{2i(0)}]/[C_{2i(t)}])$	$[C_{2i\text{-}d3}]$	$\ln([C_{2i\text{-}d3(0)}]/[C_{2i\text{-}d3(t)}])$
0	1	0	1	0
1800	0.65	0.43078	0.75	0.28768
3600	0.44	0.82098	0.68	0.38566
5400	0.36	1.02165	0.41	0.8916
7200	0.29	1.23787	0.37	0.99425
9000	0.22	1.51413	0.3	1.20397

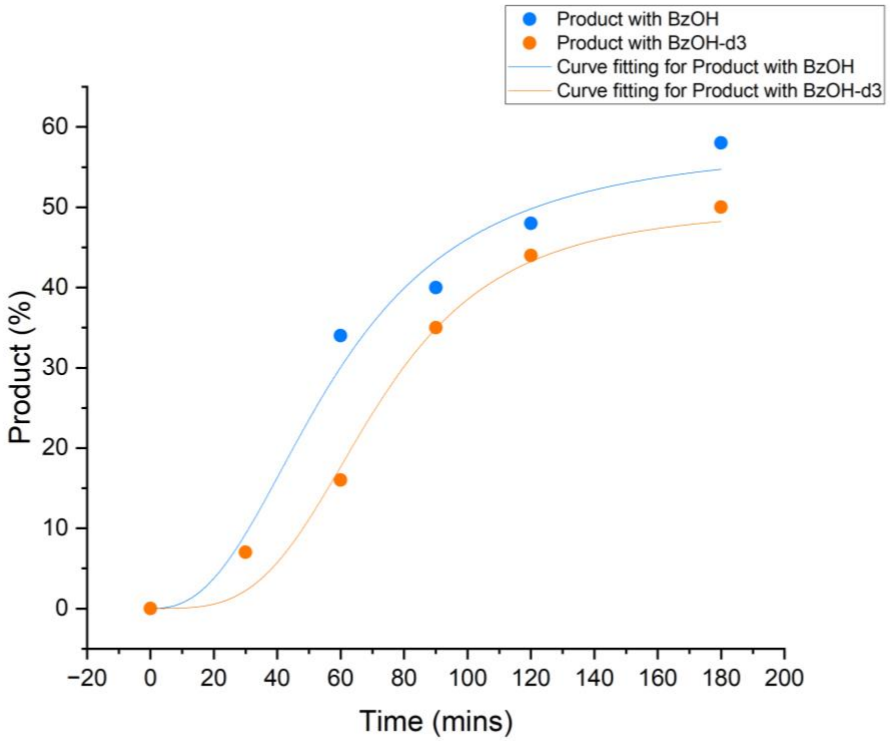

Figure S5. Product evolution of **3ai** and **3ai-d₃** with respect to time.

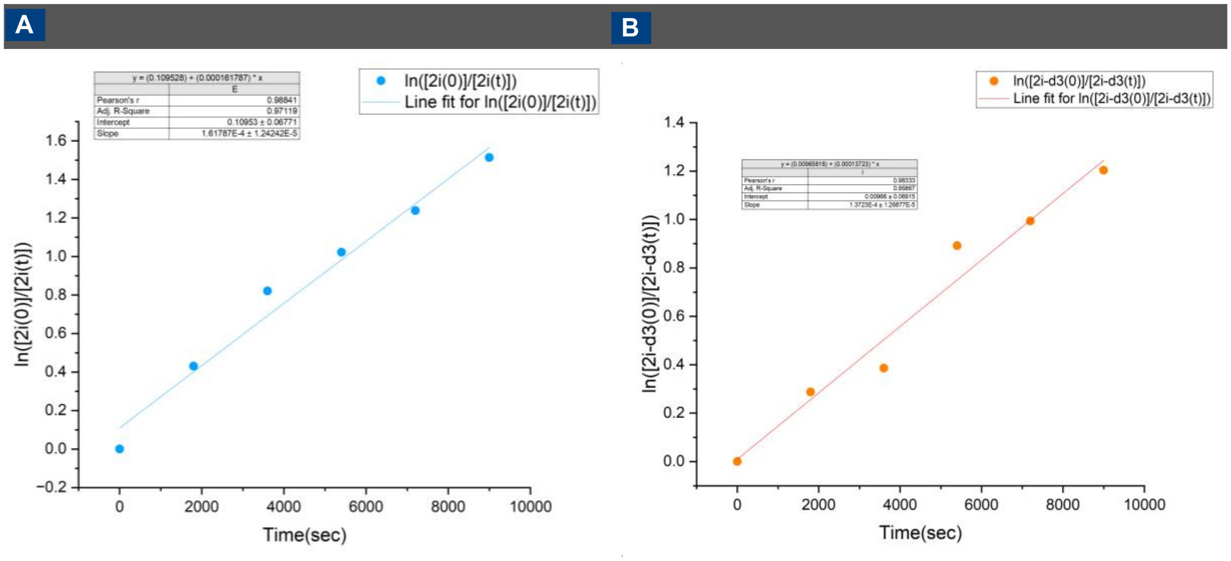

Figure S6. A. ln([2i(0)]/[2i(t)]) *vs* time: where, [2i(0)] and [2i(t)] represents the initial concentration and concentration at time '*t*' of **2i** respectively; **B.** ln([2i-d3(0)]/[2i-d3(t)]) vs time: where [2i-d3(0)] and [2i-d3(t)] are the initial concentration and concentration at time 't' of **2i-d$_3$** respectively.

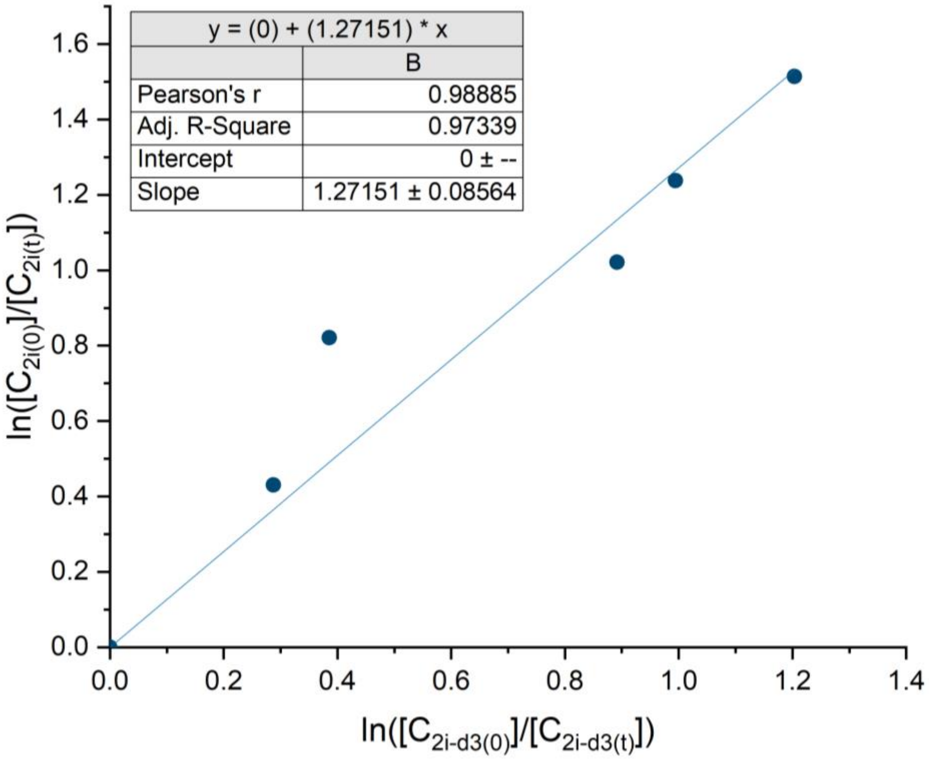

Figure S7. ln([$C_{2i\text{-}d3(0)}$]/[$C_{2i\text{-}d3(t)}$]) vs ln([$C_{2i(0)}$]/[$C_{2i(t)}$]) plot to find the value of *KIE*= 1.27±0.09 (R^2= 0.97).

NMR spectra for KIE.

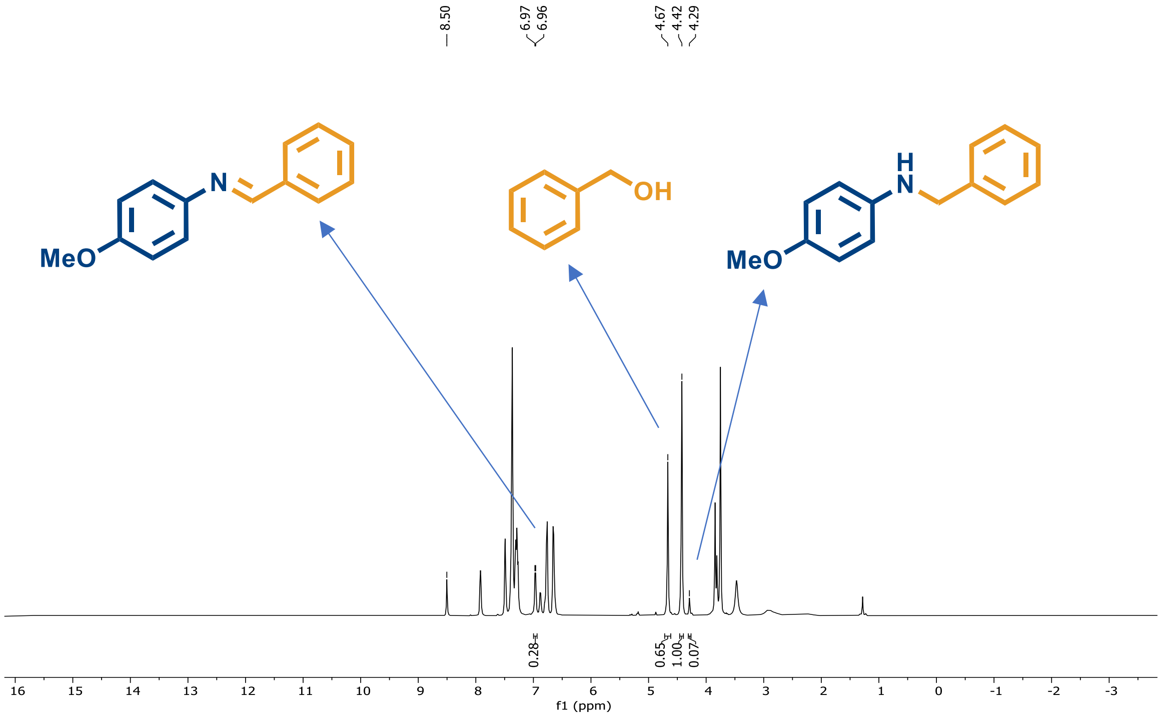

Figure S8. Quantitative ^{1}H-NMR for **3ai**, t=30 minutes with ethylene carbonate as internal standard.

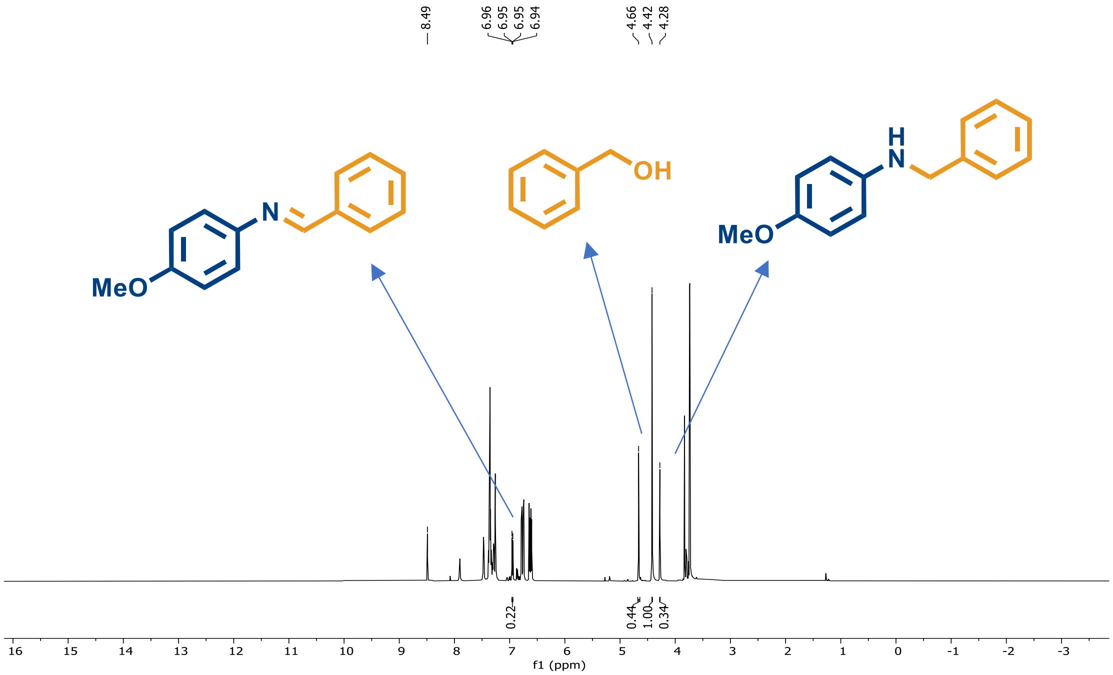

Figure S9. Quantitative ^{1}H-NMR for **3ai**, t=60 minutes with ethylene carbonate as internal standard.

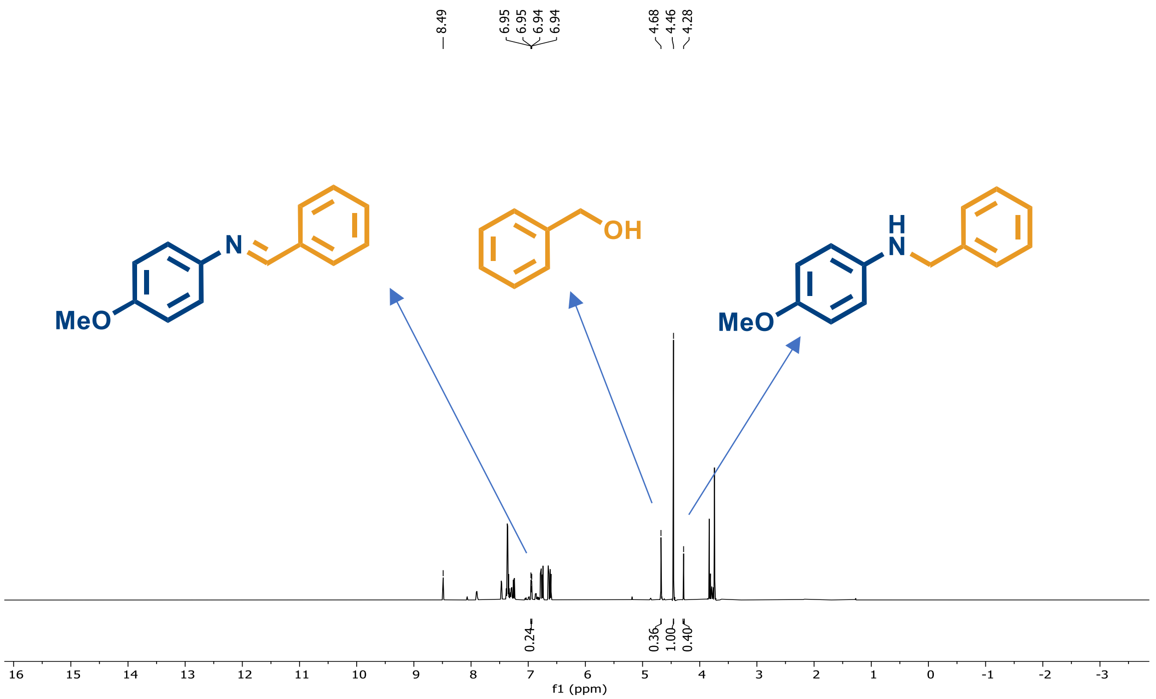

Figure S10. Quantitative ^{1}H-NMR for **3ai**, t=90 minutes with ethylene carbonate as internal standard.

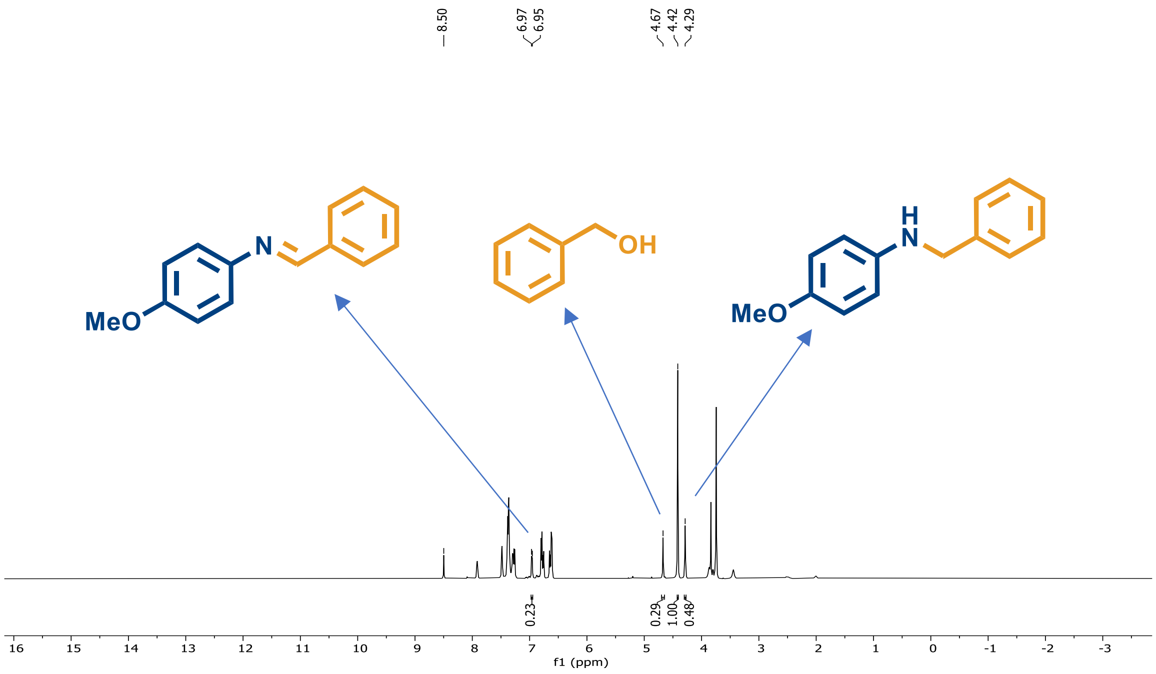

Figure S11. Quantitative ^{1}H-NMR for **3ai**, t=120 minutes with ethylene carbonate as internal standard.

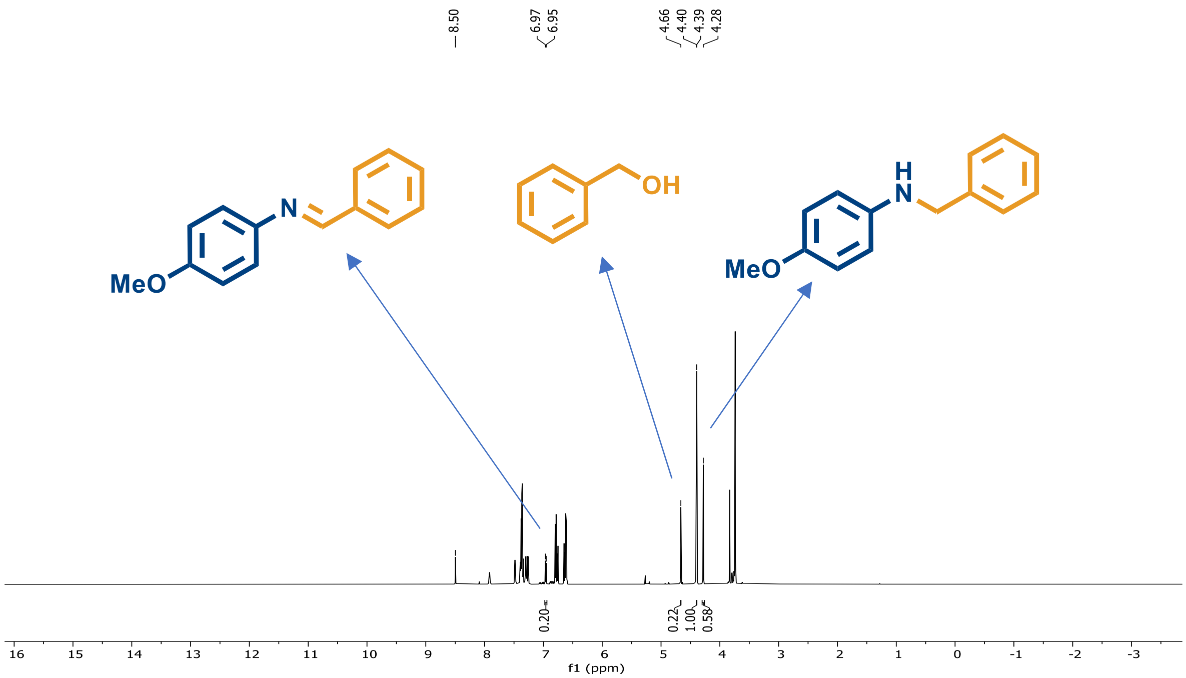

Figure S12. Quantitative ^{1}H-NMR for **3ai**, t=150 minutes with ethylene carbonate as internal standard.

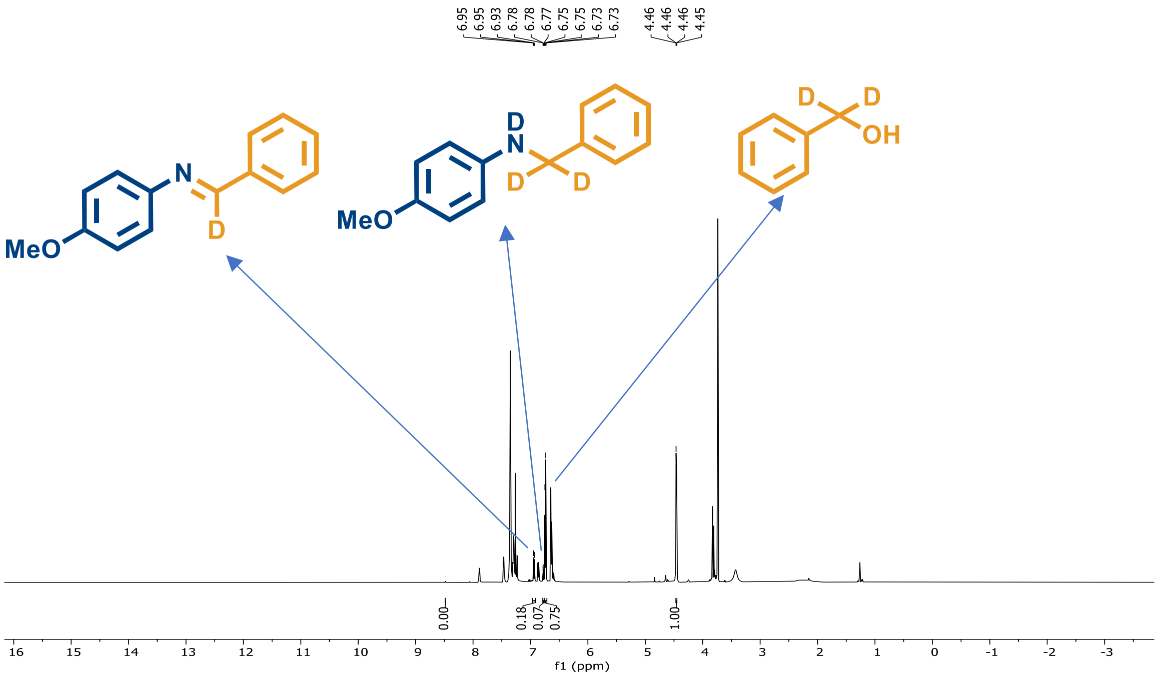

Figure S13. Quantitative ^{1}H-NMR for **3ai-d$_3$**, t=30 minutes with ethylene carbonate as internal standard.

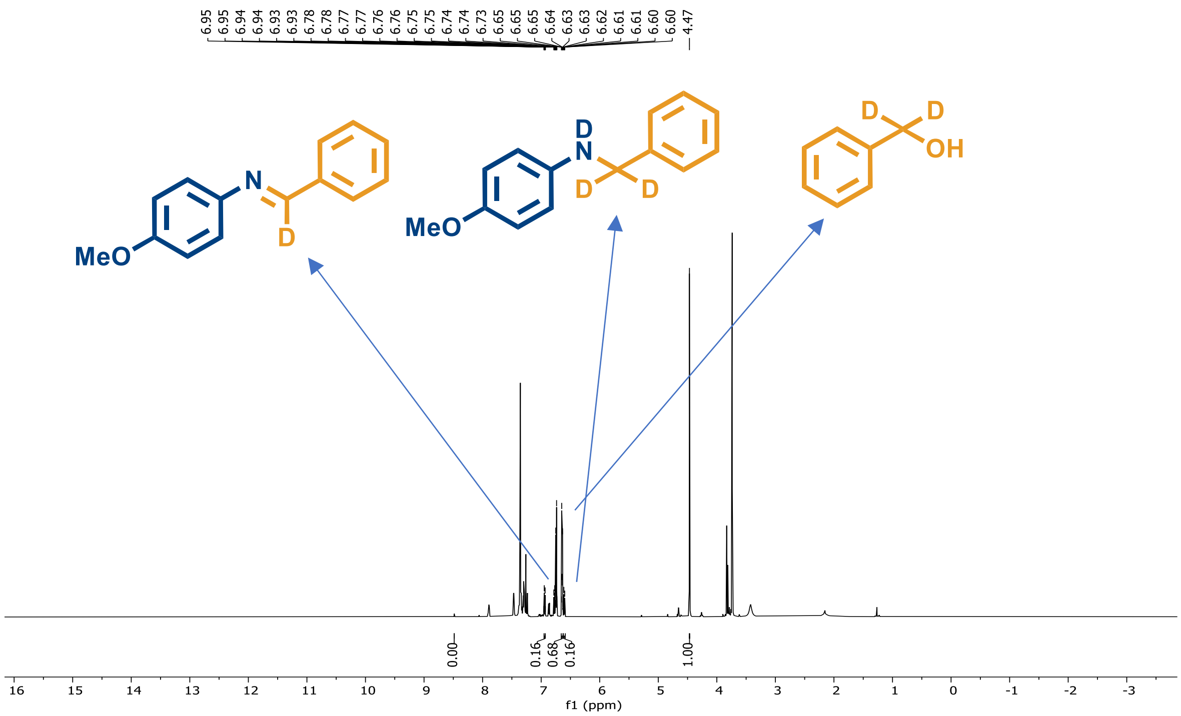

Figure S14. Quantitative ^{1}H-NMR for **3ai-d$_3$**, t=60 minutes with ethylene carbonate as internal standard.

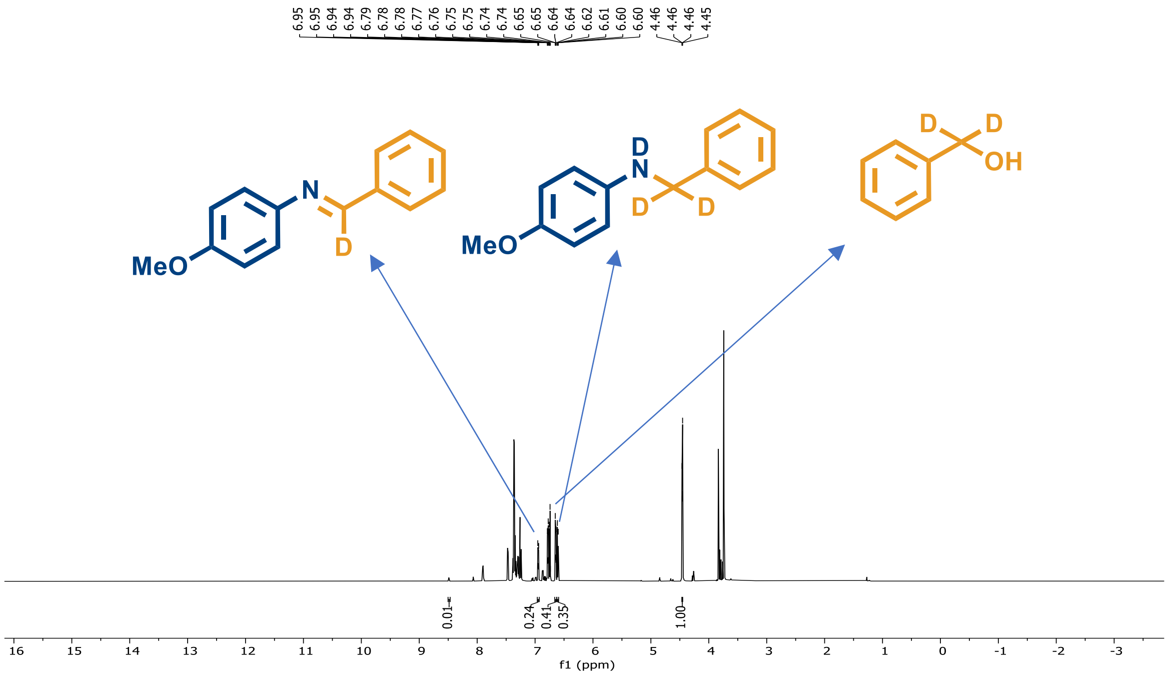

Figure S15. Quantitative ^{1}H-NMR for **3ai-d$_3$**, t=90 minutes with ethylene carbonate as internal standard.

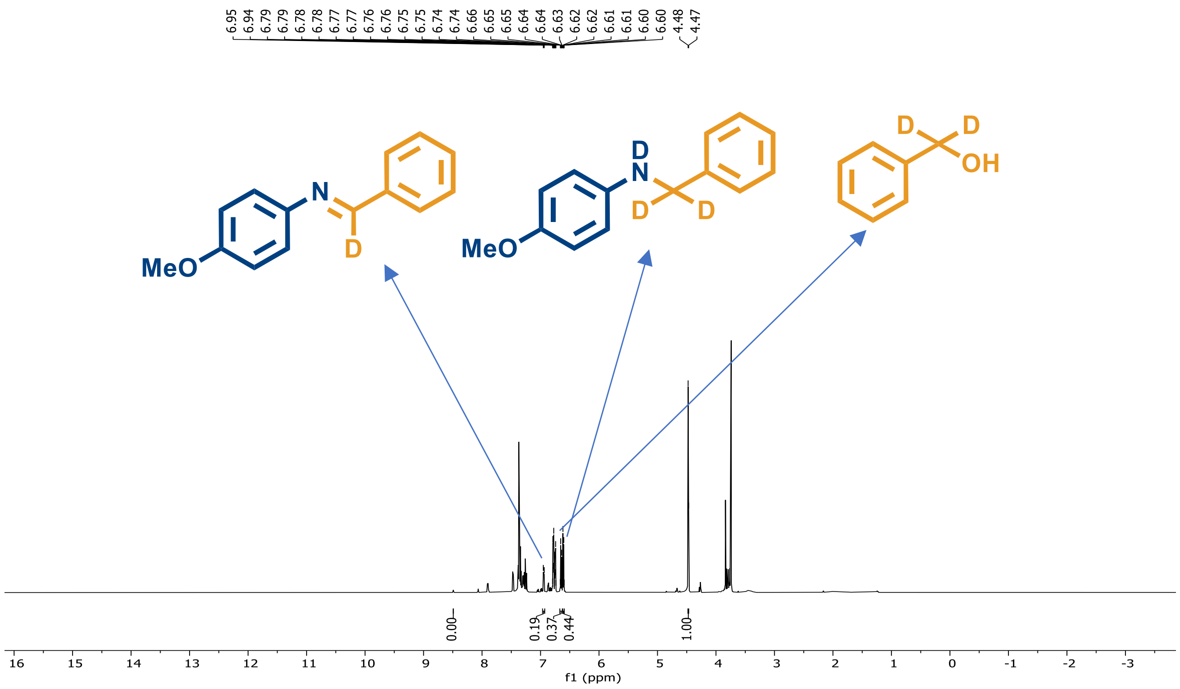

Figure S16. Quantitative ^{1}H-NMR for **3ai-d₃**, t = 120 minutes with ethylene carbonate as internal standard.

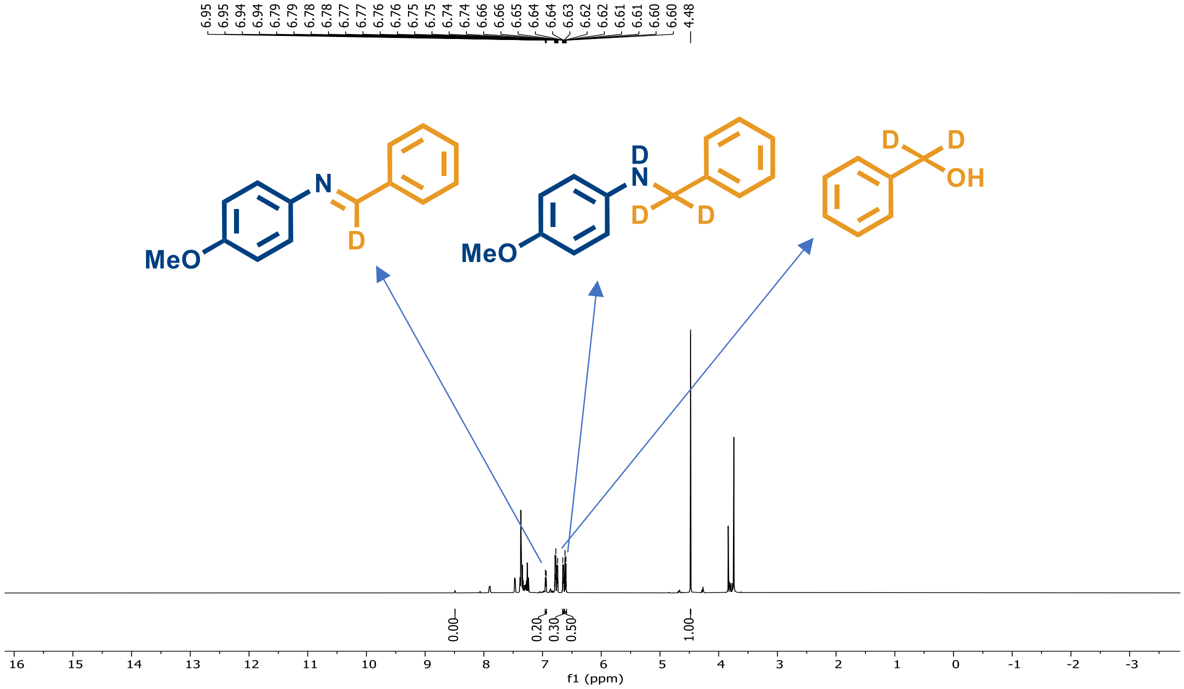

Figure S17. Quantitative ^{1}H-NMR for **3ai-d₃**, t=150 minutes with ethylene carbonate as internal standard.

Hammett analysis

General set-up for Hammett analysis

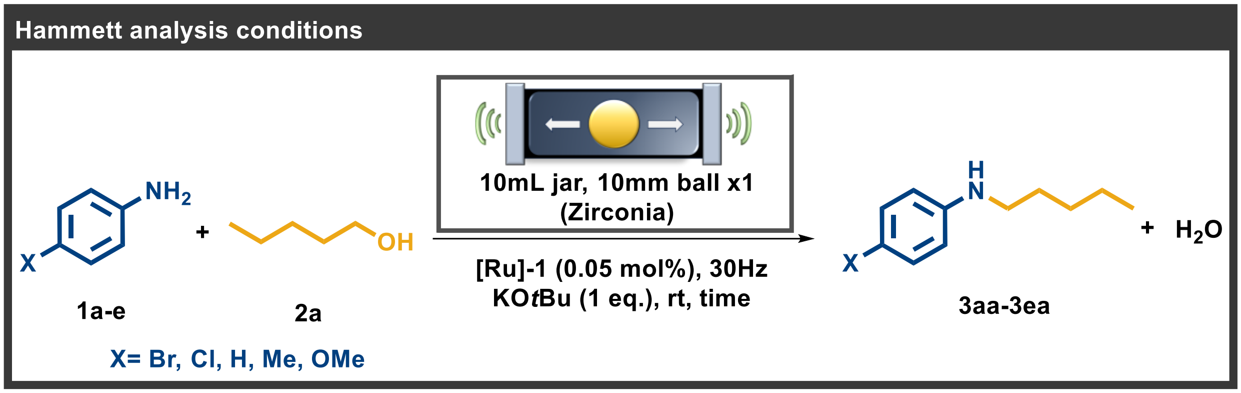

Amine (**1a-e**, 4 mmol), pentanol (**2a**, 4 mmol), potassium *tert*-butoxide (KO*t*Bu, 4 mmol) and Ru-MACHO catalyst (0.05 mol%, 1.2 mg) were loaded into a zirconium dioxide grinding jar (10 mL) equipped with 1 ball (ϕ = 10 mm, m 2.87 g) of the same material. The jar was sealed and milled at 30Hz in a shaker mill MM-500 Vario at room temperature. All the reaction experiments were carried out separately with an interval of 10 minutes from time ranging from 0 minutes to 60 minutes. After completion of the reactions, the resulting rection mixture was filtered using a sintered vacuum funnel through a small bed of celite with 20 mL of EtOAc. The filtrate was then evaporated under high vacuum. The products were then subjugated to quantitative ^{1}H-NMR spectrometry using ethylene carbonate (2 mmol, 176 mg) as an internal standard in $CDCl_3$. The reaction rate constants (k) were calculated and $\log(k_X/k_H)$ were plotted against substituent constants (**σ**) to analyze the dependency of the substituent groups on anilines upon the product formation.

Table S4. Reaction rate coefficient calculation for para substituted anilines (**1a-e**) with pentanol (**2a**).

**Time (sec)**	**$[C_{X=Br}]$**	$\ln([C_{X=Br(0)}]/[C_{X=Br(t)}])$	**$[C_{X=Cl}]$**	$\ln([C_{X=Cl(0)}]/[C_{X=Cl(t)}])$	**$[C_{X=H}]$**	$\ln([C_{X=H(0)}]/[C_{X=H(t)}])$	**$[C_{X=Me}]$**	$\ln([C_{X=Me(0)}]/[C_{X=Me(t)}])$	**$[C_{X=OMe}]$**	$\ln([C_{X=OMe(0)}]/[C_{X=OMe(t)}])$
0	1	0	1	0	1	0	1	0	1	0
600	0.70	0.35667	0.71	0.34249	0.74	0.30111	0.80	0.22314	0.93	0.07257
1200	0.53	0.63488	0.56	0.57982	–	–	0.69	0.37106	0.83	0.18633
1800	0.35	1.04982	–	–	0.41	0.8916	0.57	0.56212	0.71	0.34249
2400	0.22	1.51413	0.24	1.42712	0.30	1.20397	0.49	0.71335	0.61	0.4943
3000	0.14	1.96611	0.17	1.77196	0.26	1.34707	0.39	0.94161	0.53	0.63488
3600	0.08	2.52573	0.10	2.30259	0.22	1.51413	0.33	1.10866	0.47	0.75502

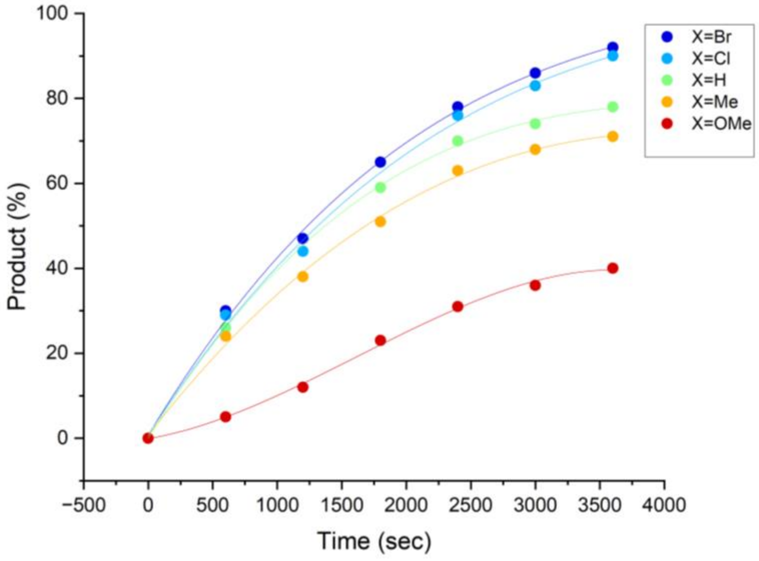

Figure S18. Product evolution over time for Hammett plot for different substituents in aniline.

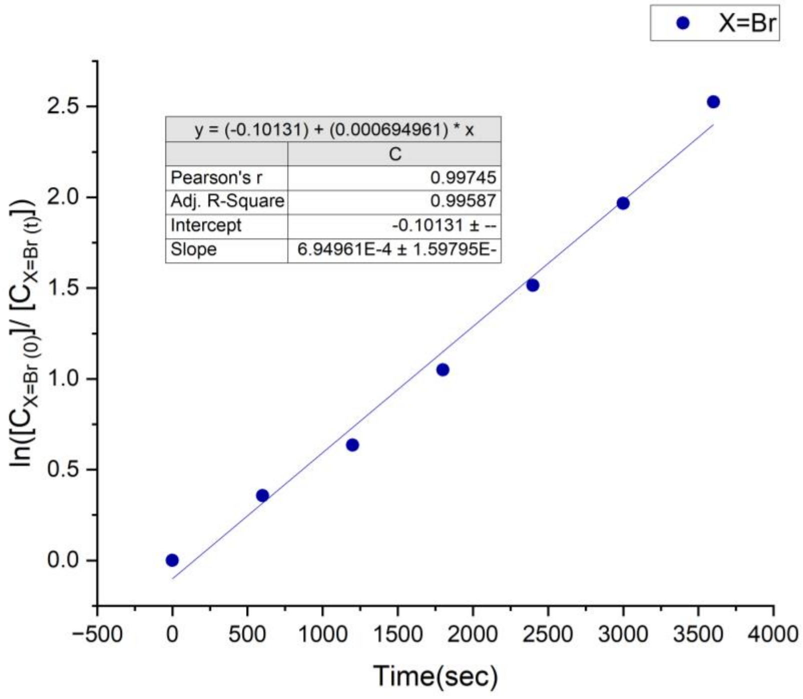

Figure S19. Plot between ln([$C_{X=Br(0)}$]/[$C_{X=Br(t)}$]) vs time to get $k_{X=Br}$.

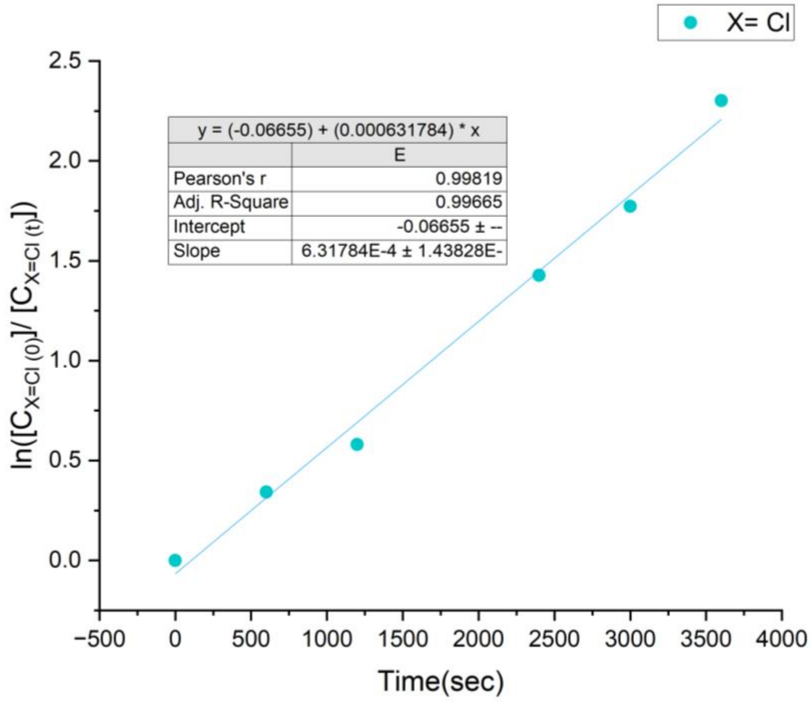

Figure S20. Plot between ln([$C_{X=Cl(0)}$]/[$C_{X=Cl(t)}$]) *vs* time to get $k_{X=Cl}$.

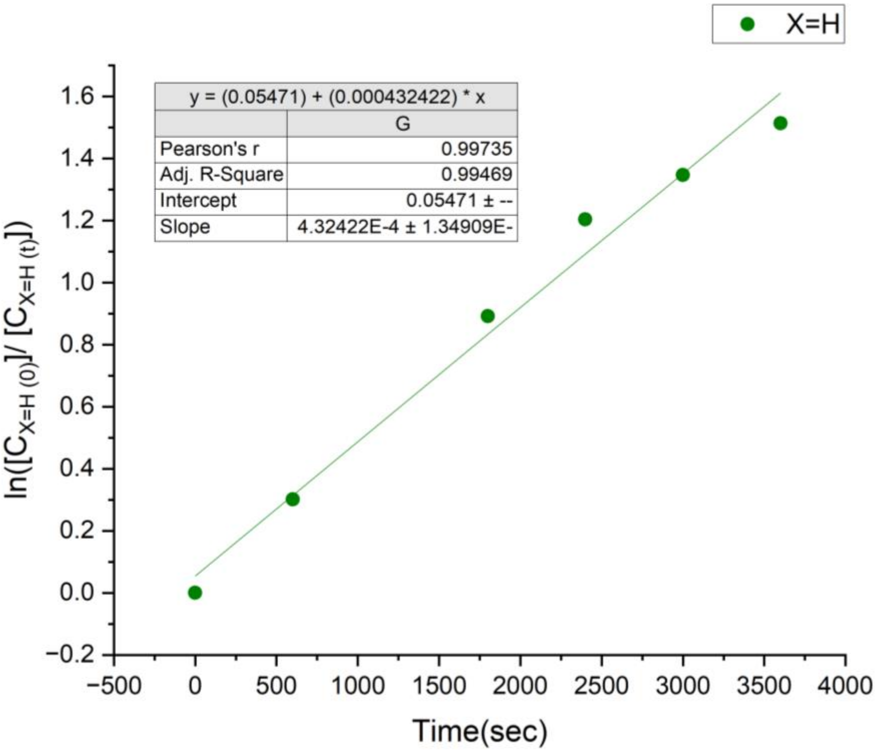

Figure S21. Plot between $\ln([C_{X=H(0)}]/[C_{X=H(t)}])$ *vs* time to get $k_{X=H}$.

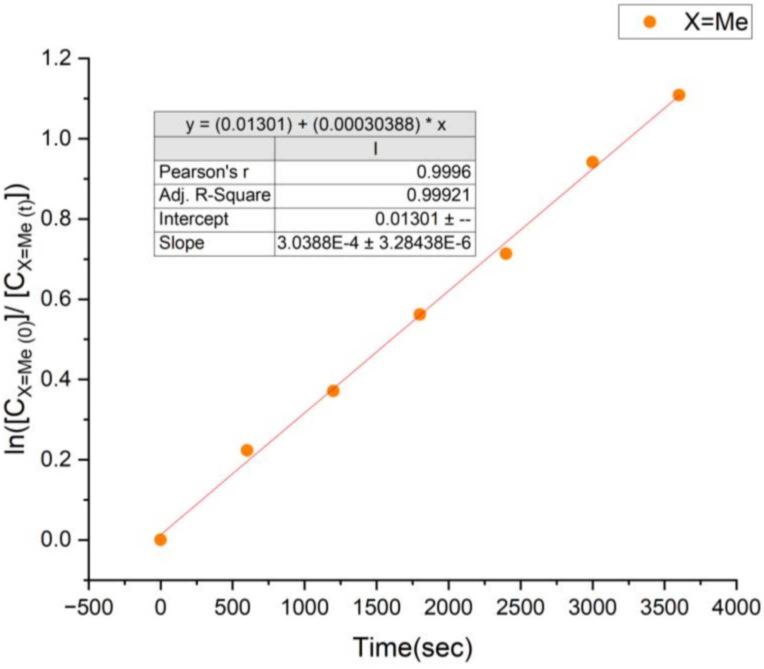

Figure S22. Plot between $\ln([C_{X=Me(0)}]/[C_{X=Me(t)}])$ vs time to get $k_{X=Me}$.

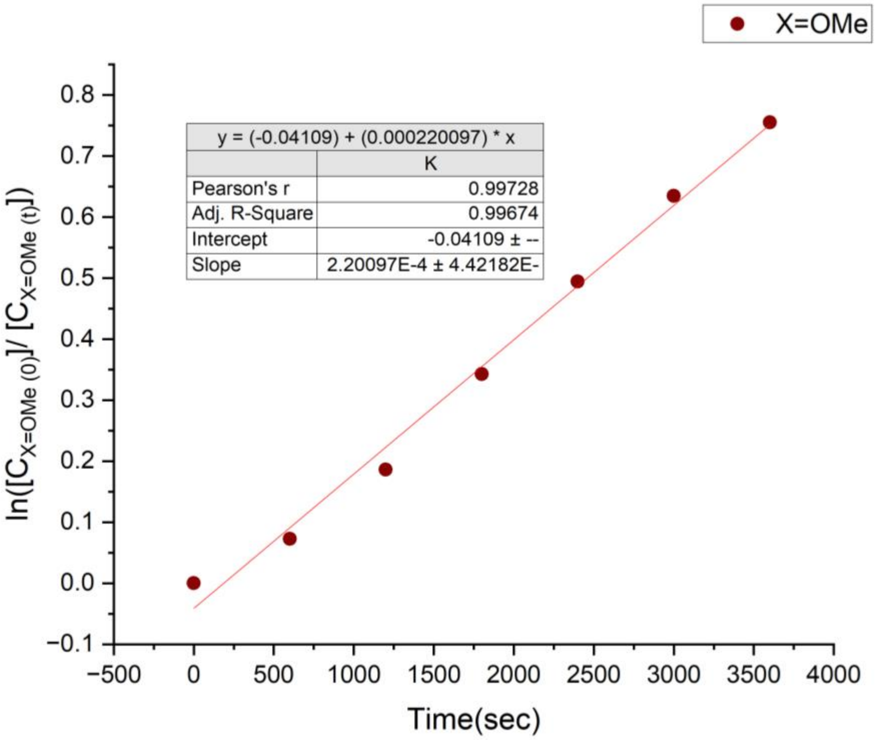

Figure S23. Plot between ln([$C_{X=OMe(0)}$]/[$C_{X=OMe(t)}$]) *vs* time to get $k_{X=OMe}$.

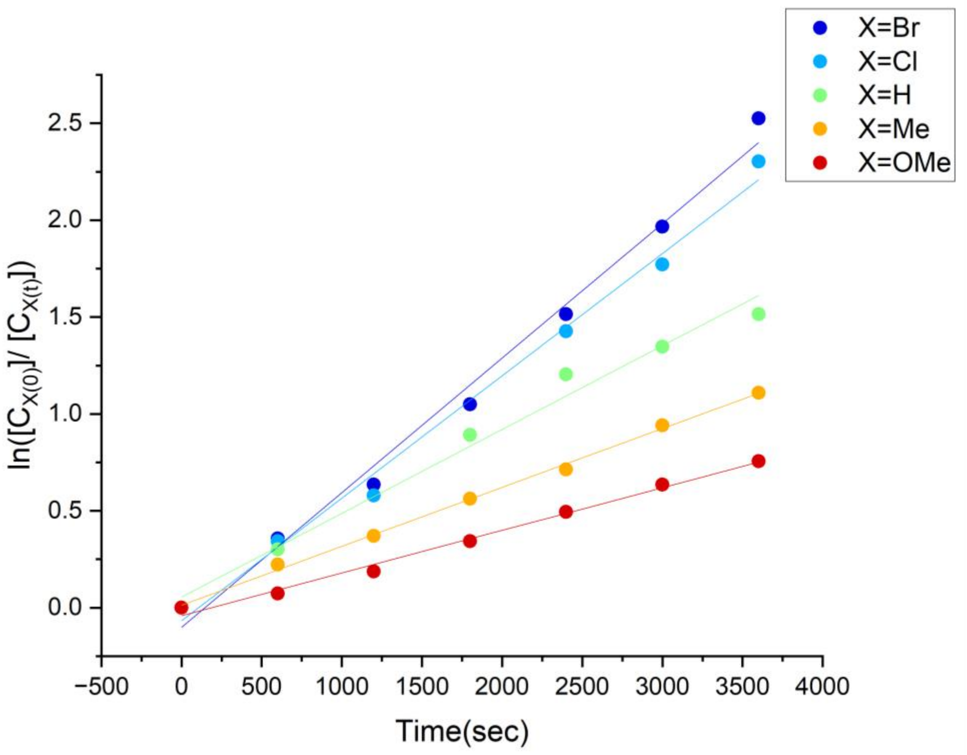

Figure S24. Plot between ln([$C_{X(0)}$]/[$C_{X(t)}$]) *vs* time for X=Br, Cl, H, Me, OMe.

Table S5. Hammett analysis.

X	Substituent Constant (σ)	$\log(k_X/k_H)$
Br	0.232	0.20605
Cl	0.227	0.16466
H	0	0
Me	-0.17	-0.15321
OMe	-0.268	-0.29329

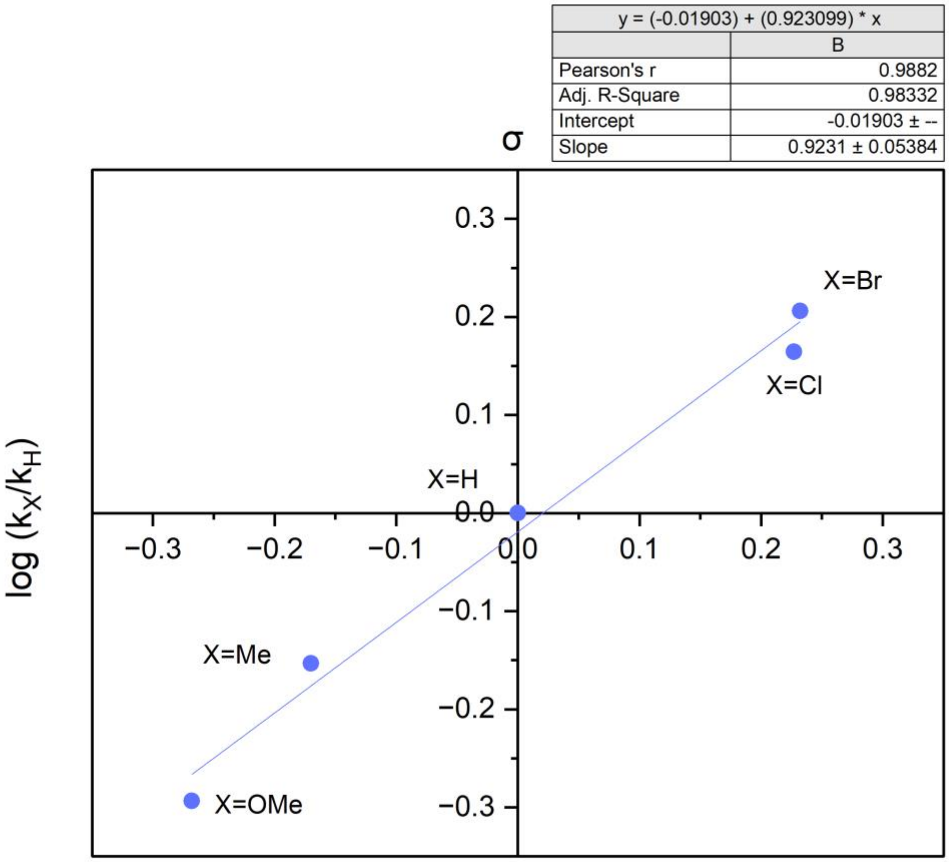

Figure S25. Hammett plot between log (**k**$_X$/**k**$_H$) with substituent constants (**σ**) where X=Br, Cl, H, Me, OMe.

Green Chemistry Metrics Calculations

Calculation of the Green Chemistry Metrics for the Mechanochemical Preparation of amine **3aa**

Environmental Factor (**E**) for the mechanochemical preparation of amine **3aa**

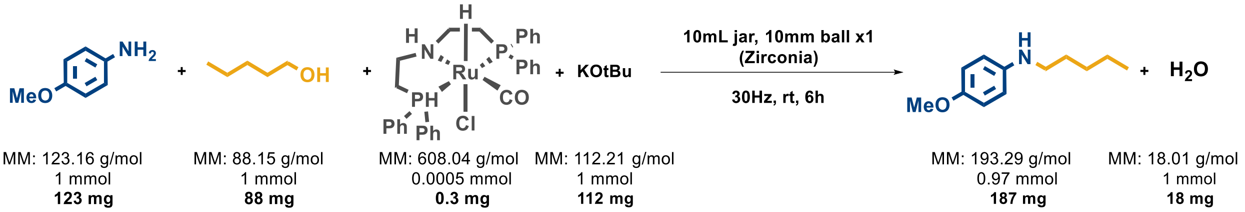

Scheme S1. Mechanochemical 4-methoxy-*N*-pentylaniline (**3aa**)

$$\text{Enviromental factor } (E) = \frac{\textit{mass of total waste}}{\textit{mass of desired product}} = \frac{(123 + 88 + 0.3 + 112 - 187)\text{ mg}}{187\text{ mg}} = \frac{136\text{ mg}}{187\text{ mg}} = \mathbf{0.7}$$

Work-up: 4510 mg EtOAc and 1000 mg celite

$$\boldsymbol{E}\text{ (after purification, silica filtration work-up)} = \frac{(5510 + 136)\text{ mg}}{187\text{ mg}} = \frac{5646\text{ mg}}{187\text{ mg}} = \mathbf{30}$$

Environmental Factor (E) for the preparation of amine **3aa** based on solution synthesis[2]

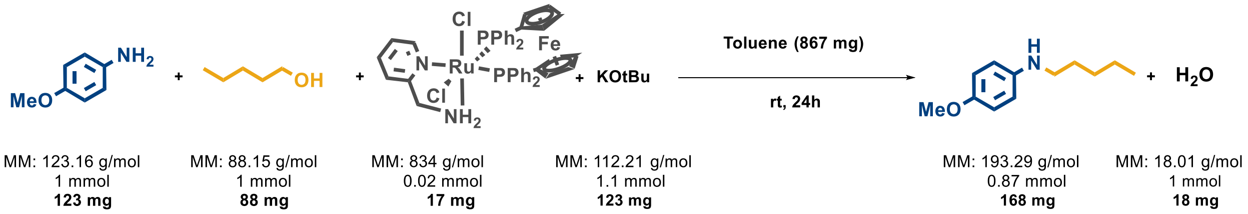

Scheme S2. Mechanochemical 4-methoxy-*N*-pentylaniline (**3aa**)

$$\text{Enviromental factor } (E) = \frac{\textit{mass of total waste}}{\textit{mass of desired product}} = \frac{(123 + 88 + 17 + 123 + 867 - 168)\text{ mg}}{168\text{ mg}} = \frac{1050\text{ mg}}{168\text{ mg}} = \mathbf{6.3}$$

Work-up: 59490 mg *n*-hexane, 9020 mg EtOAc and 20000 mg silica

$$\boldsymbol{E}\text{ (after purification, column chromatography} = \frac{(88510 + 1050)\text{ mg}}{168\text{ mg}} = \frac{89560\text{ mg}}{168\text{ mg}} = \mathbf{533}$$

The Eco-scale Score for the Mechanochemical Preparation of Amine **3aa**

EcoScale: 100 – sum of penalty points

Table S6. Calculation of Ecoscale score for the Mechanochemical Preparation of Amine **3aa** [a]

Reagents	MF	MW	g	mmol	Equiv.
p-Anisidine	C_7H_9NO	123.15	0.123	1.0	1.0
1-Pentanol	$C_5H_{12}O$	88.15	0.088	1.0	1.0
Potassium *tert*-butoxide	C_4H_9KO	112.21	0.112	1.0	1.0
Ru-MACHO	$C_{29}H_{30}ClNOP_2Ru$	607.03	0.0003	0.0005	0.0005
**Product**	**MF**	**MW**	**g**	**mmol**	**Yield**
4-methoxy-*N*-pentylaniline (**3aa**)	$C_{12}H_{19}NO$	193.29	0.193	0.97	97%

Entry	Parameters	Penalty Points
1	Yield (97%)	-2
2	Price/availability	-5
3	Safety	-20
4	Technical set-up (Unconventional activation technique)	-2
5	Room temperature (< 24h)	-1
6	Work-up and purification	0
	**EcoScale Score**	**70**

[a]Values calculated using the eco scale calculator software available at the link: http://ecoscale.cheminfo.org/calculator

The Eco-scale Score for the of Amine **3aa** based on solution

Reagents	MF	MW	g	mmol	Equiv.
p-Anisidine	C_7H_9NO	123.15	0.123	1	1.0
1-Pentanol	$C_5H_{12}O$	88.15	0.088	1	1
Potassium tert-butoxide	C_4H_9KO	112.21	0.123	1.1	1.1
cis-[RuCl$_2$(ampy)(dppf)]	$C_{40}H_{36}Cl_2FeN_2P_2Ru$	834.50	0.017	0.02	0.02
Toluene	C_7H_8	92.14	0.866	9.4	9.4
**Product**	**MF**	**MW**	**g**	**mmol**	**Yield**
4-methoxy-N-pentylaniline (**3aa**)	$C_{12}H_{19}NO$	193.29	0.168	0.87	87%

Entry	Parameters	Penalty Points
1	Yield (87%)	-6.5
2	Price/availability	-5
3	Safety	-25
4	Technical set-up (Common set-up)	0
5	Room temperature (< 24h)	-1
6	Classical chromatography	-10
	**EcoScale Score**	**52.5**

Spectral data for products

Spectral data for aromatic amines **3aa-3am**

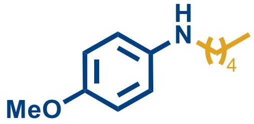

4-methoxy-*N*-pentylaniline (3aa) Pale yellow oil, Yield: 749 mg, 97%. ^{1}H NMR (600 MHz, $CDCl_3$) δ 6.82 – 6.76 (m, 2H), 6.62 – 6.57 (m, 2H), 3.75 (s, 3H), 3.07 (t, *J* = 7.2 Hz, 2H), 1.62 (dq, *J* = 11.1, 6.6 Hz, 2H), 1.46 – 1.27 (m, 4H), 0.93 (t, *J* = 7.1 Hz, 3H). ^{13}C NMR (151 MHz, $CDCl_3$) δ 152.3, 142.8, 115.0, 114.3, 55.9, 45.2, 29.5, 29.5, 22.6, 14.1. Spectroscopic data are in agreement with those reported earlier.[3]

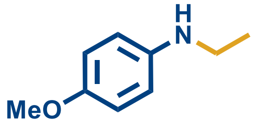

***N*-ethyl-4-methoxyaniline (3ab)** Colourless oil, Yield: 522 mg, 86%.^{1}H NMR (600 MHz, $CDCl_3$) δ 6.79 (d, *J* = 8.9 Hz, 2H), 6.65 (d, *J* = 8.9 Hz, 2H), 3.75 (s, 3H), 3.13 (q, *J* = 7.1 Hz, 2H), 1.25 (t, *J* = 7.1 Hz, 3H). ^{13}C NMR (151 MHz, $CDCl_3$) δ 152.7, 141. 9, 115.0, 115.0, 55.9, 40.2, 14.8. Spectroscopic data are in agreement with those reported earlier.[4]

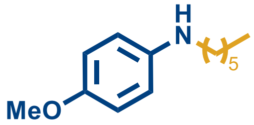

***N*-hexyl-4-methoxyaniline (3ac)** Yellow oil, Yield: 743 mg, 90%. ^{1}H NMR (600 MHz, $CDCl_3$) δ 6.81 – 6.75 (m, 2H), 6.61 – 6.55 (m, 2H), 3.75 (s, 3H), 3.06 (t, *J* = 7.2 Hz, 2H), 1.60 (p, *J* = 7.3 Hz, 2H), 1.47 – 1.36 (m, 2H), 1.35 – 1.29 (m, 4H), 0.97 – 0.83 (m, 3H). ^{13}C NMR (151 MHz, $CDCl_3$) δ 152.1, 143.0, 115.1, 114.2, 56.0, 45.2, 31.8, 29.8, 27.0, 22.8, 14.2. Spectroscopic data are in agreement with those reported earlier.[5]

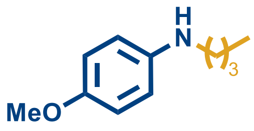

***N*-butyl-4-methoxyaniline (3ad)** Yellow oil, Yield: 602 mg, 84%. ^{1}H NMR (600 MHz, $CDCl_3$) δ 6.94 – 6.74 (m, 2H), 6.66 – 6.47 (m, 2H), 3.75 (s, 3H), 3.07 (t, *J* = 7.1 Hz, 2H), 1.59 (dtd, *J* = 8.8, 7.4, 5.9 Hz, 2H), 1.47 – 1.37 (m, 2H), 0.96 (t, *J* = 7.4 Hz, 3H). ^{13}C NMR (151 MHz, $CDCl_3$) δ 152.2, 143.0, 115.1, 114.2, 56.0, 44.9, 31.9, 20.5, 14.1. Spectroscopic data are in agreement with those reported earlier.[6]

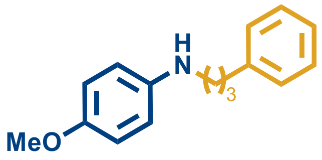

4-methoxy-*N*-(3-phenylpropyl)aniline (3af) Yellow oil, Yield: 906 mg, 94%. ^{1}H NMR (600 MHz, $CDCl_3$) δ 7.30 (dd, *J* = 8.4, 6.8 Hz, 2H), 7.24 – 7.18 (m, 3H), 6.81 – 6.74 (m, 2H), 6.59 – 6.50 (m, 2H), 3.75 (s, 3H), 3.12 (t, *J* = 7.0 Hz, 2H), 2.74 (t, *J* = 7.6 Hz, 2H), 1.95 (dq, *J* = 8.7, 7.1 Hz, 2H). ^{13}C NMR (151 MHz, $CDCl_3$) δ 152.2, 142.8, 141.9, 128.6, 128.5, 126.1, 115.1, 114.3, 56.0, 44.6, 33.6, 31.3. Spectroscopic data are in agreement with those reported earlier.[7]

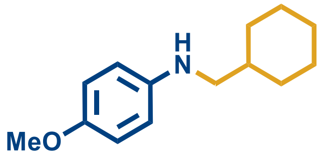

***N*-(cyclohexylmethyl)-4-methoxyaniline (3ag)** Colourless oil, Yield: 858 mg, 98%. ^{1}H NMR (600 MHz, $CDCl_3$) δ 6.83 – 6.74 (m, 2H), 6.60 – 6.54 (m, 2H), 3.75 (s, 3H), 2.91 (d, *J* = 6.7 Hz, 2H), 1.90 – 1.78 (m, 2H), 1.77 – 1.71 (m, 2H), 1.68 (dddd, *J* = 12.1, 4.7, 3.1, 1.5 Hz, 1H), 1.56 (dddd, *J* = 14.5, 11.3, 6.8, 3.2 Hz, 1H), 1.31 – 1.15 (m, 3H), 0.98 (qd, *J* = 12.0, 3.4 Hz, 2H). ^{13}C NMR (151 MHz, $CDCl_3$) δ 152.0, 143.1, 115.1, 114.1, 56.0, 51.8, 37.78, 31.5, 26.8, 26.1. Spectroscopic data are in agreement with those reported earlier.[8]

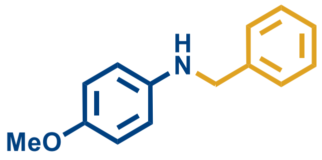

***N*-benzyl-4-methoxyaniline (3ai)** Yellow oil, Yield: 716 mg, 84%. ^{1}H NMR (600 MHz, $CDCl_3$) δ 7.43 – 7.35 (m, 4H), 7.32 – 7.28 (m, 1H), 6.83 – 6.77 (m, 2H), 6.63 (dd, *J* = 8.6, 1.5 Hz, 2H), 4.31 (s, 2H), 3.77 (s, 3H). ^{13}C NMR (151 MHz, $CDCl_3$) δ 152.3, 142.6, 139.8, 128.7, 127.6, 127.3, 115.0, 114.2, 55.9, 49.3. Spectroscopic data are in agreement with those reported earlier.[9]

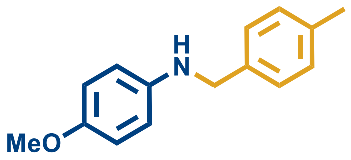

4-methoxy-*N*-(4-methylbenzyl)aniline (3aj) Yellow oil, Yield: 520 mg, 57%. ^{1}H NMR (600 MHz, $CDCl_3$) δ 7.18 (t, *J* = 3.9 Hz, 3H), 7.07 (d, *J* = 7.7 Hz, 2H), 6.73 – 6.67 (m, 2H), 6.56 – 6.51 (m, 2H), 4.16 (s, 2H), 3.67 (s, 3H), 2.27 (s, 3H), 1.50 (br, 1H). ^{13}C NMR (151 MHz, $CDCl_3$) δ 152.3, 142.7, 136.9, 136.7, 129.4, 127.7, 115.1, 114.3, 56.0, 49.2, 21.2. Spectroscopic data are in agreement with those reported earlier.[10]

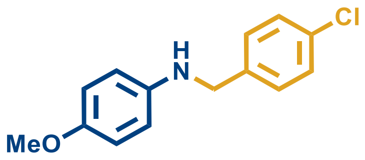

***N*-(4-chlorobenzyl)-4-methoxyaniline (3ak)** Yellow oil, Yield: 624 mg, 63%. ^{1}H NMR (600 MHz, $CDCl_3$) δ 7.30 (s, 4H), 6.81 – 6.73 (m, 2H), 6.62 – 6.53 (m, 2H), 4.26 (s, 2H), 3.74 (s, 3H). ^{13}C NMR (151 MHz, $CDCl_3$) δ 152.5, 142.2, 138.4, 132.3, 128.9, 128.9, 115.1, 114.3, 56.9, 48.7. Spectroscopic data are in agreement with those reported earlier.[11]

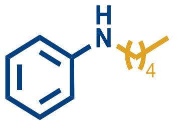

***N*-pentylaniline (3ba)** Yellow oil, Yield: 594 mg, 91%. ^{1}H NMR (600 MHz, $CDCl_3$) δ 7.20 (ddt, *J* = 9.5, 7.4, 2.2 Hz, 2H), 6.71 (tt, *J* = 7.3, 1.0 Hz, 1H), 6.63 (dt, *J* = 8.8, 1.6 Hz, 2H), 3.61 (br, 1H), 3.30 – 2.99 (m, 2H), 1.79 – 1.53 (m, 2H), 1.44 – 1.36 (m, 4H), 0.95 (t, *J* = 7.1 Hz, 3H). ^{13}C NMR (151 MHz,

$CDCl_3$) δ 148.7, 129. 3, 117.2, 112.8, 44.1, 29.5, 29.4, 22.6, 14.2. Spectroscopic data are in agreement with those reported earlier.[12]

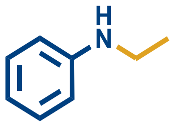

***N*-ethylaniline (3bb)** Yellow oil, Yield: 465 mg, 96%. ^{1}H NMR (600 MHz, $CDCl_3$) δ 7.22 – 7.14 (m, 2H), 6.71 (tt, *J* = 7.3, 1.1 Hz, 1H), 6.65 – 6.57 (m, 2H), 3.54 (br, 1H), 3.17 (q, *J* = 7.1 Hz, 2H), 1.27 (t, *J* = 7.1 Hz, 3H). ^{13}C NMR (151 MHz, $CDCl_3$) δ 148.6, 129.3, 117.3, 112.9, 38.6, 15.0. Spectroscopic data are in agreement with those reported earlier.[13]

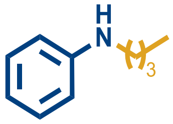

***N*-butylaniline (3bd)** Yellow oil, Yield: 527 mg, 88%. ^{1}H NMR (600 MHz, $CDCl_3$) δ 7.21 – 7.16 (m, 2H), 6.70 (tt, *J* = 7.3, 1.0 Hz, 1H), 6.65 – 6.57 (m, 2H), 3.60 (br, 1H), 3.13 (t, *J* = 7.1 Hz, 2H), 1.62 (tt, *J* = 8.7, 7.0 Hz, 2H), 1.50 – 1.38 (m, 2H), 0.98 (t, *J* = 7.4 Hz, 3H). ^{13}C NMR (151 MHz, $CDCl_3$) δ 148.7, 129.3, 117.2, 112.8, 43.8, 31.8, 20.4, 14.0. Spectroscopic data are in agreement with those reported earlier.[14]

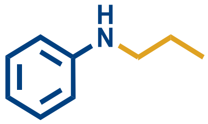

***N*-propylaniline (3be)** Yellow oil, Yield: 493 mg, 91%. ^{1}H NMR (600 MHz, $CDCl_3$) δ 7.23 – 7.16 (m, 2H), 6.71 (tt, *J* = 7.4, 1.1 Hz, 1H), 6.65 – 6.60 (m, 2H), 3.63 (br, 1H), 3.10 (t, *J* = 7.1 Hz, 2H), 1.66 (h, *J* = 7.3 Hz, 2H), 1.02 (t, *J* = 7.4 Hz, 3H). ^{13}C NMR (151 MHz, $CDCl_3$) δ 148.6, 129.3, 117.2, 112.8, 45.9, 22.9, 11.8. Spectroscopic data are in agreement with those reported earlier.[15]

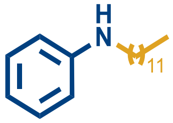

***N*-dodecylaniline (3bh)** Yellow oil, Yield: 847 mg, 81%. ^{1}H NMR (600 MHz, $CDCl_3$) δ 7.19 – 7.14 (m, 2H), 6.69 (t, *J* = 7.3 Hz, 1H), 6.63 – 6.58 (m, 2H), 3.10 (t, *J* = 7.1 Hz, 2H), 1.62 (p, *J* = 7.2 Hz,

2H), 1.44 – 1.36 (m, 2H), 1.28 (d, J = 9.2 Hz, 16H), 0.89 (t, J = 6.9 Hz, 3H). ^{13}C NMR (151 MHz, $CDCl_3$) δ 148.7, 129.4, 117.2, 112.9, 44.2, 32.1, 29.8, 29. 8, 29.7, 29.7, 29.6, 29.5, 27.3, 22.8, 14.3. Spectroscopic data are in agreement with those reported earlier.[15]

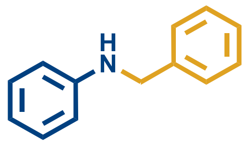

***N*-benzylaniline (3bi)** Yellow oil, Yield: 667 mg, 91%. ^{1}H NMR (600 MHz, $CDCl_3$) δ 7.43 – 7.35 (m, 4H), 7.31 (t, J = 7.2 Hz, 1H), 7.21 (t, J = 7.7 Hz, 2H), 6.76 (t, J = 7.4 Hz, 1H), 6.67 (d, J = 8.0 Hz, 2H), 4.36 (s, 2H), 4.05 (br, 1H). ^{13}C NMR (151 MHz, $CDCl_3$) δ 148.3, 139.6, 129.4, 128.8, 127.6, 127.3, 117.7, 113.0, 48.4. Spectroscopic data are in agreement with those reported earlier.[16]

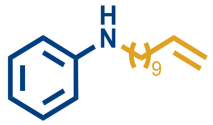

***N*-(undec-10-en-1-yl)aniline (3bl)** Yellow oil, Yield: 676 mg, 69%. ^{1}H NMR (600 MHz, $CDCl_3$) δ 7.21 – 7.13 (m, 2H), 6.69 (td, J = 7.3, 1.4 Hz, 1H), 6.61 (dt, J = 7.4, 1.2 Hz, 2H), 5.83 (ddt, J = 16.9, 10.2, 6.7 Hz, 1H), 5.01 (dq, J = 17.1, 1.7 Hz, 1H), 4.94 (ddt, J = 10.2, 2.2, 1.2 Hz, 1H), 3.63 (br, 1H), 3.11 (t, J = 7.1 Hz, 2H), 2.11 – 1.99 (m, 2H), 1.62 (p, J = 7.2 Hz, 2H), 1.40 (h, J = 6.8 Hz, 4H), 1.35 – 1.30 (m, 8H). ^{13}C NMR (151 MHz, $CDCl_3$) δ 148.6, 139.3, 129.3, 117.3, 114.3, 112.9, 44.2, 44.2, 33.9, 29.7, 29.7, 29.6, 29.2, 29.1, 27.3. Spectroscopic data are in agreement with those reported earlier.[17]

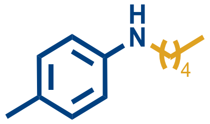

4-methyl-*N*-pentylaniline (3ca) Yellow oil, Yield: 658 mg, 93%. ^{1}H NMR (600 MHz, $CDCl_3$) δ 7.11 – 6.97 (m, 2H), 6.63 – 6.48 (m, 2H), 3.10 (t, J = 7.2 Hz, 2H), 2.26 (s, 3H), 1.71 – 1.56 (m, 2H), 1.39 (h, J = 2.9 Hz, 4H), 1.03 – 0.85 (m, 3H). ^{13}C NMR (151 MHz, $CDCl_3$) δ 146.3, 129.8, 126.5, 113.1, 44.6, 29.5, 29.4, 22.6, 20.5, 14.2. Spectroscopic data are in agreement with those reported earlier.[18]

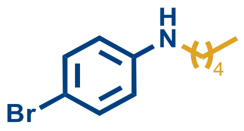

4-bromo-*N*-pentylaniline (3da) Yellow oil, Yield: 920 mg, 95%. ^{1}H NMR (600 MHz, $CDCl_3$) δ 7.33 – 7.24 (m, 2H), 6.59 – 6.43 (m, 2H), 3.65 (br, 1H), 3.09 (t, J = 7.2 Hz, 2H), 1.64 (p, J = 7.4 Hz, 2H), 1.41 (dt, J = 7.3, 3.8 Hz, 4H), 1.02 – 0.90 (m, 3H). ^{13}C NMR (151 MHz, $CDCl_3$) δ 147.6, 131.9, 114.3, 108.5, 44.0, 29.4, 29.2, 22.6, 14.1. Spectroscopic data are in agreement with those reported earlier.[18]

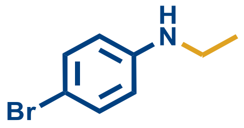

4-bromo-*N*-ethylaniline (3db) Yellow oil, Yield: 801 mg, 95%. ^{1}H NMR (600 MHz, $CDCl_3$) δ 7.26 – 7.19 (m, 2H), 6.53 – 6.43 (m, 2H), 3.57 (br, 1H), 3.12 (q, J = 7.0 Hz, 2H), 1.25 (td, J = 7.1, 1.2 Hz, 3H). ^{13}C NMR (151 MHz, $CDCl_3$) δ 147.5, 132.0, 114.4, 108.81, 38.61, 14.84. Spectroscopic data are in agreement with those reported earlier.[13b]

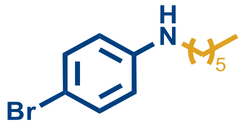

4-bromo-*N*-hexylaniline (3dc) Yellow oil, Yield: 942 mg, 92%. ^{1}H NMR (600 MHz, $CDCl_3$) δ 7.27 – 7.11 (m, 2H), 6.58 – 6.29 (m, 2H), 3.65 (br, 1H), 3.06 (t, J = 7.2 Hz, 2H), 1.60 (p, J = 7.3 Hz, 2H), 1.39 (dq, J = 8.9, 6.8 Hz, 2H), 1.35 – 1.30 (m, 4H), 0.94 – 0.88 (m, 3H). ^{13}C NMR (151 MHz, $CDCl_3$) δ 147.6, 132.0, 114.4, 108.7, 44.2, 31.7, 29.5, 26.9, 22.7, 14.2. Spectroscopic data are in agreement with those reported earlier.[5]

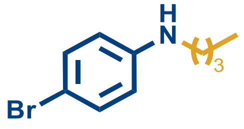

4-bromo-*N*-butylaniline (3dd) Yellow oil, Yield: 828 mg, 91%. ^{1}H NMR (600 MHz, $CDCl_3$) δ 7.33 – 7.12 (m, 2H), 6.51 – 6.39 (m, 2H), 3.62 (br, 1H), 3.07 (t, J = 7.1 Hz, 2H), 1.72 – 1.53 (m, 2H), 1.43 (dt, J = 14.9, 7.4 Hz, 2H), 0.96 (t, J = 7.4 Hz, 3H). ^{13}C NMR (151 MHz, $CDCl_3$) δ 147.6, 131.0, 114.3, 108.6, 43.8, 31.6, 20.4, 14.0. Spectroscopic data are in agreement with those reported earlier.[19]

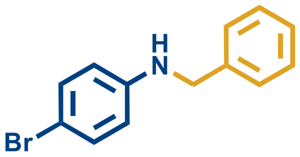

***N*-benzyl-4-bromoaniline (3di)** Yellow oil, Yield: 933 mg, 89%. ^{1}H NMR (600 MHz, $CDCl_3$) δ 7.35 (d, *J* = 4.4 Hz, 4H), 7.31 – 7.27 (m, 1H), 7.26 – 7.22 (m, 2H), 6.55 – 6.48 (m, 2H), 4.31 (s, 2H), 4.10 (br, 1H). ^{13}C NMR (151 MHz, $CDCl_3$) δ 147.2, 139.0, 132.1, 128.8, 127.5, 127.5, 114.6, 109.3, 48.4. Spectroscopic data are in agreement with those reported earlier.[20]

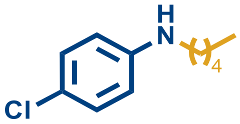

4-chloro-*N*-pentylaniline (3ea) Yellow oil, Yield: 688 mg, 87%. ^{1}H NMR (600 MHz, $CDCl_3$) δ 7.17 – 7.00 (m, 2H), 6.71 – 6.36 (m, 2H), 3.65 (br, 1H), 3.07 (t, *J* = 7.2 Hz, 2H), 1.80 – 1.53 (m, 2H), 1.42 – 1.30 (m, 4H), 1.05 – 0.83 (m, 3H). ^{13}C NMR (151 MHz, $CDCl_3$) δ 147.2, 129.1, 121.7, 113.9, 44.3, 29.4, 29.3, 22.6, 14.2. Spectroscopic data are in agreement with those reported earlier.[21]

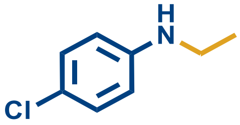

4-chloro-*N*-ethylaniline (3eb) Yellow oil, Yield: 597 mg, 96%. ^{1}H NMR (600 MHz, $CDCl_3$) δ 7.18 – 7.01 (m, 2H), 6.60 – 6.41 (m, 2H), 3.55 (br, 1H), 3.12 (q, *J* = 7.1 Hz, 2H), 1.25 (t, *J* = 7.1 Hz, 3H). ^{13}C NMR (151 MHz, $CDCl_3$) δ 147.1, 129.1, 121.8, 113.9, 38.7, 14.9. Spectroscopic data are in agreement with those reported earlier.[22]

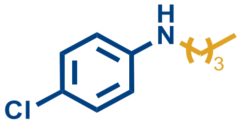

***N*-butyl-4-chloroaniline (3ed)** Yellow oil, Yield: 660 mg, 90%. ^{1}H NMR (600 MHz, $CDCl_3$) δ 7.17 – 7.05 (m, 2H), 6.56 – 6.43 (m, 2H), 3.60 (br, 1H), 3.08 (t, *J* = 7.1 Hz, 2H), 1.69 – 1.54 (m, 2H), 1.42 (dt, *J* = 14.6, 7.4 Hz, 2H), 0.97 (t, *J* = 7.4 Hz, 3H). ^{13}C NMR (151 MHz, $CDCl_3$) δ 147.2, 129.1, 121.7, 113.8, 43.9, 31.7, 20.4, 14.0. Spectroscopic data are in agreement with those reported earlier.[23]

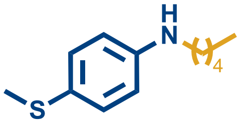

4-(methylthio)-*N*-pentylaniline (3fa) Yellow oil, Yield: 326 mg, 39%. 1H NMR (600 MHz, CDCl3) δ 7.24 – 7.20 (m, 2H), 6.67 – 6.46 (m, 2H), 3.70 (br, 1H), 3.09 (t, *J* = 7.2 Hz, 2H), 2.41 (s, 3H), 1.62 (ddt, *J* = 14.4, 10.2, 5.8 Hz, 2H), 1.37 (h, *J* = 4.0 Hz, 4H), 1.01 – 0.87 (m, 3H). ^{13}C NMR (151 MHz, CDCl3) δ 147.5, 131.8, 124.0, 113.4, 44.1, 29.4, 29.3, 22.6, 19.4, 14.1. Spectroscopic data are in agreement with those reported earlier.[2]

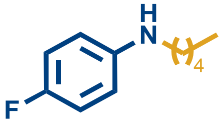

4-fluoro-*N*-pentylaniline (3ga) Yellow oil, Yield: 340 mg, 51%. ^{1}H NMR (600 MHz, $CDCl_3$) δ 7.09 – 6.76 (m, 2H), 6.63 – 6.38 (m, 2H), 3.06 (t, *J* = 7.1 Hz, 2H), 1.62 (tt, *J* = 8.8, 6.1 Hz, 2H), 1.45 – 1.32 (m, 4H), 1.02 – 0.83 (m, 3H). ^{13}C NMR (151 MHz, $CDCl_3$) δ 155.8 (d, *J* = 234.4 Hz), 145.0 (d, *J* = 1.6 Hz), 115.7 (d, *J* = 22.2 Hz), 113.6 (d, *J* = 7.5 Hz), 44.8, 29.5, 29.4, 22.6, 14.2. Spectroscopic data are in agreement with those reported earlier.[2]

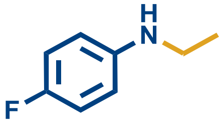

***N*-ethyl-4-fluoroaniline (3gb)** Yellow oil, Yield: 517 mg, 93%. ^{1}H NMR (600 MHz, $CDCl_3$) δ 7.07 – 6.75 (m, 2H), 6.54 (ddd, *J* = 9.0, 4.4, 0.9 Hz, 2H), 3.12 (q, *J* = 7.1 Hz, 2H), 1.25 (t, *J* = 7.1 Hz, 3H). ^{13}C NMR (151 MHz, $CDCl_3$) δ 155.9 (d, *J* = 234.6 Hz), 144.9, 115.7 (d, *J* = 22.4 Hz), 113.7 (dd, *J* = 7.3, 3.5 Hz), 39.3, 15.0. Spectroscopic data are in agreement with those reported earlier.[24]

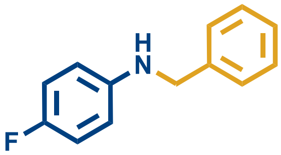

***N*-benzyl-4-fluoroaniline (3gi)** Yellow oil, Yield: 426 mg, 53%. ^{1}H NMR (600 MHz, $CDCl_3$) δ 7.42 – 7.33 (m, 4H), 7.31 – 7.27 (m, 1H), 6.95 – 6.81 (m, 2H), 6.62 – 6.53 (m, 2H), 4.30 (s, 2H). ^{13}C NMR

(151 MHz, $CDCl_3$) δ 156.9, 155.39, 144.5, 139.3, 128.2 (d, J = 174.7 Hz), 127.5, 115.8 (d, J = 22.2 Hz), 113.9 (d, J = 7.3 Hz), 49.1. Spectroscopic data are in agreement with those reported earlier.[12, 25]

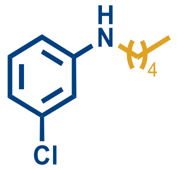

3-Chloro-*N*-pentylaniline (3ha) Yellow oil, Yield: 703 mg, 89%. ^{1}H NMR (600 MHz, $CDCl_3$) δ 7.06 (t, J = 8.0 Hz, 1H), 6.65 (dd, J = 7.9, 2.0 Hz, 1H), 6.57 (t, J = 2.1 Hz, 1H), 6.46 (dd, J = 8.2, 2.3 Hz, 1H), 3.69 (br, 1H), 3.08 (t, J = 7.2 Hz, 2H), 1.62 (dq, J = 9.6, 7.1 Hz, 2H), 1.38 (tt, J = 6.2, 2.9 Hz, 4H), 0.94 (td, J = 5.8, 2.3 Hz, 3H). ^{13}C NMR (151 MHz, $CDCl_3$) δ 149.8, 135.2, 130.3, 117.0, 112.3, 111.1, 43.9, 29.4, 29.2, 22.6, 14.1. Spectroscopic data are in agreement with those reported earlier.[2]

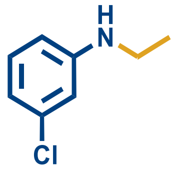

3-Chloro-*N*-ethylaniline (3hb) Yellow oil, Yield: 529 mg, 85%. ^{1}H NMR (600 MHz, $CDCl_3$) δ 7.07 (t, J = 8.0 Hz, 1H), 6.66 (ddd, J = 7.8, 2.0, 0.9 Hz, 1H), 6.57 (t, J = 2.2 Hz, 1H), 6.46 (ddd, J = 8.2, 2.3, 0.9 Hz, 1H), 3.64 (br, 1H), 3.14 (q, J = 7.2 Hz, 2H), 1.25 (t, J = 7.2 Hz, 3H). ^{13}C NMR (151 MHz, $CDCl_3$) δ 149.6, 135.1, 130.3, 117.1, 112.3, 111.2, 38.4, 14.8. Spectroscopic data are in agreement with those reported earlier.[22]

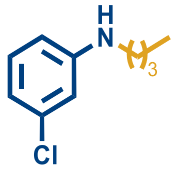

***N*-Butyl-3-chloroaniline (3hd)** Yellow oil, Yield: 639 mg, 87%. ^{1}H NMR (600 MHz, $CDCl_3$) δ 7.06 (t, J = 8.0 Hz, 1H), 6.65 (ddd, J = 7.9, 2.0, 0.9 Hz, 1H), 6.57 (t, J = 2.1 Hz, 1H), 6.46 (ddd, J = 8.2, 2.3, 0.9 Hz, 1H), 3.68 (br, 1H), 3.09 (t, J = 7.1 Hz, 2H), 1.84 – 1.52 (m, 2H), 1.43 (m, 2H), 0.97 (t, J = 7.4 Hz, 3H). ^{13}C NMR (151 MHz, $CDCl_3$) δ 149.8, 135.1, 130.2, 116.9, 112.3, 111.2, 43.6, 31.6, 20.4, 14.0. Spectroscopic data are in agreement with those reported earlier. [22]

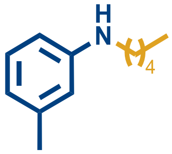

3-methyl-*N*-pentylaniline (3ia) Yellow oil, Yield: 588 mg, 83%. ^{1}H NMR (600 MHz, $CDCl_3$) δ 7.08 (t, *J* = 7.6 Hz, 1H), 6.53 (d, *J* = 7.4 Hz, 1H), 6.44 (d, *J* = 8.2 Hz, 2H), 3.55 (br, 1H), 3.11 (t, *J* = 7.2 Hz, 2H), 2.30 (s, 3H), 1.69 – 1.56 (m, 2H), 1.47 – 1.33 (m, 4H), 0.94 (td, *J* = 6.8, 2.7 Hz, 3H). ^{13}C NMR (151 MHz, $CDCl_3$) δ 148.7, 139.1, 129.2, 118.2, 113.6, 110.0, 44.1, 29.45, 29.5, 22.6, 21.8, 14.2. Spectroscopic data are in agreement with those reported earlier. [20]

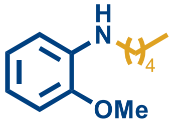

2-methoxy-*N*-pentylaniline (3ja) Yellow oil, Yield: 526 mg, 68%. ^{1}H NMR (600 MHz, $CDCl_3$) δ 6.89 (tt, *J* = 7.6, 1.3 Hz, 1H), 6.78 (dd, *J* = 8.0, 1.6 Hz, 1H), 6.67 (tt, *J* = 7.8, 1.4 Hz, 1H), 6.63 (dt, *J* = 7.8, 1.4 Hz, 1H), 4.18 (br, 1H), 3.86 (s, 3H), 3.18 – 3.08 (m, 2H), 1.68 (p, *J* = 6.8 Hz, 2H), 1.49 – 1.36 (m, 4H), 0.95 (td, *J* = 7.0, 1.4 Hz, 3H). ^{13}C NMR (151 MHz, $CDCl_3$) δ 146.9, 138.7, 121.4, 116.2, 109.8, 109.5, 55.5, 43.8, 29.6, 29.4, 22.7, 14.2. Spectroscopic data are in agreement with those reported earlier.[2, 21]

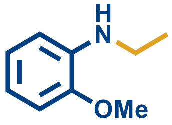

***N*-ethyl-2-methoxyaniline (3jb)** Yellow oil, Yield: 442 mg, 73%. ^{1}H NMR (600 MHz, $CDCl_3$) δ 6.94 (td, *J* = 7.6, 1.4 Hz, 1H), 6.83 (dd, *J* = 7.9, 1.4 Hz, 1H), 6.73 (td, *J* = 7.7, 1.5 Hz, 1H), 6.68 (dd, *J* = 7.8, 1.5 Hz, 1H), 4.17 (s, 1H), 3.90 (s, 3H), 3.23 (q, *J* = 7.1 Hz, 2H), 1.35 (t, *J* = 7.2 Hz, 3H). ^{13}C NMR (151 MHz, CDCl3) δ 146.8, 138.5, 121.4, 116.3, 109.9, 109.4, 55.4, 38.2, 14.9. Spectroscopic data are in agreement with those reported earlier.[4]

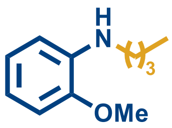

***N*-butyl-2-methoxyaniline (3jd)** Yellow oil, Yield: 445 mg, 62%. ^{1}H NMR (600 MHz, $CDCl_3$) δ 6.92 (tdd, *J* = 8.6, 2.7, 1.4 Hz, 1H), 6.81 (dq, *J* = 7.9, 1.5 Hz, 1H), 6.73 – 6.61 (m, 2H), 4.22 (s, 1H), 3.96 – 3.73 (m, 3H), 3.18 (ddd, *J* = 9.4, 5.6, 2.0 Hz, 2H), 1.69 (dtdd, *J* = 9.4, 7.3, 5.6, 3.0 Hz, 2H), 1.50 (ttdd, *J* = 9.6, 7.3, 4.0, 2.1 Hz, 2H), 1.02 (dtd, *J* = 7.4, 4.4, 2.2 Hz, 3H). ^{13}C NMR (151 MHz, CDCl3) δ 146.8, 138.6, 121.4, 116.1, 109.8, 109.4, 55.4, 43.5, 31.8, 20.5, 14.0. Spectroscopic data are in agreement with those reported earlier.[4]

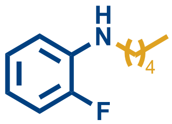

2-fluoro-*N*-pentylaniline (3ka) Yellow oil, Yield: 340 mg, 47%. ^{1}H NMR (600 MHz, $CDCl_3$) δ 7.05 – 6.91 (m, 2H), 6.70 (ddd, *J* = 9.2, 8.0, 1.6 Hz, 1H), 6.61 (tdd, *J* = 7.7, 4.9, 1.6 Hz, 1H), 3.86 (s, 1H), 3.15 (t, *J* = 7.1 Hz, 2H), 1.67 (dq, *J* = 9.3, 7.0 Hz, 2H), 1.51 – 1.28 (m, 4H), 0.95 (t, *J* = 7.0 Hz, 3H). ^{13}C NMR (151 MHz, $CDCl_3$) δ 151.7 (d, *J* = 237.8 Hz), 137.1 (d, *J* = 11.6 Hz), 124.7 (d, *J* = 3.4 Hz), 116.3 (d, *J* = 6.9 Hz), 114.4 (d, *J* = 18.5 Hz), 112.1(d, *J* = 3.6 Hz), 43.7, 29.4, 29.3, 22.6, 14.1. Spectroscopic data are in agreement with those reported earlier.[20]

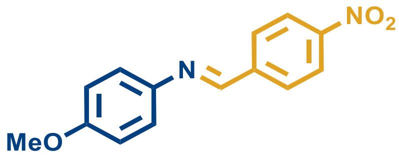

(E)-*N*-(4-methoxyphenyl)-1-(4-nitrophenyl)methanimine (3am) Orange solid, Yield: 921 mg, 90%. ^{1}H NMR (600 MHz, $CDCl_3$) δ 8.58 (s, 1H), 8.31 (d, *J* = 8.7 Hz, 2H), 8.05 (d, *J* = 8.7 Hz, 2H), 7.35 – 7.28 (m, 2H), 6.99 – 6.94 (m, 2H), 3.85 (s, 3H). Spectroscopic data are in agreement with those reported earlier.[26]

Spectral data for aliphatic amines **5aa-5ah**

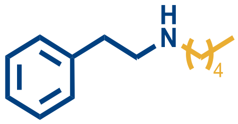

***N*-phenethylpentan-1-amine (5aa)** Colorless liquid, Yield: 170 mg, 89%. ^{1}H NMR (600 MHz, $CDCl_3$) δ 7.29 (dd, *J* = 8.3, 6.9 Hz, 2H), 7.21 (d, *J* = 7.4 Hz, 3H), 2.93 – 2.86 (m, 2H), 2.82 (t, *J* = 6.8 Hz, 2H), 2.61 (t, *J* = 7.4 Hz, 2H), 1.47 (p, *J* = 7.4 Hz, 2H), 1.34 – 1.25 (m, 4H), 0.88 (t, *J* = 7.1 Hz, 3H). ^{13}C NMR (151 MHz, $CDCl_3$) δ 140.2, 128.8, 128.6, 126.2, 51.3, 50.0, 36.5, 29.8, 29.7, 22.7, 14.1. Spectroscopic data are in agreement with those reported earlier.[27]

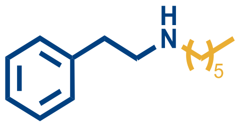

***N*-phenethylhexan-1-amine (5ac)** Colorless liquid, Yield: 186 mg, 91%. ^{1}H NMR (600 MHz, $CDCl_3$) δ 7.33 – 7.27 (m, 2H), 7.23 – 7.18 (m, 3H), 2.89 (td, *J* = 6.9, 1.2 Hz, 2H), 2.82 (dd, *J* = 8.3, 6.7 Hz, 2H), 2.64 – 2.58 (m, 2H), 1.52 – 1.43 (m, 2H), 1.28 (dddd, *J* = 11.1, 8.8, 7.0, 2.2 Hz, 6H), 0.87 (t, *J* = 6.9 Hz, 3H). ^{13}C NMR (151 MHz, $CDCl_3$) δ 140.2, 128.8, 128.6, 126.3, 51.3, 50.0, 36.4, 31.9, 30.0, 27.1, 22.72, 14.1.[28]

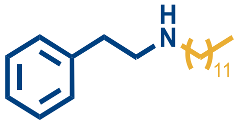

***N*-phenethyldodecan-1-amine (5ah)** Colorless liquid, Yield: 227 mg, 79%. ^{1}H NMR (600 MHz, $CDCl_3$) δ 7.31 – 7.27 (m, 2H), 7.23 – 7.18 (m, 3H), 2.89 (ddd, *J* = 7.5, 6.6, 1.3 Hz, 2H), 2.83 (dd, *J* = 8.5, 6.7 Hz, 2H), 2.65 – 2.59 (m, 2H), 1.48 (t, *J* = 7.3 Hz, 2H), 1.35 – 1.20 (m, 18H), 0.88 (t, *J* = 7.0 Hz, 3H). ^{13}C NMR (151 MHz, $CDCl_3$) δ 140.1, 128.8, 128.6, 126.2, 51.2, 49.9, 36.3, 32.0, 30.0, 29.8, 29.7 (3C), 29.6, 29.5, 27.4, 22.8, 14.2.[29]

Spectral data for *N*-heterocyclic products **6-7**.

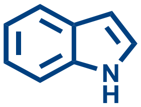

1*H*-Indole (6) white solid, Yield: 109 mg, 93%. ^{1}H NMR (600 MHz, $CDCl_3$) δ 7.89 (s, 1H), 7.57 (dq, *J* = 7.9, 0.9 Hz, 1H), 7.25 (dq, *J* = 8.2, 1.0 Hz, 1H), 7.11 (ddd, *J* = 8.2, 6.2, 1.2 Hz, 1H), 7.07 – 7.00 (m, 2H), 6.46 (ddd, *J* = 3.1, 2.0, 1.0 Hz, 1H). ^{13}C NMR (151 MHz, $CDCl_3$) δ 135.9, 127.9, 124.3, 122.1, 120.8, 119.9, 111.2, 102.7. Spectroscopic data are in agreement with those reported earlier.[2]

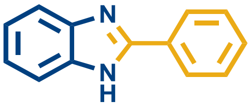

2-phenyl-1H-benzimidazole (7) white solid, Yield: 73 mg, 38%. ^{1}H NMR (600 MHz, DMSO) δ 8.18 (d, *J* = 7.6 Hz, 2H), 7.67 – 7.62 (m, 2H), 7.58 (t, *J* = 7.5 Hz, 2H), 7.54 (t, *J* = 7.3 Hz, 1H), 7.30 – 7.24 (m, 2H). ^{13}C NMR (151 MHz, DMSO) δ 150.8, 138.0, 130.5, 129.1, 128.9, 126.7, 122.8, 114.9. Spectroscopic data are in agreement with those reported earlier.[30]

NMR Spectra

^{1}H NMR (600 MHz, $CDCl_3$) spectrum of **4-methoxy-*N*-pentylaniline (3aa).**

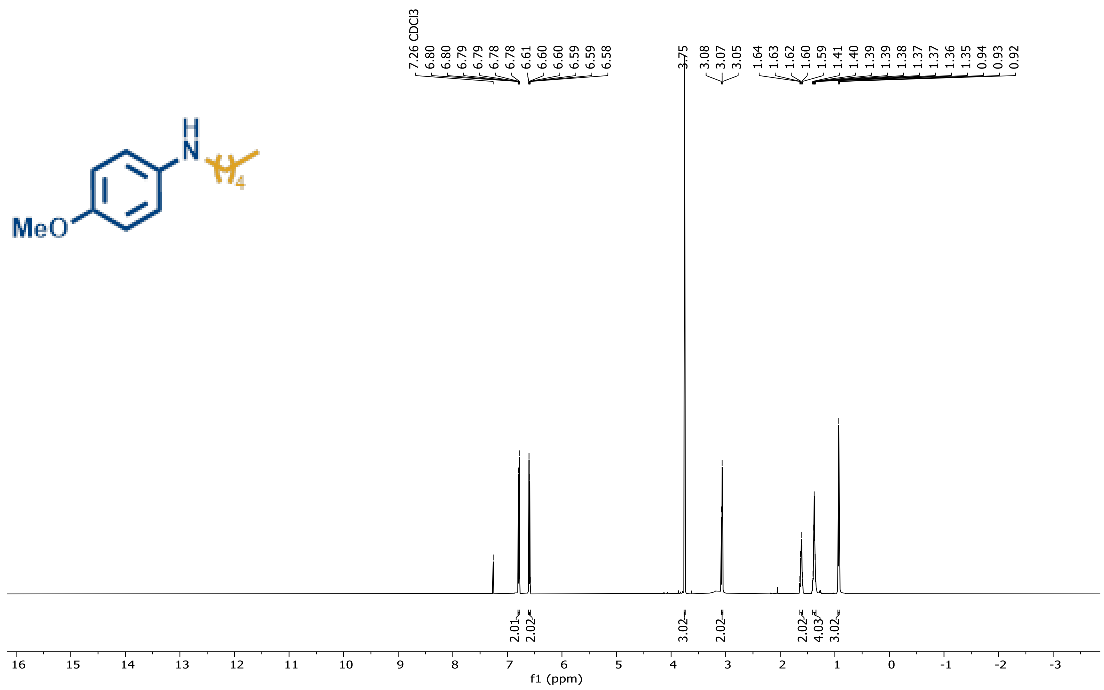

^{13}C NMR (151 MHz, $CDCl_3$) spectrum of **4-methoxy-*N*-pentylaniline (3aa).**

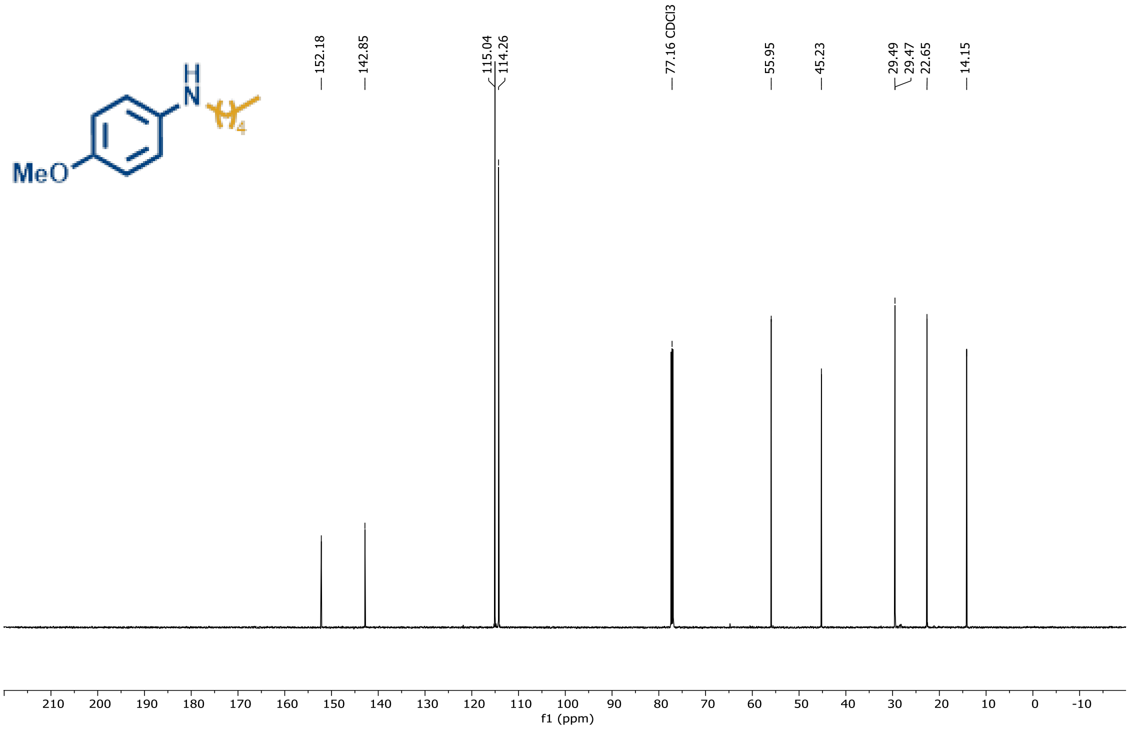

^{1}H NMR (600 MHz, $CDCl_3$) spectrum of ***N*-ethyl-4-methoxyaniline (3ab).**

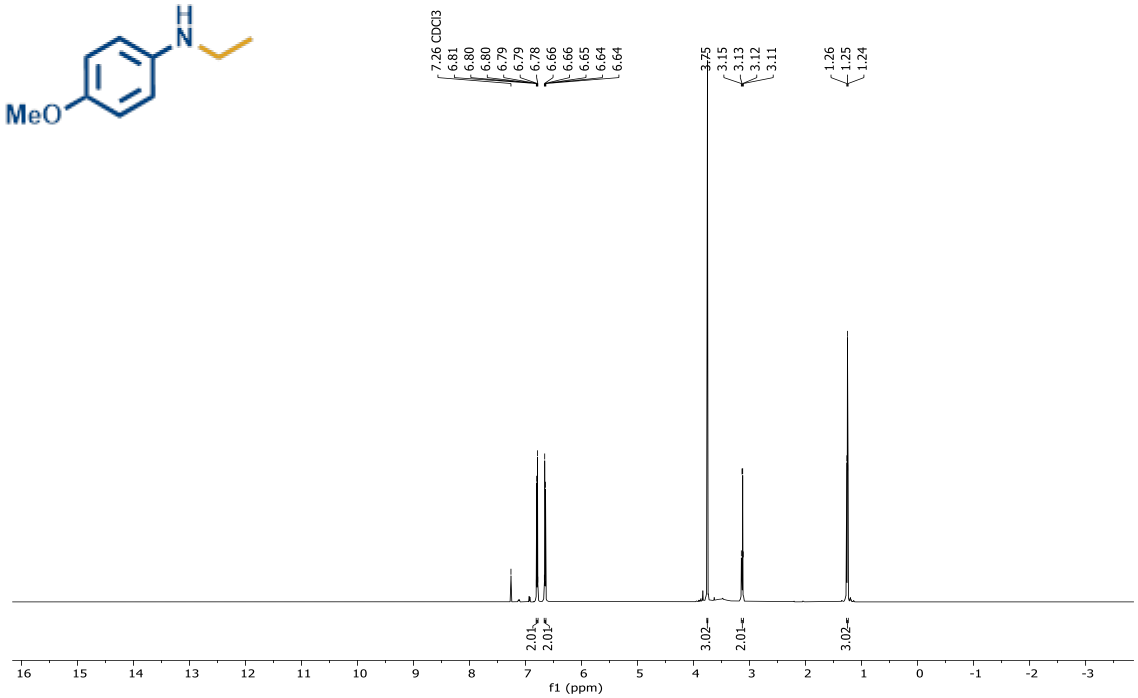

^{13}C NMR (151 MHz, $CDCl_3$) spectrum of ***N*-ethyl-4-methoxyaniline (3ab).**

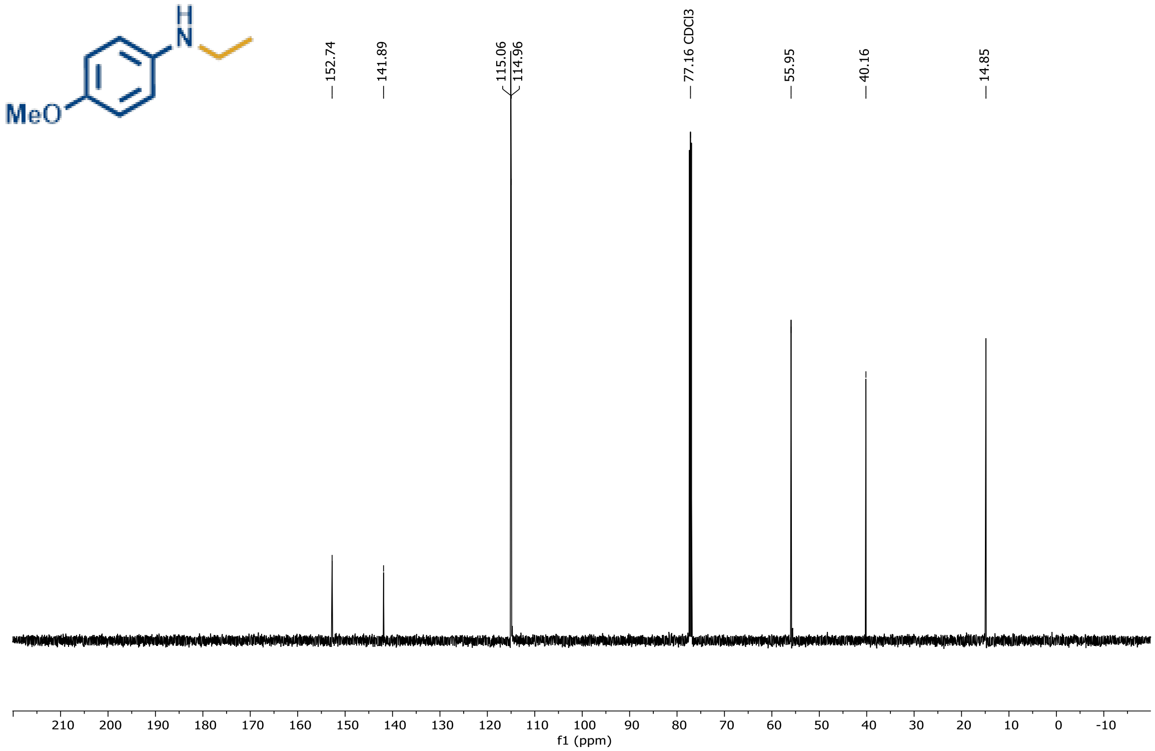

^{1}H NMR (600 MHz, $CDCl_3$) spectrum of ***N*-hexyl-4-methoxyaniline (3ac).**

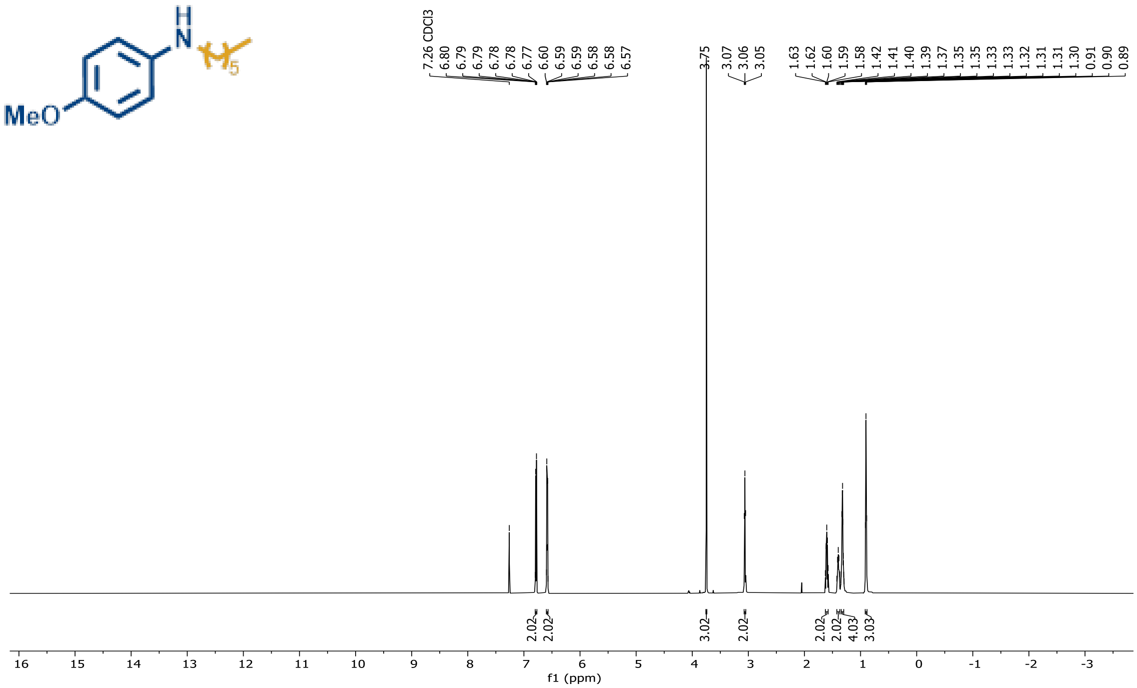

^{13}C NMR (151 MHz, $CDCl_3$) spectrum of ***N*-hexyl-4-methoxyaniline (3ac).**

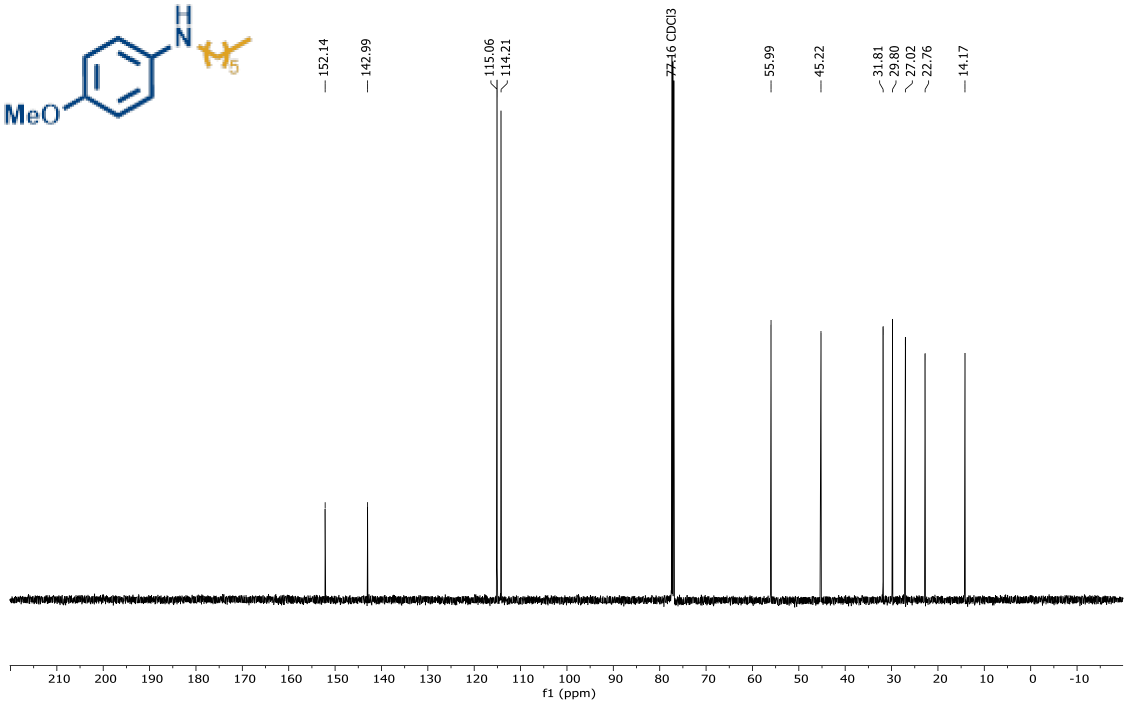

^{1}H NMR (600 MHz, $CDCl_3$) spectrum of ***N*-butyl-4-methoxyaniline (3ad).**

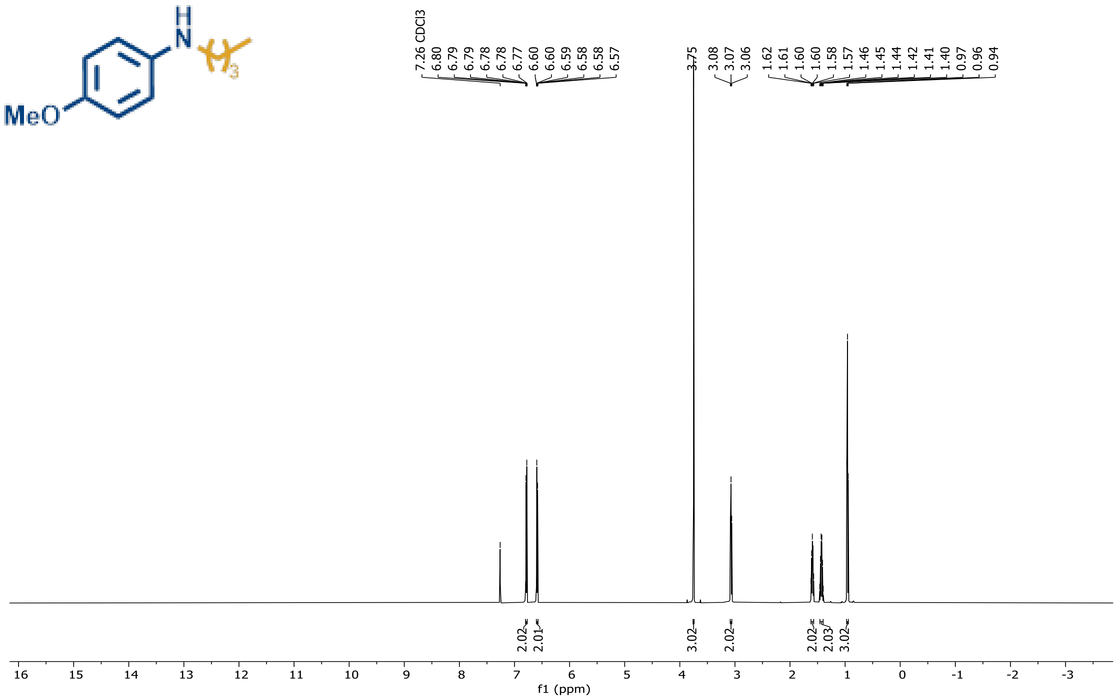

^{13}C NMR (151 MHz, $CDCl_3$) spectrum of ***N*-butyl-4-methoxyaniline (3ad).**

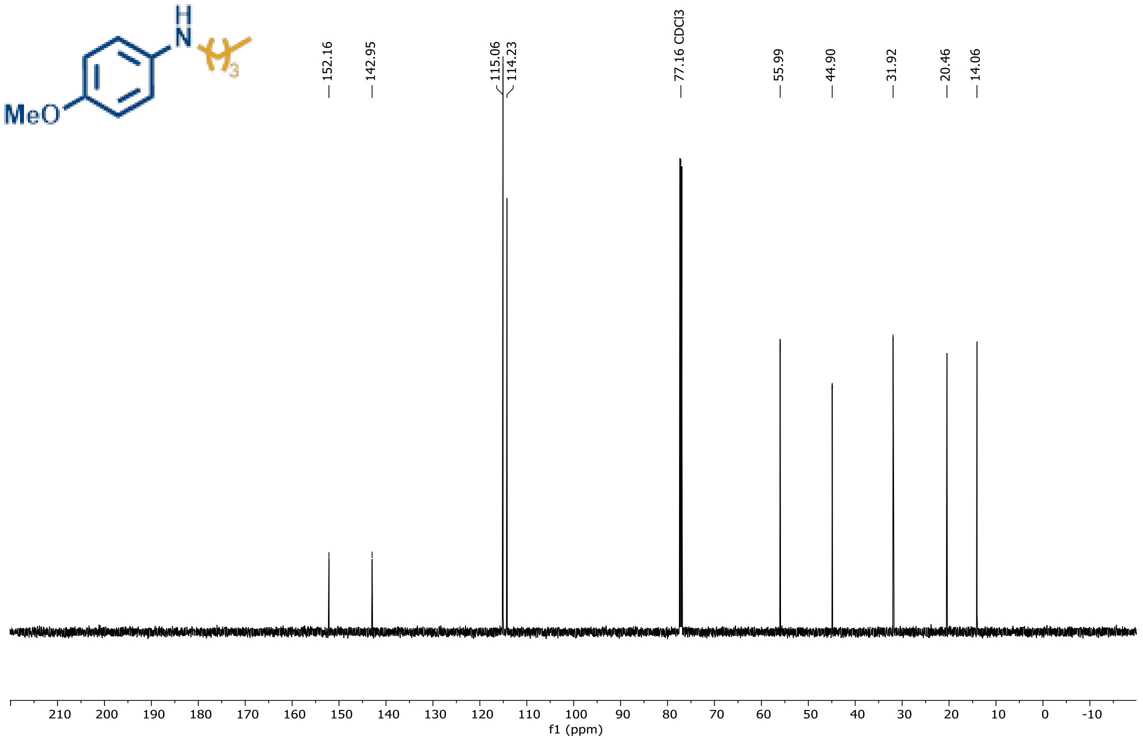

^{1}H NMR (600 MHz, $CDCl_3$) spectrum of **4-methoxy-*N*-(3-phenylpropyl)aniline (3af).**

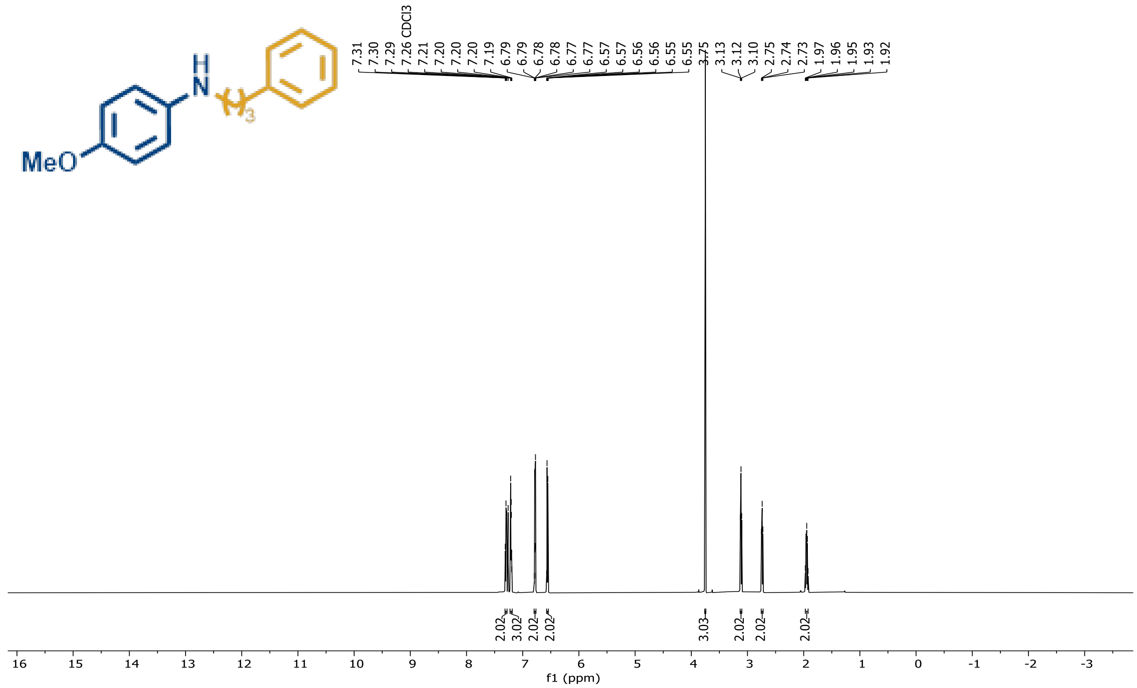

^{13}C NMR (151 MHz, $CDCl_3$) spectrum of **4-methoxy-*N*-(3-phenylpropyl)aniline (3af).**

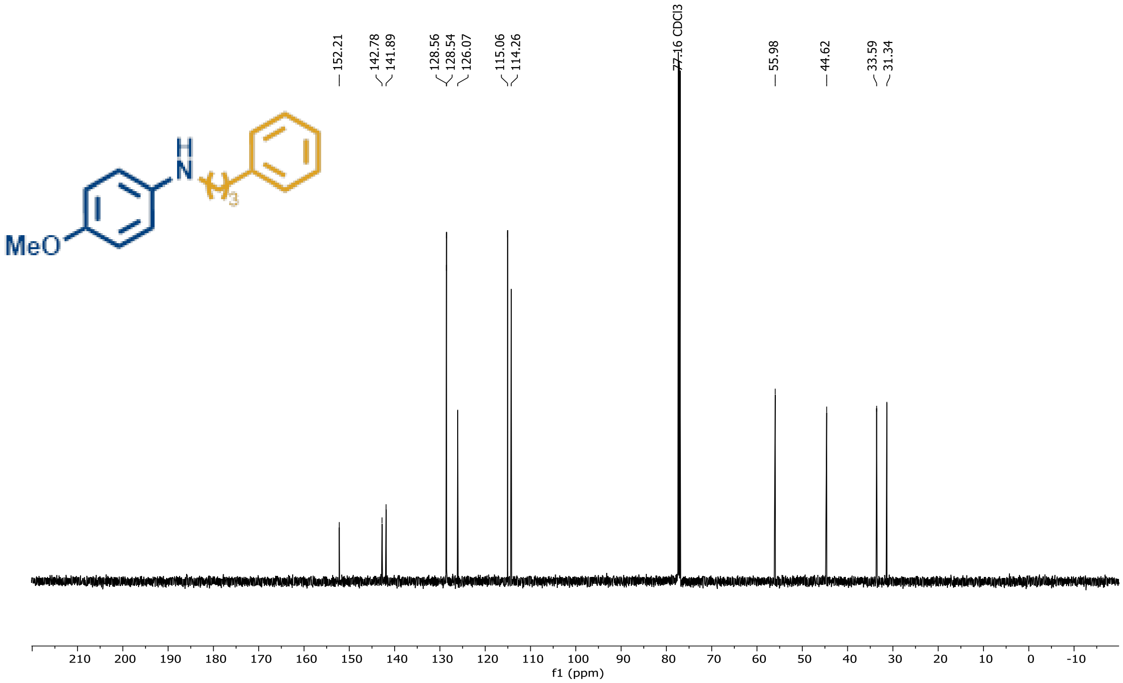

^{1}H NMR (600 MHz, $CDCl_3$) spectrum of ***N*-(cyclohexylmethyl)-4-methoxyaniline (3ag).**

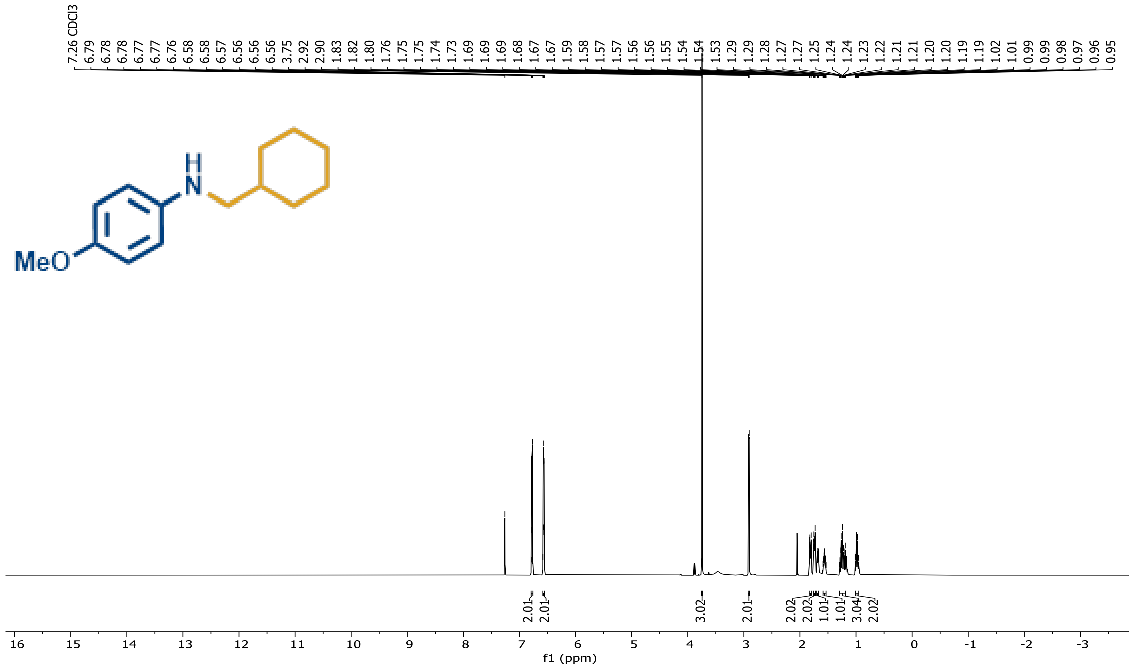

^{13}C NMR (151 MHz, $CDCl_3$) spectrum of ***N*-(cyclohexylmethyl)-4-methoxyaniline (3ag).**

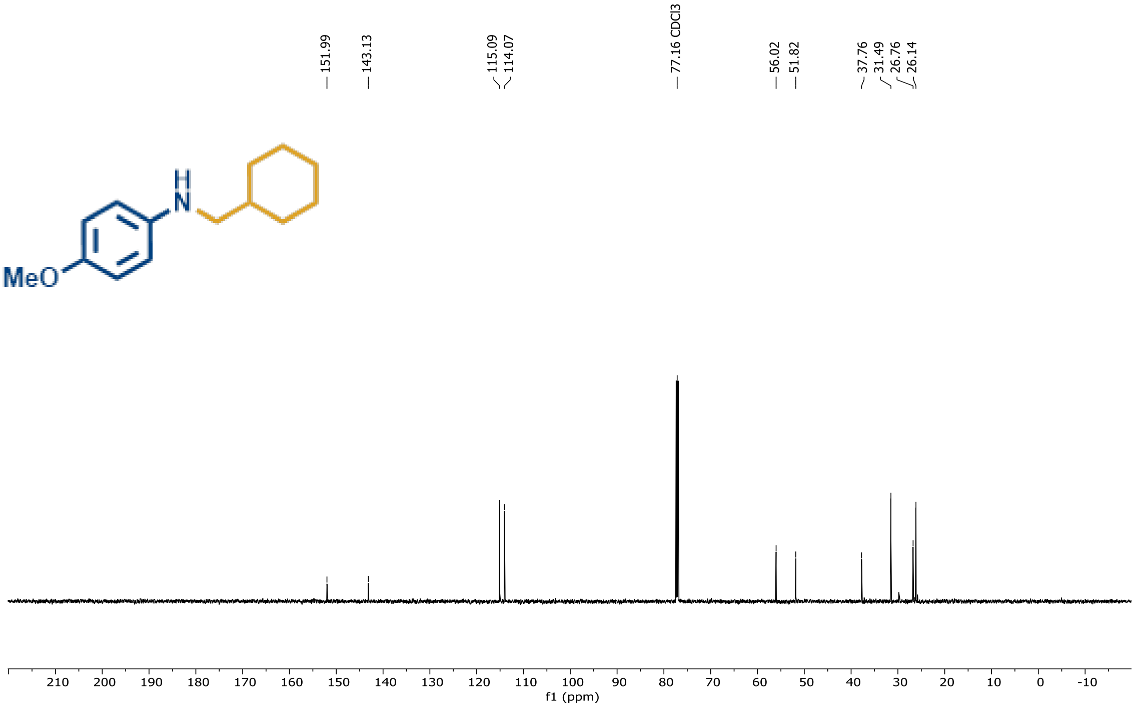

^{1}H NMR (600 MHz, $CDCl_3$) spectrum of ***N*-benzyl-4-methoxyaniline (3ai).**

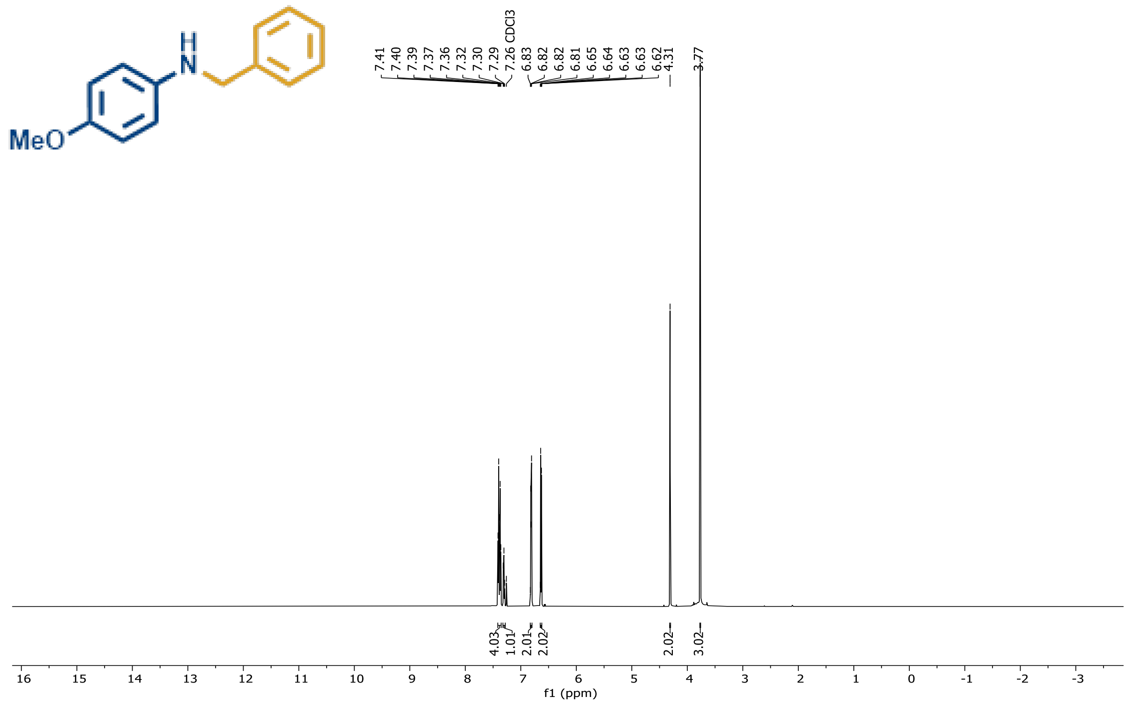

^{13}C NMR (151 MHz, $CDCl_3$) spectrum of ***N*-benzyl-4-methoxyaniline (3ai).**

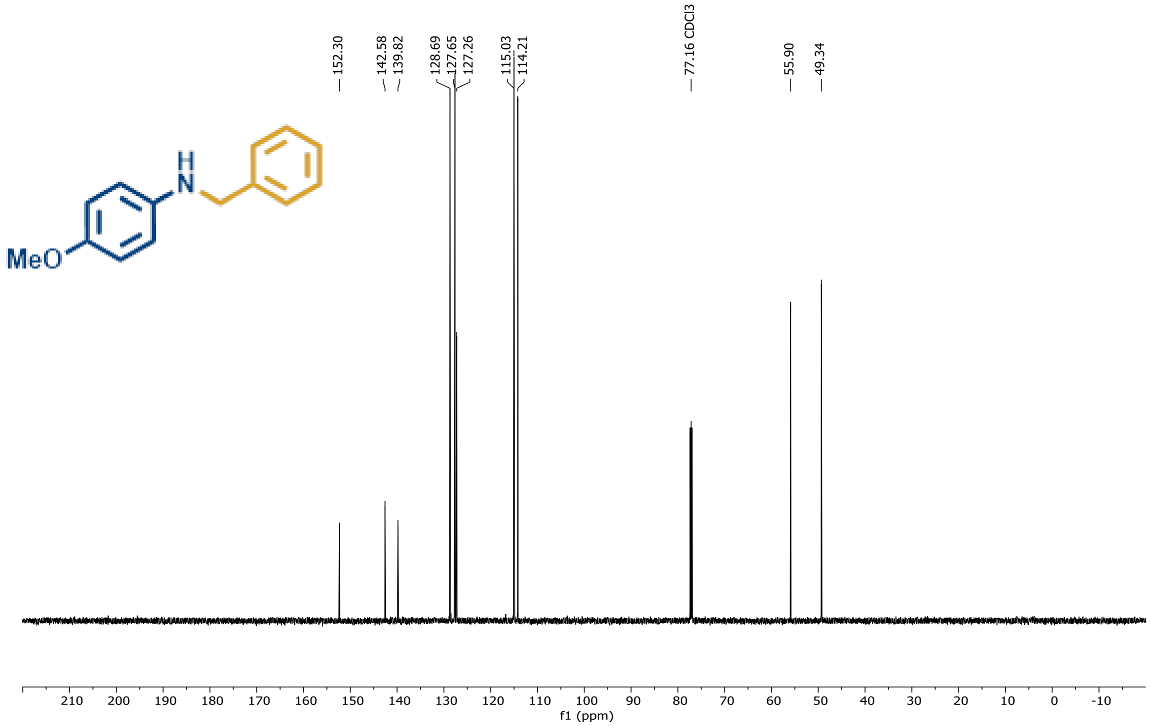

^{1}H NMR (600 MHz, $CDCl_3$) spectrum of **4-methoxy-*N*-(4-methylbenzyl)aniline (3aj).**

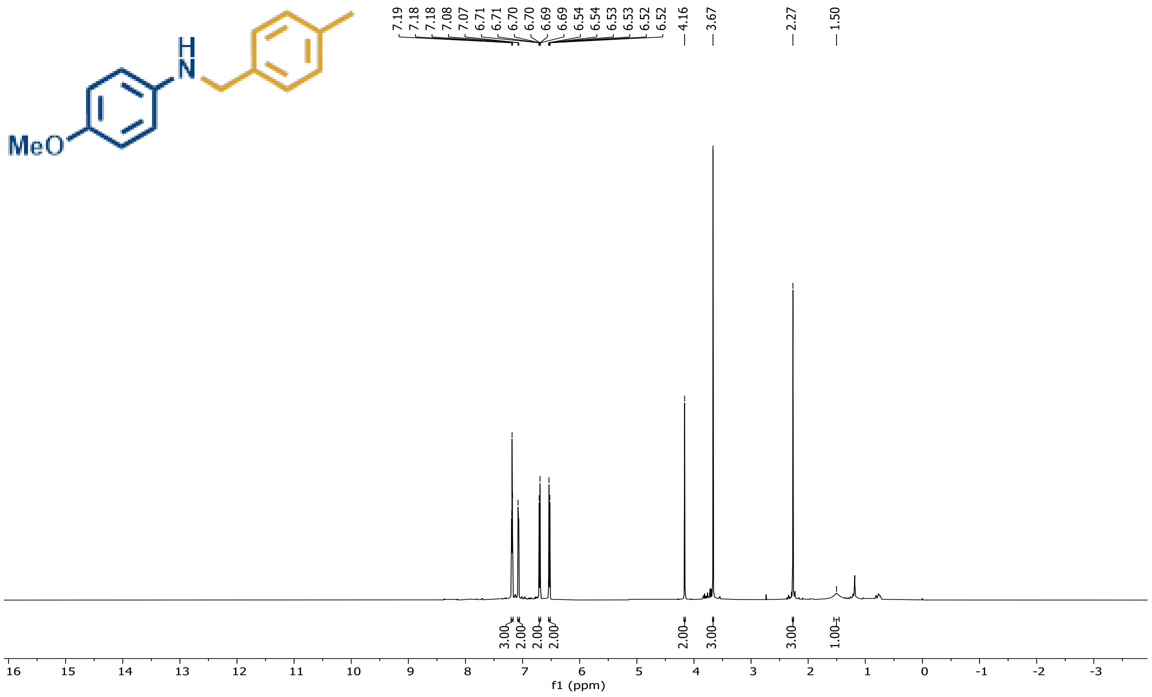

^{13}C NMR (151 MHz, $CDCl_3$) spectrum of **4-methoxy-*N*-(4-methylbenzyl)aniline (3aj).**

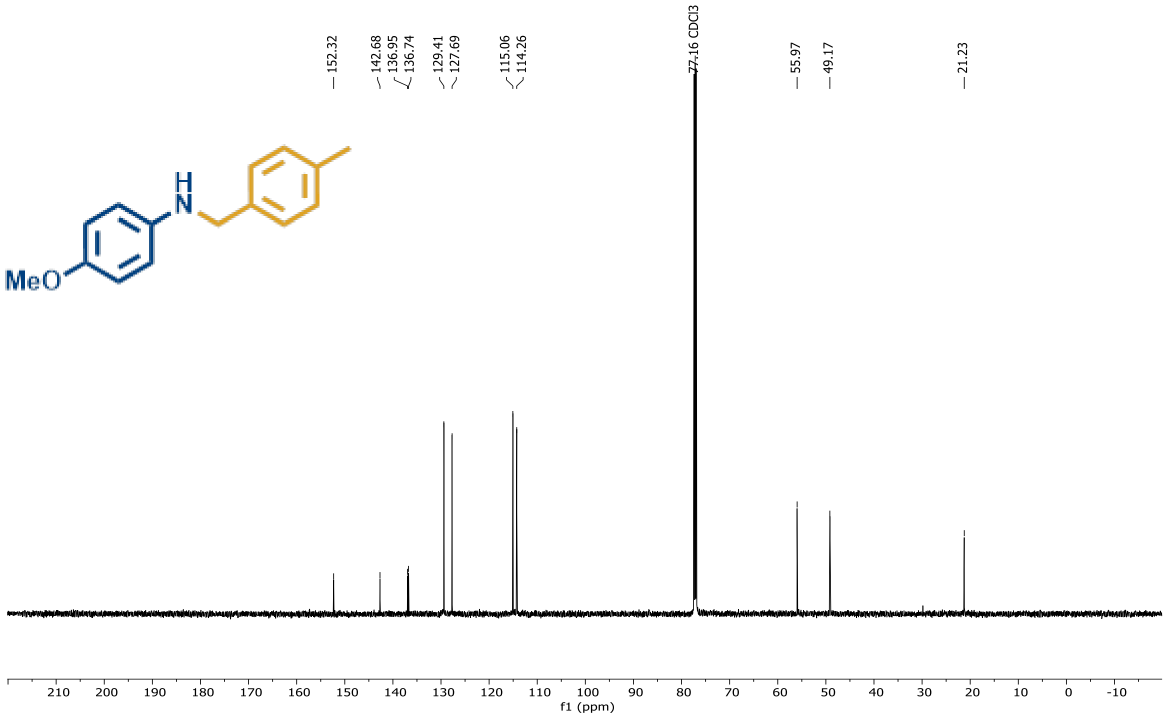

^{1}H NMR (600 MHz, $CDCl_3$) spectrum of ***N*-(4-chlorobenzyl)-4-methoxyaniline (3ak).**

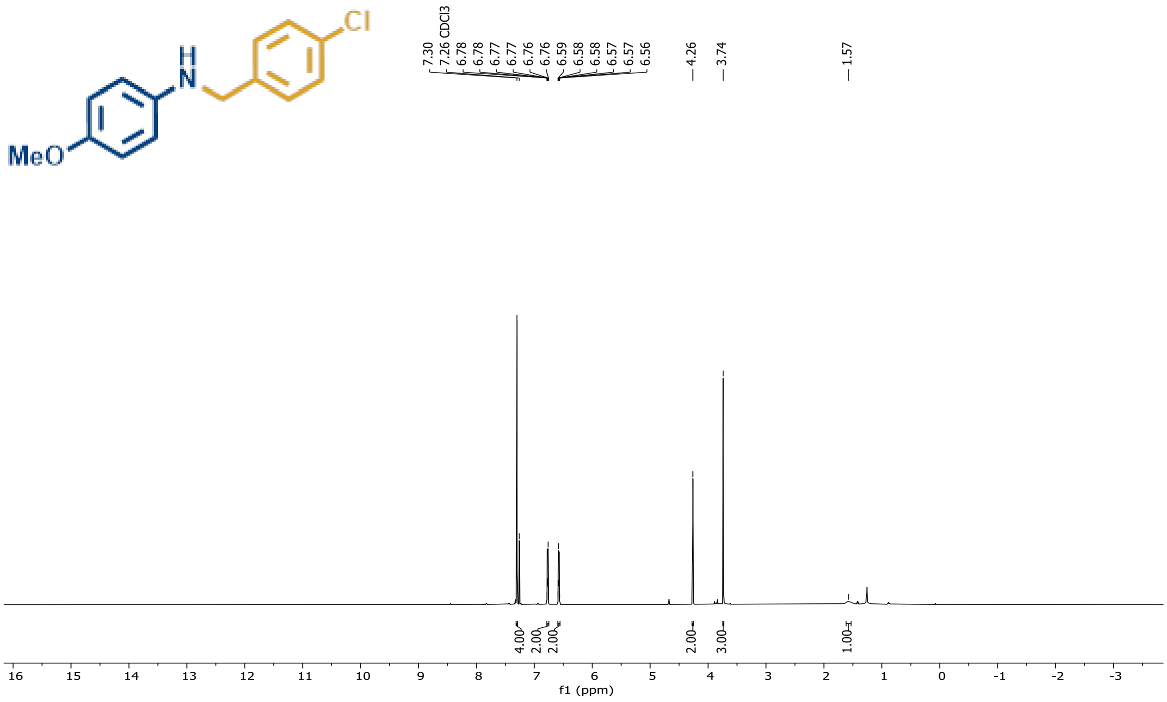

^{13}C NMR (151 MHz, $CDCl_3$) spectrum of ***N*-(4-chlorobenzyl)-4-methoxyaniline (3ak).**

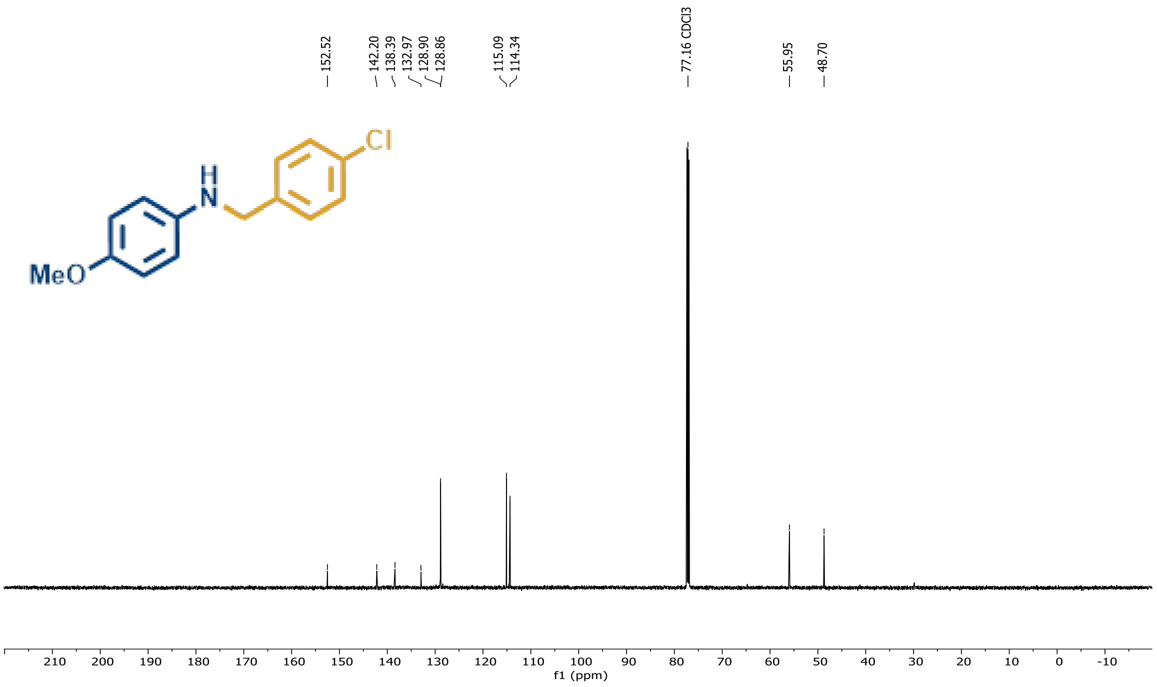

^1H NMR (600 MHz, $CDCl_3$) spectrum of ***N*-pentylaniline (3ba).**

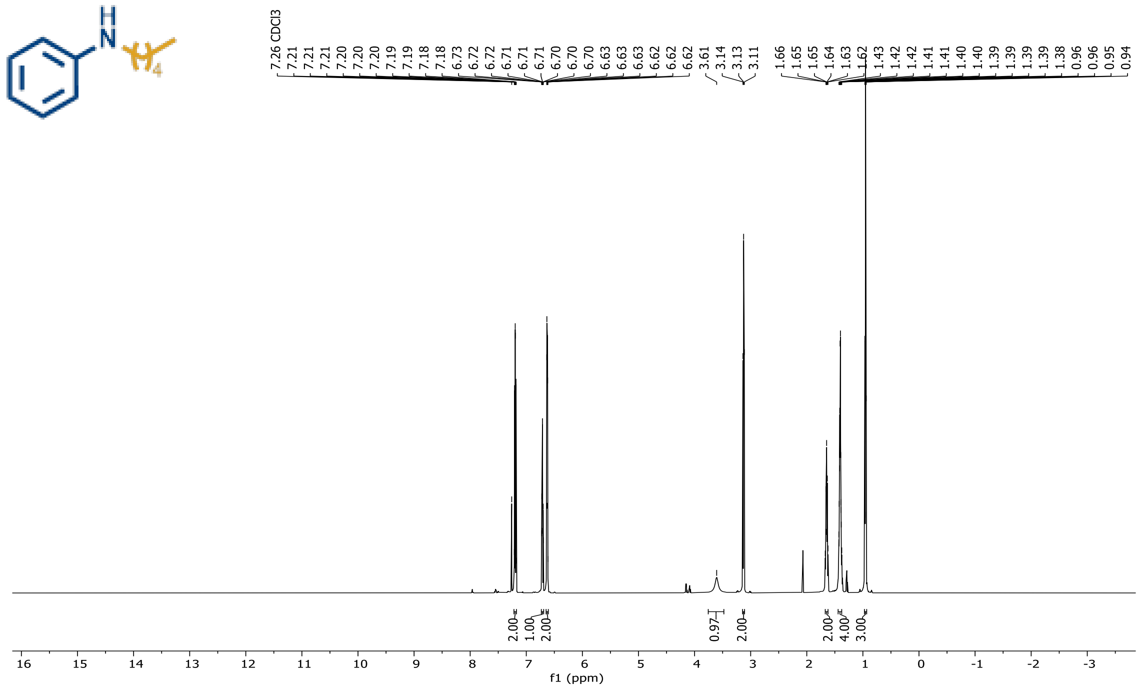

^{13}C NMR (151 MHz, $CDCl_3$) spectrum of ***N*-pentylaniline (3ba).**

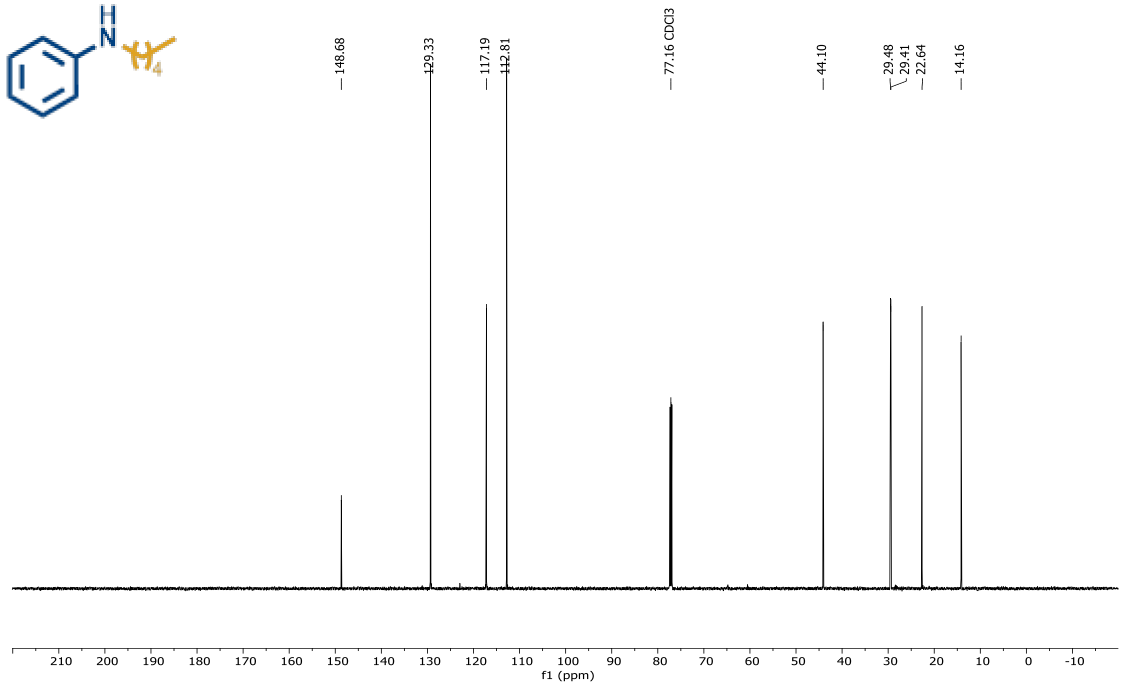

^{1}H NMR (600 MHz, $CDCl_3$) spectrum of ***N*-ethylaniline (3bb).**

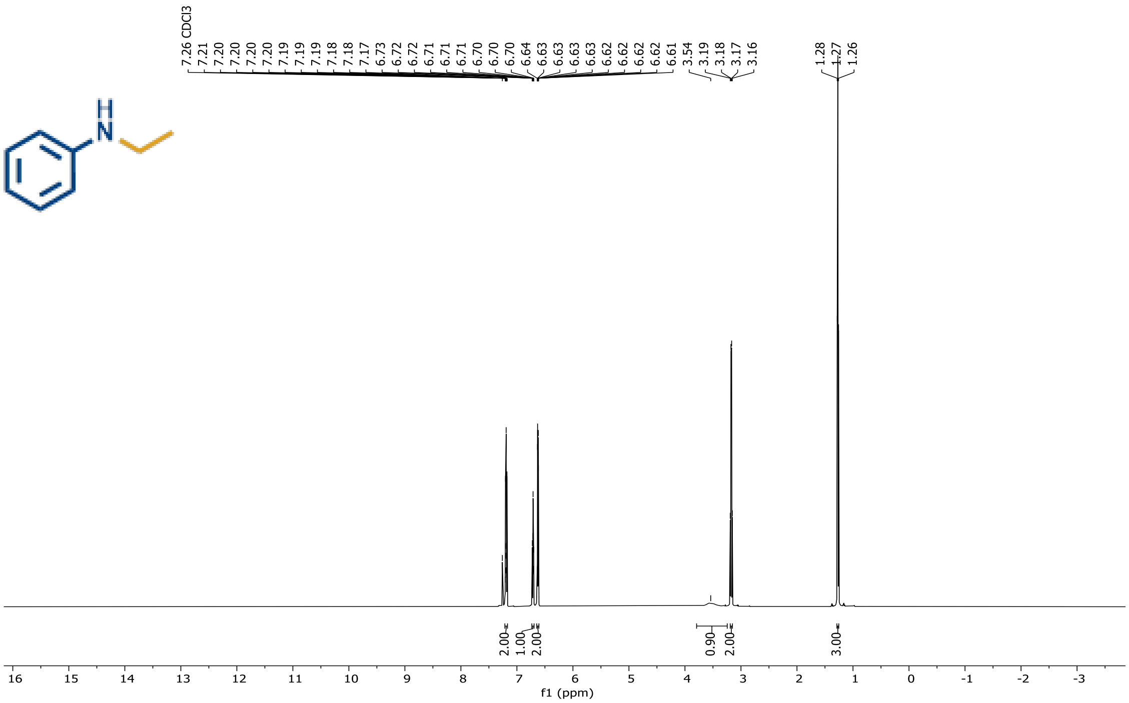

^{13}C NMR (151 MHz, $CDCl_3$) spectrum of ***N*-ethylaniline (3bb).**

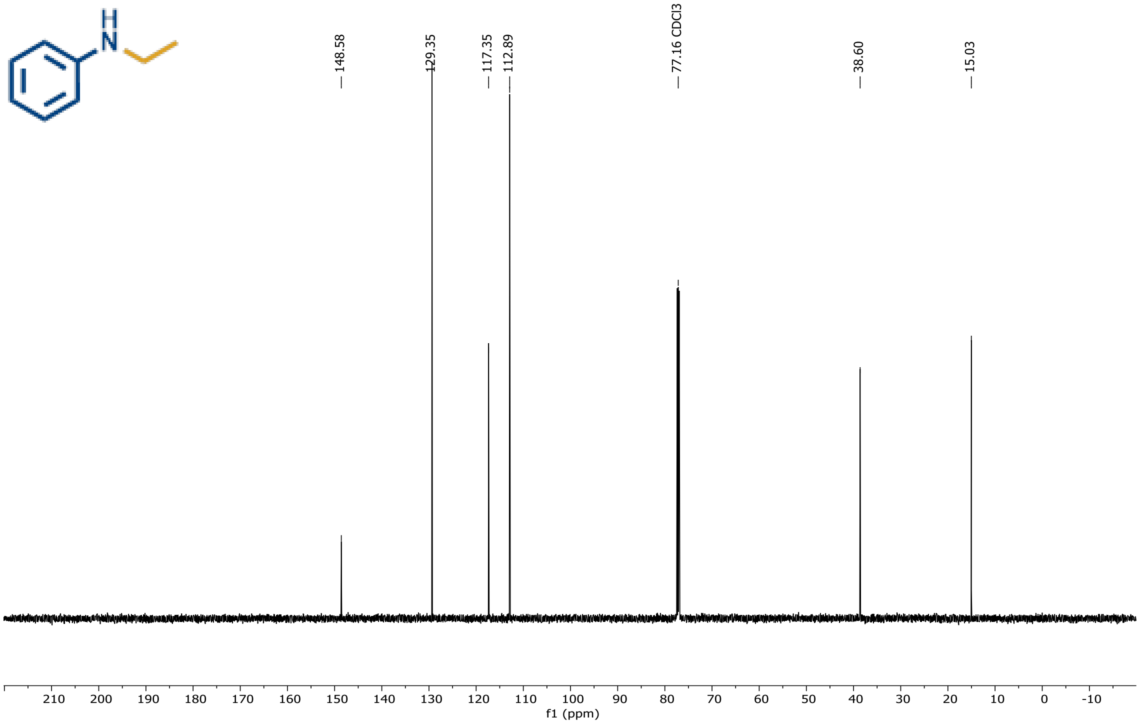

^{1}H NMR (600 MHz, $CDCl_3$) spectrum of ***N*-butylaniline (3bd).**

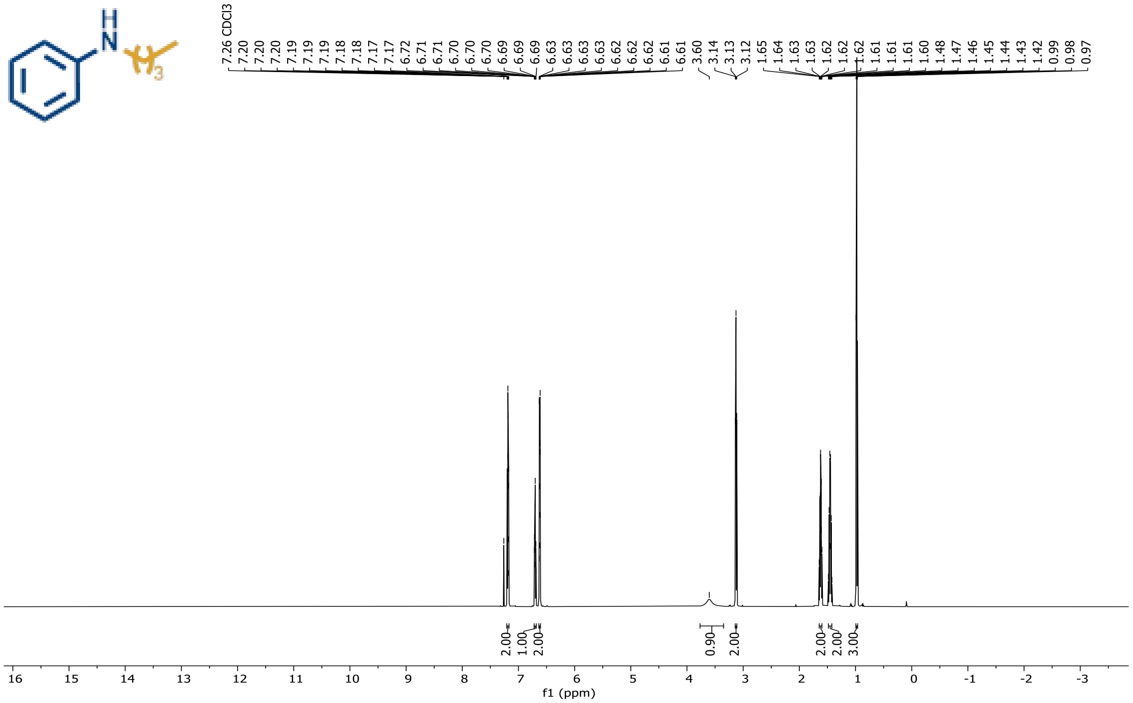

^{13}C NMR (151 MHz, $CDCl_3$) spectrum of ***N*-butylaniline (3bd).**

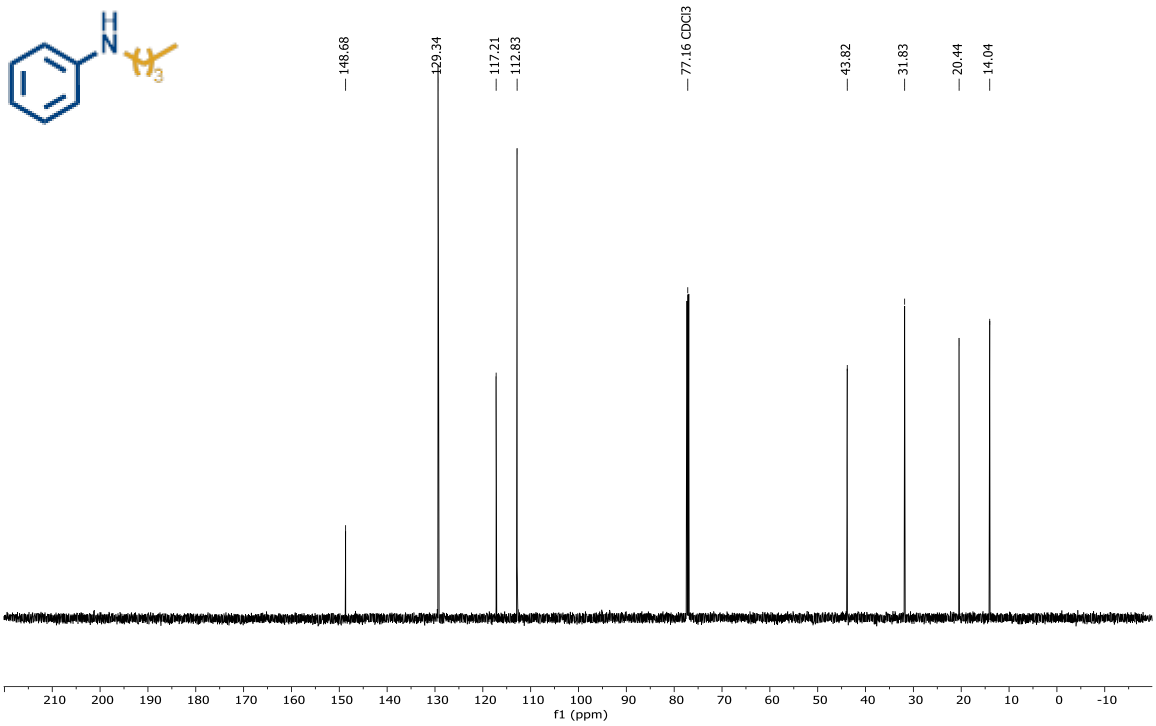

^{1}H NMR (600 MHz, $CDCl_3$) spectrum of ***N*-propylaniline (3be).**

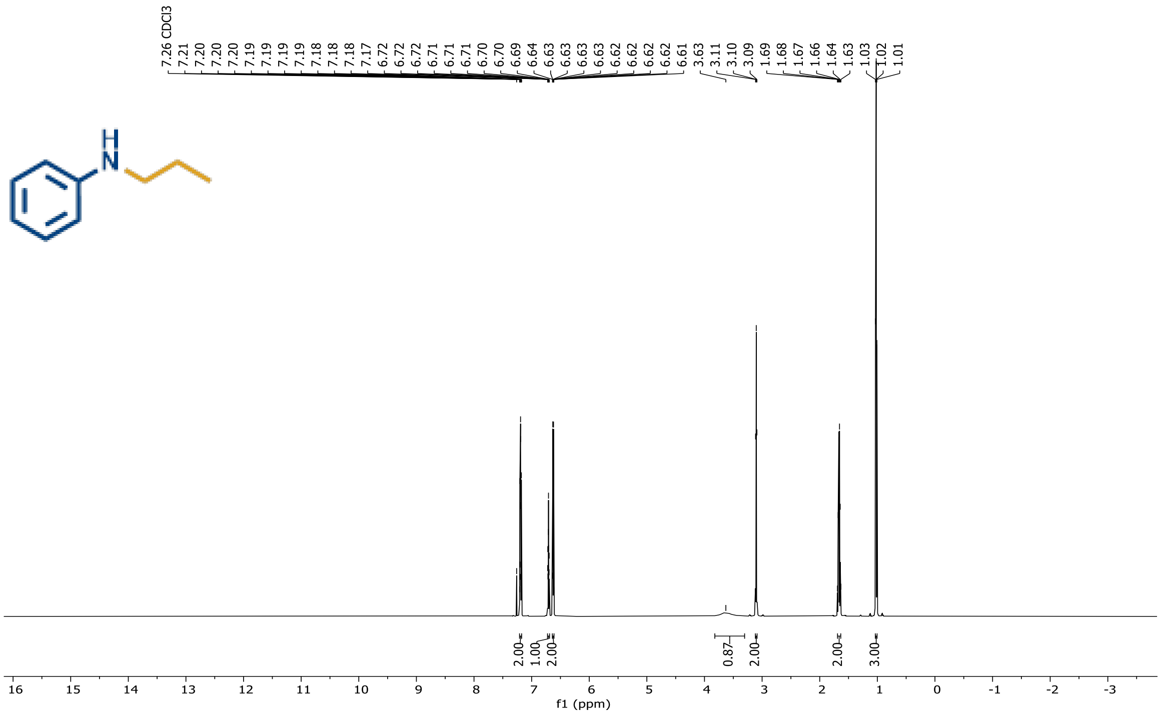

^{13}C NMR (151 MHz, $CDCl_3$) spectrum of ***N*-propylaniline (3be).**

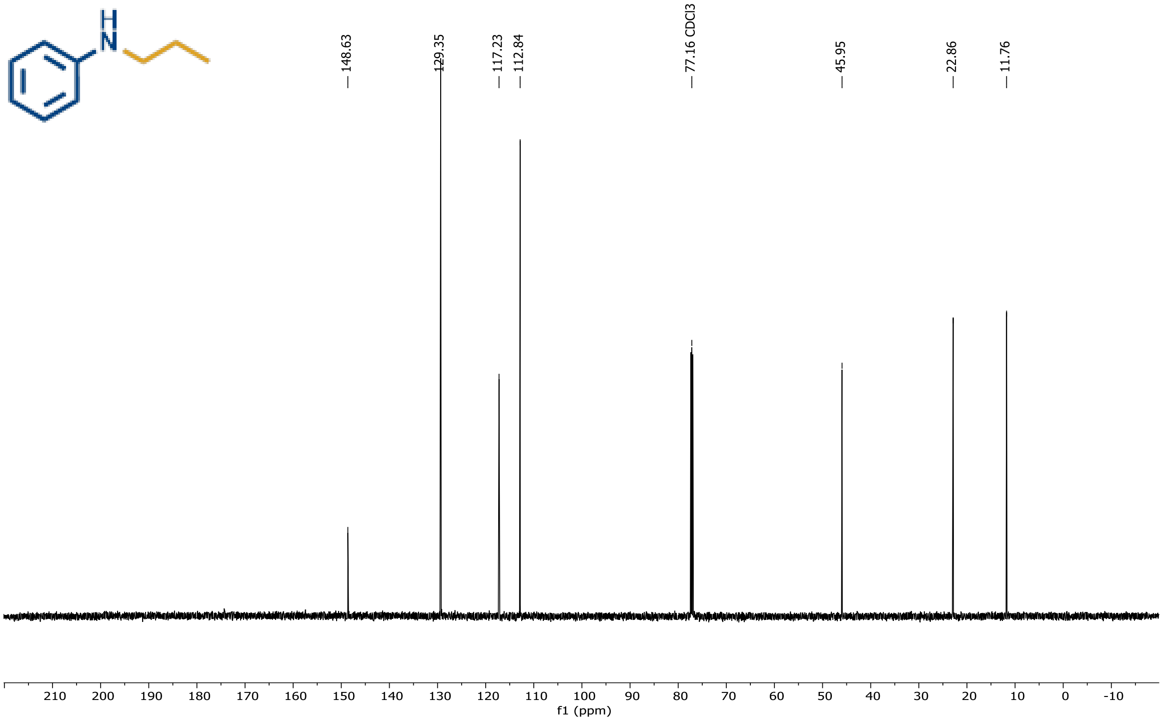

^{1}H NMR (600 MHz, $CDCl_3$) spectrum of ***N*-dodecylaniline (3bh).**

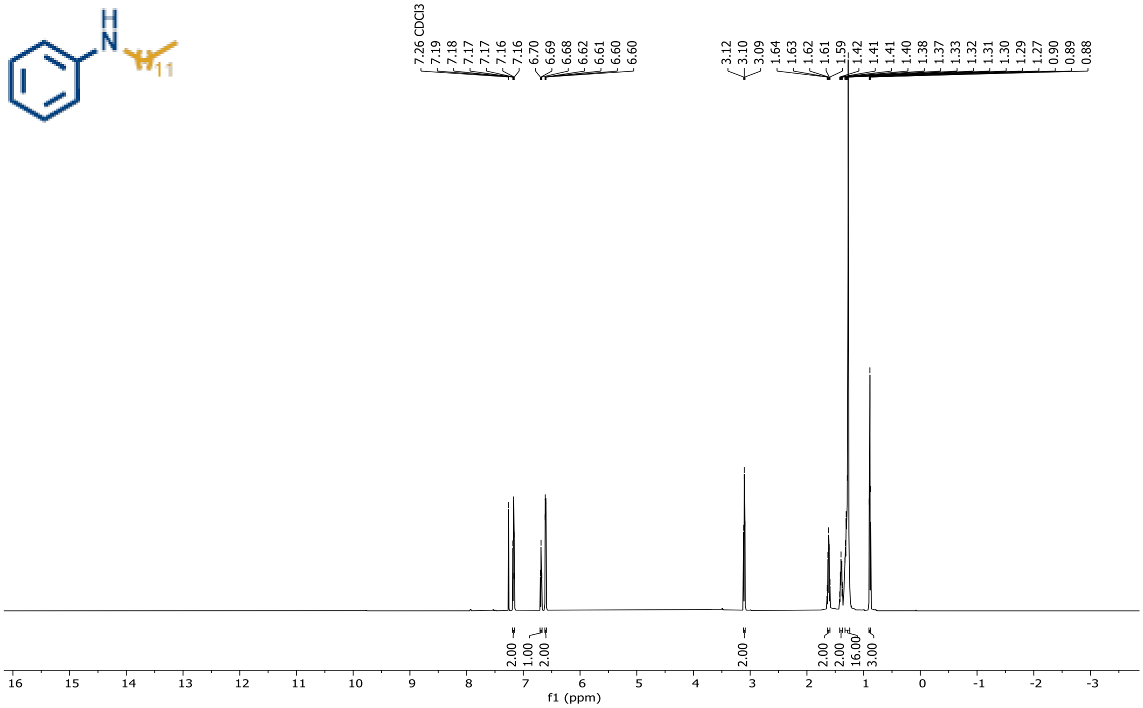

^{13}C NMR (151 MHz, $CDCl_3$) spectrum of ***N*-dodecylaniline (3bh).**

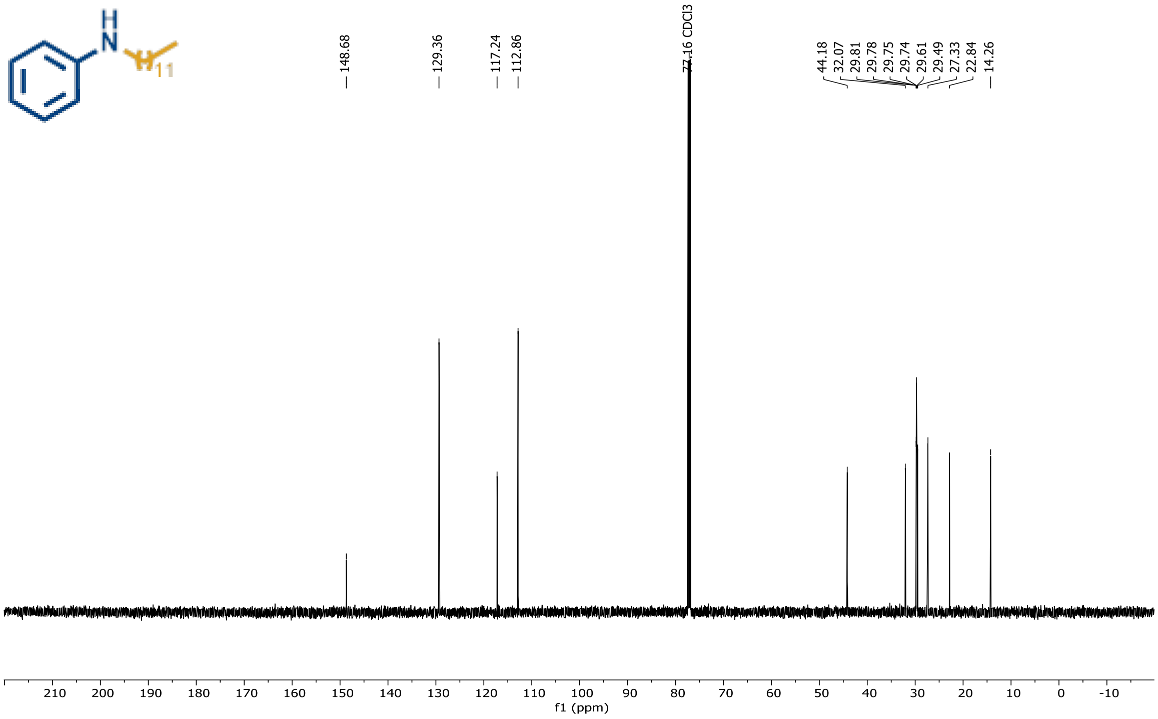

^{1}H NMR (600 MHz, $CDCl_3$) spectrum of ***N*-benzylaniline (3bi).**

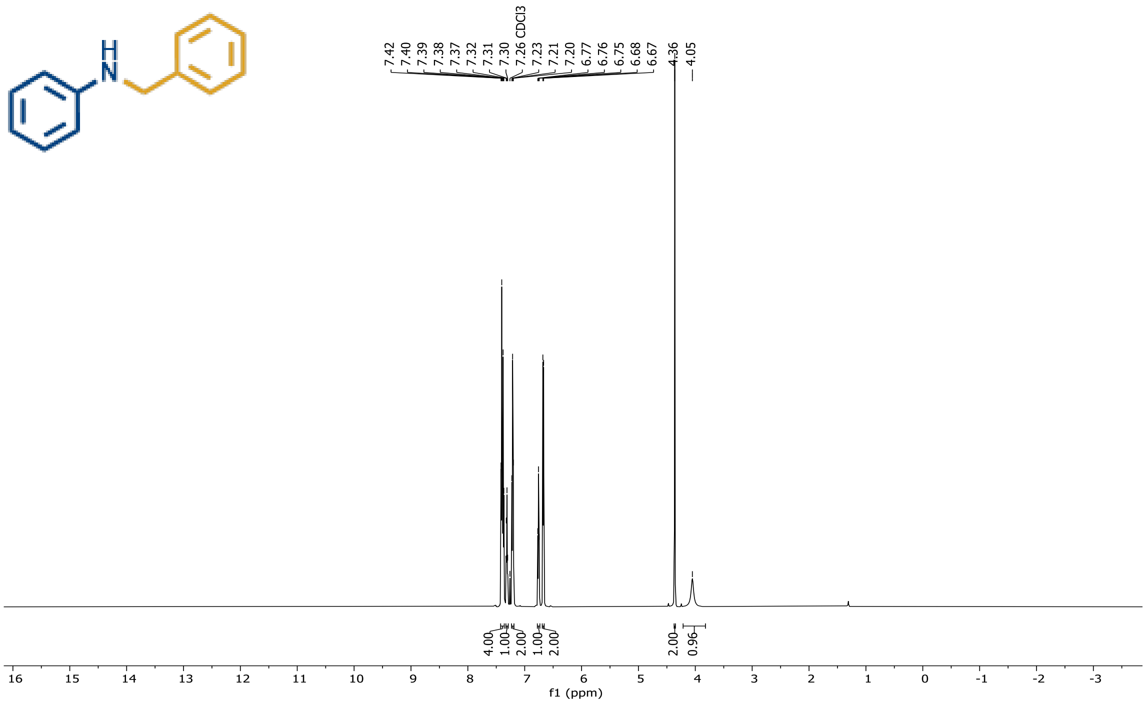

^{13}C NMR (151 MHz, $CDCl_3$) spectrum of ***N*-benzylaniline (3bi).**

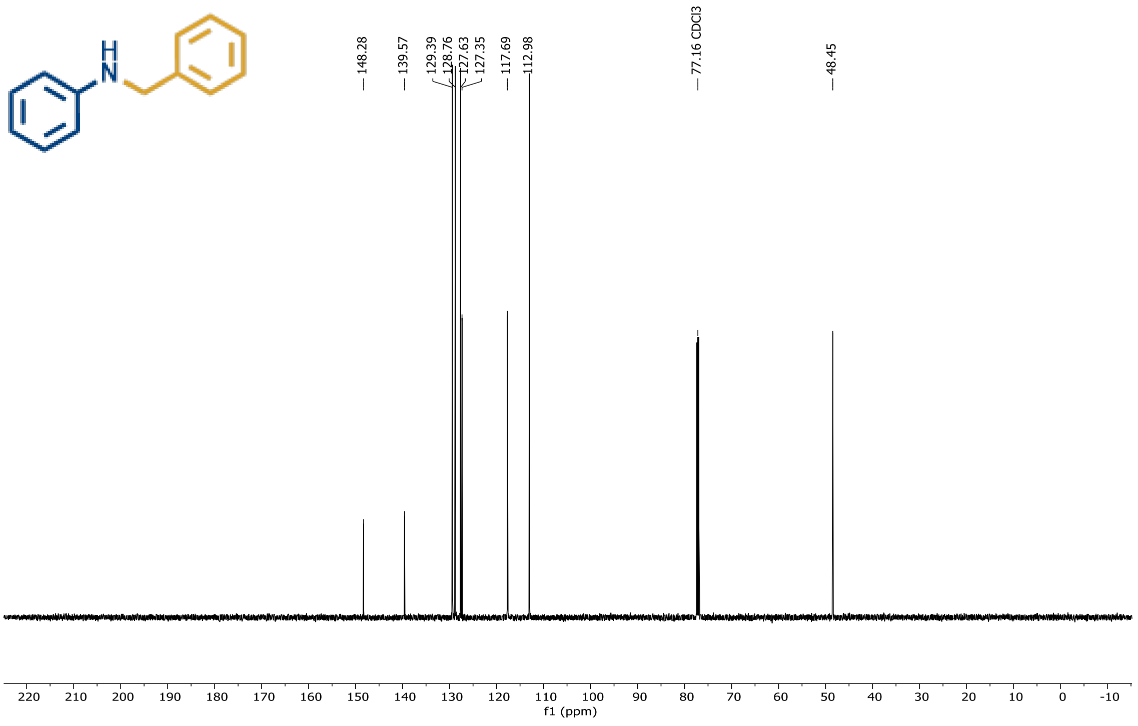

^{1}H NMR (600 MHz, $CDCl_3$) spectrum of ***N*-(undec-10-en-1-yl)aniline (3bl).**

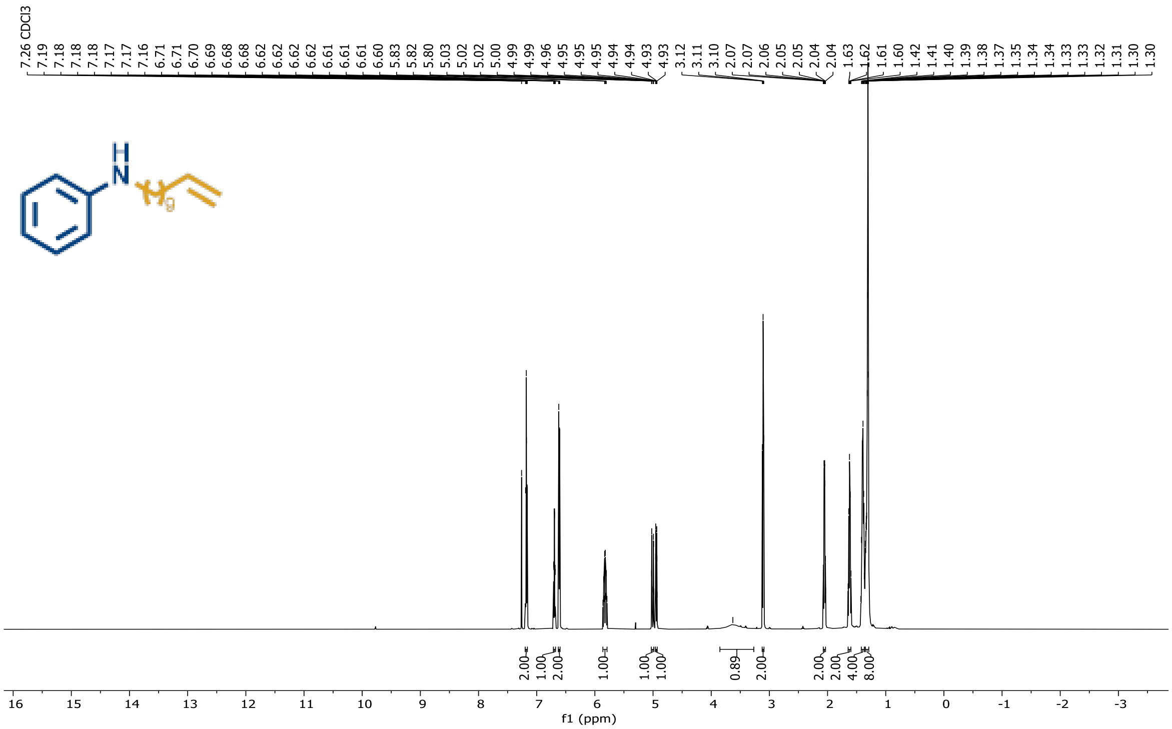

^{13}C NMR (151 MHz, $CDCl_3$) spectrum of ***N*-(undec-10-en-1-yl)aniline (3bl).**

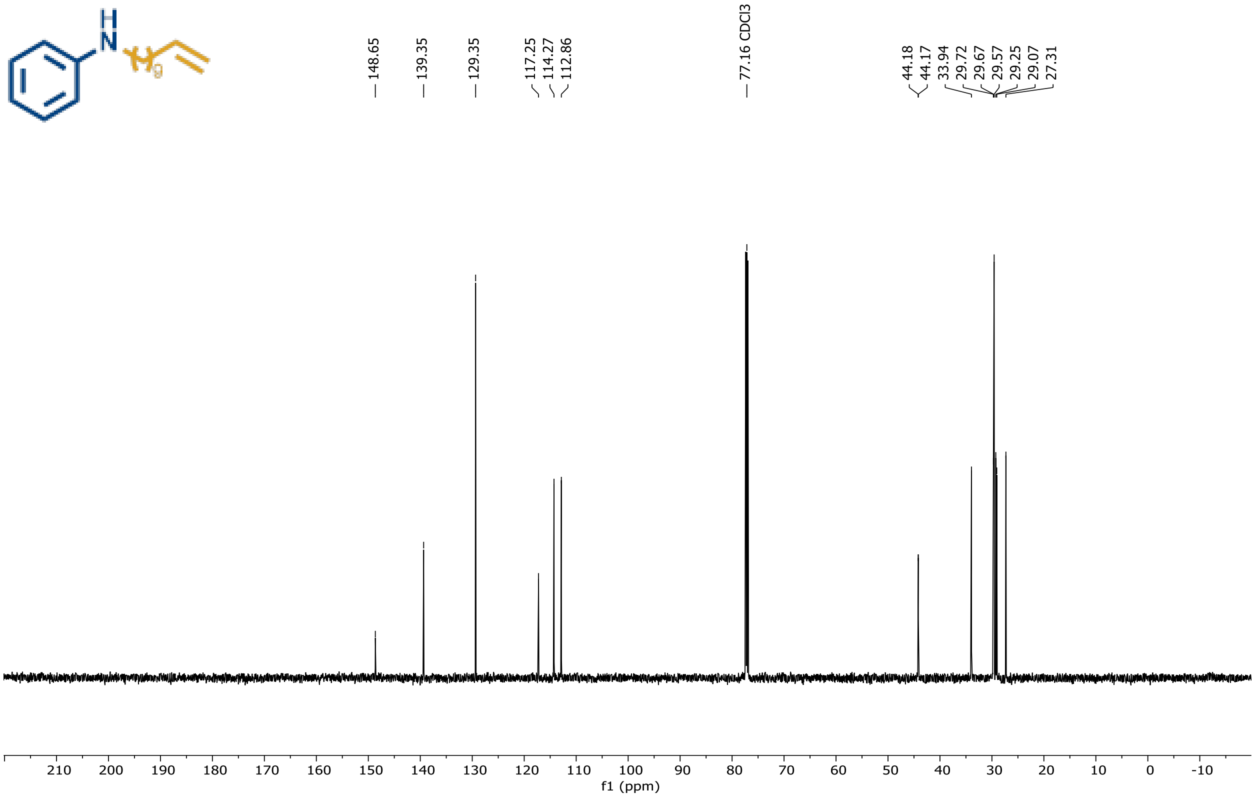

^{1}H NMR (600 MHz, $CDCl_3$) spectrum of **4-methyl-*N*-pentylaniline (3ca).**

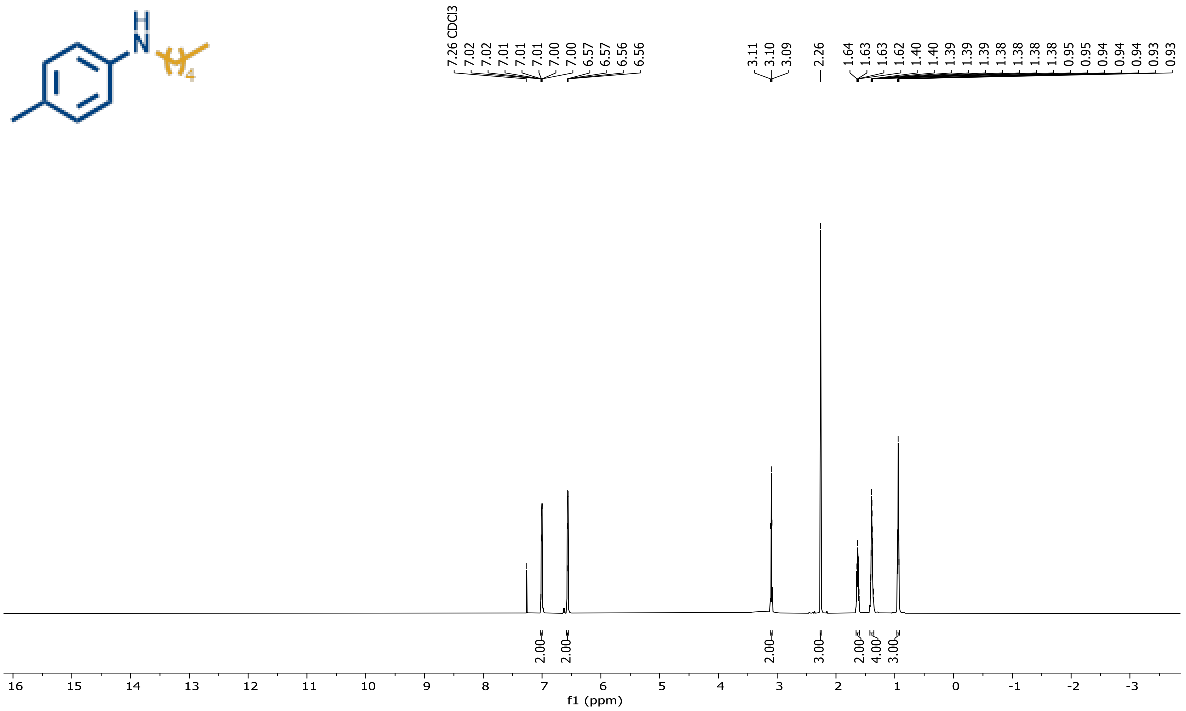

^{13}C NMR (151 MHz, $CDCl_3$) spectrum of **4-methyl-*N*-pentylaniline (3ca).**

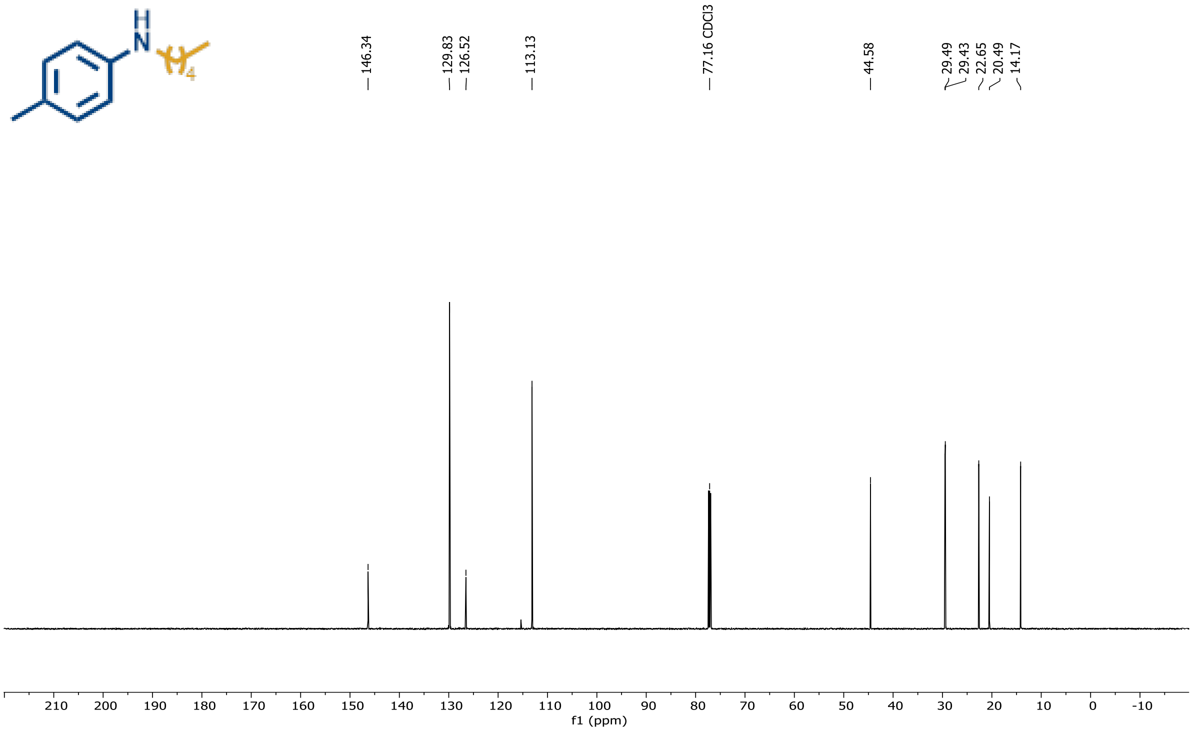

^1H NMR (600 MHz, $CDCl_3$) spectrum of **4-bromo-*N*-pentylaniline (3da).**

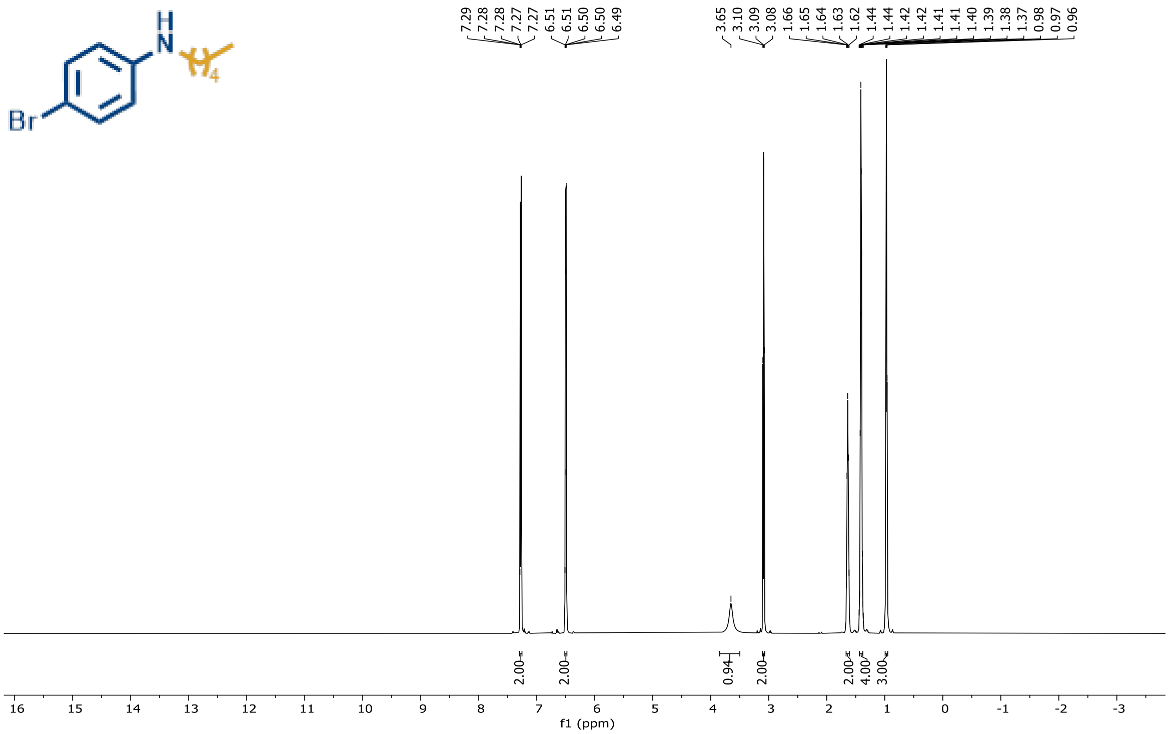

^{13}C NMR (151 MHz, $CDCl_3$) spectrum of **4-bromo-*N*-pentylaniline (3da).**

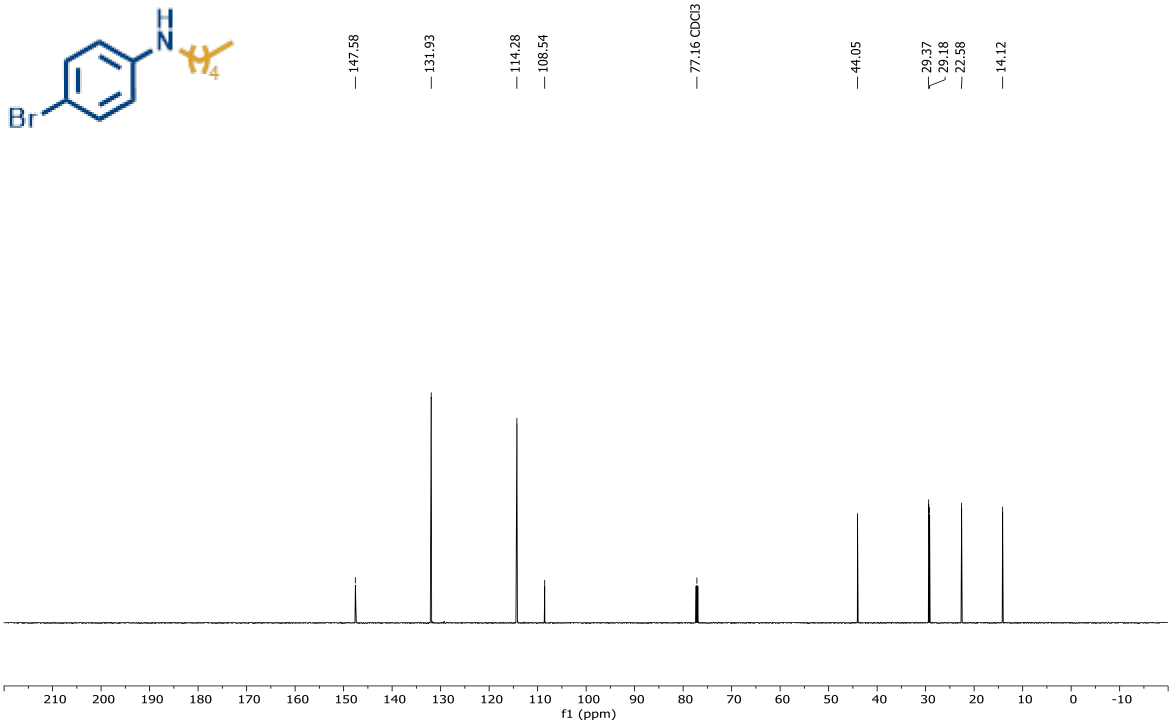

^{1}H NMR (600 MHz, $CDCl_3$) spectrum of **4-bromo-*N*-ethylaniline (3db).**

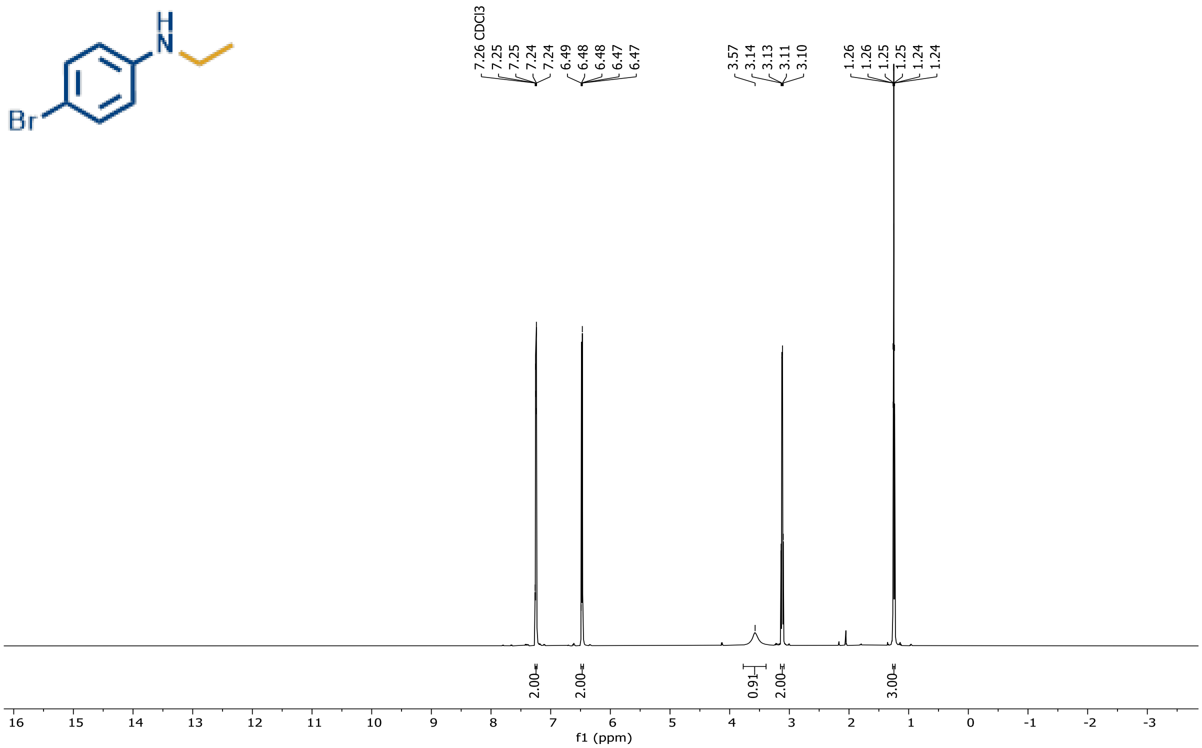

^{13}C NMR (151 MHz, $CDCl_3$) spectrum of **4-bromo-*N*-ethylaniline (3db).**

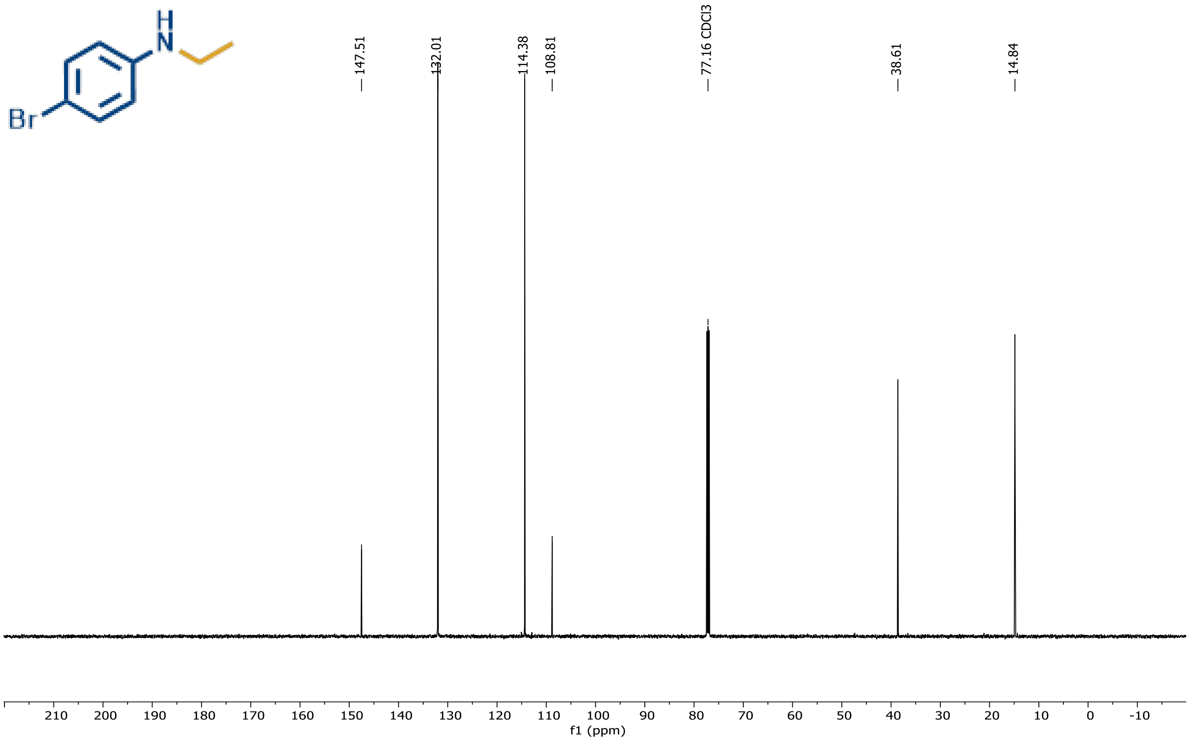

^{1}H NMR (600 MHz, $CDCl_3$) spectrum of **4-bromo-*N*-hexylaniline (3dc).**

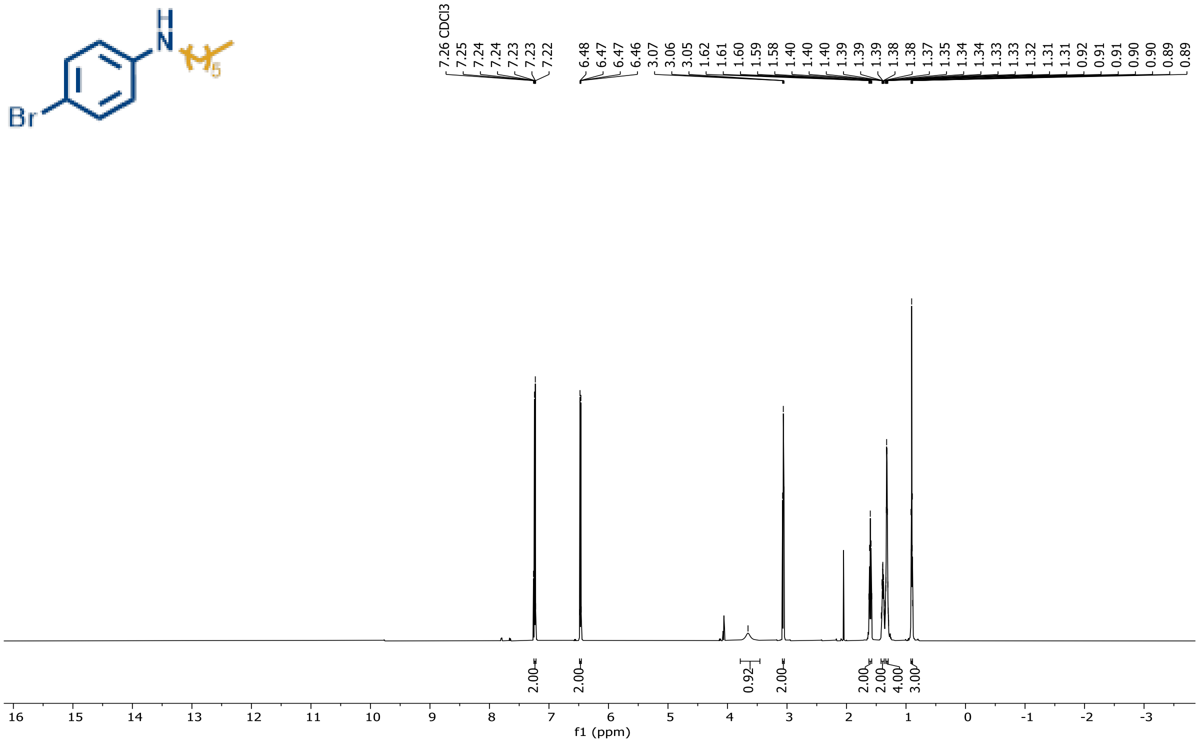

^{13}C NMR (151 MHz, $CDCl_3$) spectrum of **4-bromo-*N*-hexylaniline (3dc).**

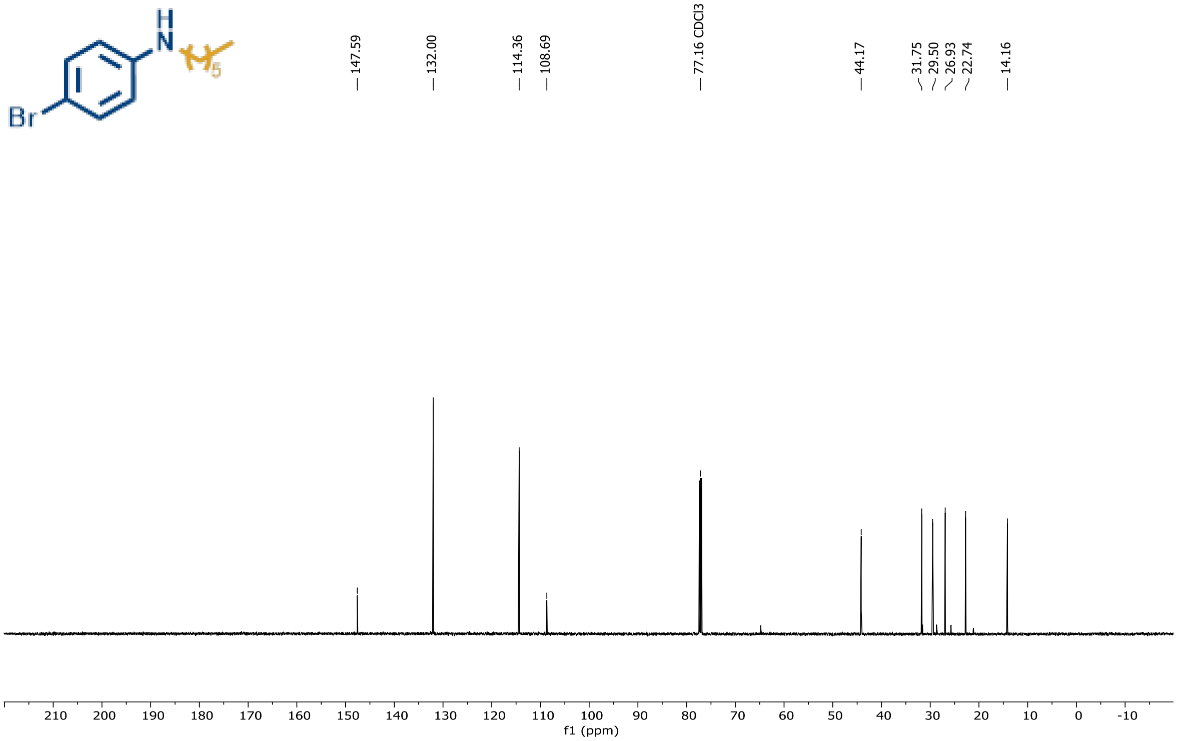

^{1}H NMR (600 MHz, $CDCl_3$) spectrum of **4-bromo-*N*-butylaniline (3dd).**

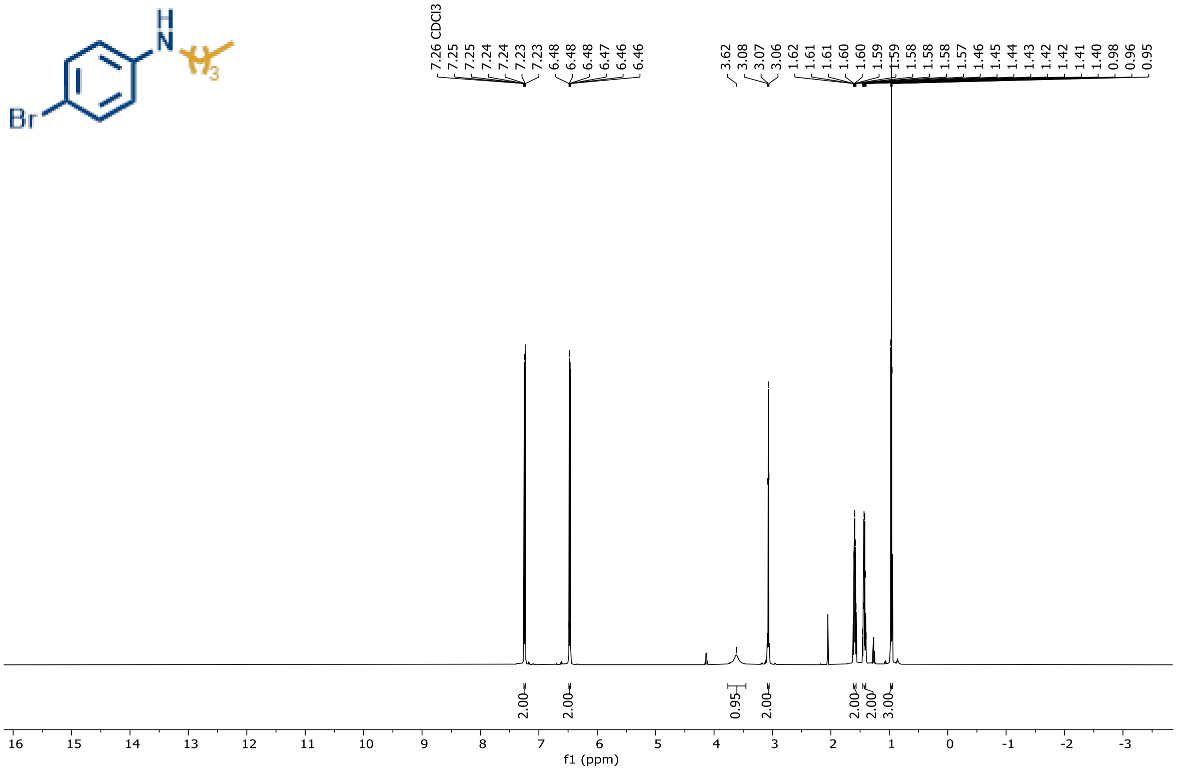

^{13}C NMR (151 MHz, $CDCl_3$) spectrum of **4-bromo-*N*-butylaniline (3dd).**

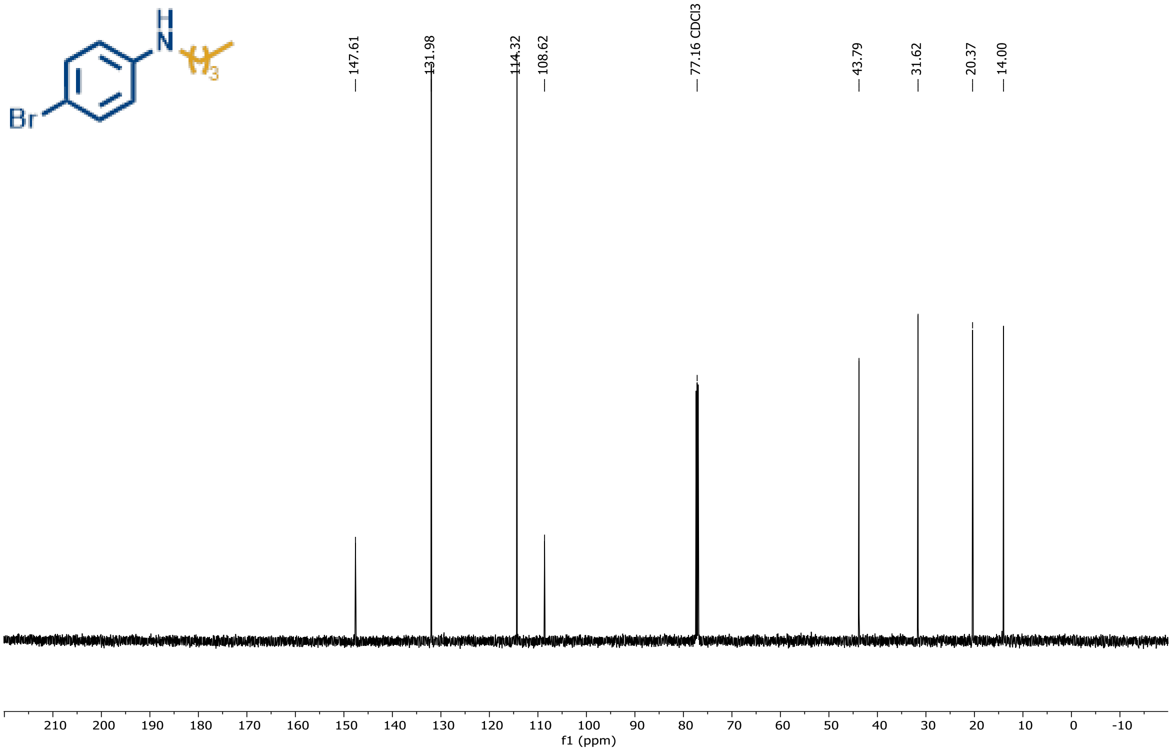

^{1}H NMR (600 MHz, $CDCl_3$) spectrum of ***N*-benzyl-4-bromoaniline (3di).**

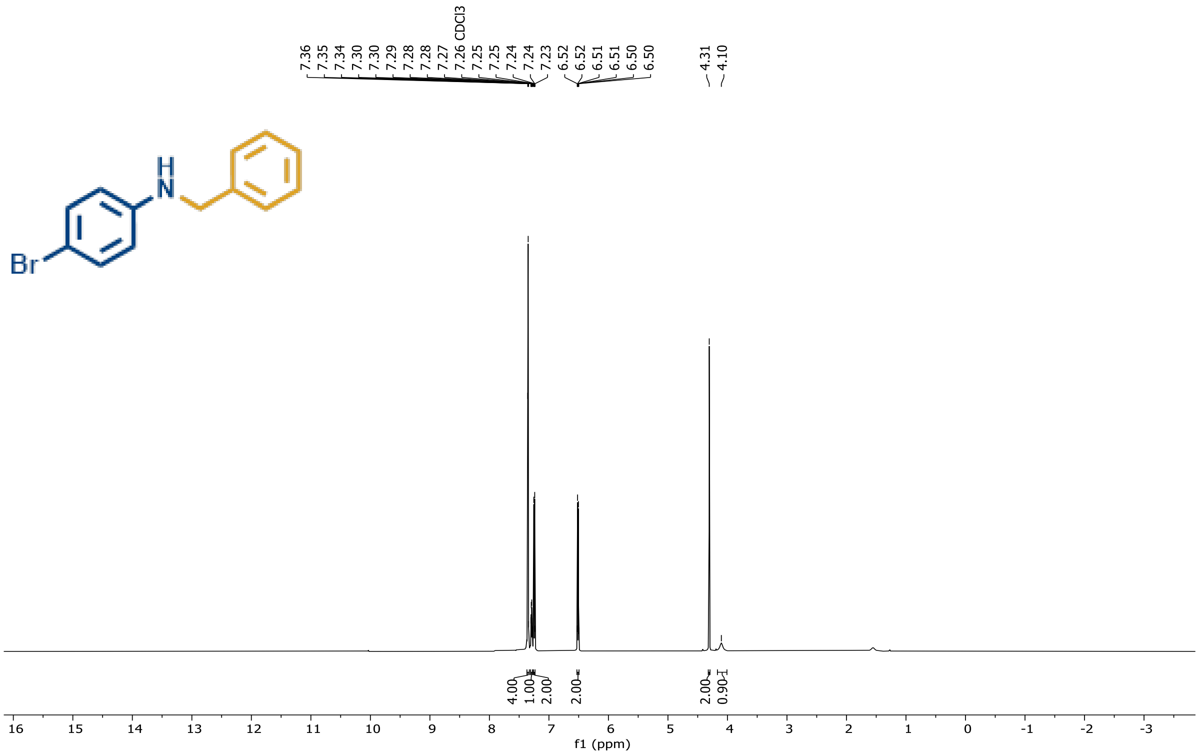

^{13}C NMR (151 MHz, $CDCl_3$) spectrum of ***N*-benzyl-4-bromoaniline (3di).**

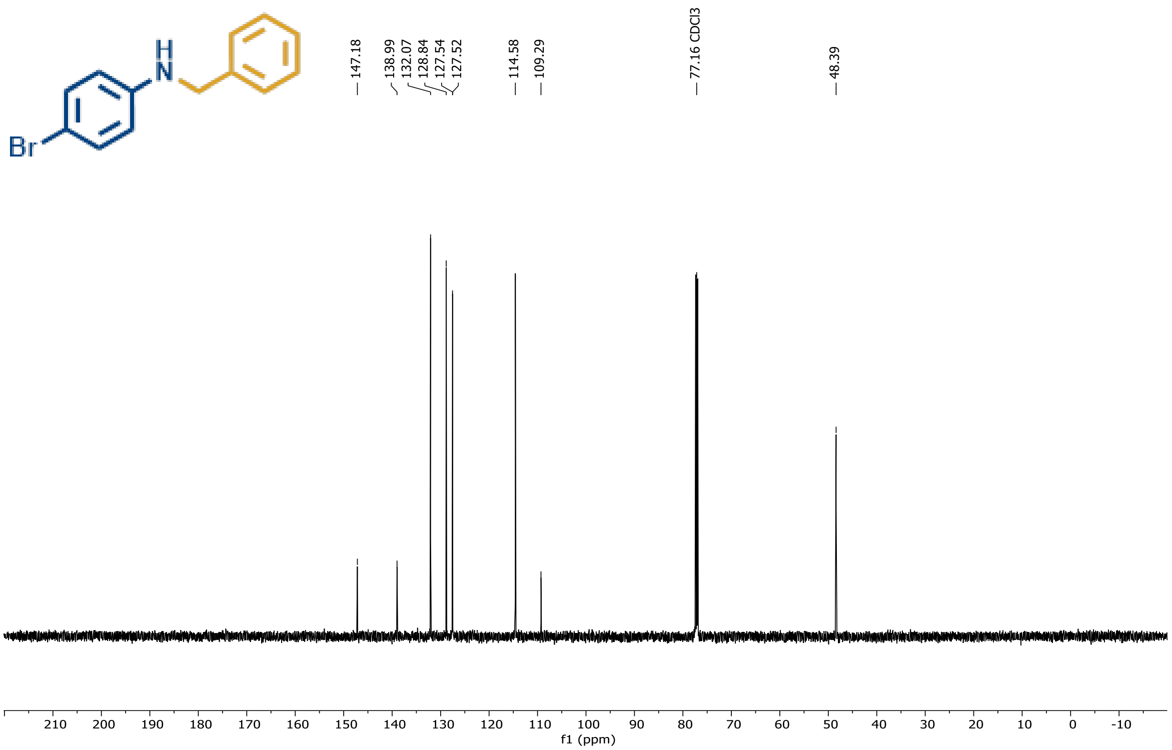

^{1}H NMR (600 MHz, $CDCl_3$) spectrum of **4-chloro-*N*-pentylaniline (3ea).**

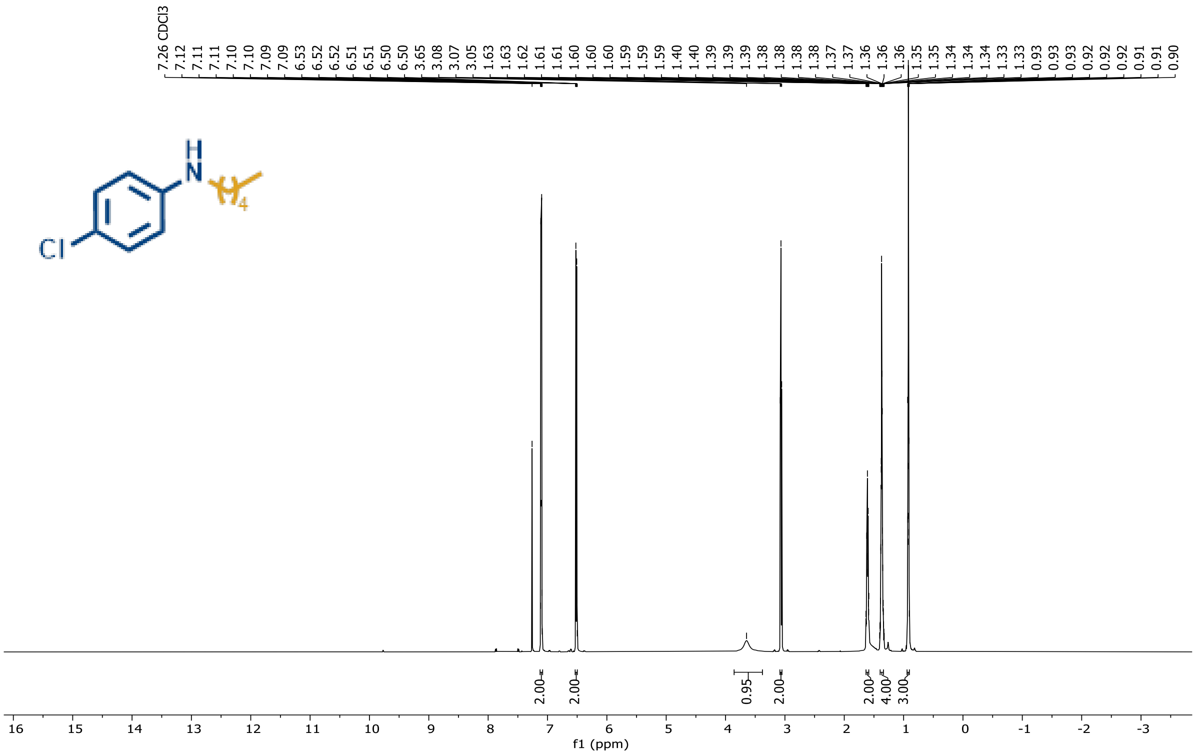

^{13}C NMR (151 MHz, $CDCl_3$) spectrum of **4-chloro-*N*-pentylaniline (3ea).**

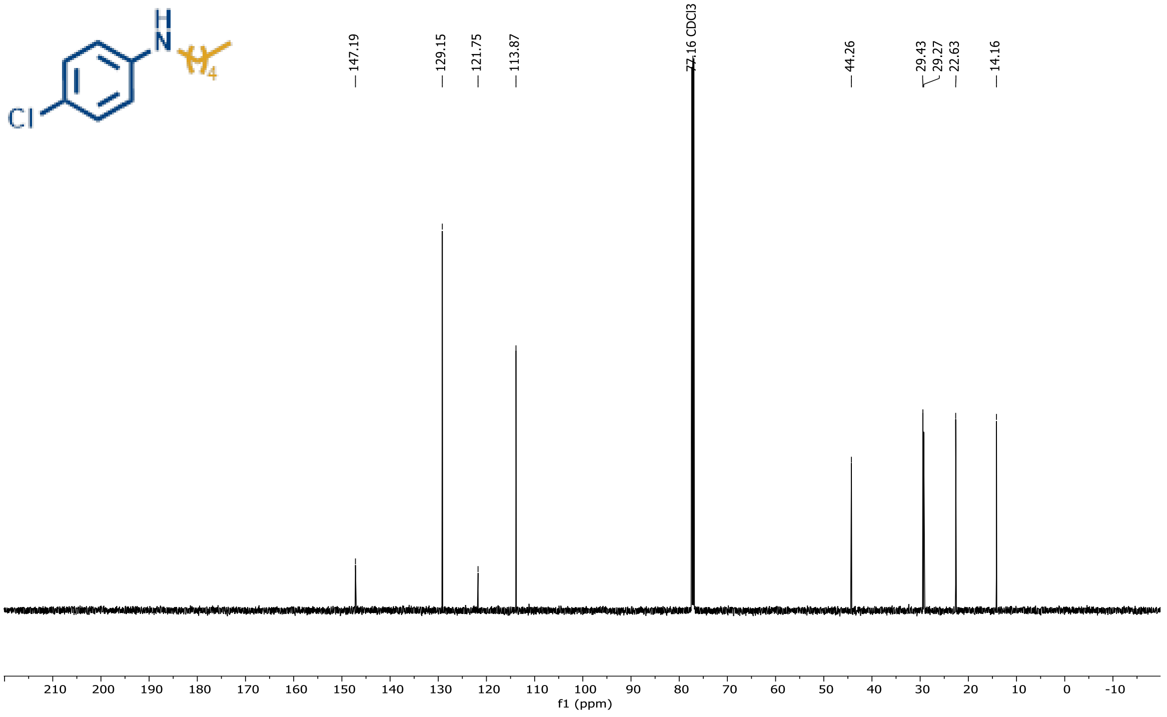

^{1}H NMR (600 MHz, $CDCl_3$) spectrum of **4-chloro-*N*-ethylaniline (3eb).**

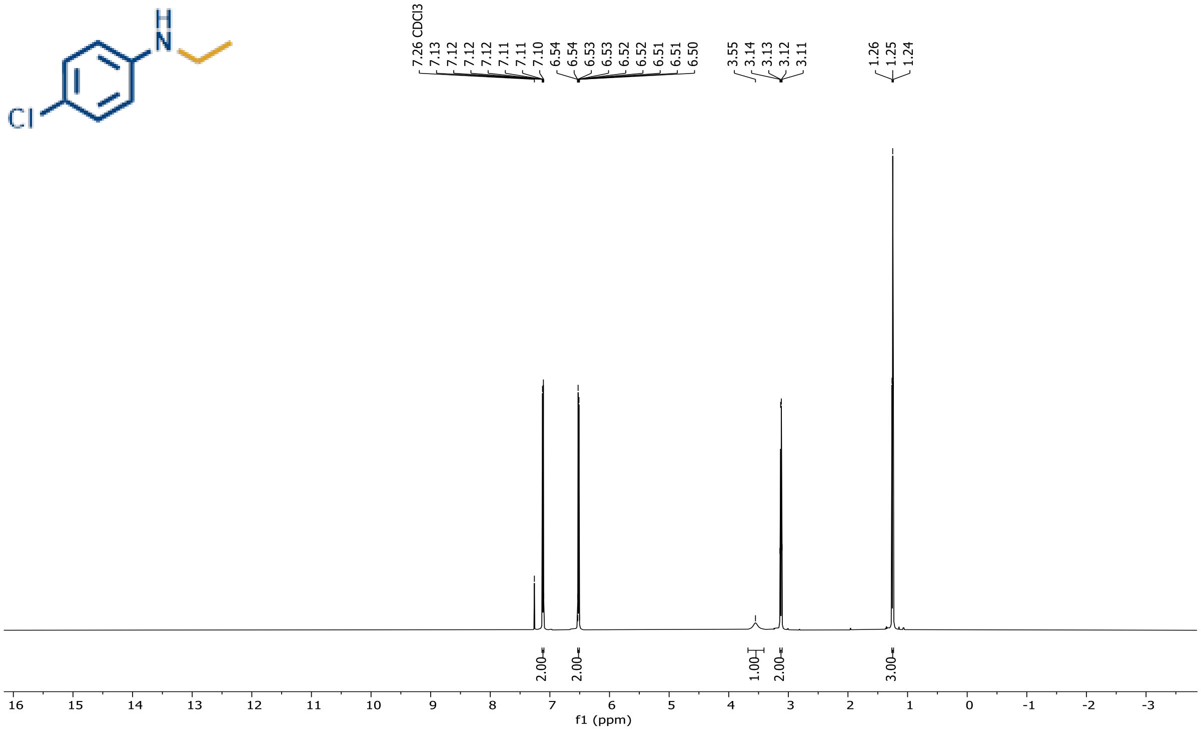

^{13}C NMR (151 MHz, $CDCl_3$) spectrum of **4-chloro-*N*-ethylaniline (3eb).**

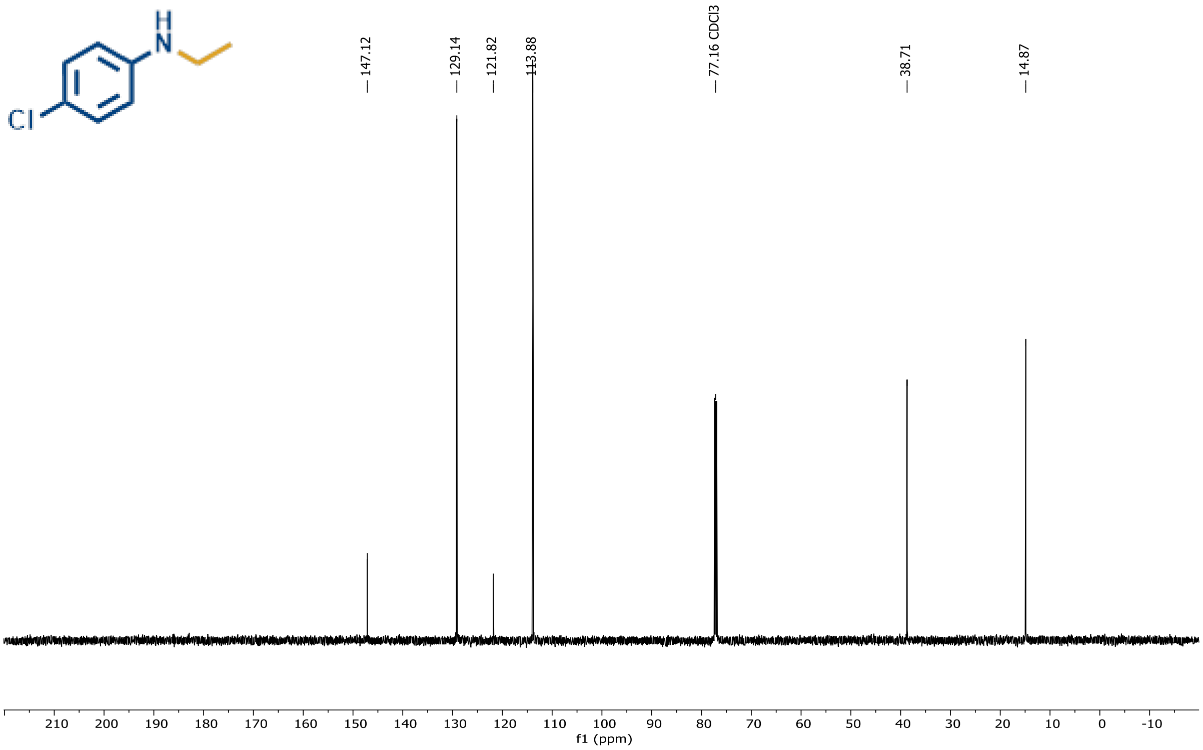

^{1}H NMR (600 MHz, $CDCl_3$) spectrum of ***N*-butyl-4-chloroaniline (3ed).**

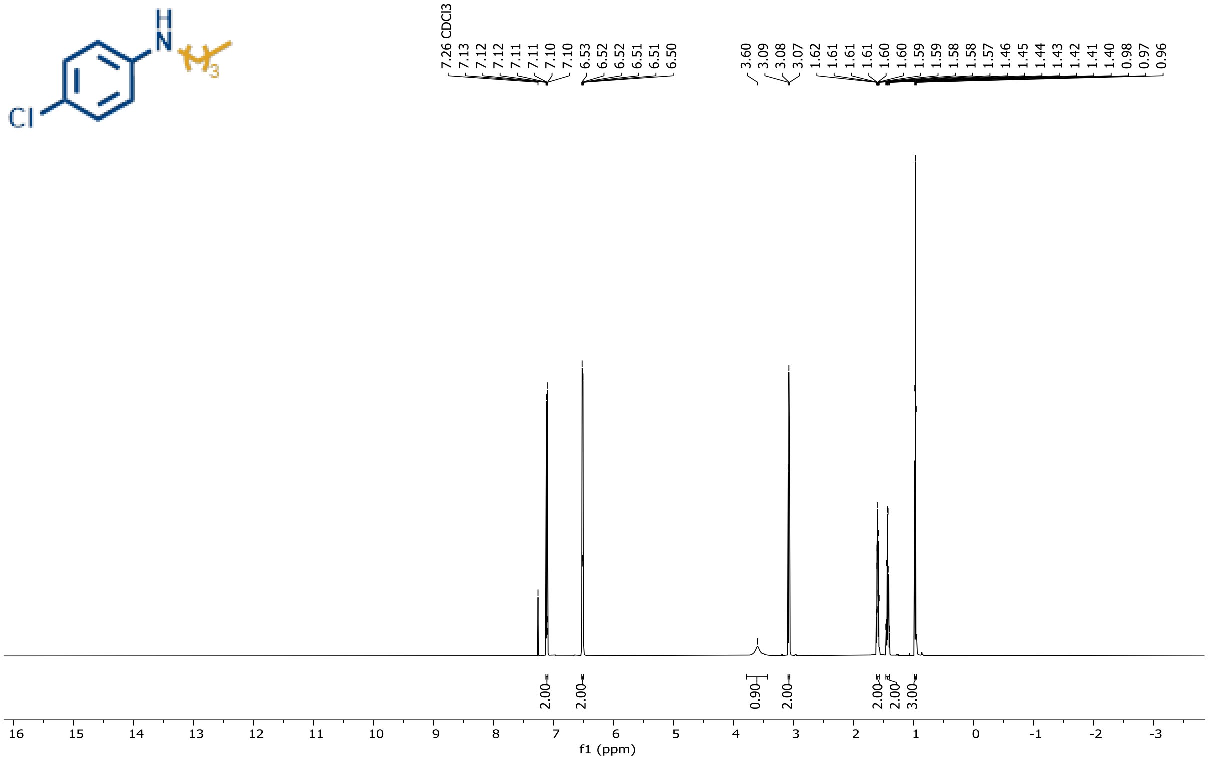

^{13}C NMR (151 MHz, $CDCl_3$) spectrum of ***N*-butyl-4-chloroaniline (3ed).**

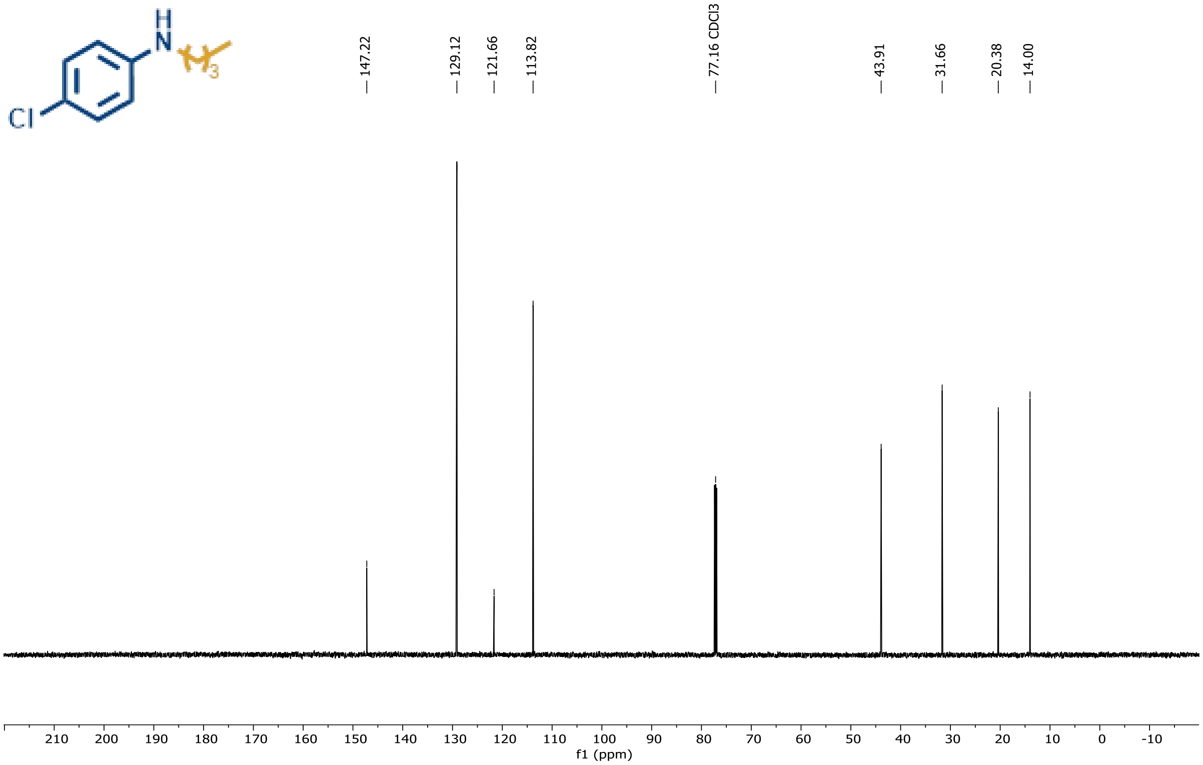

^{1}H NMR (600 MHz, $CDCl_3$) spectrum of **4-(methylthio)-*N*-pentylaniline (3fa).**

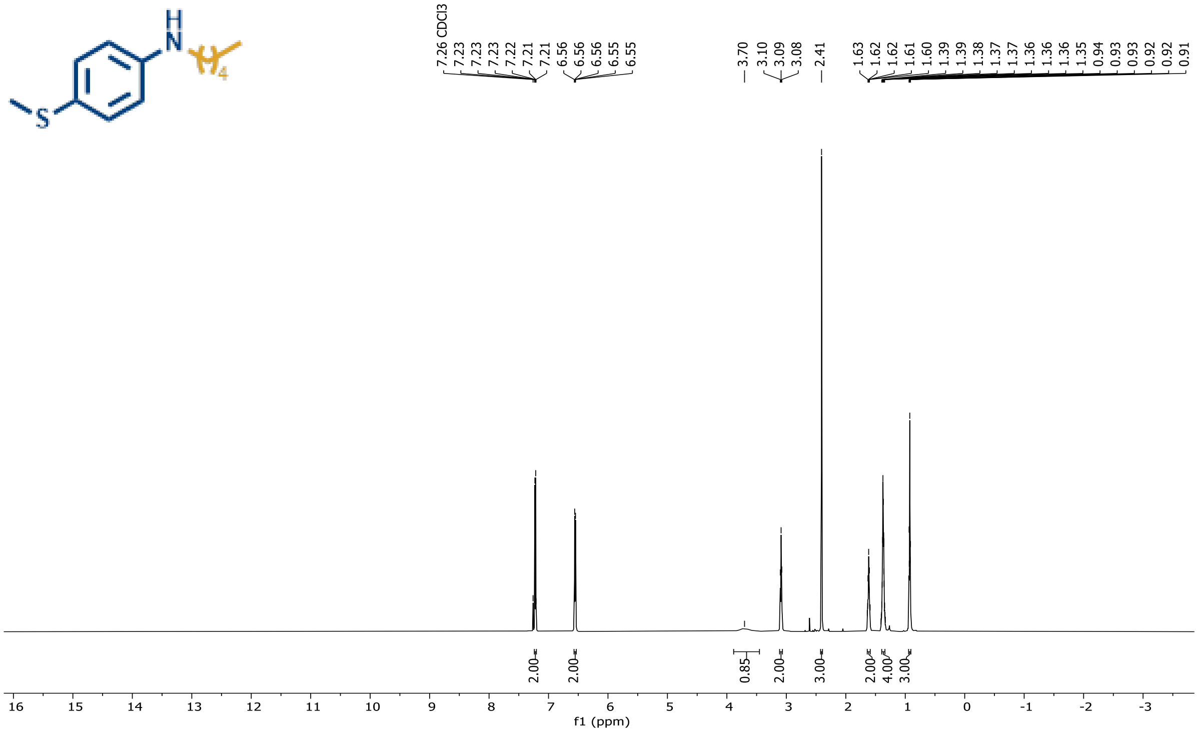

^{13}C NMR (151 MHz, $CDCl_3$) spectrum of **4-(methylthio)-*N*-pentylaniline (3fa).**

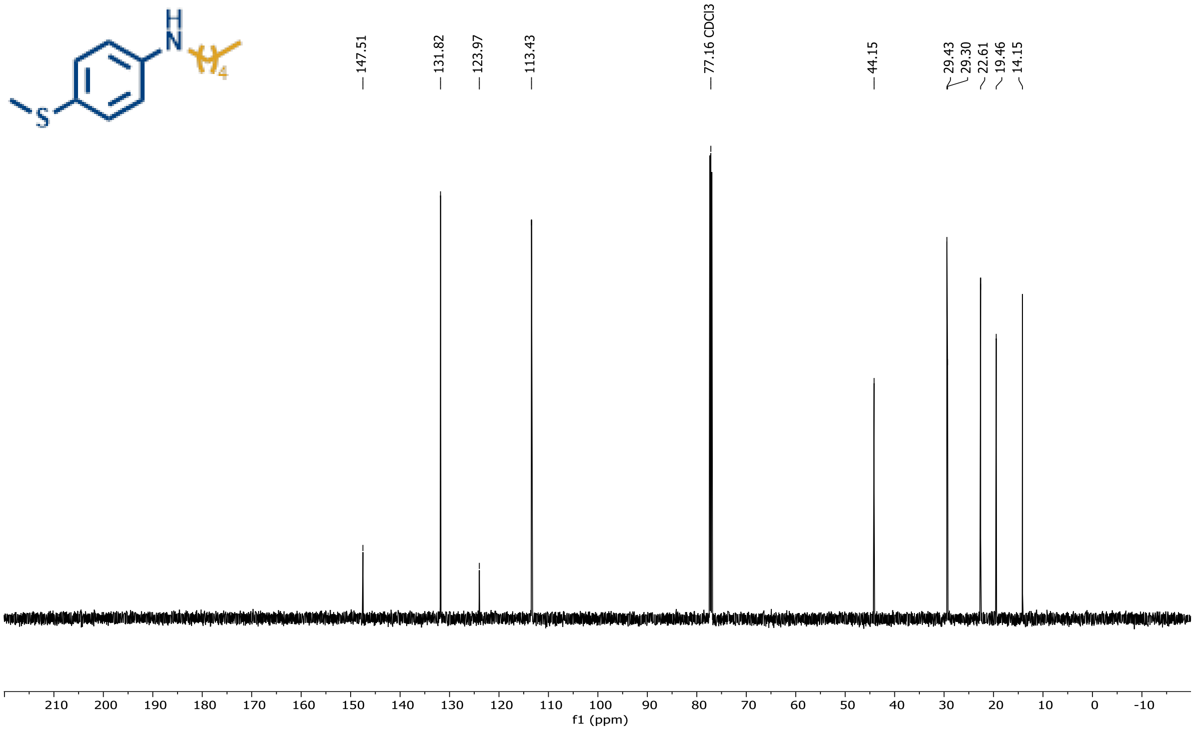

^{1}H NMR (600 MHz, $CDCl_3$) spectrum of **4-fluoro-*N*-pentylaniline (3ga).**

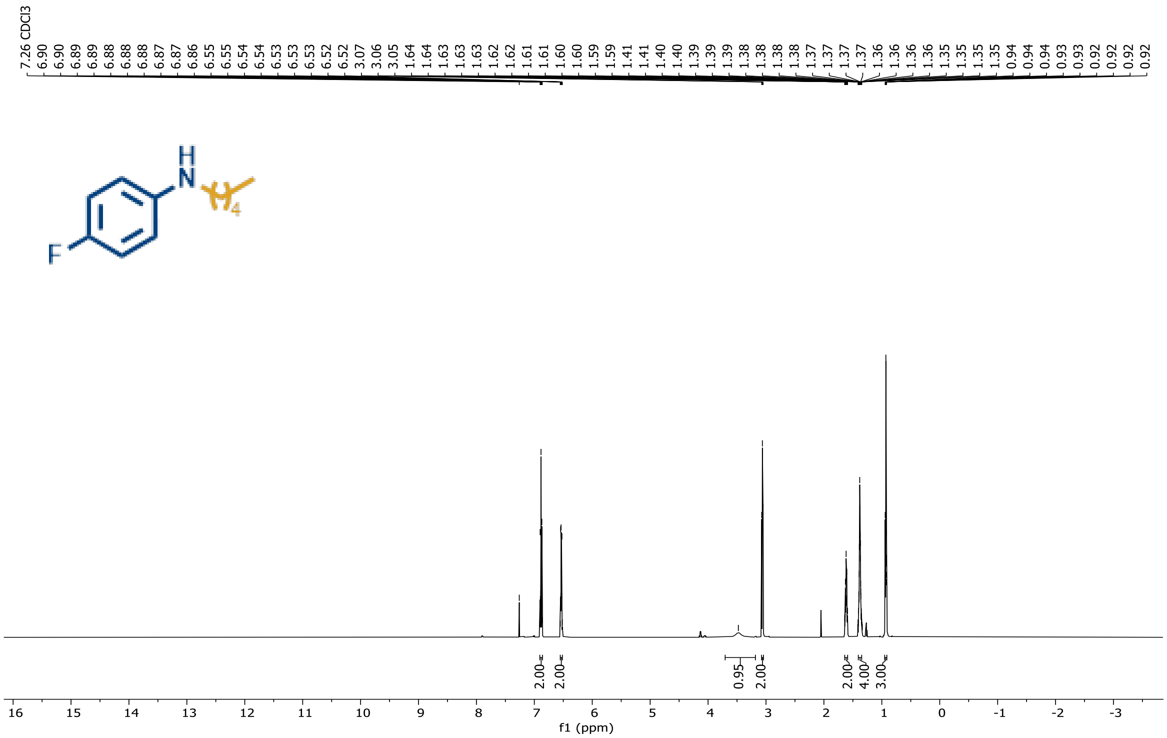

^{13}C NMR (151 MHz, $CDCl_3$) spectrum of **4-fluoro-*N*-pentylaniline (3ga).**

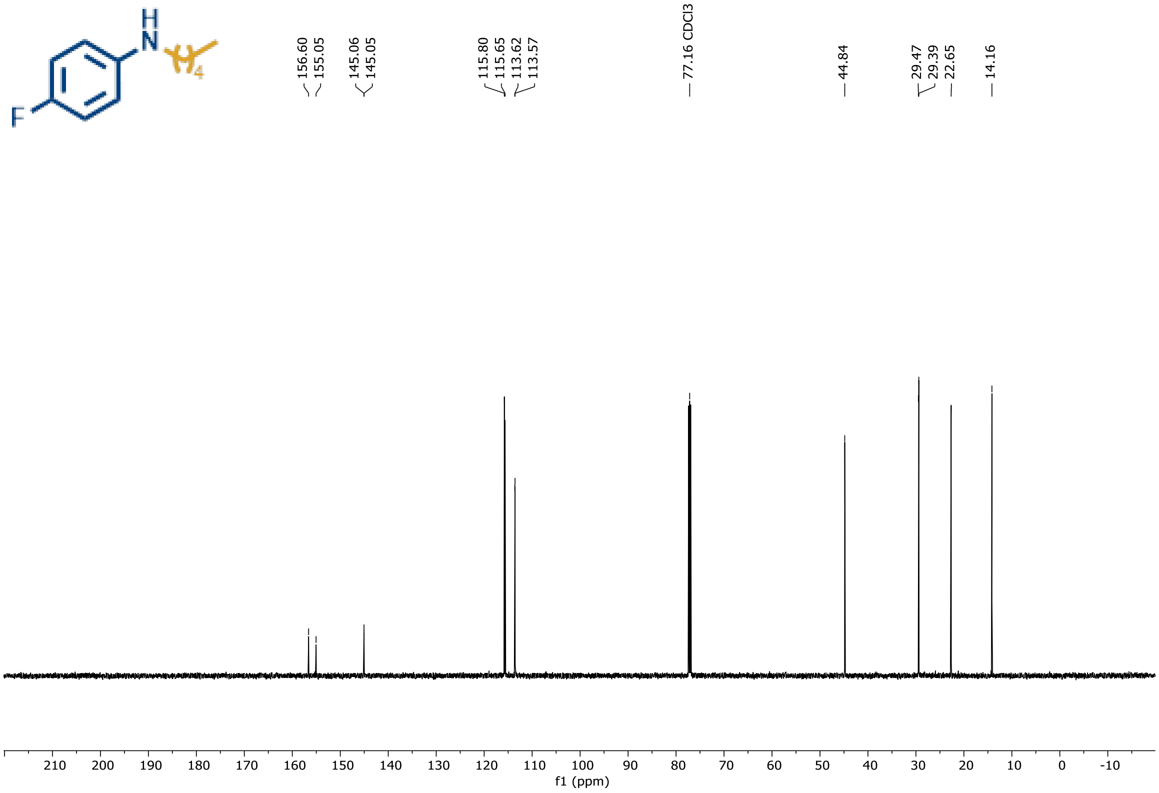

^{1}H NMR (600 MHz, $CDCl_3$) spectrum of ***N*-ethyl-4-fluoroaniline (3gb).**

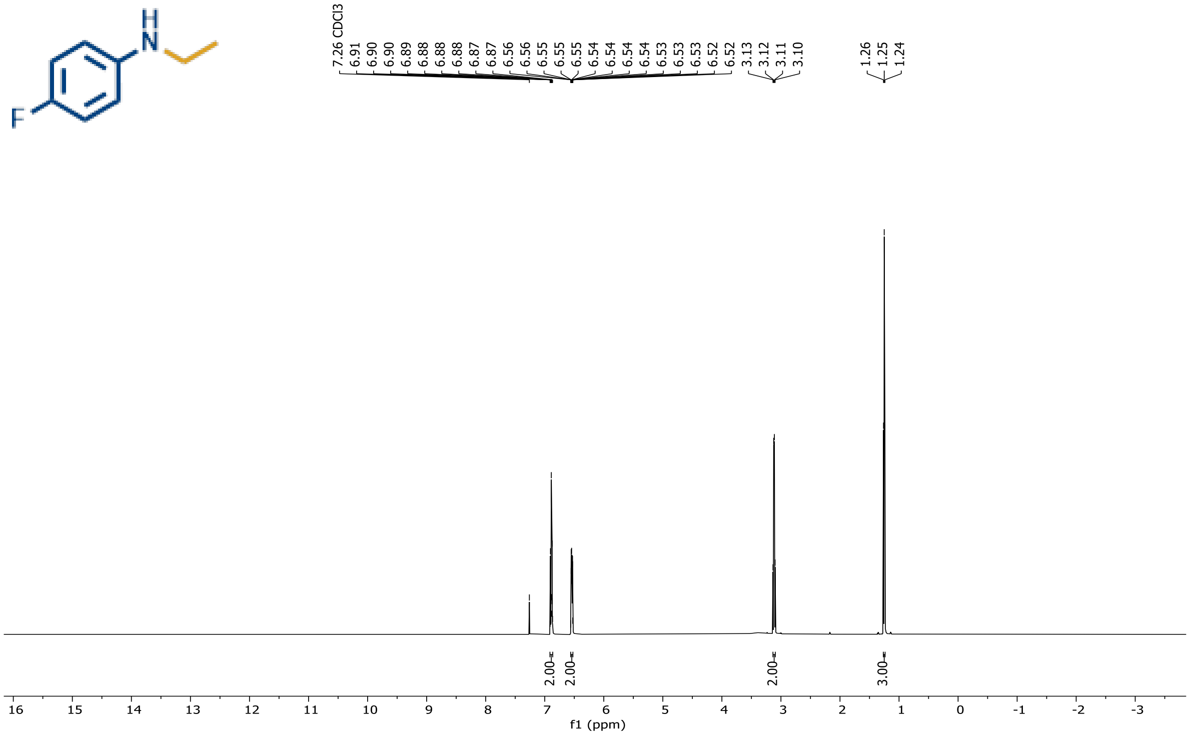

^{13}C NMR (151 MHz, $CDCl_3$) spectrum of ***N*-ethyl-4-fluoroaniline (3gb).**

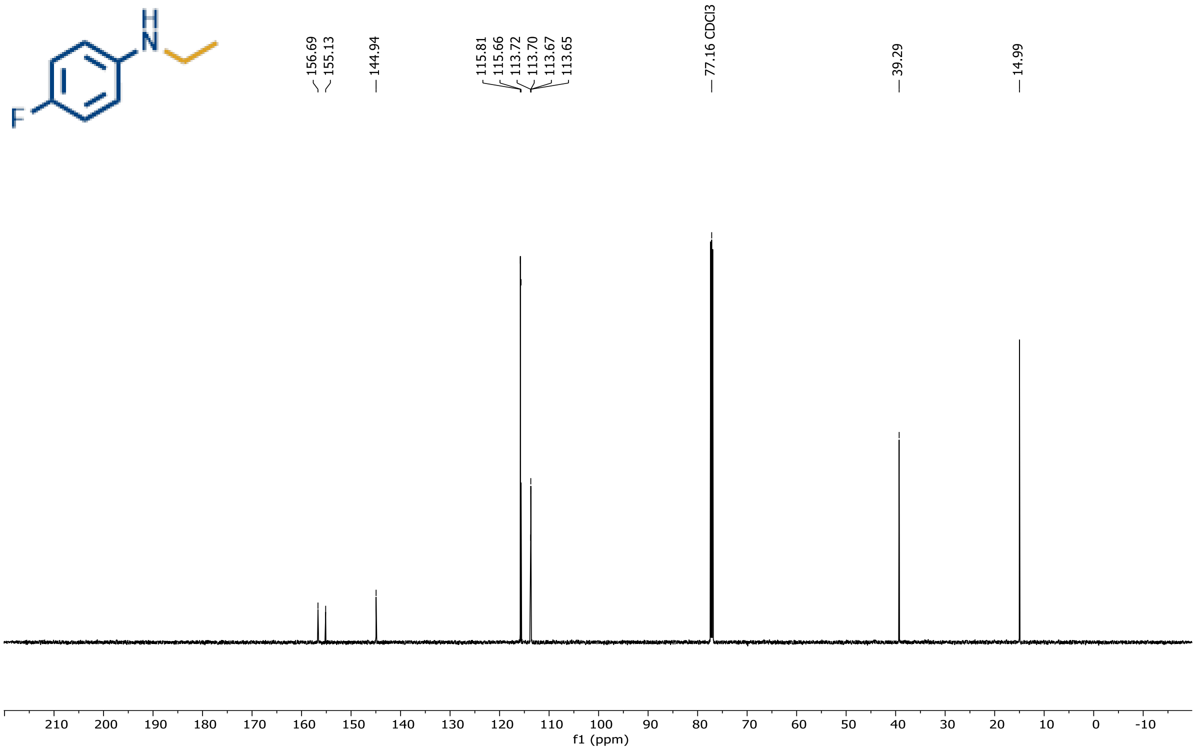

^{1}H NMR (600 MHz, $CDCl_3$) spectrum of ***N*-benzyl-4-fluoroaniline (3gi).**

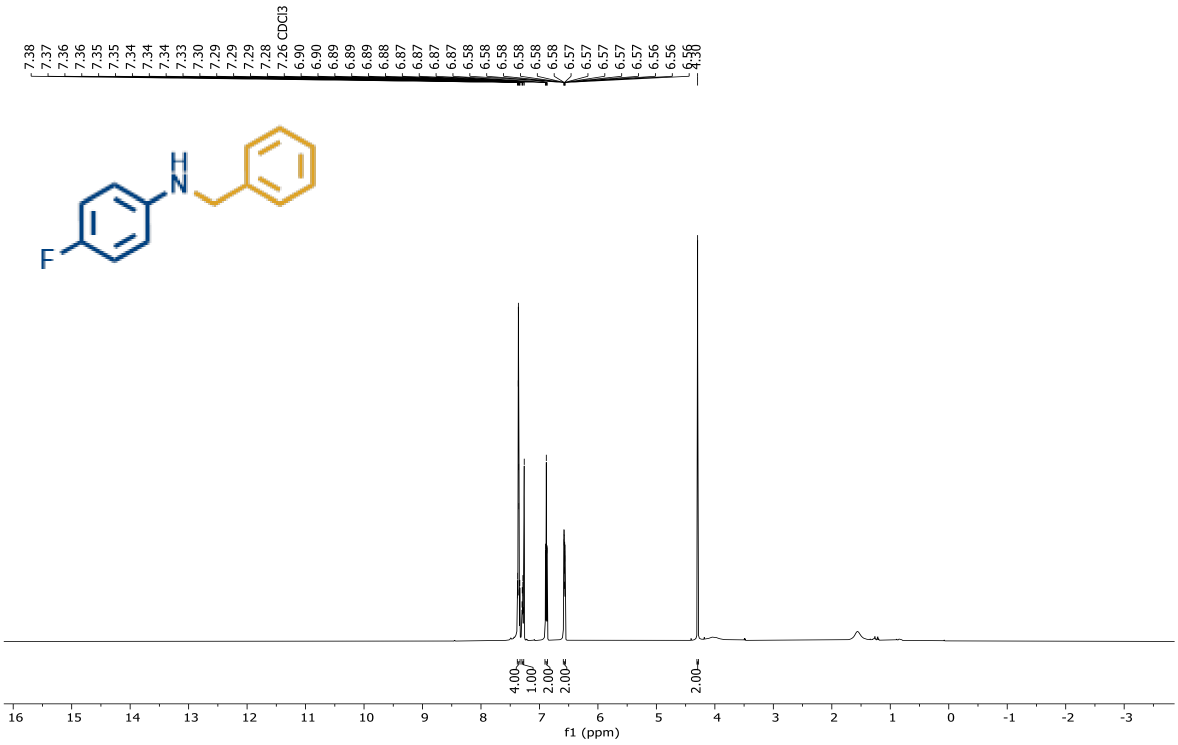

^{13}C NMR (151 MHz, $CDCl_3$) spectrum of ***N*-benzyl-4-fluoroaniline (3gi).**

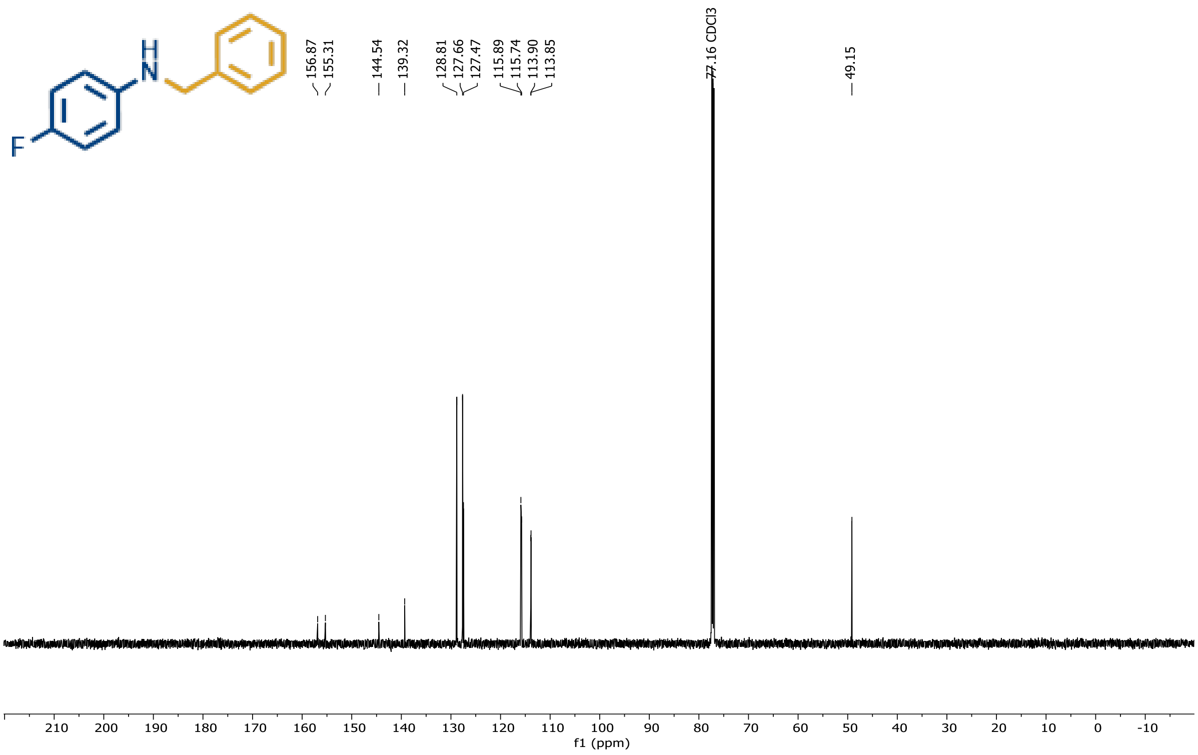

^{1}H NMR (600 MHz, $CDCl_3$) spectrum of **3-chloro-*N*-pentylaniline (3ha).**

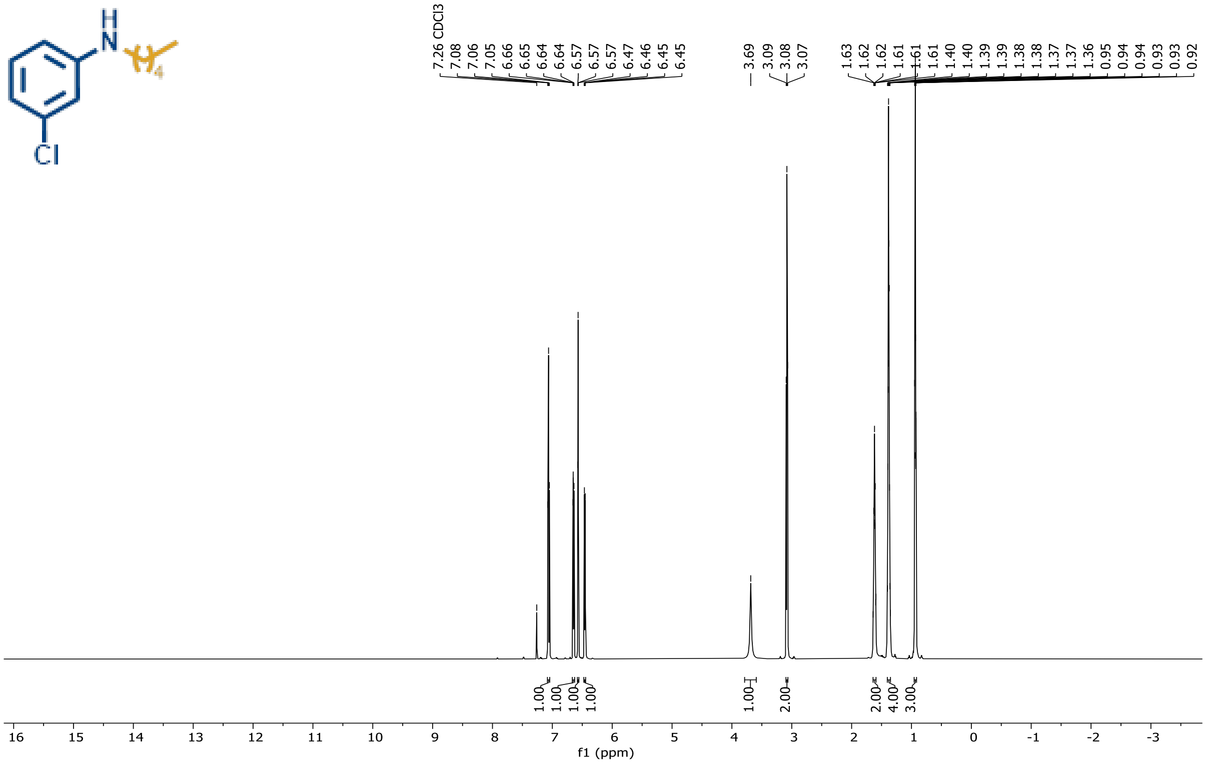

^{13}C NMR (151 MHz, $CDCl_3$) spectrum of **3-chloro-*N*-pentylaniline (3ha).**

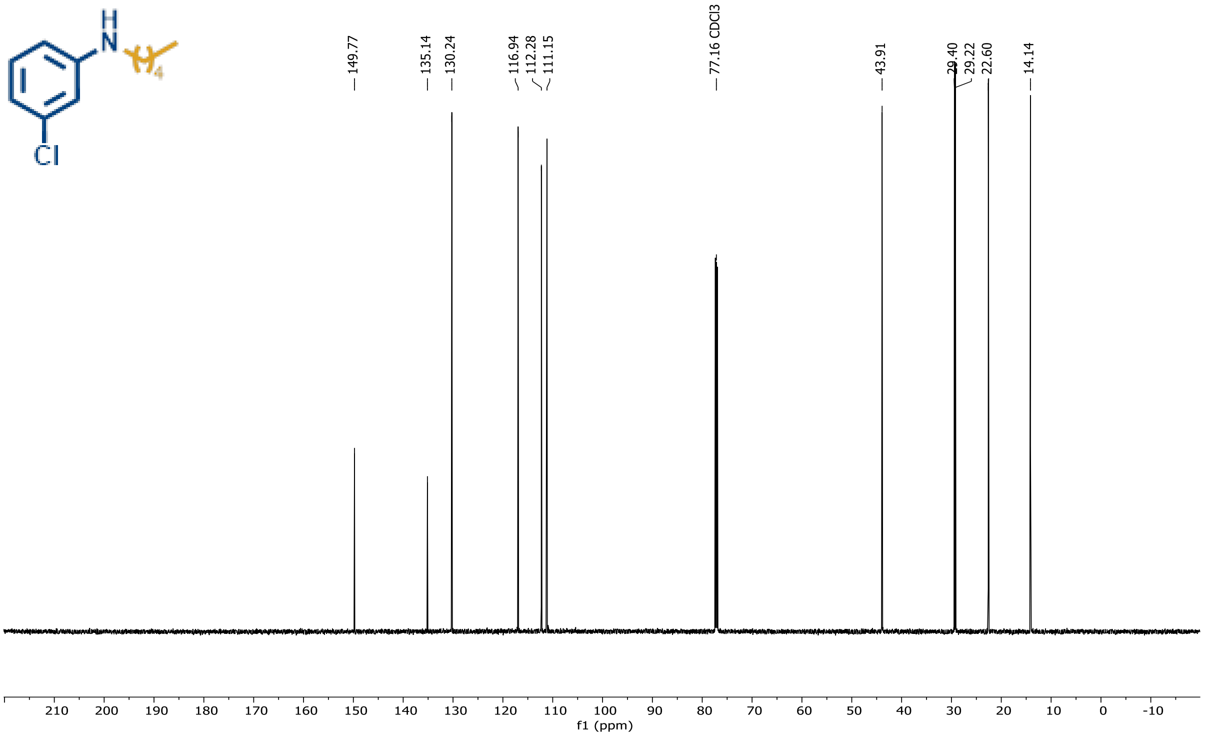

^{1}H NMR (600 MHz, $CDCl_3$) spectrum of **3-chloro-*N*-ethylaniline (3hb).**

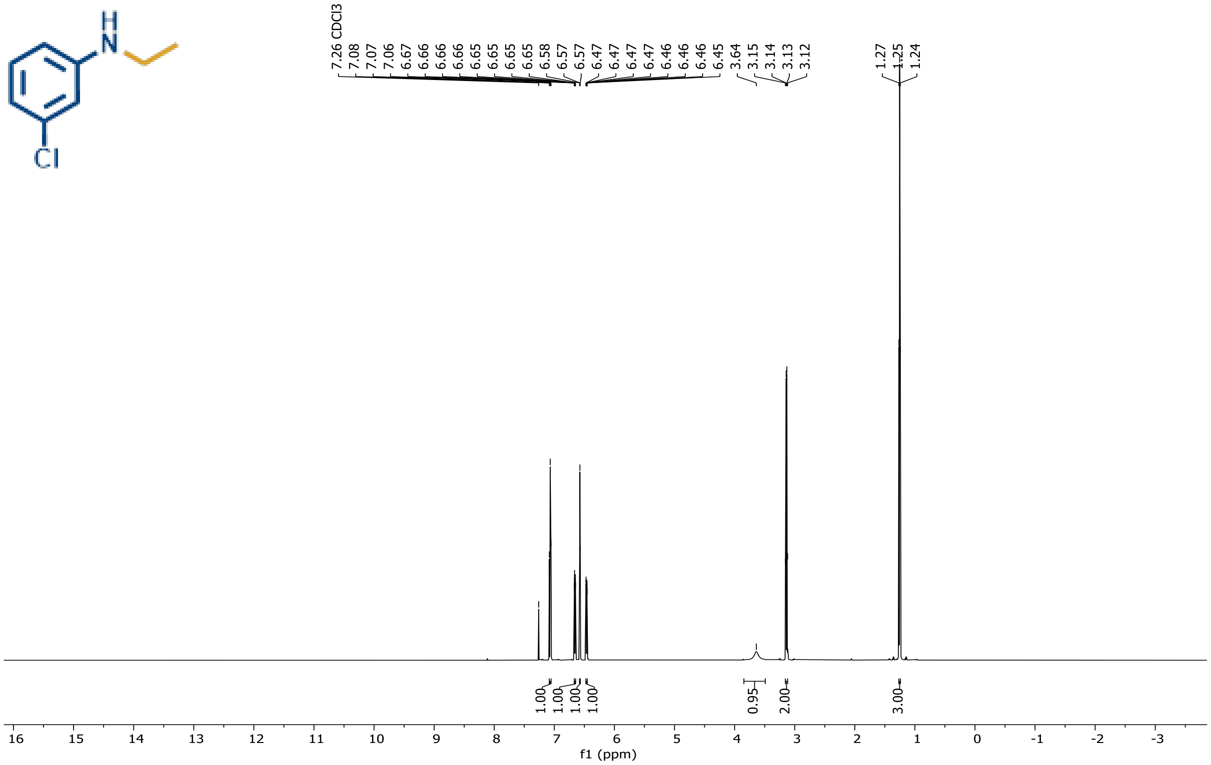

^{13}C NMR (151 MHz, $CDCl_3$) spectrum of **3-chloro-*N*-ethylaniline (3hb).**

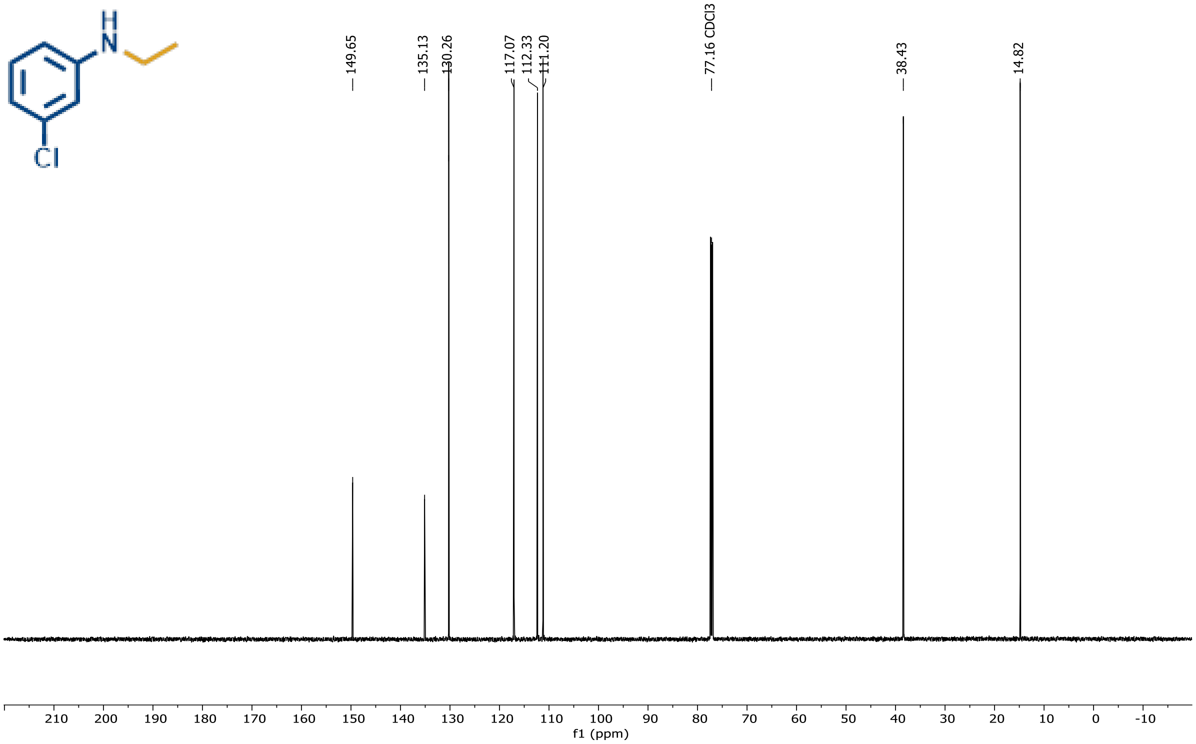

^{1}H NMR (600 MHz, $CDCl_3$) spectrum of ***N*-butyl-3-chloroaniline (3hd).**

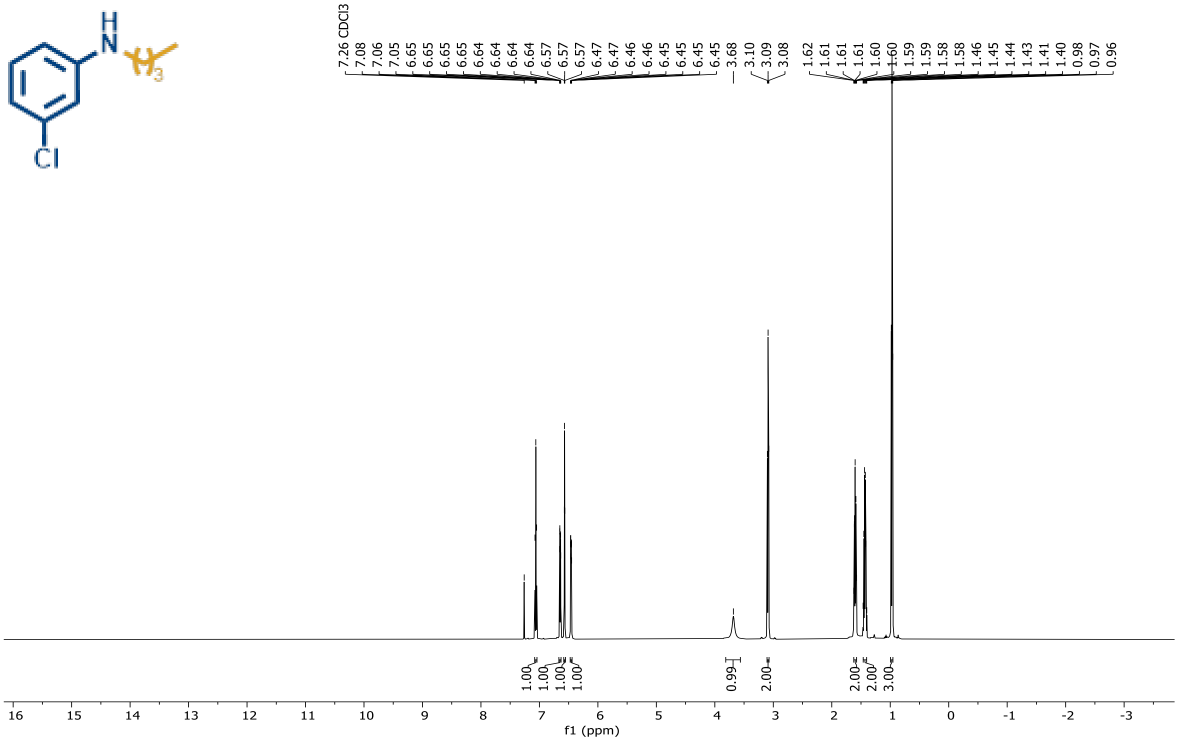

^{13}C NMR (151 MHz, $CDCl_3$) spectrum of ***N*-butyl-3-chloroaniline (3hd).**

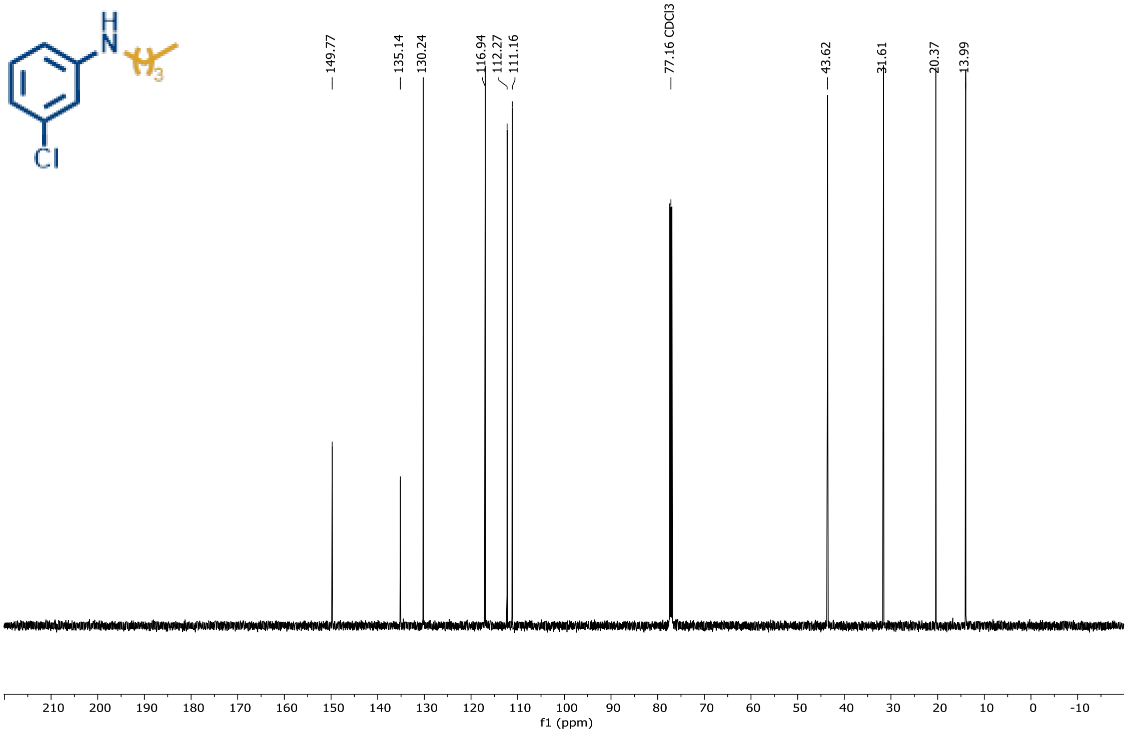

^{1}H NMR (600 MHz, $CDCl_3$) spectrum of **3-methyl-*N*-pentylaniline (3ia).**

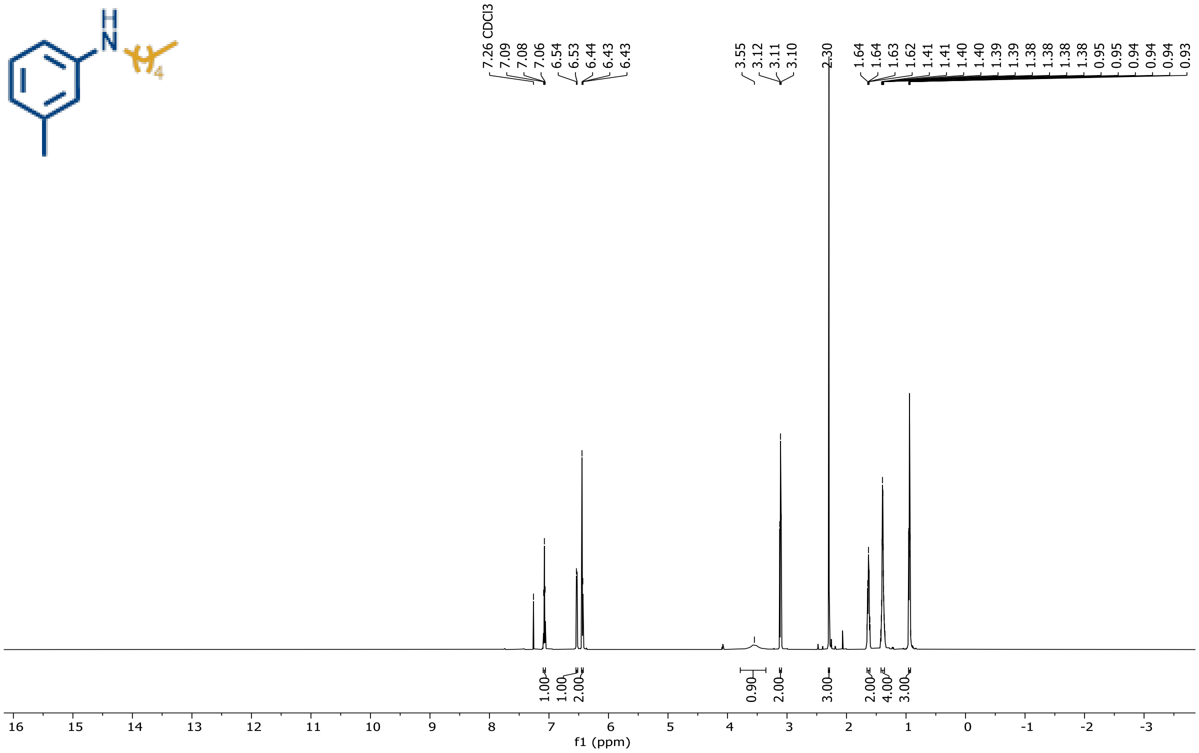

^{13}C NMR (151 MHz, $CDCl_3$) spectrum of **3-methyl-*N*-pentylaniline (3ia).**

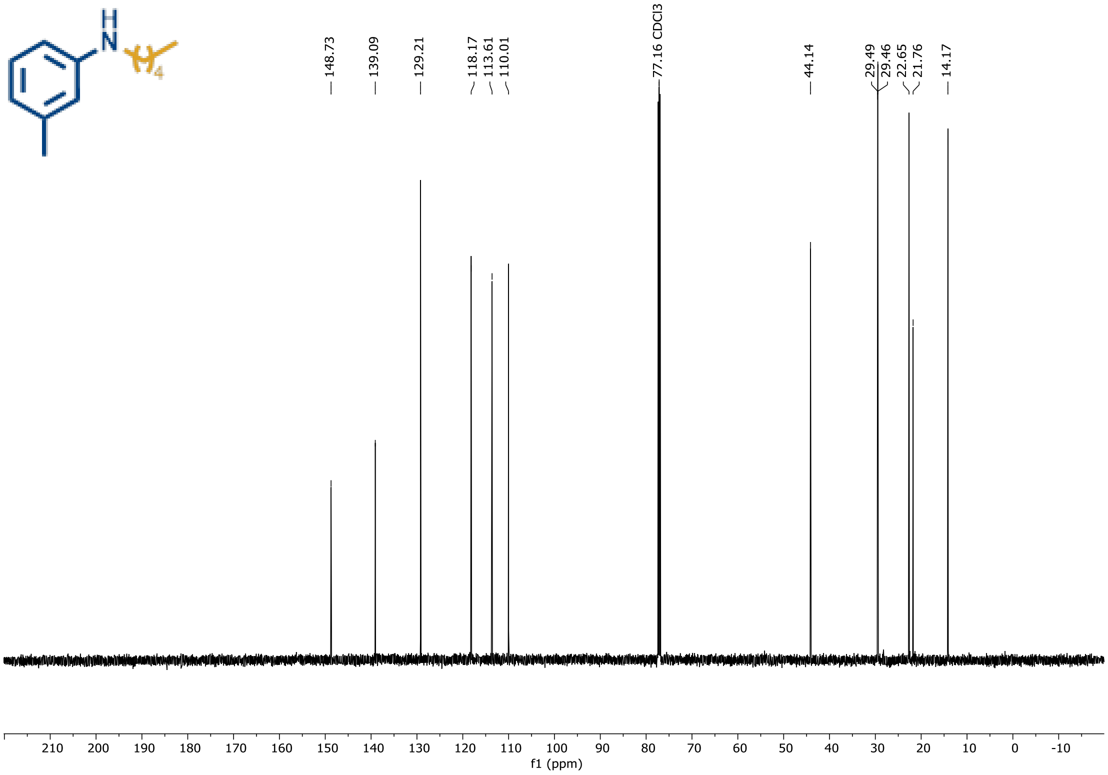

^{1}H NMR (600 MHz, $CDCl_3$) spectrum of **2-methoxy-*N*-pentylaniline (3ja).**

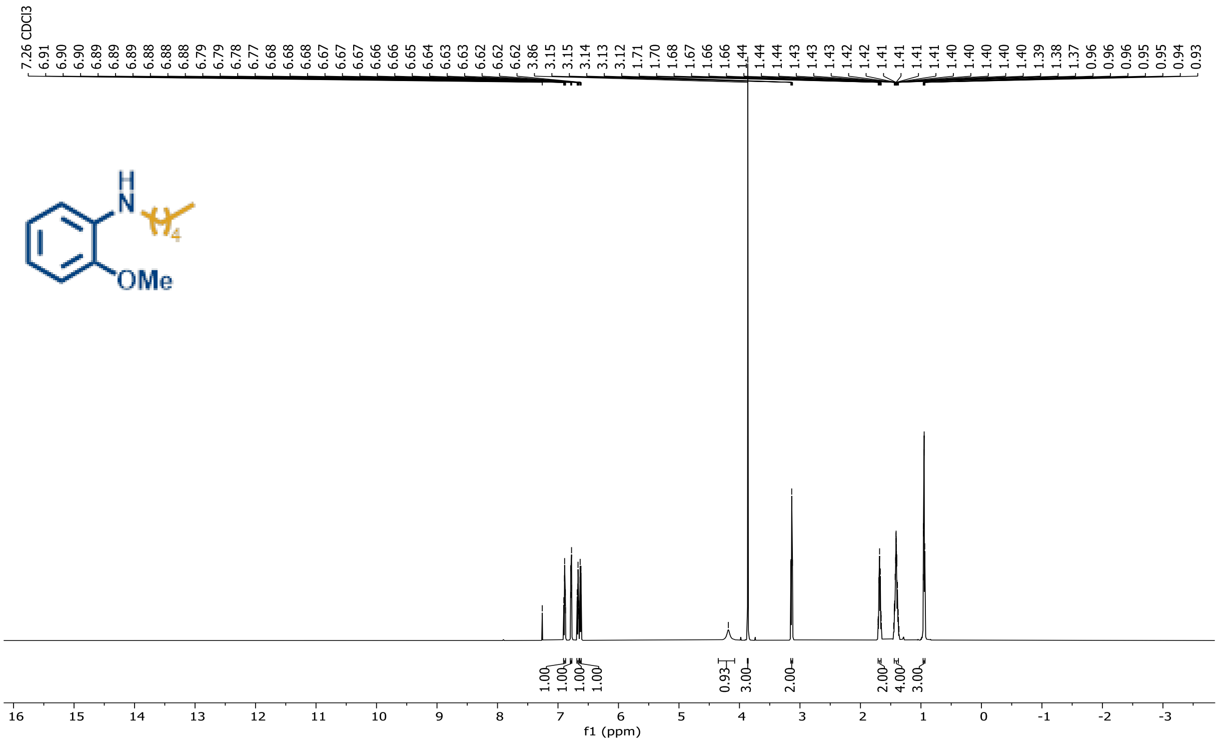

^{13}C NMR (151 MHz, $CDCl_3$) spectrum of **2-methoxy-*N*-pentylaniline (3ja).**

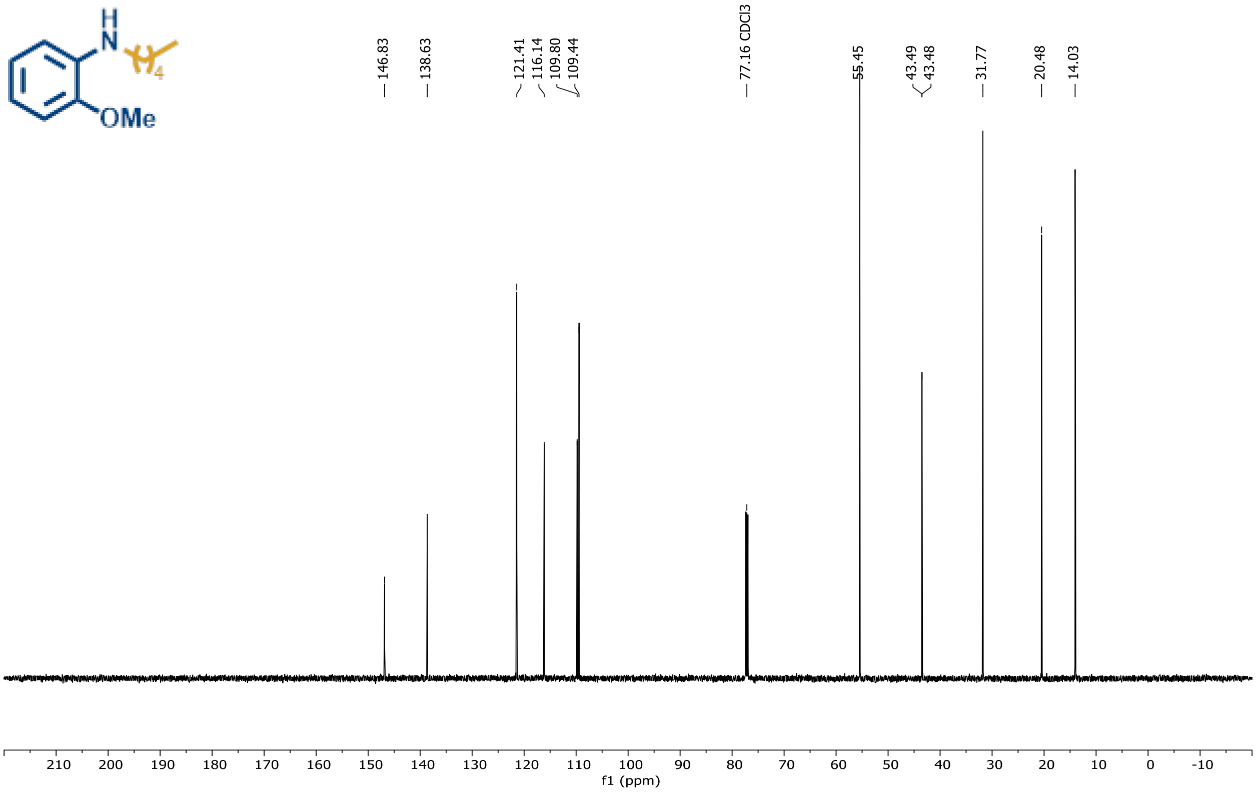

^{1}H NMR (600 MHz, $CDCl_3$) spectrum of ***N*-ethyl-2-methoxyaniline (3jb).**

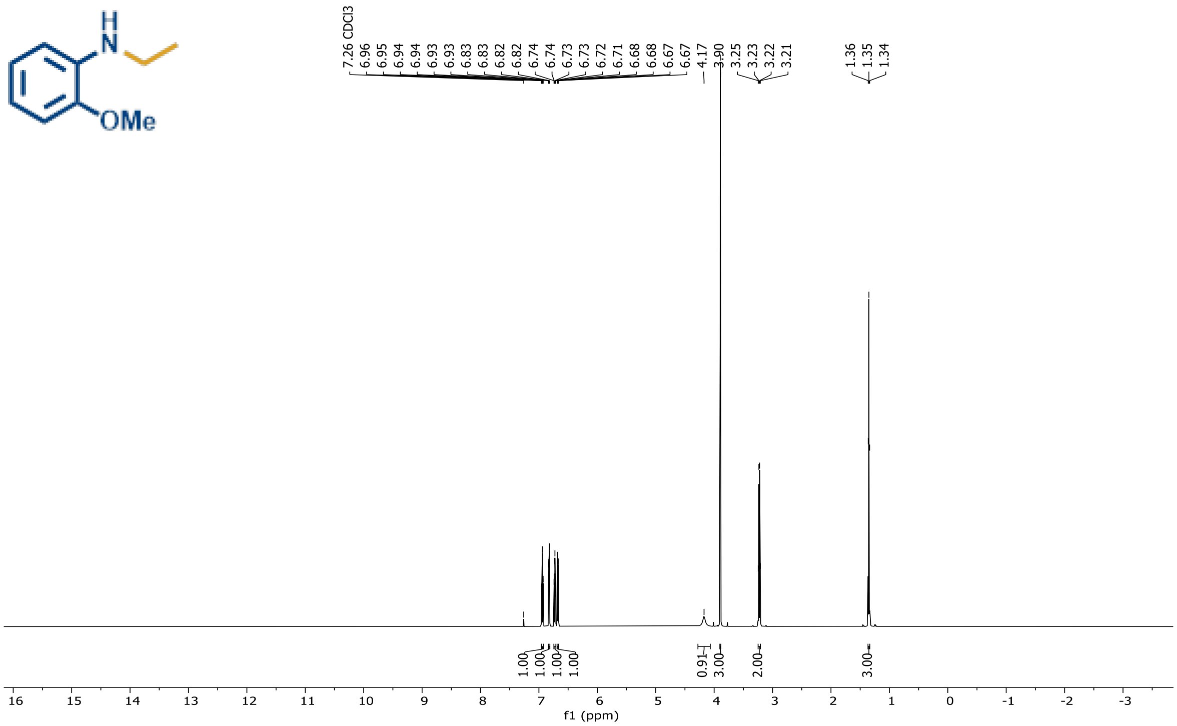

^{13}C NMR (151 MHz, $CDCl_3$) spectrum of ***N*-ethyl-2-methoxyaniline (3jb).**

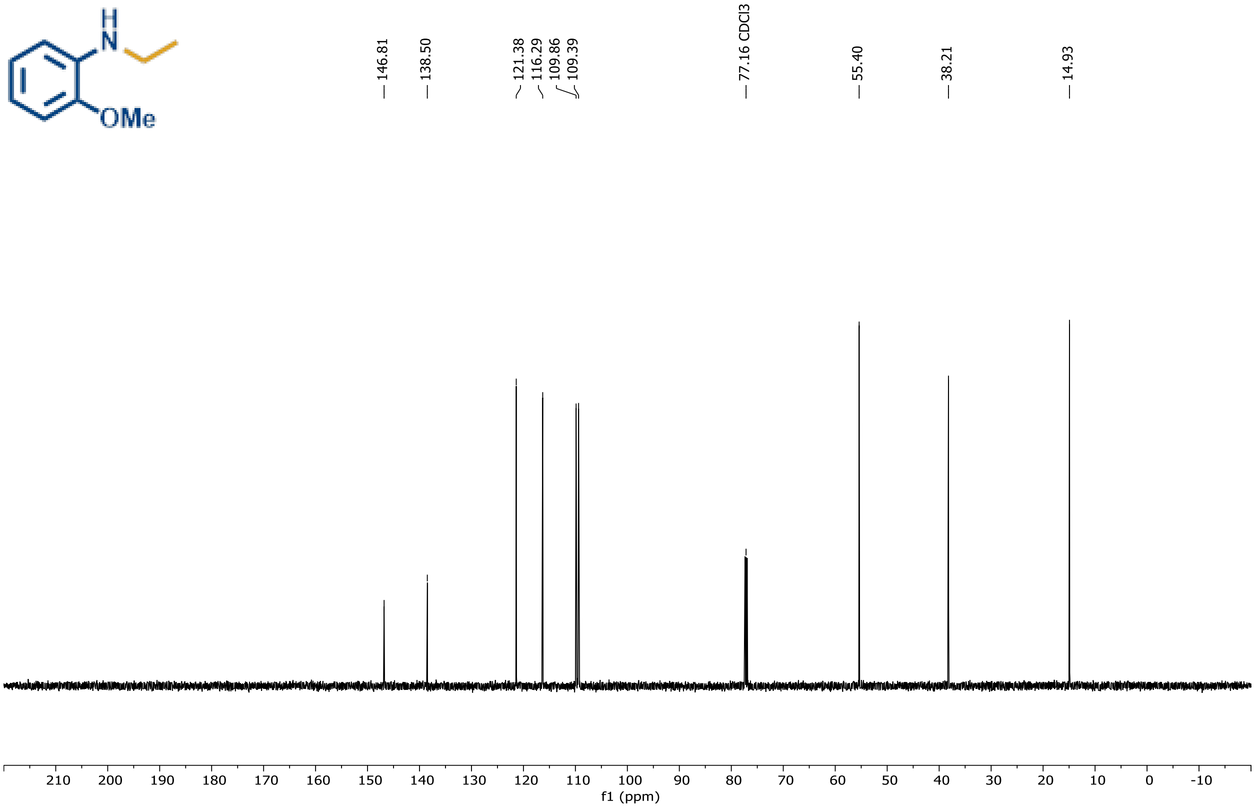

^{1}H NMR (600 MHz, $CDCl_3$) spectrum of ***N*-butyl-2-methoxyaniline (3jd).**

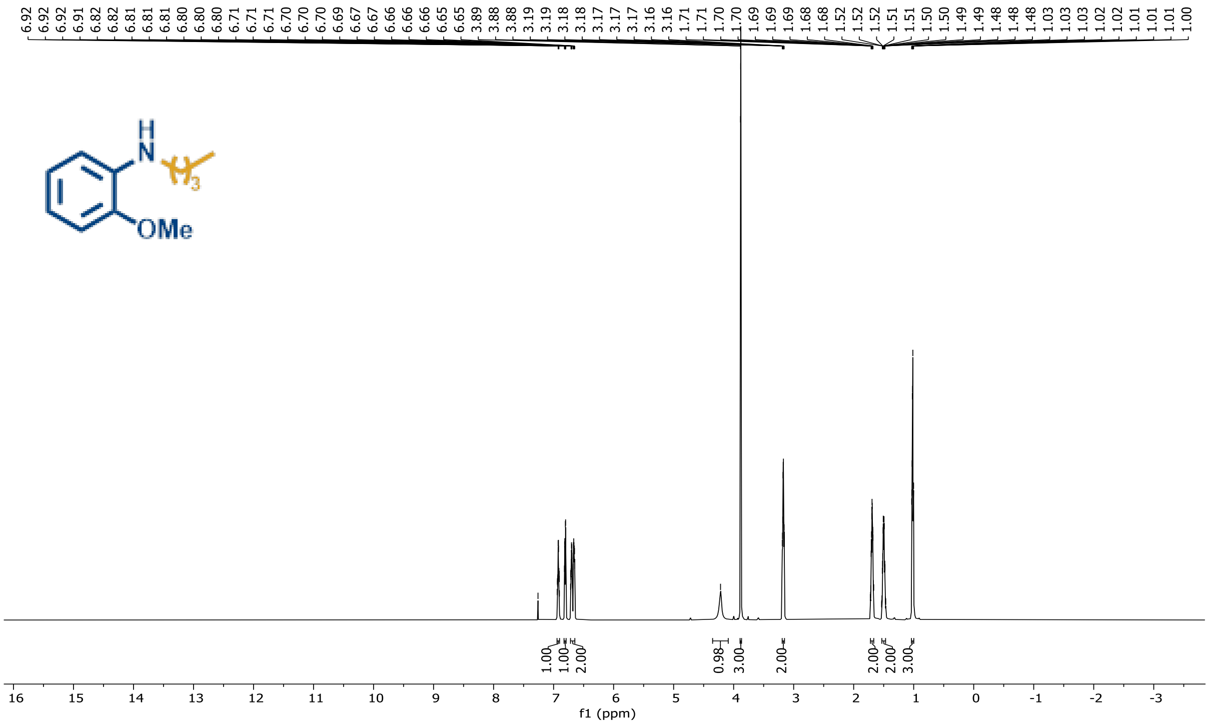

^{13}C NMR (151 MHz, $CDCl_3$) spectrum of ***N*-butyl-2-methoxyaniline (3jd).**

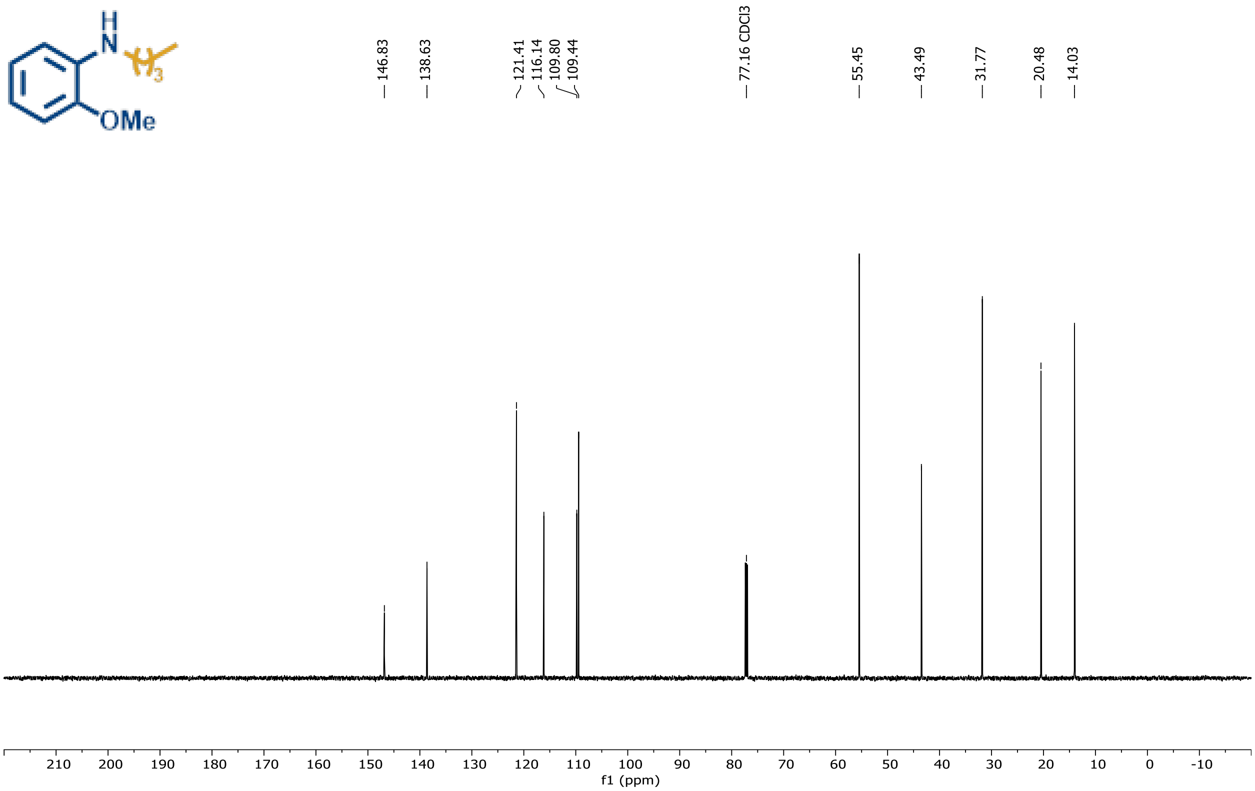

^{1}H NMR (600 MHz, $CDCl_3$) spectrum of **2-fluoro-*N*-pentylaniline (3ka).**

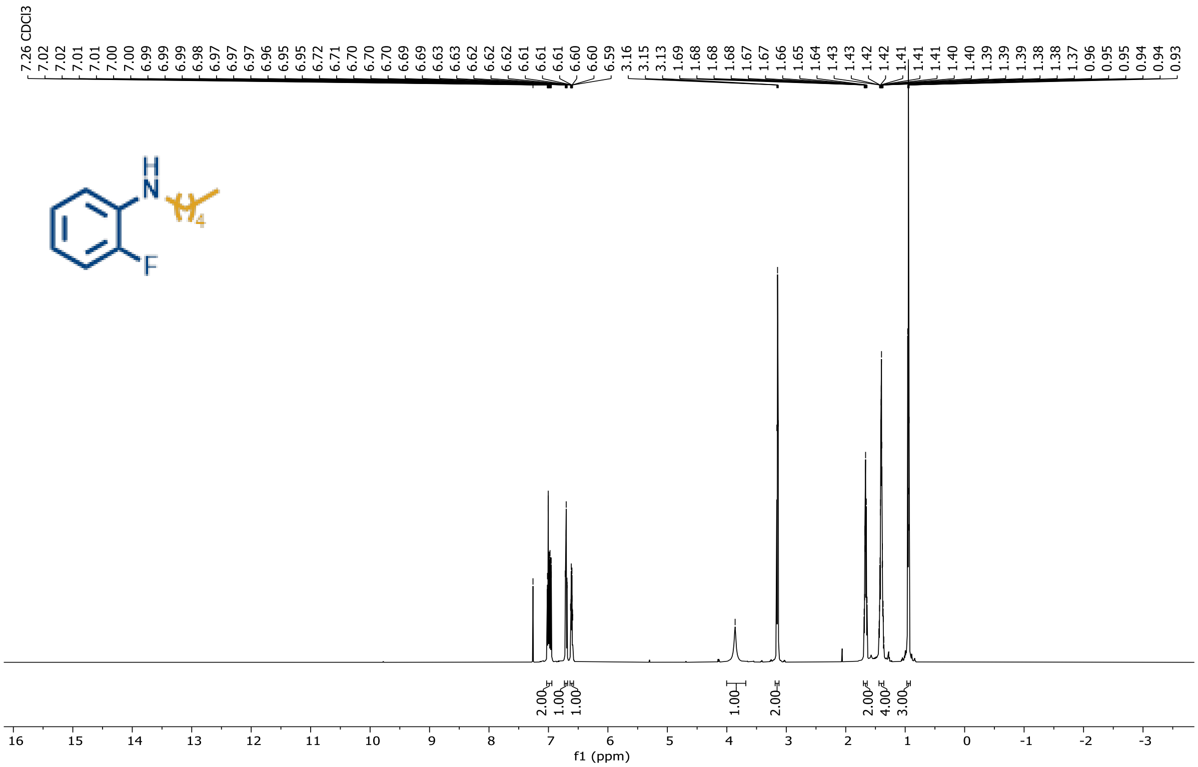

^{13}C NMR (151 MHz, $CDCl_3$) spectrum of **2-fluoro-*N*-pentylaniline (3ka).**

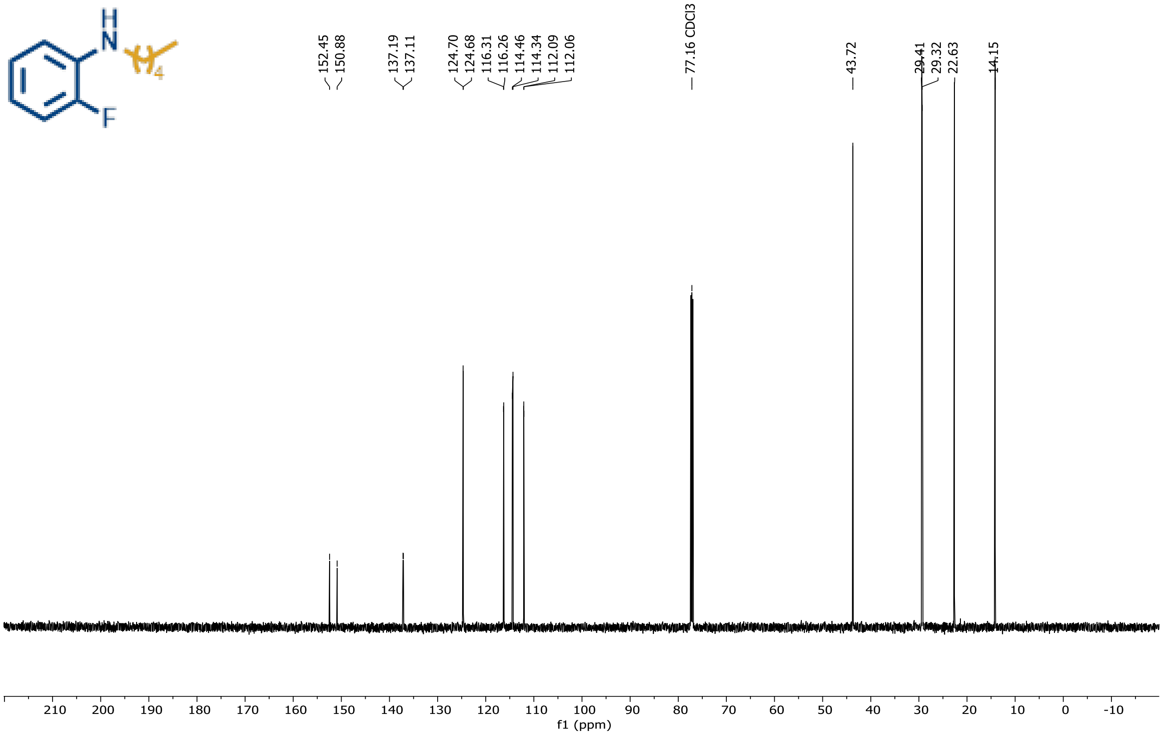

^{1}H NMR (600 MHz, CDCl₃) spectrum of **(E)-*N*-(4-methoxyphenyl)-1-(4-nitrophenyl)methanimine (3am).**

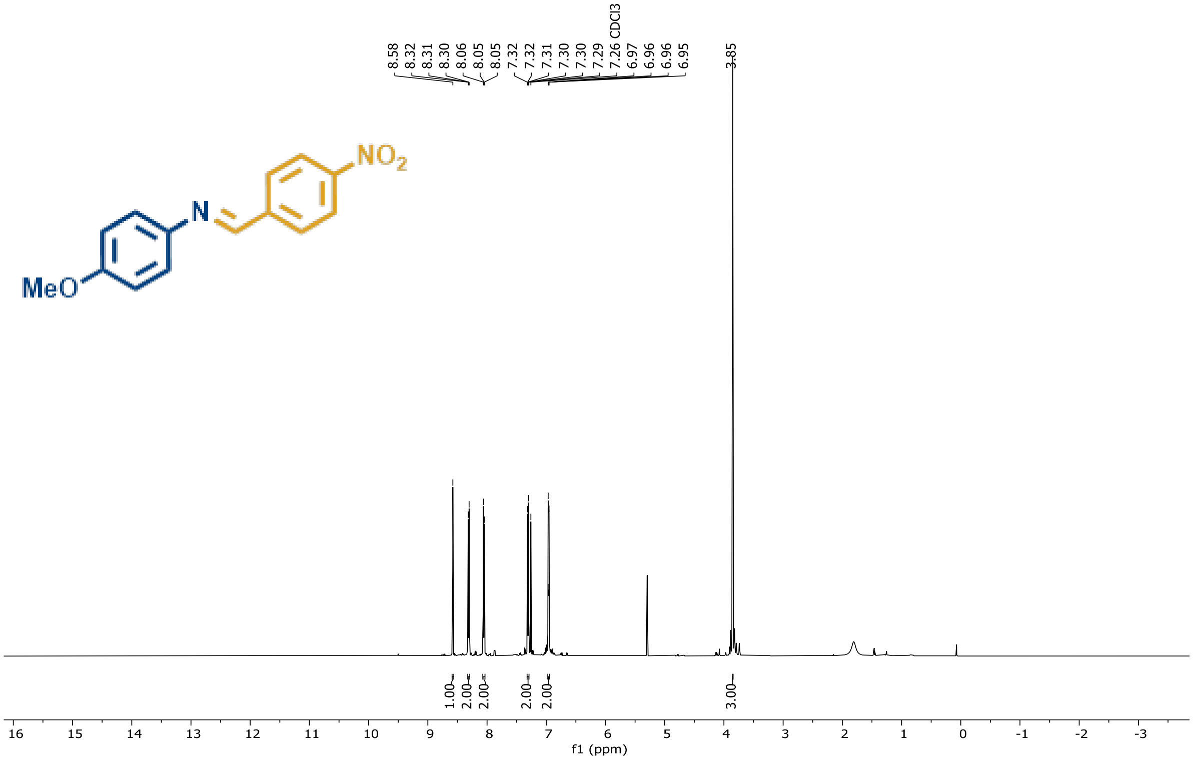

^{1}H NMR (600 MHz, $CDCl_3$) spectrum of ***N*-phenethylpentan-1-amine (5aa).**

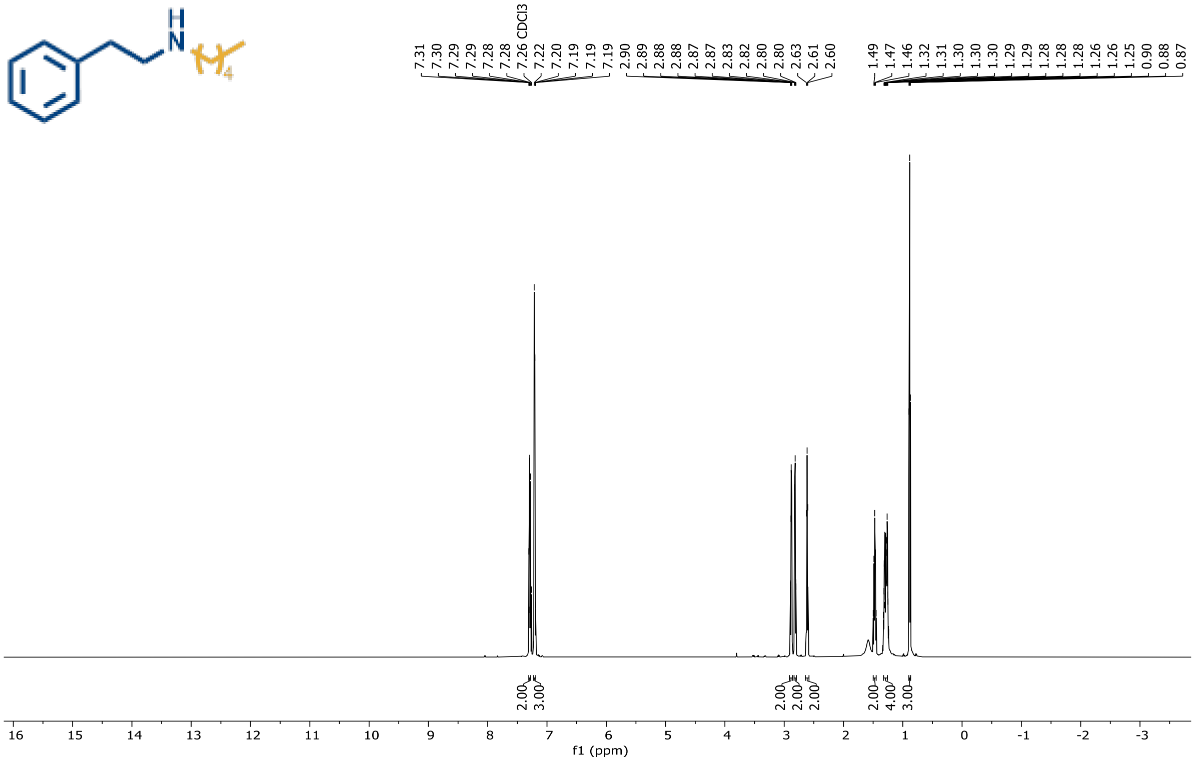

^{13}C NMR (151 MHz, $CDCl_3$) spectrum of ***N*-phenethylpentan-1-amine (5aa).**

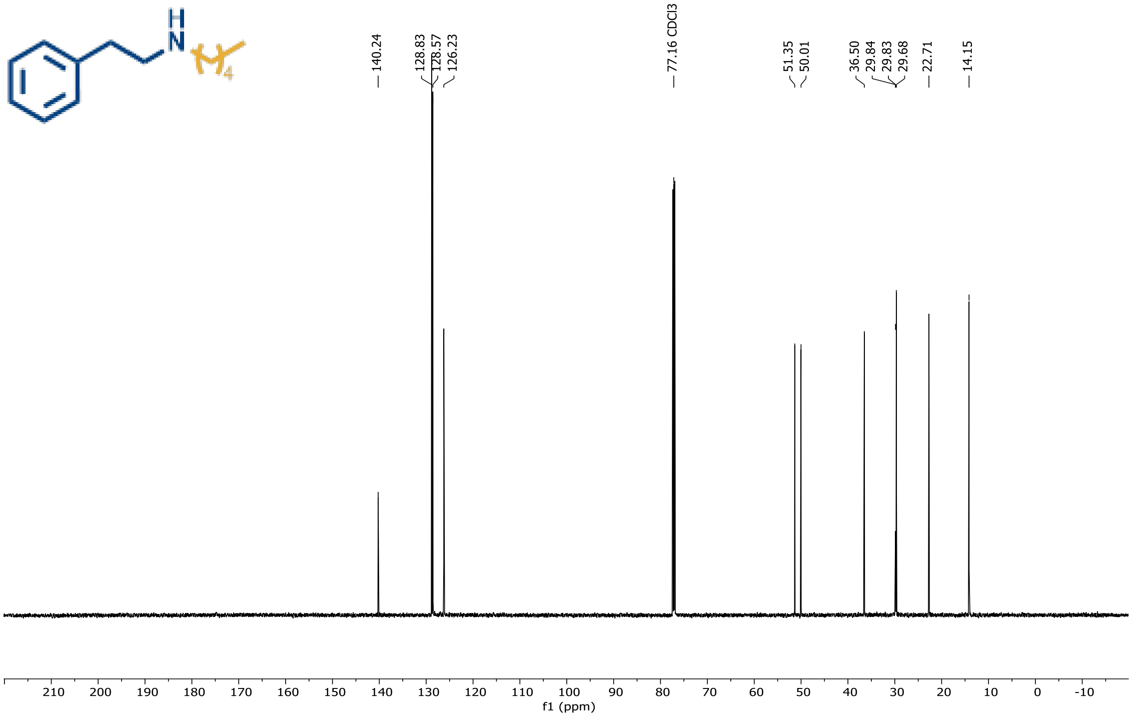

^{1}H NMR (600 MHz, $CDCl_3$) spectrum of ***N*-phenethylhexan-1-amine (5ac).**

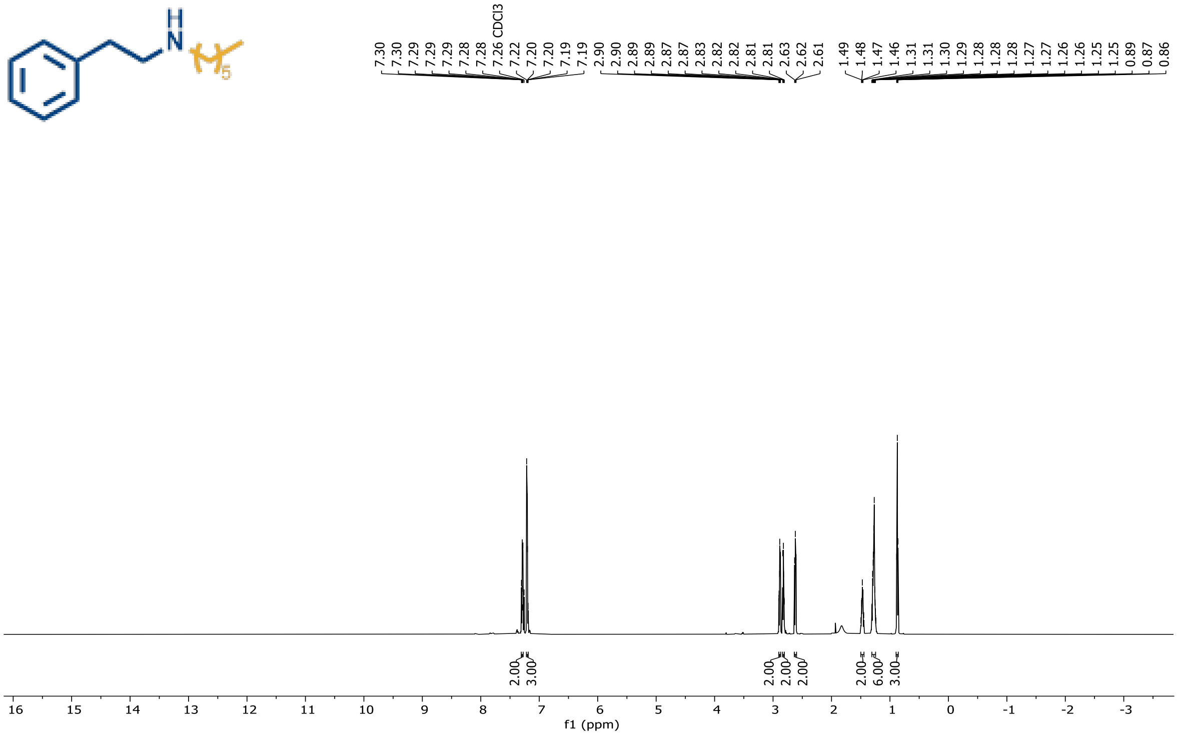

^{13}C NMR (151 MHz, $CDCl_3$) spectrum of ***N*-phenethylhexan-1-amine (5ac).**

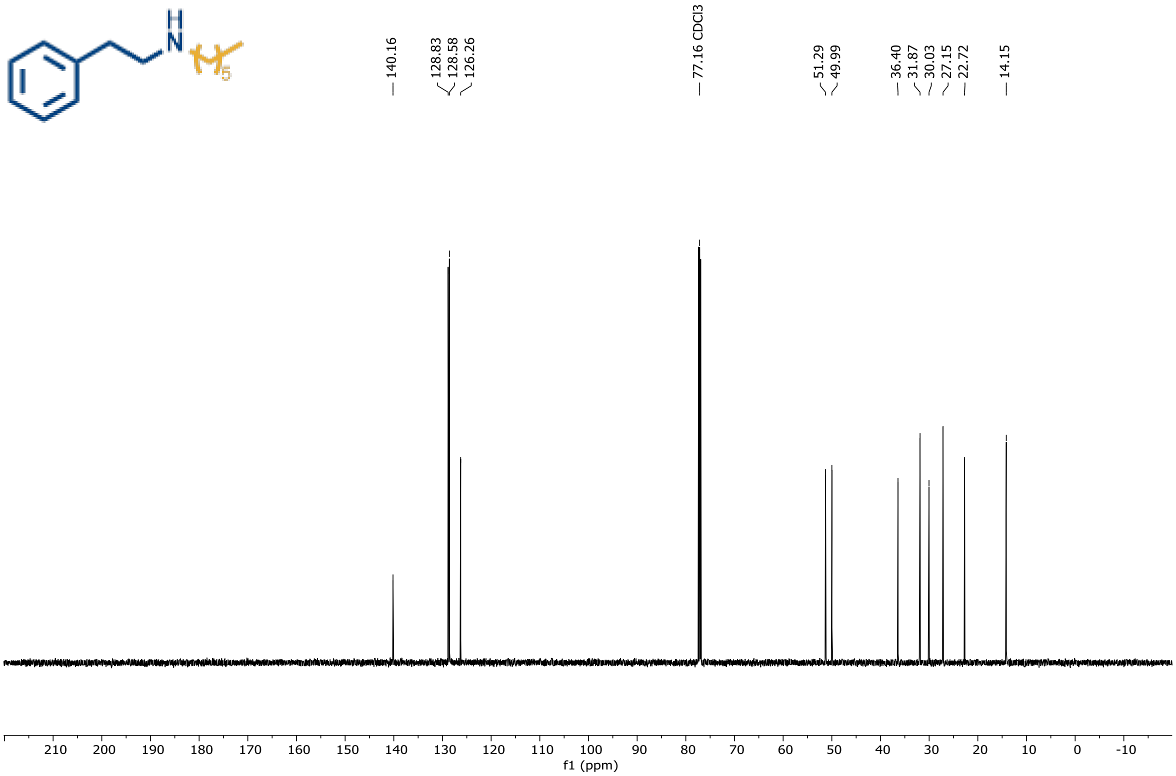

^{1}H NMR (600 MHz, $CDCl_3$) spectrum of ***N*-phenethyldodecan-1-amine (5ah).**

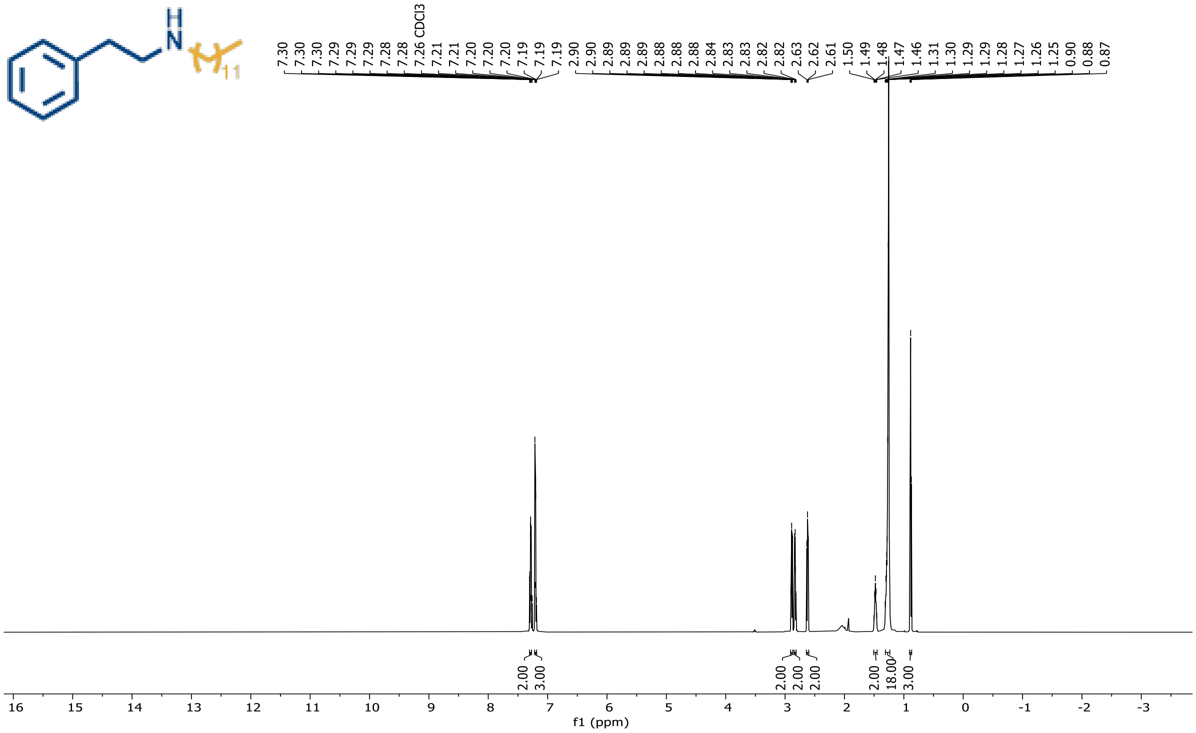

^{13}C NMR (151 MHz, $CDCl_3$) spectrum of ***N*-phenethyldodecan-1-amine (5ah).**

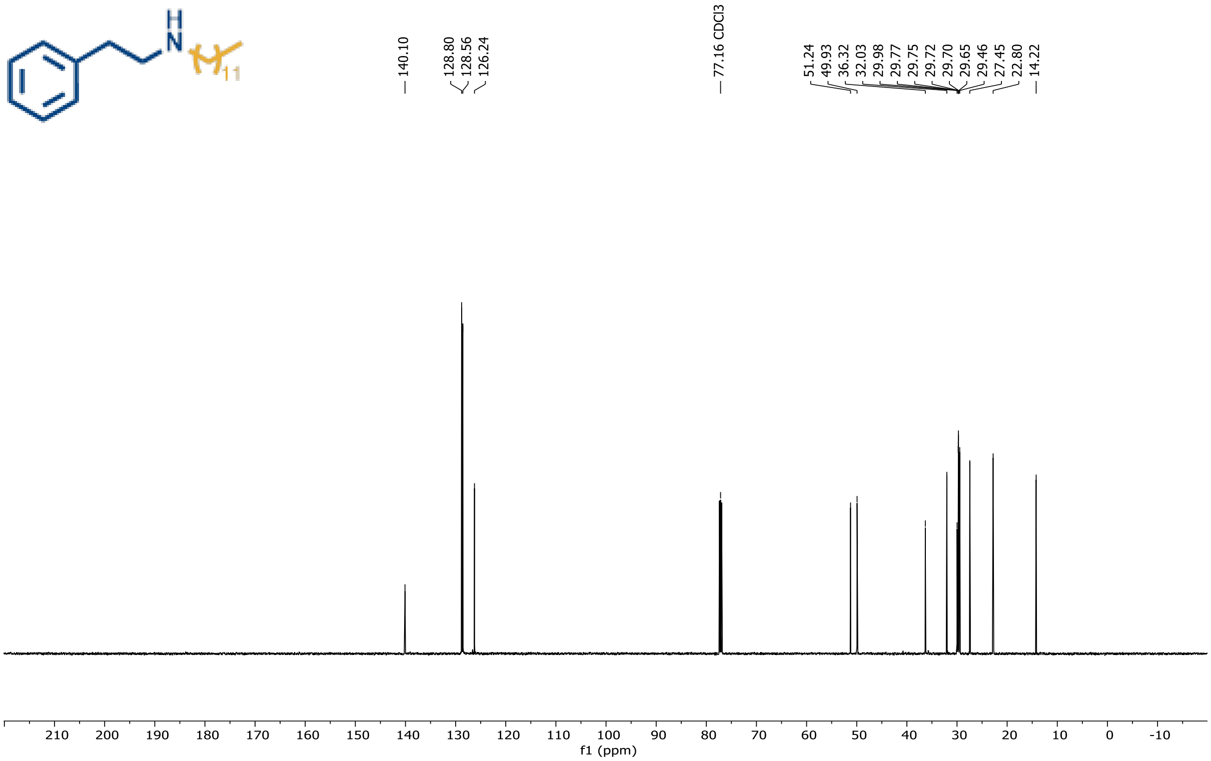

^{1}H NMR (600 MHz, $CDCl_3$) spectrum of **1H-Indole (6).**

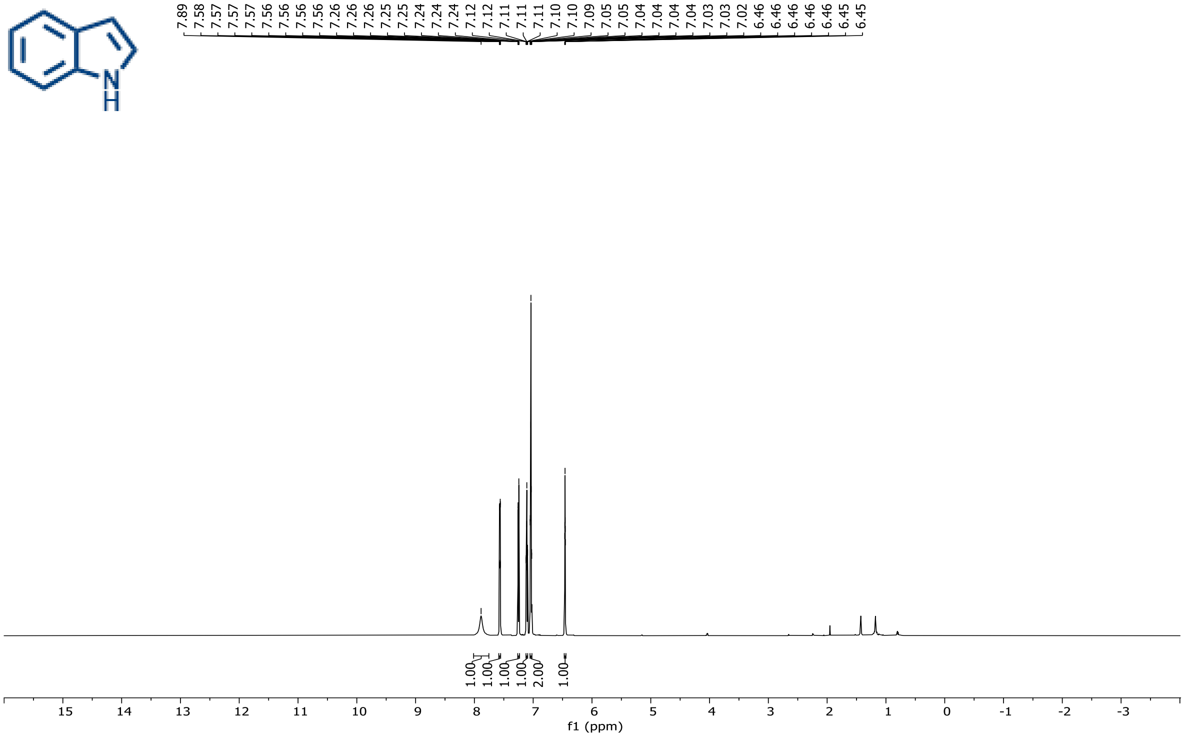

^{13}C NMR (151 MHz, $CDCl_3$) spectrum of **1H-Indole (6).**

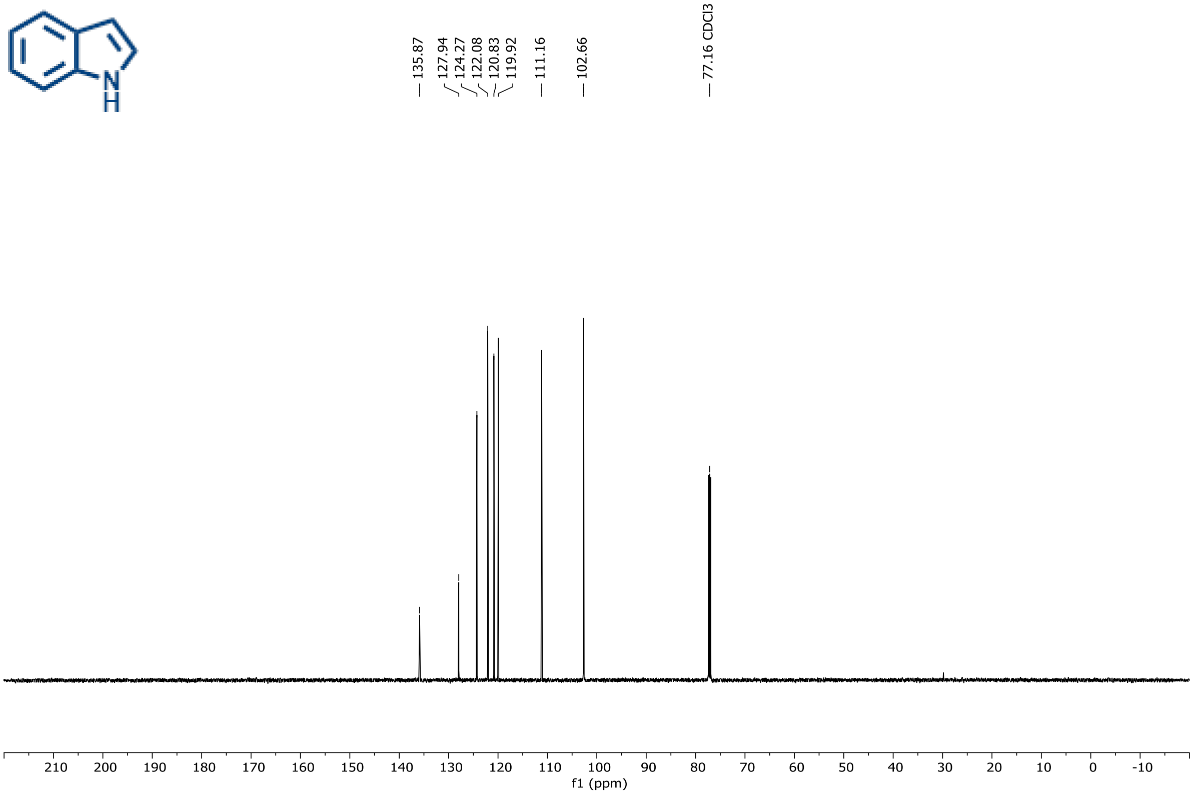

^{1}H NMR (600 MHz, $CDCl_3$) spectrum of **2-phenyl-1H-benzimidazole (7).**

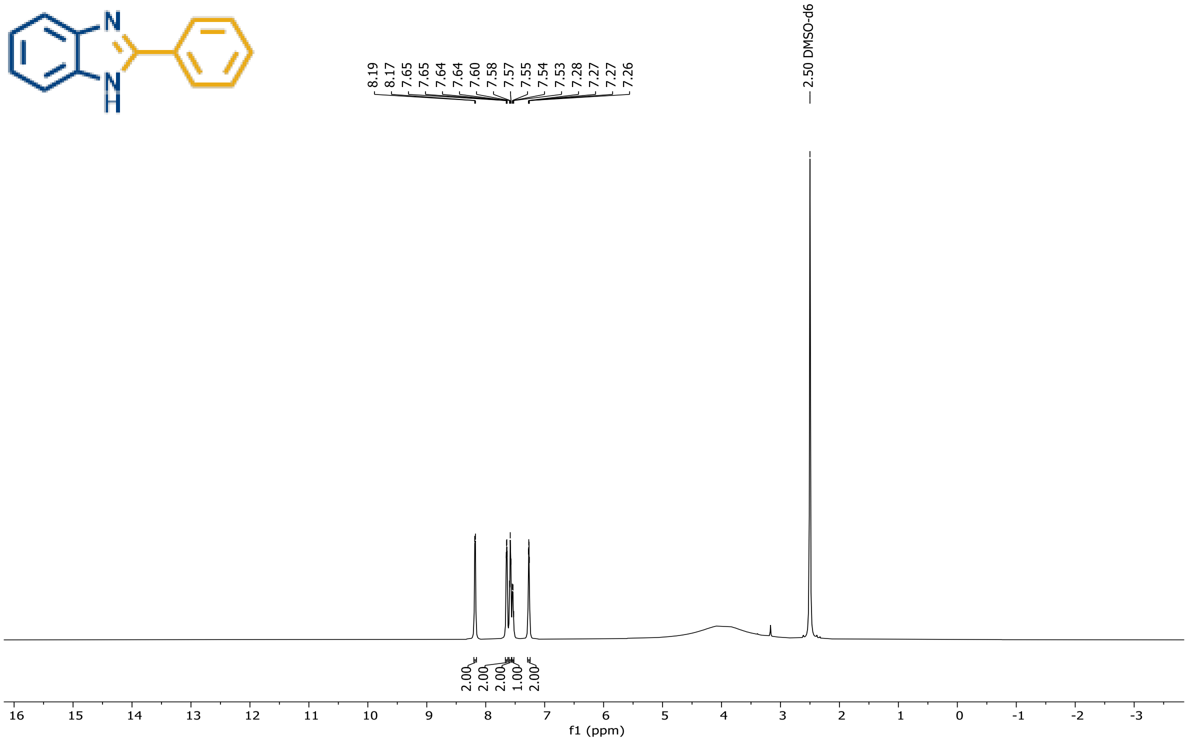

^{13}C NMR (151 MHz, $CDCl_3$) spectrum of **2-phenyl-1H-benzimidazole (7).**

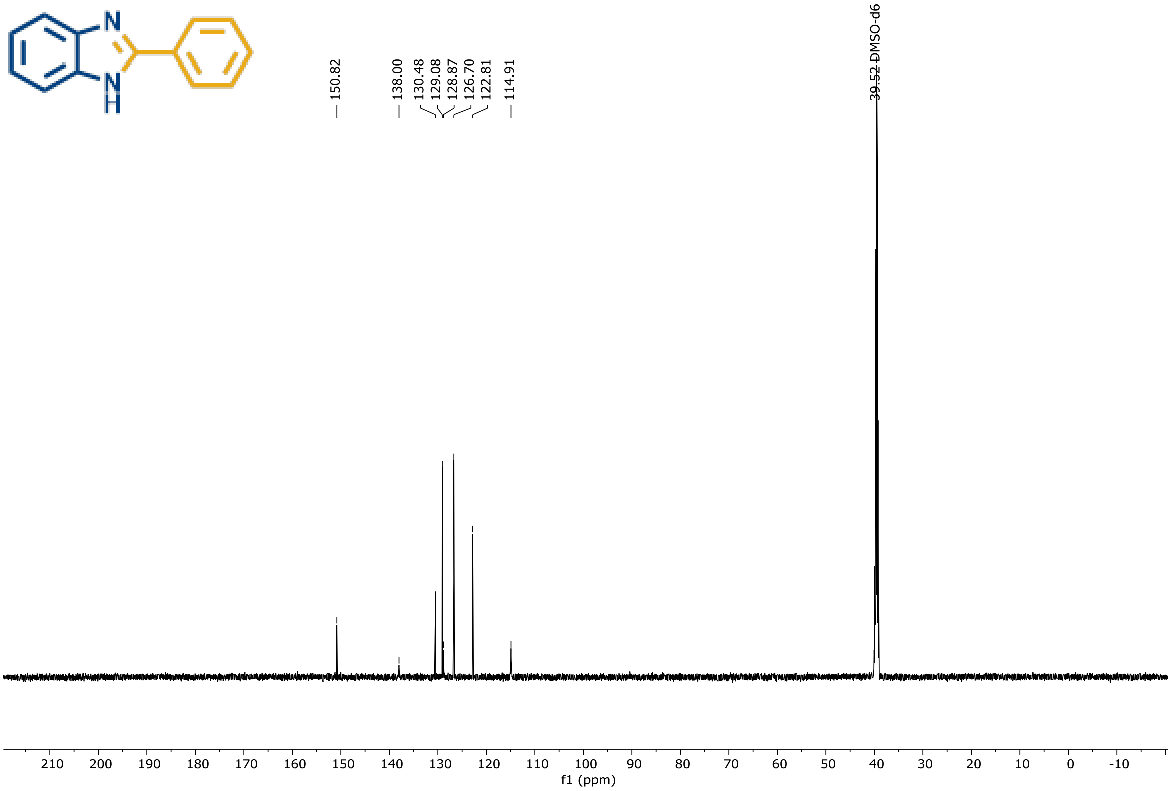

REFERENCES

[1] R. C. A. Silva, E. G. R. de Sousa, J. L. Mazzei, E. M. de Carvalho, *J. Pharm. Biomed. Anal.* **2022**, *210*, 114585.

[2] R. Mocci, L. Atzori, W. Baratta, L. De Luca, A. Porcheddu, *RSC Adv.* **2023**, *13*, 34847-34851.

[3] X. Xie, T. Y. Zhang, Z. Zhang, *J. Org. Chem.* **2006**, *71*, 6522-6529.

[4] R. Nacario, S. Kotakonda, D. M. D. Fouchard, L. M. V. Tillekeratne, R. A. Hudson, *Org. Lett.* **2005**, *7*, 471-474.

[5] D. Hollmann, S. Bähn, A. Tillack, M. Beller, *Angew. Chem. Int. Ed.* **2007**, *46*, 8291-8294.

[6] A. J. A. Watson, A. C. Maxwell, J. M. J. Williams, *J. Org. Chem.* **2011**, *76*, 2328-2331.

[7] Y. Wei, C. Zhao, Q. Xuan, Q. Song, *Org. Chem. Front.* **2017**, *4*, 2291-2295.

[8] C. Braun, M. Nieger, S. Bräse, L. L. Schafer, *ChemCatChem* **2019**, *11*, 5264-5268.

[9] a) K.-i. Shimizu, K. Shimura, M. Nishimura, A. Satsuma, *RSC Adv.* **2011**, *1*, 1310-1317; b) A. K. Jaladi, H. Kim, J. H. Lee, W. K. Shin, H. Hwang, D. K. An, *New J. Chem.* **2019**, *43*, 16524-16529.

[10] H. C. Maytum, J. Francos, D. J. Whatrup, J. M. J. Williams, *Chem. Asian J.* **2010**, *5*, 538-542.

[11] S. Fleischer, S. Zhou, K. Junge, M. Beller, *Chem. Asian J.* **2011**, *6*, 2240-2245.

[12] H. Kato, I. Shibata, Y. Yasaka, S. Tsunoi, M. Yasuda, A. Baba, *Chem. Commun.* **2006**, 4189-4191.

[13] aM.-C. Fu, R. Shang, W.-M. Cheng, Y. Fu, *Angew. Chem. Int. Ed.* **2015**, *54*, 9042-9046; bM. Barbero, S. Cadamuro, S. Dughera, G. Ghigo, *Eur. J. Org. Chem.* **2008**, *2008*, 862-868.

[14] A. Bartoszewicz, R. Marcos, S. Sahoo, A. K. Inge, X. Zou, B. Martín-Matute, *Chem. Eur. J.* **2012**, *18*, 14510-14519.

[15] D. Hollmann, S. Bähn, A. Tillack, M. Beller, *Chem. Commun.* **2008**, 3199-3201.

[16] F. Kallmeier, R. Fertig, T. Irrgang, R. Kempe, *Angew. Chem. Int. Ed.* **2020**, *59*, 11789-11793.

[17] N. Sakai, M. Takeoka, T. Kumaki, H. Asano, T. Konakahara, Y. Ogiwara, *Tetrahedron Lett.* **2015**, *56*, 6448-6451.

[18] D. Jiang, H. Fu, Y. Jiang, Y. Zhao, *J. Org. Chem.* **2007**, *72*, 672-674.

[19] T. Kubo, C. Katoh, K. Yamada, K. Okano, H. Tokuyama, T. Fukuyama, *Tetrahedron* **2008**, *64*, 11230-11236.

[20] a) H. Zhang, Q. Cai, D. Ma, *J. Org. Chem.* **2005**, *70*, 5164-5173; b) S. Elangovan, J. Neumann, J.-B. Sortais, K. Junge, C. Darcel, M. Beller, *Nat. Commun.* **2016**, *7*, 12641.

[21] T. Yan, B. L. Feringa, K. Barta, *Nat. Commun.* **2014**, *5*, 5602.

[22] I. Sorribes, J. R. Cabrero-Antonino, C. Vicent, K. Junge, M. Beller, *J. Am. Chem. Soc.* **2015**, *137*, 13580-13587.

[23] G. Song, Q. Li, J. Song, D.-Z. Nong, J. Dong, G. Li, J. Fan, C. Wang, D. Xue, *ACS Catal.* **2024**, *14*, 4968-4974.

[24] H. Sajiki, T. Ikawa, K. Hirota, *Org. Lett.* **2004**, *6*, 4977-4980.

[25] C. Ge, X. Sang, W. Yao, L. Zhang, D. Wang, *Green Chem.* **2018**, *20*, 1805-1812.

[26] J. S. Bennett, K. L. Charles, M. R. Miner, C. F. Heuberger, E. J. Spina, M. F. Bartels, T. Foreman, *Green Chem.* **2009**, *11*, 166-168.

[27] C. Guisado, J. E. Waterhouse, W. S. Price, M. R. Jorgensen, A. D. Miller, *Org. Biomol. Chem.* **2005**, *3*, 1049-1057.

[28] B. Liu, T. Roisnel, J.-F. Carpentier, Y. Sarazin, *Angew. Chem. Int. Ed.* **2012**, *51*, 4943-4946.
[29] M. L. Buil, M. A. Esteruelas, A. M. López, A. C. Mateo, *Organometallics* **2006**, *25*, 4079-4089.
[30] a) H. Prokopcová, C. O. Kappe, *J. Org. Chem.* **2007**, *72*, 4440-4448; b) L. Tang, X. Guo, Y. Yang, Z. Zha, Z. Wang, *Chem. Commun.* **2014**, *50*, 6145-6148.
